# 1,3- and 1,4-Benzdiyne equivalents for regioselective synthesis of polycyclic heterocycles†
†Electronic supplementary information (ESI) available. See DOI: 10.1039/c6sc00798h


**DOI:** 10.1039/c6sc00798h

**Published:** 2016-04-18

**Authors:** Takashi Ikawa, Shigeaki Masuda, Akira Takagi, Shuji Akai

**Affiliations:** a Graduate School of Pharmaceutical Sciences , Osaka University , 1-6 Yamadaoka , Suita , Osaka 565-0871 , Japan . Email: ikawa@phs.osaka-u.ac.jp ; Email: akai@phs.osaka-u.ac.jp

## Abstract

We have devised a novel 1,3-benzdiyne equivalent, capable of quadruple functionalization by sequential benzyne generation and reaction with arynophiles. The key features of this method include the chemoselective generation of two triple bonds in a single benzene ring under fluoride-mediated mild conditions, and the regiocontrol of each benzyne reaction by the substituent next to the triple bond. This method produced various benzo-fused heteroaromatic compounds *via* reactions with arynophiles, such as furans, azides, and diazo compounds. A validation of the method is given in the convergent synthesis of the antipsychotic drug risperidone. A similar strategy has also been applied to a 1,4-benzdiyne equivalent to construct linearly benzo-fused heteroaromatics.

## Abstract 2

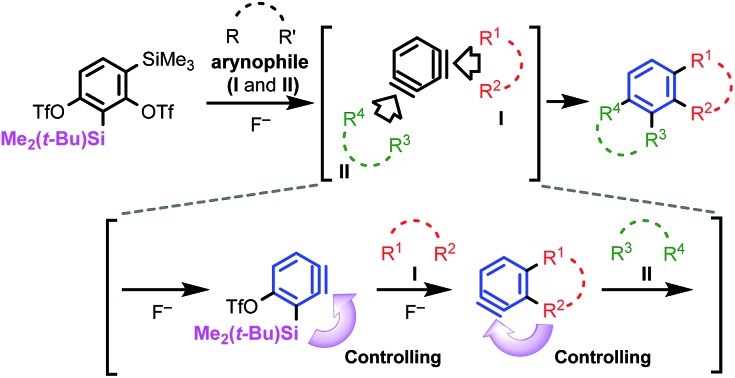
We developed a novel 1,3-benzdiyne equivalent sequentially generating two triple bonds in a single benzene ring and controlling the regiochemistry.

## Introduction

The reactions of benzynes with arynophiles are widely utilized for introducing substituents to adjacent carbons of benzene rings.^1^ The direct installation of fused rings onto benzenes is an advantage specific to the benzyne reaction and is not possible through other methods. Furthermore, a variety of new arynophiles have been recently reported, enriching the diversity of the method.^2^

The reactions of benzdiynes, possessing two triple bonds in a single benzene ring, and two arynophiles, would provide a few-step synthesis for the convergent preparation of multi-fused benzenes. However, benzdiynes are observed only under gas-phase conditions due to their extreme instability,^3^ and it would be impossible to react one with two different arynophiles for the synthesis of unsymmetrically fused benzene rings.

An alternative approach is to use benzdiyne equivalents, where two benzynes are generated sequentially in one pot to provide substituted acenes and polycyclic aromatic compounds. If we could control the regiochemistry of consecutive benzyne reactions, starting from benzdiyne equivalents, each with different arynophiles, we could produce a wide range of multi-ring fused unsymmetrical aromatic compounds.^4^ However, only a limited number of such reactions have been reported, and most of them require several steps for functional group transformations to generate the second benzyne.^5^,^6^ Therefore, the development of more sophisticated benzdiyne equivalents is needed to facilitate two-step sequential benzyne reactions. Crucial factors in the design of these benzdiyne equivalents include suitable functional groups which enable the generation of the second benzyne without further transformations, and a way to control the regiochemistry of each benzyne reaction. The work of Suzuki *et al.* involving their original 1,4-benzdiyne equivalent meets these requirements, which uses *n*-butyllithum to generate the benzynes.6*b* Very recently, Peña *et al.* have demonstrated that triple bonds were sequentially generated twice under fluoride-mediated mild conditions from 1,4-benzdiyne equivalents and reacted with two different arynophiles in both stepwise and one-pot manners.^7^ In contrast, there have been no reports of a suitable 1,3-benzdiyne equivalent.^5^,^8^,^9^

**Scheme 1:**
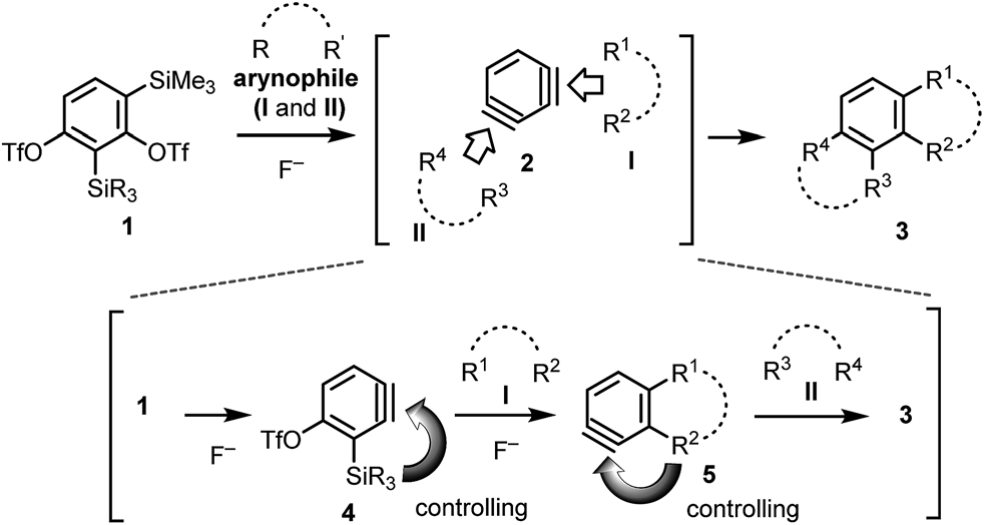
Design of benzdiyne equivalent **1** that can sequentially generate two triple bonds in a single benzene ring and control the regiochemistry of two benzyne reactions by the substituents next to each triple bond.

We have attempted sequential benzyne reactions starting from 1,3-benzdiyne equivalents **1**, with various arynophiles (Scheme 1). This method was designed to afford unsymmetrically substituted polycyclic aromatic compounds **3**, possessing consecutive fused-rings, as are often seen in material and pharmaceutical science.^10^ While compounds like **3** have been mainly synthesized *via* linear, multi-step routes, our approach is convergent and rapid, proceeding by the combination of **1** and two different arynophiles (**I** and **II**), and allows a rational design for the production of a library of compounds. We were particularly interested in its application to the synthesis of benzo-fused heterocycles for medicinal chemistry. Therefore, we planned reactions using heteroatomic 1,3-dipoles, such as azides, nitrones, diazo compounds, and nitrile oxides, as the arynophiles.

We aimed to develop a synthetic methodology in which (1) two benzynes (**4** and **5**) are chemoselectively generated in a stepwise manner without any additional functionalization steps, (2) each benzyne is generated under mild conditions using a fluoride, and (3) the two cycloaddition reactions of **4** and **5** with **I** and **II** proceed in a highly regioselective manner (Scheme 1). In this article, we report the preparation of a new 1,3-benzdiyne equivalent **1b** [SiR_3_ = Si(*t*-Bu)Me_2_], and a method for the preparation of unsymmetrical, angular, and multi-ring fused heterocyclic compounds **3**, which satisfies the above criteria. One significant advantage of this method is the high regioselectivity of both benzyne reactions, in which the first step is controlled by the traceless directing group, R_3_Si (ref. 11) of **4**, and the second step by the cyclic systems^12^ of **5**.

## Results and discussion

**Scheme 2:**
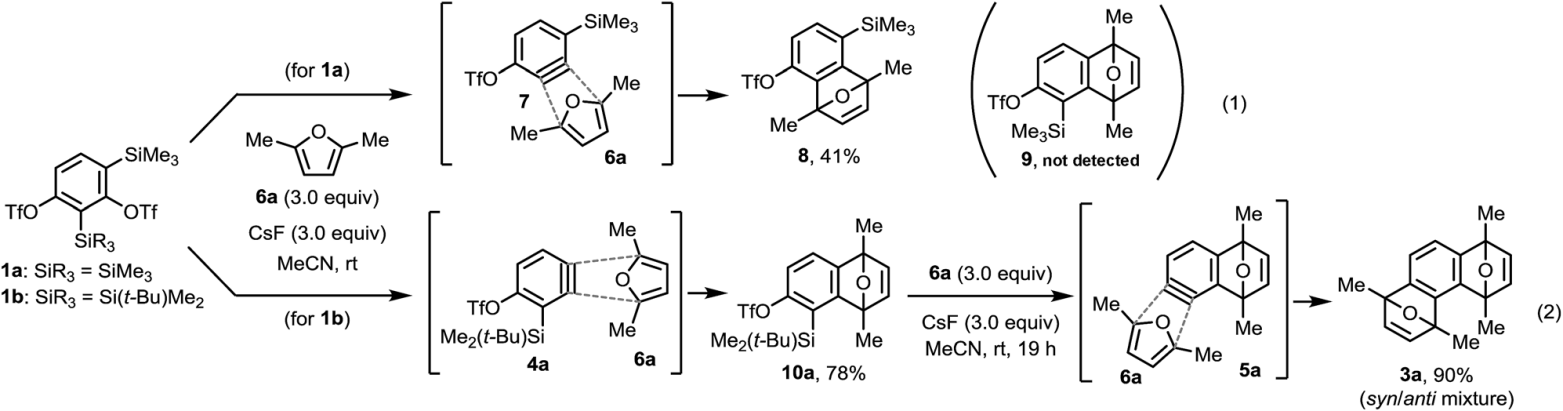
Sequential benzyne generation from benzdiyne equivalents **1a** and **1b** followed by Diels–Alder reaction with furan **6a**.

We synthesized two 1,3-benzdiyne equivalents, **1a** and **1b**, which were treated with CsF in the presence of 2,4-dimethylfuran **6a**. The reaction of **1a** with **6a** produced the undesired cycloaddition product **8***via* benzyne **7** (Scheme 2). However, the reaction of **1b** afforded the desired cycloaddition product **10a** (78% yield) through the Diels–Alder reaction of the expected benzyne **4a** with **6a**. An important observation is that the double cycloaddition product **3a** was not detected by GC analysis of the crude reaction mixture after 30 min (see ESI†). This may be due to the lower reactivity of the Me_2_(*t*-Bu)Si group, even in the presence of excess CsF and **6a**. The generation of the second benzyne **5a** was achieved after long reaction time (19 h) under the same reaction conditions using CsF to give **3a** in 90% isolated yield.

We attempted to synthesize compounds **3b–f** through stepwise benzyne cycloaddition reactions from **1b** (Table 1). All reactions of 3-silylbenzyne **4a** with arynophiles **6b–e** provided cycloaddition products **10** with good regioselectivities due to synergetic effect of the neighboring Me_2_(*t*-Bu)Si (ref. 11*f*) and the distant triflyloxy (TfO) groups.^13^ Among them, unexpected proximal regioselectivity (*proximal*-**10d** : *distal*-**10d** = 78 : 22) was observed in the reaction between 3-silylbenzyne **4a** and nitrone **6d** (entry 4-1), which was opposite to the previously reported reactions of 3-silylbenzynes with nitrones (for structural determinations, see ESI†).11f–h This result is probably due to the inductively electron-withdrawing effect of the TfO group at C4.13*a* The reaction of **4a** with sydnone **6e** to give *distal*-2*H*-indazole **10e** selectively (entry 5-1) is particularly noteworthy, as the reactions of unsymmetrical benzynes such as 3-methoxybenzyne with sydnones have been reported to afford mixtures of regioisomers in 1 : 1 ratio.2*f* The reaction of benzynes **5** with arynophiles **6b** and **6f–g** provided polycyclic compounds **3b–f**. This is the first report of the generation and reaction of 4,5-benzotriazolyne **5b** (entry 2-2), 6,7-benzisoxazolyne **5c** (entry 3-2), 4,5-benzisoxazolinyne **5d** (entry 4-2), and 6,7-2*H*-indazolyne **5e** (entry 5-2). The regioselectivity of these reactions is higher than that of sterically similar 4,5-indolyne (see preliminary theoretical discussion of these regioselectivities in ESI†).12a,b The reactions of **5c** with **6b** and **5e** with **6g** provided *distal*-**3d** and *proximal*-**3f** exclusively (entries 3-2 and 5-2).

**Table 1:** Reactions of **1b** with two different arynophiles **6** for the synthesis of angular polycycles **3**^*a*^

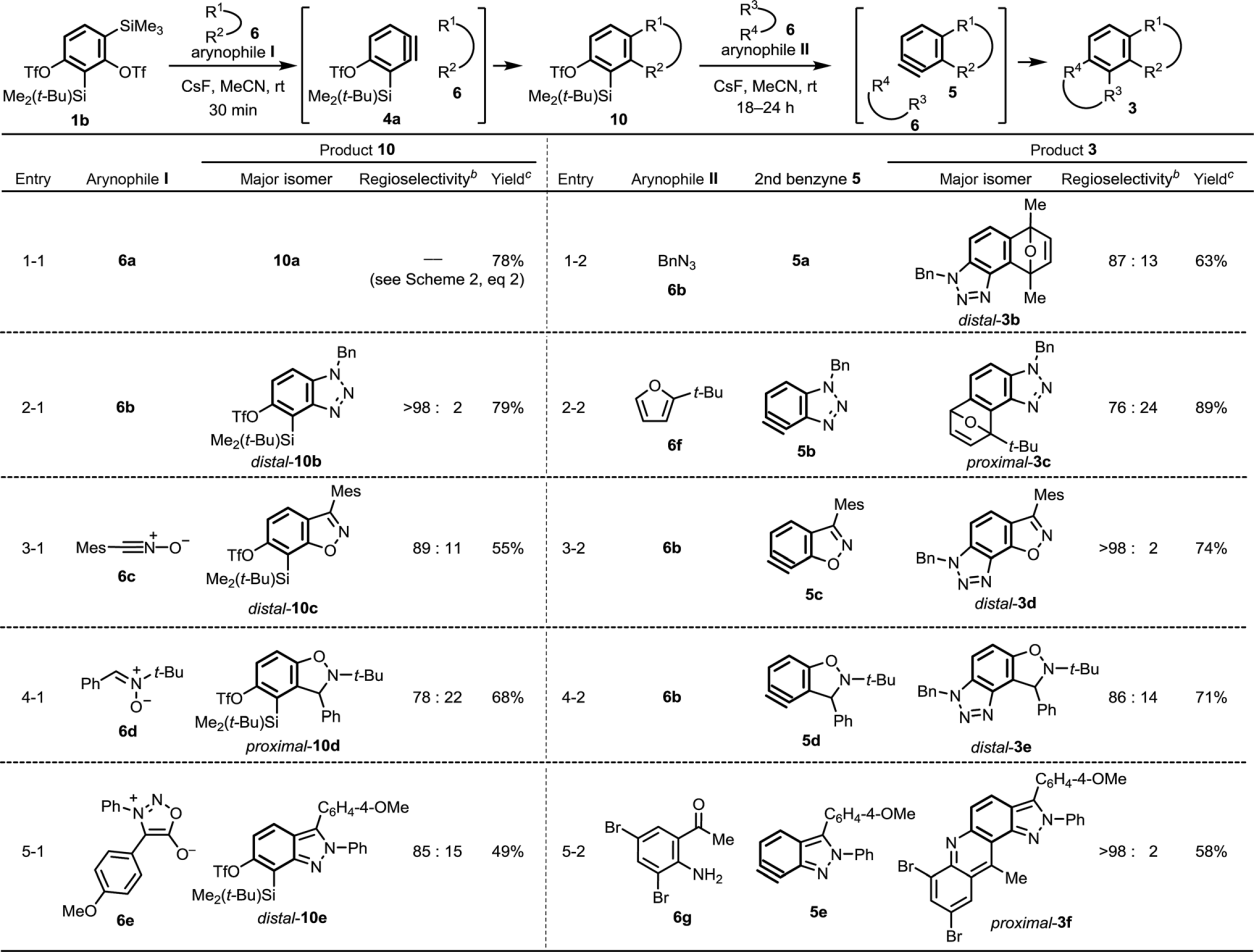

^*a*^Conditions: **1b** or **10** (1.0 equiv.), arynophile (3.0 equiv.), CsF (3.0 equiv.) in MeCN at rt.

^*b*^The ratio of major and minor products was determined by ^1^H NMR.

^*c*^Total isolated yield of *distal*-**10** (or *distal*-**3**) and its regioisomer *proximal*-**10** (or *proximal*-**3**). Mes = C_6_H_2_-2,4,6-Me_3_.

**Scheme 3:**
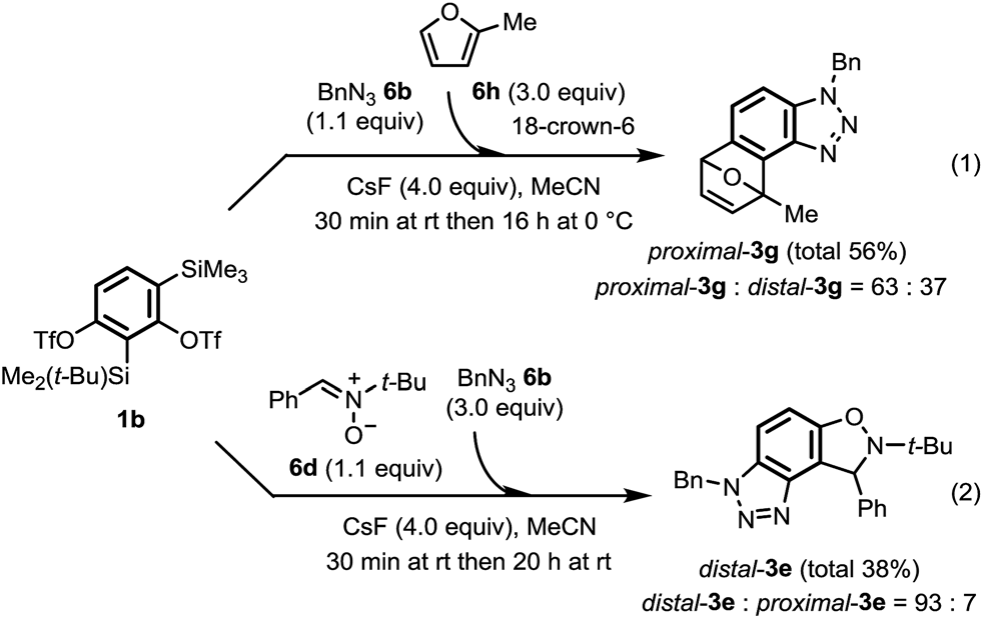
One-pot synthesis of unsymmetrical angular heterocycles **3g** and **3e**.

Next, one-pot sequential benzyne cycloadditions from **1b** were demonstrated for the synthesis of angular tricyclic heterocycles **3** without isolating **10** (Scheme 3). After a mixture of **1b** (1.0 equiv.), benzyl azide **6b** (1.1 equiv.) and CsF (4.0 equiv.) in MeCN was stirred at room temperature for 30 min, 2-methylfuran **6h** (3.0 equiv.) and 18-crown-6 (4.0 equiv.) were added and then the reaction mixture was stirred for 16 h at 0 °C (Scheme 3-1). Gratifyingly, *proximal*-**3g** was obtained as a main product (*proximal*-**3g** : *distal*-**3g** = 63 : 37, total 56%). The tricyclic compound, *distal*-**3e** was also synthesized as the predominant product (*distal*-**3e** : *proximal*-**3e** = 93 : 7, total 38%) by a similar one-pot combination of arynophiles, nitrone **6d** and **6b** (Scheme 3-2). The yield and regioselectivity of these products were comparable to those obtained by the stepwise method (Table 1, entries 4-1 and 4-2).

**Scheme 4:**
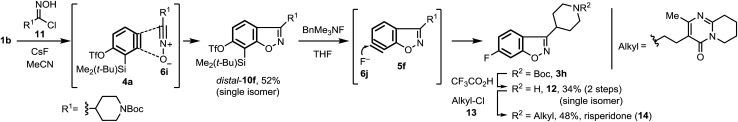
Application of 1,3-benzdiyne equivalent **1b** to the synthesis of risperidone **14**.

We applied these findings to the convergent synthesis of the antipsychotic drug risperidone **14** (Scheme 4). The silylbenzyne **4a** and a nitrile oxide **6i** (ref. 2*e*) were simultaneously generated from a mixture of **1b** and a chloro-oxime **11** and then reacted *in situ* to form *distal*-**10f** as a single regioisomer. The next reaction of 6,7-benzisoxazolyne **5f**, generated by BnMe_3_NF (ref. 14 and 15) and a fluoride **6j**, provided **3h** with excellent regioselectivity. Finally, the synthesis was completed by the *N*-deprotection of **3h** to give **12** and the alkylation with **13**.^16^ This result suggests that **1b** should be useful tool for the expeditious divergent synthesis of a wide variety of biologically active compounds and their derivatives by choosing different arynophiles **6** once **1b** become easily available (for the first synthesis of **1b**, see ESI†).

We also report the synthesis of linearly fused, unsymmetrical polycyclic aromatics **19** using **15** (ref. 17–19) as a 1,4-benzdiyne equivalent (Table 2). The first benzyne generation proceeds using CsF at room temperature in MeCN for a short time, under which conditions, generation of the second benzyne does not occur (see ESI†). The mono-cycloaddition products **17**, obtained as a mixture of two regioisomers, were subjected to the second reaction with arynophiles **II**, without separation of the regioisomers,^20^ to afford the multicyclic compounds **19**. Due to the dual effect of the TfO group13*a* and Me_3_Si group11*e* of **16**, the all first benzyne reactions proceeded in a regioselective manner beyond expectation (Table 2, entries 1-1, 2-1 and 3-1), although these selectivities were only a little lower than those of the 1,3-benzdiyne equivalent **1b** (see, Table 1). Interestingly, the second benzyne reactions also regioselectively provided cycloaddition products **19** probably because of the inductive effect of heteroatoms such as nitrogen and oxygen constructing heterocycles (entries 2-2 and 3-2). These results provide useful information for regioselectivity control of benzyne cycloadditions from distant positions. Importantly, the one-pot synthesis of a linear tricyclic compound **19a** from **15**, **6b** and **6l** was also successfully achieved (see entry 1-2).

**Table 2:** Reactions of a 1,4-benzdiyne equivalent **15** with two different arynophiles **6** for the synthesis of linear polycycles **19**^*a*^

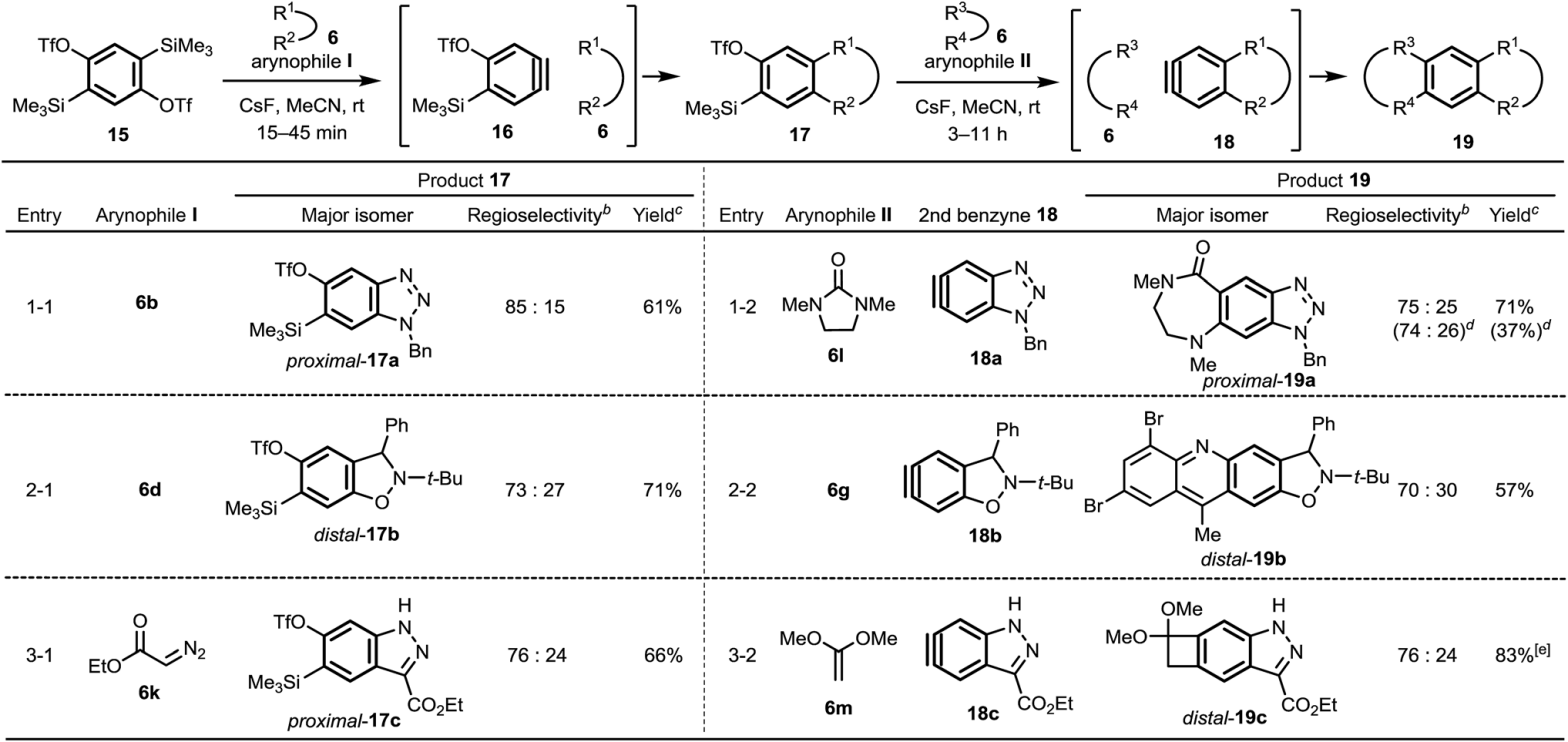

^*a*^Conditions: **15** or **17** (1.0 equiv.), arynophile **6** (3.0 equiv.), CsF (3.0 equiv.) in MeCN at rt.

^*b*^A ratio of major and minor products was determined by ^1^H NMR.

^*c*^Total isolated yield of *distal*-**17** (or *distal*-**19**) and its regioisomer *proximal*-**17** (or *proximal*-**19**).

^*d*^One-pot reaction conditions: **15** (1.0 equiv.), **6b** (1.1 equiv.), **6l** (3.0 equiv.), CsF (4.0 equiv.) in MeCN (0.1 M) at rt for 30 min and then at rt for 14 h. The yield was calculated based on **15**.

^*e*^Isolated as a corresponding ketone **19c′** after the hydrolysis of acetal **19c**.

## Conclusions

In conclusion, we have developed a novel synthetic route to multi-ring fused heterocycles by the combination of benzdiyne equivalents and arynophiles. In this study, the newly generated azole-fused benzynes were found to exhibit higher regioselectivities than those of sterically similar 4,5-indolyne.12*b* This method has facilitated the convergent synthesis of the antipsychotic risperidone. Therefore, we believe that this synthetic methodology will be invaluable to drug discovery. Work is ongoing into easier, scalable synthetic methods for these benzdiyne equivalents,^19^ analysis of the origin of the regioselectivity (see ESI†), and applications to medicinal chemistry.

## Supplementary Material

Supplementary information
