# Supplementary material for: 1,3- and 1,4-Benzdiyne equivalents for regioselective synthesis of polycyclic heterocycles
Source: Chem Sci. 2016 Apr 18;7(8):5206–11. doi: 10.1039/c6sc00798h (PMC6020534; doi:10.1039/c6sc00798h)
Supplement: Supplementary file 1 [file SC-007-C6SC00798H-s001.pdf]

Electronic Supplementary Material (ESI) for Chemical Science.
This journal is © The Royal Society of Chemistry 2016

Electronic Supplementary Information

1,3- and 1,4-Benzdiyne Equivalents for Regioselective Synthesis of Polycyclic Heterocycles

Takashi Ikawa,* Shigeaki Masuda, Akira Takagi, and Shuji Akai*

Graduate School of Pharmaceutical Sciences, Osaka University, Yamadaoka, Suita, Osaka, Japan

E-mail: ikawa@phs.osaka-u.ac.jp, akai@phs.osaka-u.ac.jp

Table of Contents

Time-course of the reactions of benzdiyne equivalents **1b** and **15**……………………………………S2
Other conditions for the reaction of 1,3-benzdiyne equivalents **1b**……………………………………S4
Theoretical analysis of 4,5-benzotriazolyne **5b** and 4,5-indolyne **5b'**……………………………S5
General considerations………………………………………………………………………………S6
General procedure for Tables and Schemes ……………………………………………………………S7
Experimental details, NMR and numerical data
Sequential benzyne generation from **1a** and **1b** (Scheme 2, Fig. S1 and S2)……………S9
Reactions of 1,3-benzdiyne equivalent **1b** (Table 1 and Scheme 3)………………………S12
Synthesis of risperidone **14** from 1,3-benzdiyne equivalent **1b** (Scheme 4)………………S22
Reactions of 1,4-benzdiyne equivalent **15** (Table 2)…………………………………………S25
Synthesis of 1,3-benzdiyne equivalent candidate **1a**…………………………………………S32
Synthesis of 1,3-benzdiyne equivalent **1b**……………………………………………………S34
Synthesis of 1,4-benzdiyne equivalent **15**……………………………………………………S39
Cartesian coordinates of optimized structures of 4,5-benzotriazolyne **5b** and 4,5-indolyne **5b'** by DFT calculation…………………………………………………………………………S41
Experimental references………………………………………………………………………………S44
1D and 2D NMR spectra of new compounds…………………………………………………………S46

Time-course of the reactions of benzdiyne equivalents 1b and 15:

a) Reaction of 1,3-benzdiyne equivalent **1b** with 2,5-dimethylfuran **6a**

For the evaluation of stepwise benzyne generation from 1,3-benzdiyne equivalent **1b**, the time-course of the reaction of **1b** was monitored by the formation of the Diels–Alder adducts (**10a** and **3a**) of each benzyne (**4a** and **5a**) with 2,5-dimethylfuran **6a** using GC (Fig. S1). We found that **1b** was rapidly consumed to produce **10a**, and its yield reached 72% during the first 30 min without formation of the double cycloaddition product **3a**. Even when **1b** was completely consumed after 2 h, the formation of **3a** was observed in only 5% yield. As we anticipated, the bulky Si(*t*-Bu)Me_2 group directed the first attack of the fluoride ion to the terminal $SiMe_3$ group to generate **4a** while efficiently retarding the second attack of another fluoride ion to **10a** (see Table 1 in the main text).

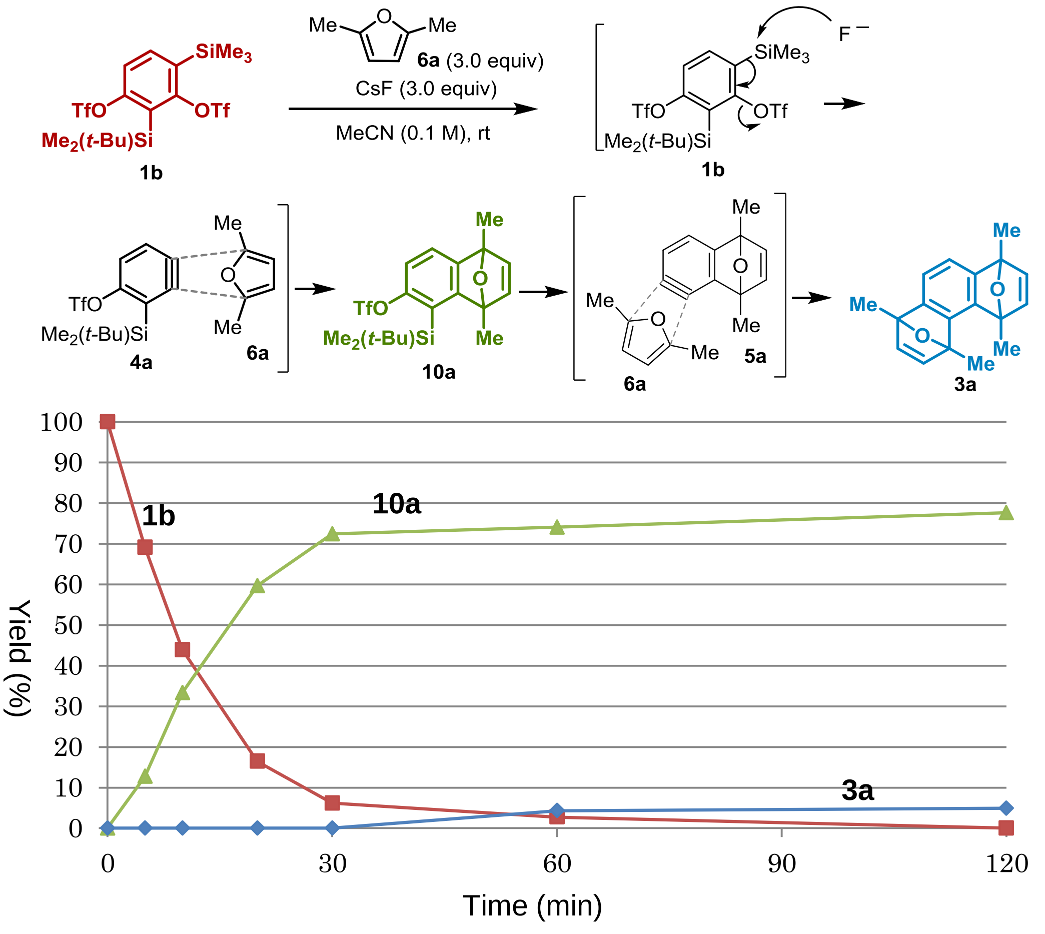

Fig. S1 Time-course of the reaction of 1,3-benzdiyne equivalent **1b** with 2,5-dimethylfuran **6a.** Conditions: **1b** (56 mg, 0.10 mmol), 2,5-dimethylfuran **6a** (32 μL, 0.30 mmol), CsF (46 mg, 0.30 mmol) and decane (21 μL, 0.10 mmol) in MeCN (1.0 mL, 0.1 M). The yield was determined by GC analysis with the aid of the internal standard, decane.

b) <u>Reaction of 1,4-benzdiyne equivalent **15** with 2,5-dimethylfuran **6a**</u>

For the evaluation of stepwise benzyne generation from **15**, the time-course of the reaction of **15** was monitored by the formation of the Diels–Alder adducts (**S1** and **S2**) of each benzyne (**16** and **18d**) with **6a** using GC (Fig. S2). The double cycloaddition product **S2** was not formed during the first 30 min, and was formed in only 2% yield after 1 h, when **15** was exhausted, and only 6% after 2 h. These results suggest that the two electron-withdrawing triflyloxy groups of **15** dramatically increase the Lewis acidity of the $SiMe_3$ group, which significantly enhances the first benzyne generation. Conversely, the second benzyne generation is relatively slow because **S1** has only one trilyloxy group. This time-course experiment clearly indicates that we can easily install two different arynophiles on **15**[1] (see Table 2 in the main text).

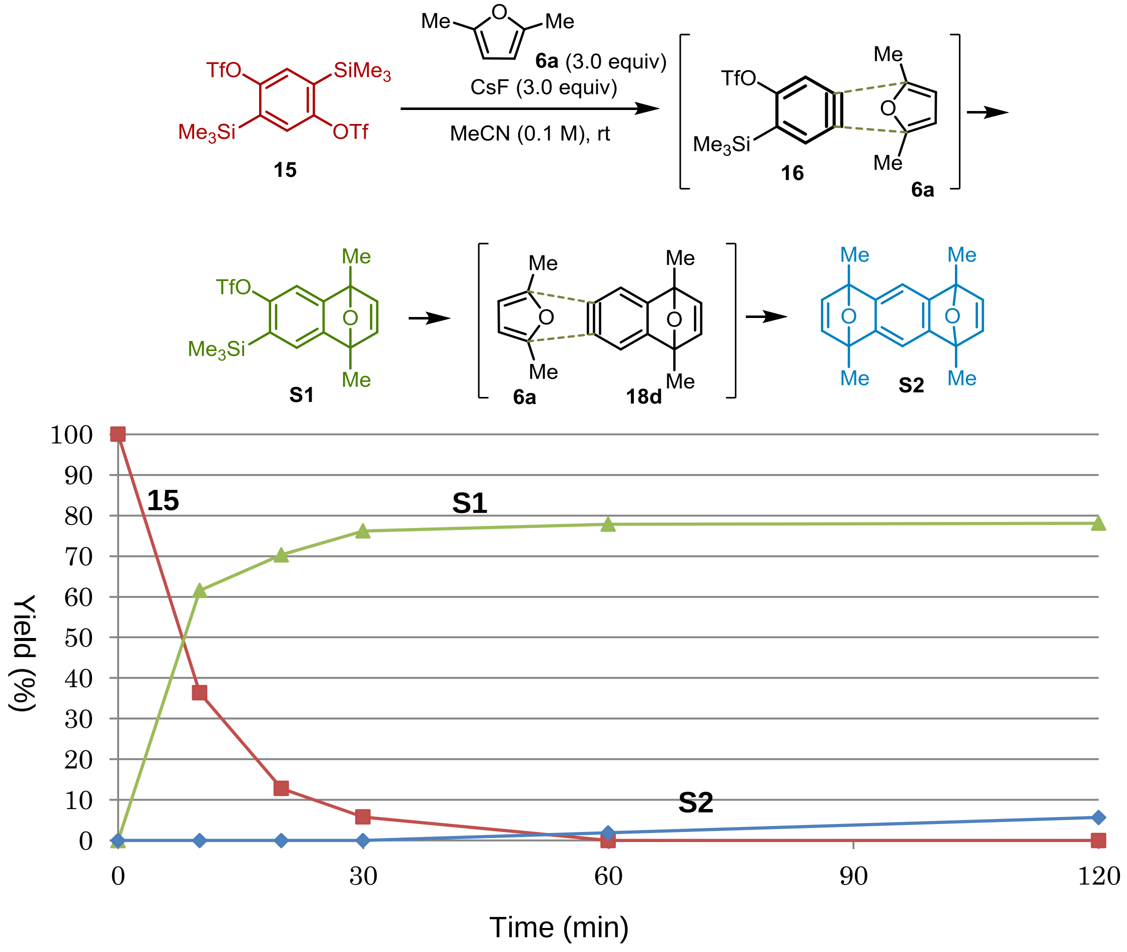

Fig. S2 Time-course of the reaction of 1,4-benzdiyne equivalent **15** with 2,5-dimethylfuran **6a**. Conditions: **15** (54 mg, 0.10 mmol), 2,5-dimethylfuran **6a** (32 μL, 0.30 mmol), CsF (46 mg, 0.30 mmol) and decane (21 μL, 0.10 mmol) in MeCN (1.0 mL, 0.1 M). The yield was determined by GC analysis with the aid of the internal standard, decane.

Other conditions for the reaction of 1,3-benzdiyne equivalents 1b:

a) KF (3.0 equiv), 18-crown-6 (3.0 equiv)

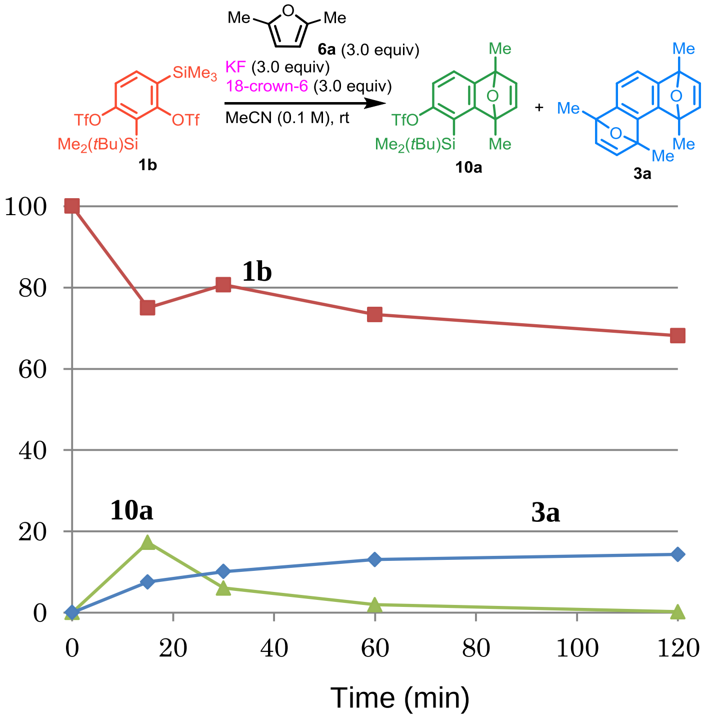

b) $BnMe_3NF$ (3.0 equiv)

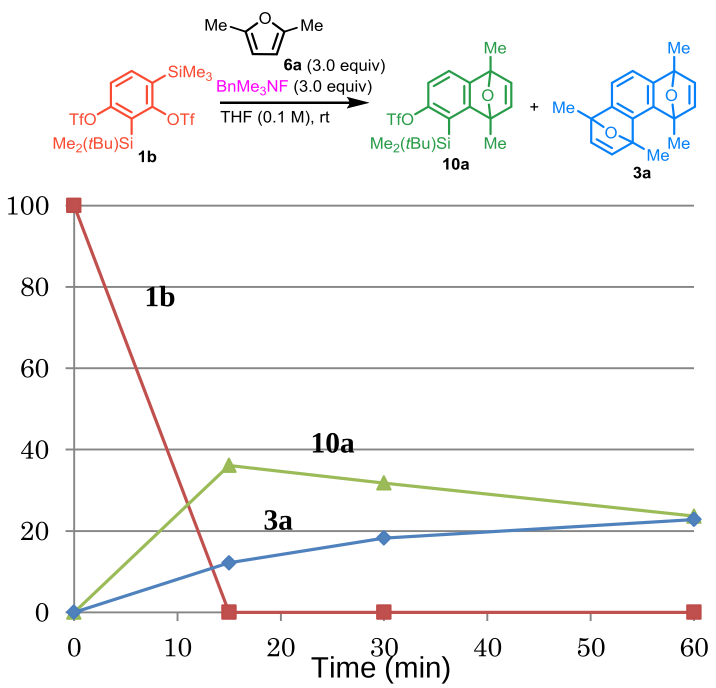

c) CsF (1.5 equiv), Furan (3.0 equiv)

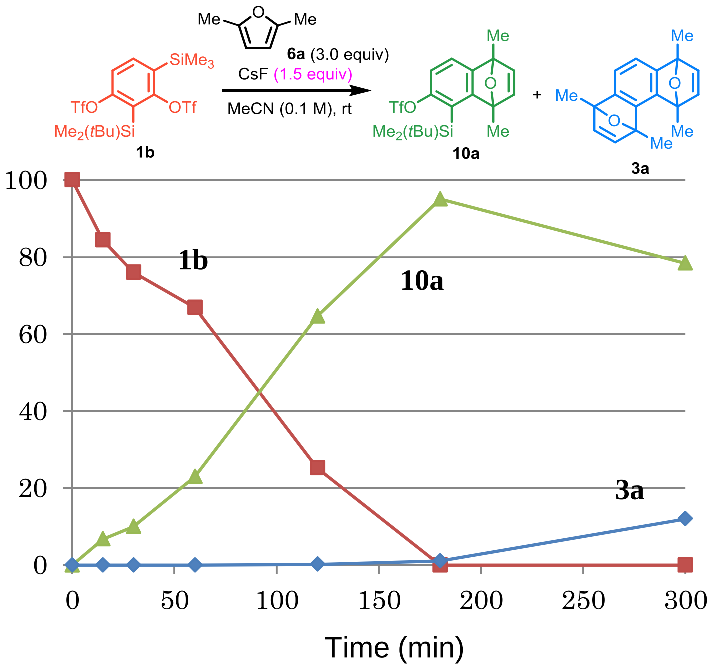

d) CsF (1.5 equiv), Furan (1.5 equiv)

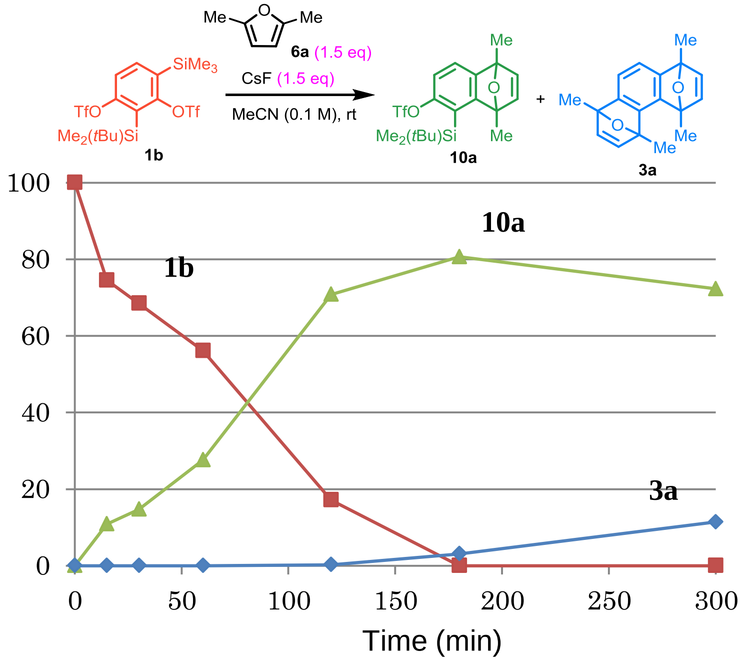

Fig. S3 Time-course of the reaction of the reaction of 1,3-benzdiyne equivalent **1b** with 2,5-dimethylfuran **6a** under other reaction conditions. Conditions: **1b** (56 mg, 0.10 mmol), 2,5-dimethylfuran **6a**, a fluoride ion and decane (21 μL, 0.10 mmol) in MeCN (1.0 mL, 0.1 M). The yield was determined by GC analysis with the aid of the internal standard, decane.

Theoretical analysis of 4,5-benzotriazolyne 5b and 4,5-indolyne 5b':

New fused benzynes, such as 4,5-benzotriazolyne **5b**, 6,7-benzisoxazolyne **5c**, and 6,7-2*H*-indazolyne **5e**, react with arynophiles **6** with good regioselectivities (Table 1, entries 2-2, 3-2, and 5-2, and Scheme 3), higher than those observed for the sterically similar 4,5-indolyne.[2a,b] To investigate the origins of these differences, distortion[2] and natural bond orbital (NBO) analyses[3–6] of 1-benzyl-4,5-benzotriazolyne **5b** and 1-benzyl-4,5-indolyne **5b'**[2a,b] have been performed, and the results are summarized in Fig. S3. These results suggest that the higher regioselectivities of **5b** than those of **5b'** can be due to the bigger differences between the internal angles at the C4 and C5 positions of **5b** and the bigger differences between the electron densities of reacting orbitals at the C4 and C5 of **5b**.

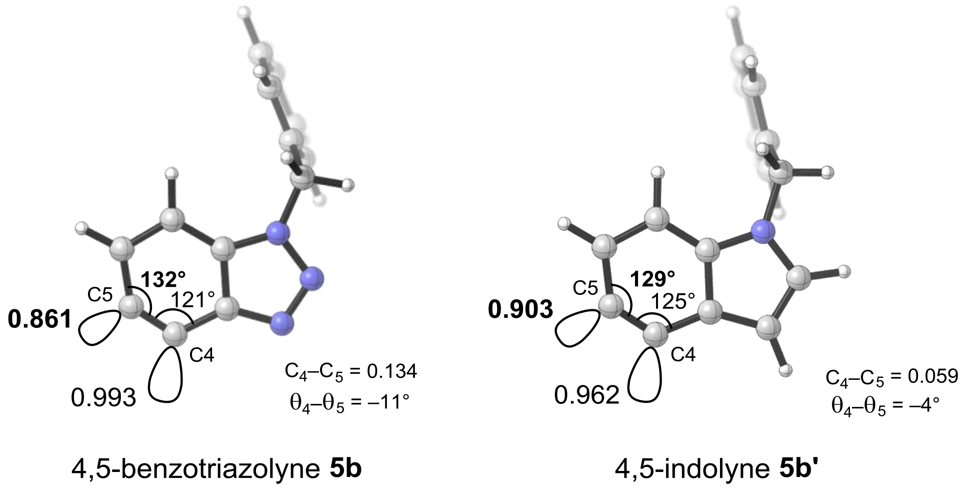

Fig. S3 Optimized geometry of 4,5-benzotriazolyne **5b** and 4,5-indolyne **5b'**, and their distortion[2] and NBO[3–6] analyses [B3LYP/6-31G(d)][7,8]

General considerations:

Reagents: All reactions were carried out under an argon or nitrogen atmosphere. A round-bottomed flask containing a magnetic stirrer with a three-way stopcock was used as a reactor. 1.6 and 2.3 M solutions of *n*-BuLi in hexane were purchased from Kanto Chemical. Anhydrous THF, CH_2Cl_2, MeCN and DMF were purchased from Kanto Chemicals, and purified with a Glass Contour solvent dispensing system (Nikko Hansen & Co., Ltd., Osaka, Japan) using two packed columns of activated molecular sieves (with an isocyanate column only for DMF). 2,4,6-Trimethylbenzonitrile oxide **6c**,[9] 4-(4-methoxyphenyl)-3-phenylsydnone **6e**,[10] and 2-acetyl-4,6-dibromoaniline **6g**,[11] were prepared according to the literature. $BnMe_3NF$ was dried at 120 °C using an oil bath under reduced pressure overnight. *N*-Bromosuccinimide (NBS) was recrystralized from boiling water before use. All other reagents were purchased from Wako Pure Chemical Industries, Tokyo Chemical Industry, Aldrich Chemical, and Kishida Chemical, and used without further purification. Flash chromatography[12] was performed with silica gel 60N, spherical neutral (40–50 μm), purchased from Kanto Chemical or Yamazen ODS column (100×2.6 cm, i.d.) packed with ODS (50 μm particle size). All reactions were monitored by thin-layer chromatography (TLC) on glass-backed silica gel 60 F254, 0.2 mm plates (Merck), and compounds were visualized under UV light (254 nm). These TLC plates were also used for preparative TLC (PTLC).

Analytical methods: Melting points were recorded on a Yanagimoto melting point apparatus and are uncorrected. IR spectra were obtained on a SHIMADZU FTIR-8400S or a SHIMADZU FTIR-IRAffinity-1 or a JASCO FT/IR 4600. ^{1}H NMR and ^{13}C NMR spectra were recorded on an Agilent Inova 600 (^{13}C: 150 MHz), JEOL JMN-ECA-500 (^{1}H: 500 MHz, ^{13}C: 125 MHz), a JEOL JMN-ECS-400 (^{1}H: 400 MHz, ^{13}C: 100 MHz), or a JEOL AL-300 (^{1}H: 300 MHz, ^{13}C: 75 MHz) instrument with chemical shifts reported in ppm relative to the residual undeuterated solvent. NOESY, ROESY, HMBC, HMQC and TOCSY spectra were taken by a JEOL JMN-ECA-500, a JEOL JMN-ECS-400, an Agelent VNS600 or a Bruker ADVANCE700. GC chromatograms were recorded on a SHIMADZU GC-2010. The mass spectra were recorded on a JEOL JMS-S3000 (MALDI), or a JEOL JMS-700 (FAB) or a JMS-T100TD (APCI) spectrometer. GPC experiment was carried out on LaboACE LC-5060 with JAIGEL-2H columns (Japan Analytical Industry). 'Yield' refers to the isolated yields of compounds showing at most only trace peaks in the ^{1}H NMR

spectra that are not attributable to the assigned structure. ^{1}H NMR and melting points (where applicable) of all known compounds were taken. All new products were further characterized by high resolution mass spectrum (HRMS). Each regiochemistry of cycloaddition products (**8**, *distal*-**3b**, *proximal*-**3b**, *distal*-**3c**, *distal*-**3d**, *distal*-**3e**, *proximal*-**3e**, *proximal*-**3g**, *distal*-**3g**, *distal*-**10b**, *distal*-**10c**, *proximal*-**10c**, *proximal*-**10d**, *distal*-**10d**, *distal*-**10e**, *proximal*-**10e**, *distal*-**10f**, *proximal*-**17a**, *distal*-**17b**, *proximal*-**17b**, *proximal*-**19a**, *distal*-**19a**, *distal*-**19b**, *proximal*-**19b**, *distal*-**19c'**, *proximal*-**19c'**) was confirmed by NOESY, those of *distal*-**17c** and *proximal*-**17c** were confirmed by HMQC and HMBC, and that of *proximal*-**3f** was confirmed by TOCSY and ROESY NMR experiments. All 1D and 2D NMR spectra were attached at the end of this material with decisive correlations circled. The structural determinations of *proximal*-**3c** and *distal*-**17a** were faild; however, those should be right sturectures because the structures of correspoinding regioisomers, *distal*-**3c** and *proximal*-**17a** were successfully defined.

General Procedures of the reactions of 1,3-benzdiyne equivalent 1b or 1,4-benzdiyne equivalent 15 with arynophiles 6 (Tables 1, 2 and Schemes 2, 4):

General Procedure A: An oven-dried round-bottom flask was charged with (**1a**,) **1b** or **15** (1.0 equiv) [and arynophile **I** (3.0 equiv) in cases where the arynophile **I** was solid] and a stirrer bar. The flask was equipped with a three-way stopcock and evacuated and back-filled with Ar. MeCN [and arynophile **I** (3.0 equiv) in cases where the arynophile **I** was liquid] was added into the flask via a syringe. Then, CsF (3.0 equiv) was quickly added to the flask. The mixture was stirred at rt for 15–30 min. The reaction mixture was passed through a short pad of silica gel using EtOAc and the solvents were removed under reduced pressure. The residue was subjected to ^{1}H NMR analysis for calculating the ratio of the two regioisomers (*distal*- and *proximal*-**10** or **17**). The crude product was purified by flash column chromatography on silica gel or PTLC (hexane or CH_2Cl_2 or a mixture of hexane and EtOAc, hexane and CH_2Cl_2, CH_2Cl_2 and EtOAc, CH_2Cl_2 and MeOH, or toluene and acetone) to afford *distal*- and *proximal*-**10** or **17**.

General Procedure B: A stirrer bar was placed into the flask with obtained one of the regioisomers **10** or a mixture of regioisomers **17** (1.0 equiv) [and arynophile **II** (3.0 equiv) in cases where the arynophile **II** was solid]. The flask was equipped with a three-way stopcock and evacuated and back-filled with Ar. MeCN [and arynophile **II** (3.0 equiv) in cases where the arynophile **II** was liquid] was added into the flask via a syringe. Then, CsF (3.0 equiv) was quickly added to the flask. The

mixture was stirred at rt for 3–24 h. The reaction mixture was passed through a short pad of silica gel using EtOAc and the solvents were removed under reduced pressure. The residue was subjected to ^{1}H NMR analysis for calculating the ratio of the two regioisomers (*distal*- and *proximal*-**3** or **19**). The crude product was purified by flash column chromatography on silica gel and/or PTLC (hexane or CH_2Cl_2 or a mixture of hexane and EtOAc, hexane and CH_2Cl_2, CH_2Cl_2 and EtOAc, CH_2Cl_2 and MeOH, or toluene and acetone) to afford *distal*- and *proximal*-**3** or **19**.

General Procedures of one-pot operation for reactions of 1,3-benzdiyne equivalent 1b or 1,4-benzdiyne equivalent 15 with arynophiles 6 (Scheme 3 and Table 2, entry 1):

General Procedure C: An oven-dried round-bottom flask was charged with **1b** or **15** (1.0 equiv) [and arynophile **6** (1.1 equiv) in cases where the arynophile **6** was solid] and a stirrer bar. The flask was equipped with a three-way stopcock and evacuated and back-filled with Ar. MeCN [and arynophile **6** (1.1 equiv) in cases where the arynophile **6** was liquid] was added into the flask via a syringe. Then, CsF (4.0 equiv) was quickly added to the flask. After the mixture was stirred for 30 min at rt, another arynophile **6** (3.0 equiv) was added via a syrindge [18-crown-6 (4.0 equiv) was also added in some case] and stirred for several hours. The reaction mixture was passed through a short pad of silica gel using EtOAc and the solvents were removed under reduced pressure. The residue was subjected to ^{1}H NMR analysis for calculating the ratio of the two regioisomers (*distal*- and *proximal*-**3** or **19**). The crude product was purified by flash column chromatography on silica gel and/or PTLC (a mixture of hexane and EtOAc or CH_2Cl_2 and EtOAc) to afford *distal*- and *proximal*-**3** or **19**.

Sequential benzyne generation from 1a and 1b (Scheme 2, Fig. S1 and S2):

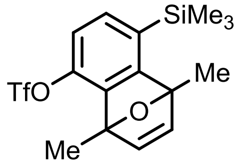

8

1,4-Dimethyl-8-(trimethylsilyl)-1,4-dihydro-5-(trifluoromethanesulfonyloxy)1,4-epoxynaphthalene (8) (Scheme 2): Following General Procedure A, a mixture of CsF (46 mg, 0.30 mmol), 2,5-dimethylfuran **6a** (32 μL, 0.30 mmol) and 2,4-bis(trimethylsilyl)-1,3-bis(trifluoromethanesulfonyloxy)benzene **1a** (52 mg, 0.10 mmol) was stirred in MeCN (1.0 mL, 0.10 M) for 10 h at rt. The crude product was purified by column chromatography on silica gel (hexane/EtOAc = 10:1) to provide the titled compound **8** as a colorless oil (16 mg, 41%) and its regiochemistry was determined by NOESY spectra. ^{1}H NMR (500 MHz, $CDCl_3$) δ: 0.36 (9 H, s), 2.026 (3 H, s), 2.029 (3 H, s), 6.80 (1 H, d, J = 8.5 Hz), 6.81 (1 H, d, J = 5.0 Hz), 6.86 (1 H, d, J = 5.0 Hz), 7.18 (1 H, d, J = 8.5 Hz). ^{13}C NMR (125 MHz, $CDCl_3$) δ: 1.03, 16.6, 18.6, 88.1 90.8, 117.2, 118.5 (q, J = 320 Hz), 131.9, 133.8, 143.3, 143.5, 146.5, 147.0, 163.9. ^{19}F NMR (470 MHz, $CDCl_3$) δ: –73.0. IR (neat): 2950, 1424 cm^{-1}. HRMS (MALDI) Calcd for $C_{16}H_{20}F_3O_4SSi$ $[M+H]^+$: 393.0798, found 393.0800.

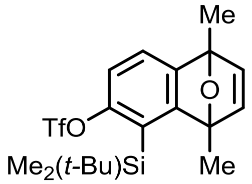

10a

1,4-Dimethyl-5-(*tert*-butyldimethylsilyl)-1,4-dihydro-6-(trifluoromethanesulfonyloxy)1,4-epoxynaphthalene (10a) (Scheme 2, Table 1, entry 1-1): Following General Procedure A, a mixture of CsF (91 mg, 0.60 mmol), 2,5-dimethylfuran **6a** (65 μL, 0.60 mmol) and 2-(*tert*-butyldimethylsilyl)-4-(trimethylsilyl)-1,3-bis(trifluoromethanesulfonyloxy)benzene **1b** (0.11 g, 0.20 mmol) was stirred in MeCN (2.0 mL) for 0.5 h at rt. The crude product was purified by column chromatography on silica gel (hexane/EtOAc = 20:1) to provide the titled compound **10a** as a colorless solid (67 mg, 78%). Mp: 79–82 °C. ^{1}H NMR (300 MHz, $CDCl_3$) δ: 0.36 (3 H, s), 0.39 (3 H,

s), 1.08 (9 H, s), 1.87 (3 H, s), 2.02 (3 H, s), 6.78 (1 H, d, J = 5.5 Hz), 6.87 (1 H, d, J = 5.5 Hz), 7.05 (1 H, d, J = 8.0 Hz), 7.12 (1 H, d, J = 8.0 Hz). ^{13}C NMR (75 MHz, $CDCl_3$) δ: 0.14, 0.85, 15.2, 18.3, 19.8, 28.3, 86.2, 91.4, 113.2, 118.6 (q, J = 322 Hz), 119.4, 124.3, 145.6, 147.3, 150.6, 154.3, 164.5. ^{19}F NMR (376 MHz, $CDCl_3$) δ: –73.1. IR (neat): 2933, 2861 cm^{-1}. HRMS (MALDI) Calcd for $C_{19}H_{26}F_3O_4SSi$ $[M+H]^+$: 435.1268, found 435.1260.

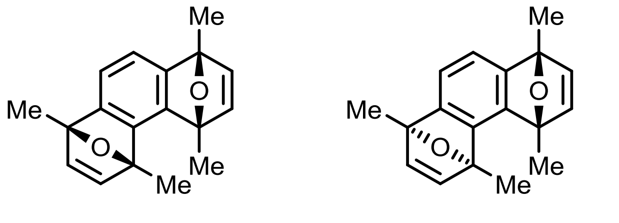

syn-**3a** *anti*-**3a**

1,4,5,8-Tetramethyl-1,4,5,8-tetrahydro-1,4:5,8-diepoxyphenanthrene (3a) (Scheme 2): Following General Procedure B, a mixture of CsF (31 mg, 0.20 mmol), 2,5-dimethylfuran **6a** (22 μL, 0.20 mmol), and **10a** (29 mg, 67 μmol) was stirred in MeCN (0.67 mL) for 19 h at rt. The crude product was purified by column chromatography on silica gel (hexane/EtOAc = 6:1) to provide the titled compound **3a** as a colorless solid (16 mg, 90%, 49:51 diastereomer mixture, determined by 500 MHz ^{1}H NMR analysis). Using 1.5 equiv of CsF, 22% of **10a** was recovered after 48 h. Mp: 88–92 °C. ^{1}H NMR (500 MHz, $CDCl_3$) δ: 1.83 (6/2 H, s), 1.84 (6/2 H, s), 1.99 (6/2 H, s), 2.05 (6/2 H, s), 6.60 (2/2 H, d, J = 5.5 Hz), 6.65–6.69 (10/2 H, m). ^{13}C NMR (125 MHz, $CDCl_3$) δ: 15.5, 15.6, 18.8, 19.8, 87.9, 88.1, 88.8, 89.3, 113.9, 114.1, 143.5, 145.2, 146.3, 146.4, 146.7, 149.4, 150.6. IR (neat): 2978, 1384 cm^{-1}. HRMS (MALDI) Calcd for $C_{18}H_{19}O_2$ $[M+H]^+$: 267.1380, found 267.1378.

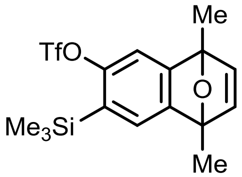

S1

1,4-Dimethyl-7-(trimethylsilyl)-1,4-dihydro-1,4-epoxynaphthalen-6-yl trifluoromethanesulfonate (S1) (Fig. S2): Following General Procedure A, a mixture of CsF (0.18 g, 1.2 mmol), 2,5-dimethylfuran **6a** (0.12 mL, 1.2 mmol) and 2,4-bis(trimethylsilyl)-1,3-bis(trifluoromethanesulfonyloxy)benzene **15** (0.20 g, 0.38 mmol) was stirred in MeCN (3.8 mL) for

10 min at rt. The crude product was purified by column chromatography on silica gel (hexane/EtOAc = 10:1) to provide the titled compound **S1** as a white solid (0.11 g, 74%). Mp: 81–83 °C. ^{1}H NMR (400 MHz, $CDCl_3$) δ: 0.34 (9 H, s), 1.87 (3 H, s), 1.90 (3 H, s), 6.77 (1 H, d, *J* = 5.5 Hz), 6.80 (1 H, d, *J* = 5.5 Hz), 7.07 (1 H, s), 7.17 (1 H, s). ^{13}C NMR (100 MHz, $CDCl_3$) δ: –0.75, 15.0, 15.1, 88.5, 88.6, 111.1, 118.4 (q, *J* = 319 Hz), 124.1, 127.9, 146.3, 146.9, 151.6, 152.4, 157.5. ^{19}F NMR (376 MHz, $CDCl_3$) δ: –73.9. IR (neat): 2979, 1420 cm^{-1}. HRMS (FAB, NBA) Calcd for $C_{16}H_{20}F_3O_4SSi$ $[M+H]^+$: 393.0798, found 393.0795.

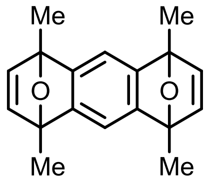

S2

1,4,5,8-Tetramethyl-1,4,5,8-tetrahydro-1,4:5,8-diepoxyanthracene (S2) (Fig. S2): Following General Procedure B, a mixture of CsF (17 mg, 0.11 mmol), 2,5-dimethylfuran **6a** (12 μL, 0.11 mmol), and **S1** (15 mg, 38 μmol) was stirred in MeCN (0.38 mL) for 20 h at rt. The crude product was purified by column chromatography on silica gel (hexane/EtOAc = 5:1) to provide the titled compound **S2** as a white solid (8.6 mg, 85%, 65:35 diastereomer mixture, determined by 400 MHz ^{1}H NMR analysis). Mp: 180 °C (decomp.). ^{1}H NMR (400 MHz, $CDCl_3$) δ: 1.86 (12/3 H, s), 1.87 (24/3 H, s), 6.77 (4/3 H, s), 6.78 (8/3 H, s), 6.96 (6/3 H, s). ^{13}C NMR (100 MHz, $CDCl_3$) δ: 15.4, 15.5, 88.78, 88.81, 110.4, 110.7, 147.3, 147.5, 151.19, 151.21. IR (neat): 2976, 1381 cm^{-1}. HRMS (MALDI) Calcd for $C_{18}H_{19}O_2$ $[M+H]^+$: 267.1380, found 267.1378.

Reactions of 1,3-benzdiyne equivalent 1b (Table 1, Scheme 3):

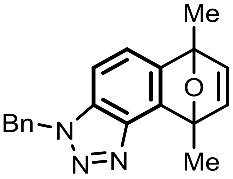

distal-**3b**

3-Benzyl-6,9-dimethyl-6,9-dihydro-3*H*-6,9-epoxynaphtho[1,2-*d*][1,2,3]triazole (*distal*-3b) (Table 1, entry 1-2): Following General Procedure B, a mixture of CsF (0.14 g, 0.90 mmol), benzyl azide **6b** (0.11 mL, 0.90 mmol), and **10a** (0.13 g, 0.30 mmol) was stirred in MeCN (3.0 mL) for 24 h at rt. The crude product (*distal*-**3b**/*proximal*-**3b** = 87:13, determined by 400 MHz ^{1}H NMR analysis) was purified by PTLC (CH_2Cl_2/EtOAc = 10:1) and ODS column (H_2O/MeOH = 63:37 to 32:68) and PTLC (CH_2Cl_2) to provide the titled compound *distal*-**3b** as a colorless oil (38 mg, 43%) and its regiochemistry was determined by NOESY spectra. ^{1}H NMR (500 MHz, $CDCl_3$) δ: 1.97 (3 H, s), 2.35 (3 H, s), 5.81 (2 H, s), 6.91 (1 H, d, J = 5.5 Hz), 6.98 (1 H, d, J = 8.0 Hz), 7.02 (1 H, d, J = 5.5 Hz), 7.23–7.33 (m, 6 H). ^{13}C NMR (125 MHz, $CDCl_3$) δ: 15.6, 16.8, 52.4, 89.6, 89.7, 105.2, 118.2, 127.5, 128.4, 128.9, 131.9, 134.7, 141.1, 145.3, 147.6, 148.7, 150.8. IR (neat): 2956, 1455 cm^{-1}. HRMS (MALDI) Calcd for $C_{19}H_{17}N_3ONa$ $[M+Na]^+$: 326.1264, found 326.1266.

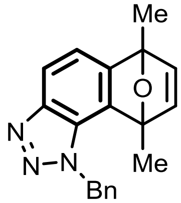

proximal-**3b**

1-Benzyl-6,9-dimethyl-6,9-dihydro-1*H*-6,9-epoxynaphtho[1,2-*d*][1,2,3]triazole (*proximal*-3b) (Table 1, entry 1-2) was obtained from the above-mentioned reaction mixture as a colorless oil (18 mg, 20%) and its regiochemistry was determined by NOESY spectra. ^{1}H NMR (500 MHz, $CDCl_3$) δ: 1.946 (3 H, s), 1.953 (3 H, s), 5.92 (1 H, d, J = 17.0 Hz), 6.25 (1 H, d, J = 5.0 Hz), 6.27 (1 H, d J = 17.0 Hz), 6.80 (1 H, d, J = 5.0 Hz), 6.81 (2 H, dd, J = 7.5 Hz, 3.5 Hz), 7.29–7.33 (m, 5 H), 7.85 (1 H, d, J = 8.0 Hz). ^{13}C NMR (125 MHz, $CDCl_3$) δ: 15.4, 18.4, 53.2, 89.3, 89.7, 115.8, 117.3, 125.5,

127.9, 128.0, 129.0, 136.0, 136.2, 146.8, 147.2, 148.5, 155.7. IR (neat): 2925, 1437 cm^{-1}. HRMS (MALDI) Calcd for $C_{19}H_{17}N_3ONa$ $[M+Na]^+$: 304.1444, found 304.1438.

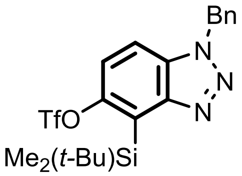

distal-**10b**

1-Benzyl-4-(*tert*-butyldimethylsilyl)-5-(trifluoromethanesulfonyloxy)benzotriazole (*distal*-10b) (Table 1, entry 2-1): Following General Procedure A, a mixture of CsF (96 mg, 0.60 mmol), benzyl azide **6b** (75 μL, 0.60 mmol), and 2-(*tert*-butyldimethylsilyl)-4-(trimethylsilyl)-1,3-bis(trifluoromethanesulfonyloxy)benzene **1b** (0.11 g, 0.20 mmol) was stirred in MeCN (2.0 mL) for 2 h at rt. The crude product (*distal*-**10b**/*proximal*-**10b** = >98:2, determined by 300 MHz ^{1}H NMR analysis) was purified by column chromatography on silica gel (hexane/EtOAc = 10:1) to provide the titled compound *distal*-**10b** as a brown solid (74 mg, 79%) and its regiochemistry was determined by NOESY spectra. Mp: 80–81°C. ^{1}H NMR (500 MHz, $CDCl_3$) δ: 0.65 (6 H, s), 0.96 (9 H, s), 5.83 (2 H, s), 7.30–7.39 (7 H, m). ^{13}C NMR (125 MHz, $CDCl_3$) δ: –2.53, 18.2, 27.0, 52.6, 111.8, 118.4 (q, J = 320 Hz), 119.8, 123.6, 127.7, 128.7, 129.2, 130.2, 134.1, 151.2, 151.6. ^{19}F NMR (376 MHz, $CDCl_3$) δ: –73.8. IR (neat): 2930, 1418 cm^{-1}. HRMS (MALDI) Calcd for $C_{20}H_{25}F_3N_3O_3SSi$ $[M+H]^+$: 472.1332, found 472.1339.

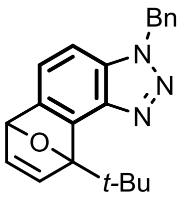

proximal-**3c**

3-Benzyl-9-(*tert*-butyl)-6,9-dihydro-3*H*-6,9-epoxynaphtho[1,2-*d*][1,2,3]triazole (*proximal*-3c) (Table 1, entry 2-2): Following General Procedure B, a mixture of CsF (32 mg, 0.21 mmol), 2-*tert*-butylfuran **6f** (30 μL, 0.21 mmol), and *distal*-**10b** (33 mg, 71 μmol) was stirred in MeCN (1.0 mL) for 18 h at rt. The crude product (*distal*-**3c**/*proximal*-**3c** = 24:76, determined by 400 MHz ^{1}H NMR analysis) was purified by column chromatography on silica gel (hexane/EtOAc = 3:1) to provide the

titled compound *proximal*-**3c** as a colorless solid (16 mg, 68%). Mp: 180–182 °C. ^{1}H NMR (500 MHz, $CDCl_3$) δ: 1.51 (9 H, s), 5.76 (1 H, d, J = 15.5 Hz), 5.82 (s, 1 H), 5.83 (1 H, d, J = 15.5 Hz), 7.02 (1 H, d, J = 8.0 Hz), 7.12–7.25 (2 H, m), 7.26–7.35 (6 H, m). ^{13}C NMR (125 MHz, $CDCl_3$) δ: 26.6, 32.4, 52.3, 82.2, 102.9, 105.6, 119.6, 127.6, 128.4, 128.9, 131.9, 134.7, 141.3, 142.6, 143.7, 146.2, 150.5. IR (neat): 2958, 1507 cm^{-1}. HRMS (MALDI) Calcd for $C_{21}H_{22}N_3O$ $[M+H]^+$: 332.1757, found 332.1757.

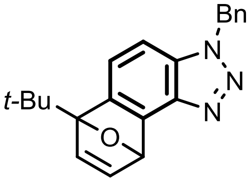

distal-**3c**

3-Benzyl-6-(*tert*-butyl)-6,9-dihydro-3*H*-6,9-epoxynaphtho[1,2-*d*][1,2,3]triazole (*distal*-3c) (Table 1, entry 2-2) was obtained from the above-mentioned reaction mixture as a colorless solid (5.0 mg, 21%) and its regiochemistry was determined by NOESY spectra. Mp: 140–142 °C. ^{1}H NMR (500 MHz, $CDCl_3$) δ: 1.30 (9 H, s), 5.80 (2 H, s), 6.36 (1 H, d, J = 1.5 Hz), 6.96 (1 H, d, J = 8.5 Hz), 7.10 (1 H, d, J = 5.5 Hz), 7.19 (1 H, dd, J = 1.5, 5.5 Hz), 7.26–7.35 (5 H, m), 7.56 (1 H, d, J = 8.5 Hz). ^{13}C NMR (125 MHz, $CDCl_3$) δ: 26.7, 32.5, 52.4, 80.0, 100.6, 104.7, 121.3, 127.6, 128.5, 129.0, 131.2, 134.6, 141.0, 144.6, 144.8, 145.5, 147.1. IR (neat): 2959, 1455 cm^{-1}. HRMS (MALDI) Calcd for $C_{21}H_{22}N_3O$ $[M+H]^+$: 332.1757, found 332.1752.

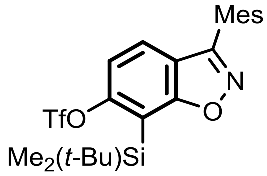

distal-**10c**

7-(*tert*-Butyldimethylsilyl)-3-mesitylbenzo[*d*]isoxazol-6-yl trifluoromethanesulfonate (*distal*-10c) (Table 1, entry 3-1): Following General Procedure A, a mixture of CsF (47 mg, 0.30 mmol), 2,4,6-trimetylphenylnitrileoxide **6c**[9] (48 mg, 0.30 mmol), and 2-(*tert*-butyldimethylsilyl)-4-(trimethylsilyl)-1,3-bis(trifluoromethanesulfonyloxy)benzene **1b** (57 mg, 0.10 mmol) was stirred in MeCN (1.0 mL) for 0.5 h at rt. The crude product (*distal*-**10c**/*proximal*-**10c** = 89:11, determined by 300 MHz ^{1}H NMR analysis) was purified by PTLC (hexane/EtOAc = 10:1) to provide the titled

compound *distal*-**10c** as a yellow oil (24 mg, 49%) and its regiochemistry was determined by NOESY spectra. ^{1}H NMR (500 MHz, $CDCl_3$) δ: 0.64 (6 H, s), 0.98 (9 H, s), 2.08 (6 H, s), 2.38 (3 H, s), 7.02 (2 H, s), 7.35 (1 H, d, *J* = 8.5 Hz), 7.43 (1 H, d, *J* = 8.5 Hz). ^{13}C NMR (125 MHz, $CDCl_3$) δ: –3.38, 18.4, 20.1, 21.2, 26.6, 113.0, 115.8, 118.5 (q, *J* = 320 Hz), 119.8, 123.4, 124.1, 128.6, 137.6, 139.6, 156.3, 157.6, 168.4. ^{19}F NMR (470 MHz, $CDCl_3$) δ: –73.5. IR (neat): 2929, 1424 cm^{-1}. HRMS (MALDI) Calcd for $C_{23}H_{29}F_3NO_4SSi$ [M+H]$^+$: 500.1533, found 500.1531.

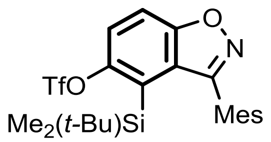

proximal-**10c**

4-(*tert*-Butyldimethylsilyl)-3-mesitylbenzo[*d*]isoxazol-5-yl trifluoromethanesulfonate (*proximal*-10c) (Table 1, entry 3-1) was obtained from the above-mentioned reaction mixture as a yellow oil (3.1 mg, 6%) and its regiochemistry was determined by NOESY spectra. ^{1}H NMR (300 MHz, $CDCl_3$) δ: 0.05 (6 H, s), 0.74 (9 H, s), 2.04 (6 H, s), 2.36 (3 H, s), 6.95 (2 H, s), 7.68 (1 H, d, *J* = 9.5 Hz), 7.76 (1 H, d, *J* = 9.5 Hz). ^{13}C NMR (150 MHz, $CDCl_3$) δ: –1.79, 18.9, 20.6, 21.2, 27.3, 112.7, 118.6 (q, *J* = 320 Hz), 120.7, 126.6, 126.8, 127.3, 128.7, 138.3, 139.7, 153.4, 159.0, 161.3. ^{19}F NMR (470 MHz, $CDCl_3$) δ: –72.8. IR (neat): 2931, 1424 cm^{-1}. HRMS (MALDI) Calcd for $C_{23}H_{29}F_3NO_4SSi$ [M+H]$^+$: 500.1533, found 500.1530.

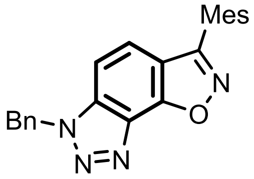

distal-**3d**

3-Benzyl-6-mesityl-3*H*-isoxazolo[5',4':3,4]benzo[1,2-*d*][1,2,3]triazole (*distal*-3d) (Table 1, entry 3-2): Following General Procedure B, a mixture of CsF (50 mg, 0.33 mmol), benzyl azide **6b** (41 µL, 0.33 mmol) and *distal*-**10c** (42 mg, 0.11 mmol) was stirred in MeCN (1.1 mL, 0.10 M) for 19 h at rt. The crude product (*distal*-**3d**/*proximal*-**3d** = >98:2, determined by 400 MHz ^{1}H NMR analysis) was purified by column chromatography on silica gel (hexane/EtOAc = 5:1) to provide the titled compound *distal*-**3d** as a brown oil (30 mg, 74%) and its regiochemistry was determined by NOESY

spectra. ^{1}H NMR (500 MHz, $CDCl_3$) δ: 2.06 (6 H, s), 2.36 (3 H, s), 5.93 (2 H, s), 7.00 (2 H, s), 7.25 (1 H, d, *J* = 9.0 Hz), 7.29 (1 H, d, *J* = 9.0 Hz), 7.31–7.37 (5 H, m). ^{13}C NMR (125 MHz, $CDCl_3$) δ: 20.1, 21.2, 52.8, 106.8, 118.1, 120.9, 123.5, 127.6, 128.5, 128.7, 129.1, 132.7, 134.1, 135.2, 137.6, 139.5, 154.8, 158.3. IR (neat): 2922, 1507 cm^{-1}. HRMS (MALDI) Calcd for $C_{23}H_{21}N_4O$ [M+H]$^+$: 369.1710, found 369.1713.

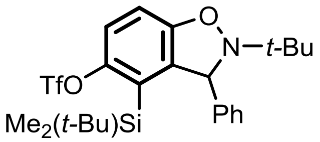

proximal-**10d**

2-(*tert*-Butyl)-4-(*tert*-butyldimethylsilyl)-5-(trifluoromethanesulfonyloxy)-3-phenyl-2,3-dihydrobenzoisoxazole (*proximal*-10d) (Table 1, entry 4-1): Following General Procedure A, a mixture of CsF (48 mg, 0.30 mmol), *N*-*tert*-butylphenyl nitrone **6d** (53 mg, 0.30 mmol), and 2-(*tert*-butyldimethylsilyl)-4-(trimethylsilyl)-1,3-bis(trifluoromethanesulfonyloxy)benzene **1b** (56 mg, 0.10 mmol) was stirred in MeCN (1.0 mL) for 0.5 h at rt. The crude product (*distal*-**10d**/*proximal*-**10d** = 22:78, determined by 500 MHz ^{1}H NMR analysis) was purified by PTLC (hexane/EtOAc = 10:1) to provide the titled compound *proximal*-**10d** as a red oil (27 mg, 53%) and its regiochemistry was determined by NOESY spectra. ^{1}H NMR (500 MHz, $CDCl_3$) δ: 0.15 (3 H, s), 0.38 (3 H, s), 0.68 (9 H, s), 1.18 (9 H, s), 5.68 (1 H, s), 6.90 (2 H, d, *J* = 7.5 Hz), 6.97 (1 H, d, *J* = 9.0 Hz), 7.21–7.7.30 (3 H, m), 7.34 (1 H, d, *J* = 9.0 Hz). ^{13}C NMR (125 MHz, $CDCl_3$) δ: –1.87, –1.80, 18.4, 25.9, 27.1, 61.4, 66.3, 108.5, 118.5 (q, *J* = 321 Hz), 119.2, 126.2, 127.79, 127.85, 128.9, 135.4, 142.6, 151.0, 156.8. ^{19}F NMR (470 MHz, $CDCl_3$) δ: –73.5. IR (neat): 2931, 1418 cm^{-1}. HRMS (MALDI) Calcd for $C_{24}H_{33}F_3NO_4SSi$ [M+H]$^+$: 516.1846, found 516.1840.

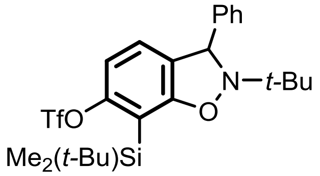

distal-**10d**

2-(*tert*-Butyl)-7-(*tert*-butyldimethylsilyl)-6-(trifluoromethanesulfonyloxy)-3-phenyl-2,3-dihydrobenzoisoxazole (*distal*-10d) (Table 1, entry 4-1) was obtained from the above-mentioned

reaction mixture as a yellow oil (7.7 mg, 15%) and its regiochemistry was determined by NOESY spectra. ^{1}H NMR (500 MHz, $CDCl_3$) δ: 0.44 (3 H, s), 0.45 (3 H, s), 0.97 (9 H, s), 1.16 (9 H, s), 5.55 (1 H, s), 6.78 (1 H, d, J = 8.5 Hz), 6.82 (1 H, d, J = 8.5 Hz), 7.27 (1 H, t, J = 7.5 Hz), 7.34 (2 H, t, J = 7.5 Hz), 7.38 (2 H, d, J = 7.5 Hz). ^{13}C NMR (125 MHz, $CDCl_3$) δ: –3.16, –3.04, 18.4, 25.7, 26.8, 60.9, 66.6, 108.2, 111.2, 118.5 (q, J = 321 Hz), 125.7, 127.5, 127.8, 128.7, 128.8, 143.4, 155.8, 162.2. ^{19}F NMR (470 MHz, $CDCl_3$) δ: –73.5. IR (neat): 2931, 1420 cm^{-1}. HRMS (MALDI) Calcd for $C_{23}H_{29}F_3NO_4SSi$ $[M+H]^+$: 516.1846, found 516.1856.

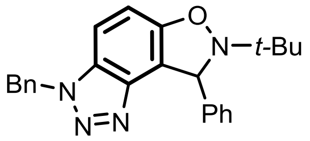

distal-**3e**

3-Benzyl-7-(*tert*-butyl)-8-phenyl-7,8-dihydro-3*H*-isoxazolo[4',5':3,4]benzo[1,2-*d*][1,2,3]triazole (*distal*-3e) (Table 1, entry 4-2 and Scheme 3-2):

Following General Procedure B, a mixture of CsF (21 mg, 0.14 mmol), benzyl azide **6b** (17 µL, 0.14 mmol) and *proximal*-**10d** (24 mg, 46 µmol) was stirred in MeCN (1.0 mL) for 3 h at rt. The crude product (*distal*-**3e**/*proximal*-**3e** = 86:14, determined by 400 MHz ^{1}H NMR analysis) was purified by PTLC (hexane/EtOAc = 5:1) to provide the titled compound *distal*-**3e** as a colorless oil (11 mg, 61%) and its regiochemistry was determined by NOESY spectra. ^{1}H NMR (500 MHz, $CDCl_3$) δ: 1.21 (9 H, s), 5.75 (1 H, s), 5.76 (1 H, s), 6.20 (1 H, s), 7.00 (1 H, J = 9.0 Hz, d), 7.18 (1 H, J = 9.0 Hz, d), 7.22–7.38 (8 H, m), 7.69 (2 H, J = 7.5 Hz, d). ^{13}C NMR (125 MHz, $CDCl_3$) δ: 25.2, 52.6, 61.7, 65.8, 109.8, 110.2, 115.2, 127.4, 127.5, 127.7, 128.6, 129.0, 129.2, 129.8, 134.4, 141.9, 142.0, 154.1. IR (neat): 2976, 1445 cm^{-1}. HRMS (MALDI) Calcd for $C_{24}H_{24}N_4ONa$ $[M+Na]^+$: 407.1842, found 407.1843.

Following General Procedure C, a mixture of CsF (60 mg, 0.40 mmol), *N*-*tert*-butylphenyl nitrone **6d** (19 mg, 0.11 mmol), benzyl azide **6b** (37 µL, 0.30 mmol), and 2-(*tert*-butyldimethylsilyl)-4-(trimethylsilyl)-1,3-bis(trifluoromethanesulfonyloxy)benzene **1b** (56 mg, 0.10 mmol) was stirred in MeCN (1.0 mL) for 20 h at rt. The crude product (*distal*-**3e**/*proximal*-**3e** = 93:7, determined by 400 MHz ^{1}H NMR analysis) was purified by PTLC (hexane/EtOAc = 5:1) to provide the titled compound *distal*-**3e** with *proximal*-**3e** as a brown oil (15 mg, 38%).

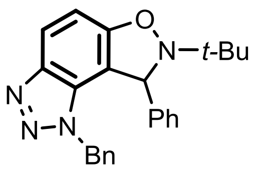

proximal-**3e**

1-Benzyl-7-(*tert*-butyl)-8-phenyl-7,8-dihydro-1*H*-isoxazolo[4',5':3,4]benzo[1,2-*d*][1,2,3]triazole (*proximal*-3e) (Table 1, entry 4-2) was obtained from the above-mentioned reaction mixture as a colorless oil (1.8 mg, 10%) and its regiochemistry was determined by NOESY spectra. ^{1}H NMR (500 MHz, $CDCl_3$) δ: 0.91 (9 H, s), 4.79 (1 H, d, *J* = 16.5 Hz), 5.09 (1 H, s), 5.90 (1 H, d, *J* = 16.5 Hz), 6.87–6.91 (2 H, m), 6.98–7.05 (3 H, m), 7.29–7.38 (6 H, m), 7.98 (1 H, d, *J* = 9.0 Hz). ^{13}C NMR (151 MHz, $CDCl_3$) δ: 25.0, 52.2, 61.6, 65.1, 106.2, 106.9, 121.3, 126.2. 127.6, 128.3, 128.4, 129.0, 129.2, 129.3, 136.7, 142.4, 143.8, 158.5. IR (neat): 2926, 1497 cm^{-1}. HRMS (MALDI) Calcd for $C_{24}H_{25}N_4O$ [M+H]$^{+}$: 385.2023, found 385.2020.

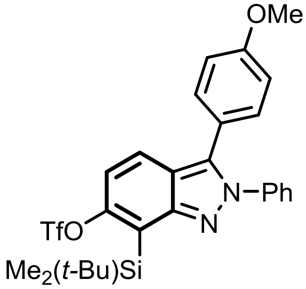

distal-**10e**

7-(*tert*-Butyldimethylsilyl)-3-(4-methoxyphenyl)-2-phenyl-2*H*-indazol-6-yl trifluoromethanesulfonate (*distal*-10e) (Table 1, entry 5-1): Following General Procedure A, a mixture of CsF (46 mg, 0.30 mmol), 3-phenyl-4-(4-methoxyphenyl)sydnone **6e**[10] (58 mg, 0.30 mmol), and 2-(*tert*-butyldimethylsilyl)-4-(trimethylsilyl)-1,3-bis(trifluoromethanesulfonyloxy)benzene **1b** (56 mg, 0.10 mmol) was stirred in MeCN (1.0 mL, 0.10 M) for 0.5 h at rt. The crude product (*distal*-**10e**/*proximal*-**10e** = 85:15, determined by 300 MHz ^{1}H NMR analysis) was purified by PTLC (hexane/EtOAc = 10:1) to provide the titled compound *distal*-**10e** as a yellow solid (26 mg, 43%) and its regiochemistry was determined by NOESY spectra. Mp: 119–121 °C. ^{1}H NMR (300 MHz, $CDCl_3$) δ: 0.61 (6 H, s), 1.02 (9 H, s), 3.86 (3 H, s), 6.94 (2 H, d, *J* = 8.5 Hz), 7.09 (1 H, d, *J* = 9.0 Hz), 7.25 (2 H, d, *J* = 8.5 Hz), 7.35–7.44 (5 H, m), 7.70 (1 H, d, *J* = 9.0 Hz). ^{13}C NMR (100 MHz, $CDCl_3$) δ: –2.30, 18.5, 27.3, 55.3, 114.4, 115.4, 118.5 (q, *J* =

320 Hz), 118.6, 119.6, 121.7, 124.1, 125.4, 128.0, 128.8, 130.9, 134.8, 140.2, 152.3, 154.6, 159.8. ^{19}F NMR (376 MHz, $CDCl_3$) δ: –73.7. IR (neat): 2925, 2854 cm^{-1}. HRMS (MALDI) Calcd for $C_{27}H_{30}F_3N_2O_4SSi$ $[M+H]^+$: 563.1642, found 563.1645.

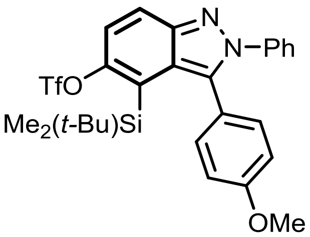

proximal-**10e**

4-(*tert*-Butyldimethylsilyl)-3-(4-methoxyphenyl)-2-phenyl-2*H*-indazol-5-yl trifluoromethanesulfonate (*proximal*-10e) (Table 1, entry 5-1) was obtained from the above-mentioned reaction mixture as a colorless oil (4.6 mg, 6%) and its regiochemistry was determined by NOESY spectra. ^{1}H NMR (400 MHz, $CDCl_3$) δ: –0.08 (6 H, s), 0.80 (9 H, s), 3.81 (3 H,s), 6.82 (2 H, d, J = 8.5 Hz), 7.12–7.17 (4 H, m), 7.27–7.30 (3 H, m), 7.42 (1 H, d, J = 9.5 Hz), 7.89 (1 H, d, J = 9.5 Hz). ^{13}C NMR (125 MHz, $CDCl_3$) δ: –0.24, 19.2, 27.8, 55.3, 113.6, 118.7 (q, J = 320 Hz), 119.0, 121.9, 122.5, 123.8, 124.3, 126.6, 128.5, 128.6, 133.8, 139.6, 140.4, 146.3, 153.8, 160.3. ^{19}F NMR (470 MHz, $CDCl_3$) δ: –73.7. IR (neat): 2930, 1415 cm^{-1}. HRMS (MALDI) Calcd for $C_{27}H_{30}F_3N_2O_4SSi$ $[M+H]^+$: 563.1642, found 563.1640.

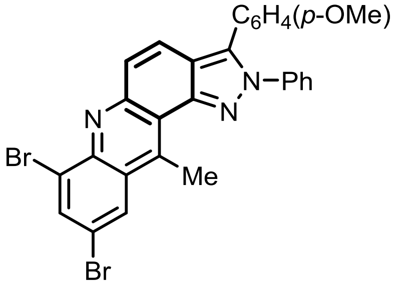

proximal-**3f**

7,9-Dibromo-3-(4-methoxyphenyl)-11-methyl-2-phenyl-2*H*-pyrazolo[3,4-*a*]acridine (*proximal*-3f) (Table 1, entry 5-2): Following General Procedure B, a mixture of CsF (31 mg, 0.21 mmol), 2-acetyl-4,6-dibromoaniline **6g**,[11] (59 mg , 0.21 mmol) and *distal*-**10e** (32 mg, 70 μmol) was stirred in MeCN (1.0 mL) for 10 h at rt. The crude product (*distal*-**3f**/*proximal*-**3f** = 2:>98, determined by 400 MHz ^{1}H NMR analysis) was purified by column chromatography on silica gel (hexane/EtOAc = 3:1)

to provide the titled compound *proximal*-**3f** as a yellow solid (18 mg, 58%) and its regiochemistry was determined by TOCSY and ROESY spectra. Mp: 255–258 °C. ^{1}H NMR (500 MHz, $CDCl_3$) δ: 3.60 (3 H, s), 3.87 (3 H, s), 6.98 (2 H, d, *J* = 8.0 Hz), 7.33 (2 H, d, *J* = 8.0 Hz), 7.37–7.47 (3 H, m), 7.51 (2 H, d, *J* = 8.0 Hz), 7.76 (1 H, d, *J* = 9.5 Hz), 7.80 (1 H, d, *J* = 9.5 Hz), 8.23 (1 H, d, *J* = 1.5 Hz), 8.52 (1 H, d, *J* = 1.5 Hz). ^{13}C NMR (125 MHz, $CDCl_3$) δ: 17.5, 55.4, 114.5, 118.6, 119.0, 120.1, 121.4, 124.7, 125.5, 126.5, 126.9, 127.0, 127.9, 128.4, 129.0, 131.1, 135.4, 136.6, 140.1, 142.7, 142.8, 146.7, 151.9, 160.0. IR (neat): 2910, 1588 cm^{-1}. HRMS (MALDI) Calcd for $C_{28}H_{20}Br_2N_3$ $[M+H]^+$: 571.9968, found 571.9960.

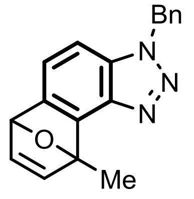

proximal-**3g**

3-Benzyl-9-methyl-6,9-dihydro-3*H*-6,9-epoxynaphtho[1,2-*d*][1,2,3]triazole (*proximal*-3g) (Scheme 3-1): Following General Procedure C, a mixture of CsF (60 mg, 0.40 mmol), benzyl azide **6b** (14 μL, 0.11 mmol), 2-methylfuran **6h** (27 μL, 0.30 mmol), 18-crown-6 (0.10 g, 0.40 mmol), and 2-(*tert*-butyldimethylsilyl)-4-(trimethylsilyl)-1,3-bis(trifluoromethanesulfonyloxy)benzene **1b** (56 mg, 0.10 mmol) was stirred in MeCN (1.0 mL) for 16 h at 0 °C. The crude product (*distal*-**3g**/*proximal*-**3g** = 37:63, determined by 400 MHz ^{1}H NMR analysis) was purified by flash column chromatography on silica gel (hexane/EtOAc = 3:1, 16 mg, 56% as a mixture of *distal*-**3g** and *proximal*-**3g**) and PTLC (hexane/EtOAc = 2:1) to provide the titled compound *proximal*-**3g** as a yellow oil (9.3 mg, 32%) and its regiochemistry was determined by NOESY spectra. ^{1}H NMR (500 MHz, $CDCl_3$) δ: 2.38 (3 H, s), 5.79 (1 H, d, *J* = 2.0 Hz), 5.81 (s, 2 H), 6.98 (1 H, d, *J* = 8.0 Hz), 7.01 (1 H, d, *J* = 5.0 Hz), 7.17 (1 H, dd, *J* = 2.0, 5.0 Hz), 7.23–7.25 (2 H, m), 7.28–7.34 (3 H, m), 7.35 (1 H, d, *J* = 8.0 Hz). ^{13}C NMR (125 MHz, $CDCl_3$) δ: 16.7, 52.4, 82.5, 90.6, 105.3, 119.6, 127.5, 128.4, 128.9, 132.1, 134.7, 141.3, 144.2, 146.28, 146.32, 149.0. IR (neat): 2932, 1456 cm^{-1}. HRMS (APCI) Calcd for $C_{18}H_{16}N_3O$ $[M+H]^+$: 290.1293, found 290.1264.

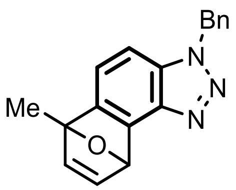

distal-**3g**

3-Benzyl-6-methyl-6,9-dihydro-3*H*-6,9-epoxynaphtho[1,2-*d*][1,2,3]triazole (*distal*-3g) (Scheme 3-1) was obtained from the above-mentioned reaction mixture as a colorless oil (5.6 mg, 19%) and its regiochemistry was determined by NOESY spectra. ^{1}H NMR (500 MHz, $CDCl_3$) δ: 2.00 (3 H, s), 5.81 (2 H, s), 6.32 (1 H, d, J = 2.0 Hz), 6.91 (1 H, d, J = 5.0 Hz), 7.00 (1 H, d, J = 8.0 Hz), 7.20 (1 H, dd, J = 2.0, 5.0 Hz), 7.22–7.25 (2 H, m), 7.28–7.32 (4 H, m). ^{13}C NMR (125 MHz, $CDCl_3$) δ: 15.4, 52.4, 80.5, 90.2, 105.4, 118.6, 127.5, 128.4, 129.0, 131.7, 134.6, 141.1, 143.7, 144.6, 147.5, 149.3. IR (neat): 2929, 1456 cm^{-1}. HRMS (APCI) Calcd for $C_{18}H_{16}N_3O$ $[M+H]^+$: 290.1293, found 290.1272.

Synthesis of risperidone 14 from 1,3-benzdiyne equivalent 1b (Scheme 4):

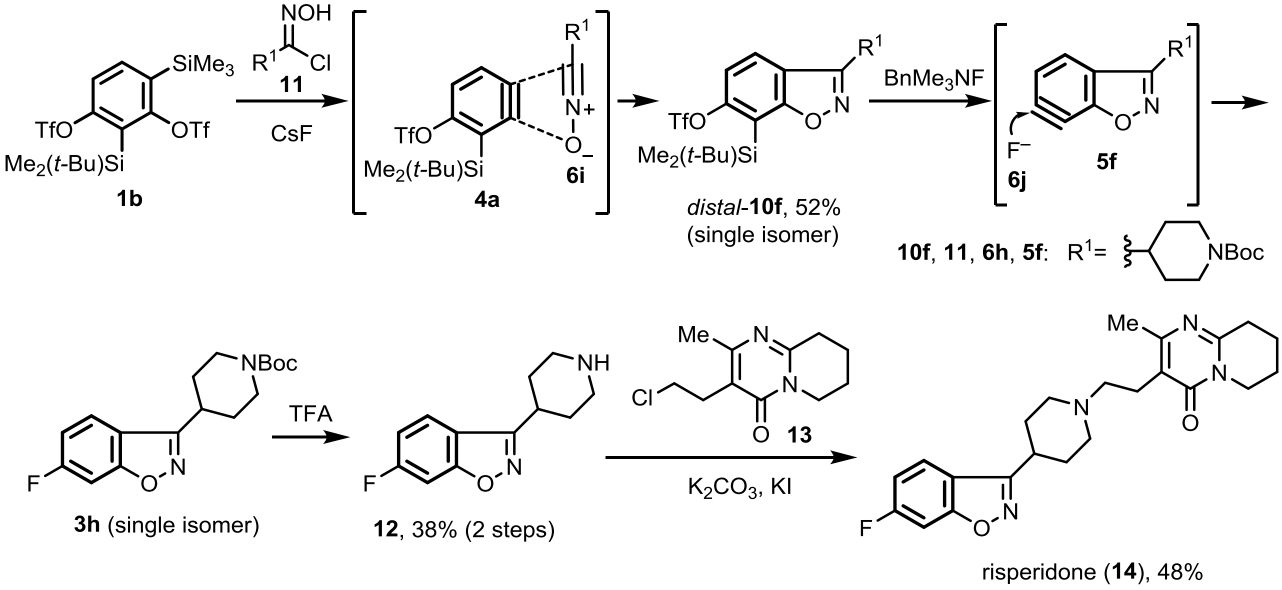

Scheme S1 Application of 1,3-benzdiyne equivalent **1b** to the synthesis of risperidone **14**.

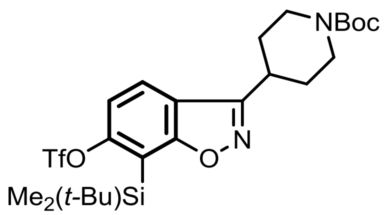

distal-**10f**

***tert*-Butyl 4-(7-(*tert*-butyldimethylsilyl)-6-(trifluoromethanesulfonyloxy)benzo[*d*]isoxazol-3-yl)piperidine-1-carboxylate (*distal*-10f) (Scheme 4):** Following General Procedure A, a mixture of CsF (0.12 g 0.54 mmol), 1-(*tert*-butoxycarbonyl)-4-(chloro(hydroxyimino)methyl)piperidine **11** (0.14 g, 54 μmol), and 2-(*tert*-butyldimethylsilyl)-4-(trimethylsilyl)-1,3-bis(trifluoromethanesulfonyloxy)benzene **1b** (50 mg, 89 μmol) was stirred in MeCN (9.0 mL) for 0.5 h at rt. The crude product (*distal*-**10f**/*proximal*-**10f** = >98:2, determined by 400 MHz ^{1}H NMR analysis) was purified by column chromatography on silica gel (hexane/EtOAc = 5:1) to provide the titled compound *distal*-**10f** (26 mg, 52%) as a brown oil and its regiochemistry was dtermined by NOESY spectra. ^{1}H NMR (300 MHz, $CDCl_3$) δ: 0.56 (6 H, s), 0.94 (9 H, s), 1.49 (9 H, s), 1.85–2.09 (4 H, m), 2.93–3.01 (2 H, m), 3.19–3.28 (m, 1 H), 4.22–4.26 (m, 2 H), 7.36 (1 H, d, J = 9.0 Hz), 7.75 (1 H, d, J = 9.0 Hz). ^{13}C NMR (125 MHz, $CDCl_3$) δ: –3.5, 18.3, 26.6, 28.4, 30.1, 34.4, 43.6 (br),

79.8, 113.2, 115.4, 118.3, 118.4 (q, J = 320 Hz), 123.5, 154.7, 156.1, 160.4, 168.5. ^{19}F NMR (470 MHz, $CDCl_3$) δ: –73.5. IR (neat): 2930, 1695 cm^{-1}. HRMS (MALDI) Calcd for $C_{24}H_{35}F_3N_2O_6NaSSi$ $[M+Na]^+$: 587.1829, found 587.1841.

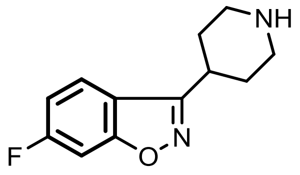

12

4-(7-Fluorobenzo[*d*]isoxazol-3-yl)piperidine (12) (Scheme 4): Following General Procedure B, a mixture of $BnMe_3NF$ (0.17 g, 1.0 mmol), and *distal*-**10f** (0.27 g, 0.48 mmol) was stirred in THF (5.0 mL) for 5 min at 0 °C. The crude product (*distal*-**3h**/*proximal*-**3h** = >98:2, determined by 400 MHz ^{1}H NMR analysis) was purified by column chromatography on silica gel (hexane/EtOAc = 5:1) to provide *distal*-**3h** (56 mg) contaminated with a small amount of impurity. A mixture of the aforementioned *distal*-**3h** (30 mg) and TFA (0.30 mL) was stirred in CH_2Cl_2 (3.0 mL) for 10 h at rt. The crude product was purified by PTLC (CH_2Cl_2/MeOH = 5:1 with 1% Et_3N) to provide pure **12** (19 mg, 34% in two-step) as a colorless oil. ^{1}H NMR (300 MHz, $CDCl_3$) δ: 1.88–2.07 (4 H, m), 2.82 (2 H, td, J = 11.5 Hz, 2.5 Hz), 3.15–3.29 (3 H, m), 7.05 (1 H, ddd, J = 9.0 Hz, 9.0 Hz, 2.0 Hz), 7.22–7.26 (1 H, m), 7.70 (1H, dd, J = 9.0 Hz, 5.0 Hz). ^{13}C NMR (125 MHz, CD_3OD) δ: 31.1, 34.8, 46.2, 98.0 (J = 26.5 Hz), 113.6 (J = 25.0 Hz), 118.4, 124.3 (J = 11.0 Hz), 162.4, 165.2, (J = 13.0 Hz), 165.8 (J = 249.5 Hz). ^{19}F NMR (376 MHz, $CDCl_3$) δ: –75.5. IR (neat): 3418, 2926, 1612, 1418 cm^{-1}. HRMS (MALDI) Calcd for $C_{12}H_{14}FN_2O$ $[M+H]^+$: 221.1085, found 221.1088.

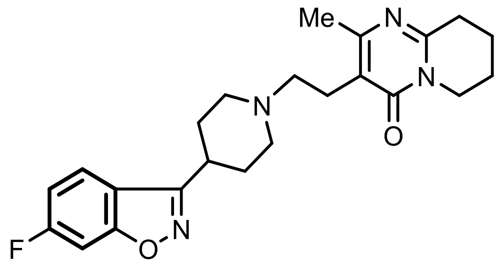

14

3-(2-(4-(6-Fluorobenzo[*d*]isoxazol-3-yl)piperidin-1-yl)ethyl)-2-methyl-6,7,8,9-tetrahydro-4*H*-pyrido[1,2-*a*]pyrimidin-4-one (14) (Scheme 4): A solution of **12** (10 mg, 45 μmol) and alkyl halide **13** (10 mg, 45 μmol) with K_2CO_3 (10 mg, 72 μmol) and KI (10 mg, 60 μmol) was stirred in DMF (1.0 mL) for 15 h at rt. The crude mixture was filtered through Celite pad and concentrated under

reduced pressure. The residue was purified by PTLC (CH_2Cl_2/MeOH = 5:1 with 1% Et_3N) to provide the titled compound **14** (8.8 mg, 48%) as a white solid. Mp: 169–172 °C. ^{1}H NMR (500 MHz, $CDCl_3$) δ: 1.85–1.91 (2 H, m), 1.93–1.99 (2 H, m), 2.04–2.15 (4 H, m), 2.55–2.34 (5 H, m), 2.51–2.56 (2 H, m), 2.74–2.79 (2 H, m), 2.87 (2 H, t, *J* = 7.0 Hz), 3.04–3.12 (1 H, m), 3.15–3.21 (2 H, m), 3.92 (2 H, d, *J* = 6.5 Hz), 7.05 (1 H, ddd, *J* = 9.0 Hz, 9.0 Hz, 2.5 Hz), 7.24, (1 H, dd, *J* = 8.0 Hz, 2.5 Hz), 7.71 (1 H, dd, *J* = 9.0 Hz, 5.0 Hz). ^{13}C NMR (125 MHz, $CDCl_3$) δ: 19.2, 21.3, 22.0. 23.7, 30.5, 31.5, 34.6, 42.7, 53.4, 56.7, 97.4 (*J* = 26.5 Hz), 112.3 (*J* = 25.0 Hz), 117.3, 119.3, 122.7 (*J* = 11.0 Hz), 155.9, 158.4, 161.1, 162.6, 163.8 (*J* = 13.0 Hz), 164.1 (*J* = 249.5 Hz).

Reaction of 1,4-benzdiyne equivalent 15 (Table 2):

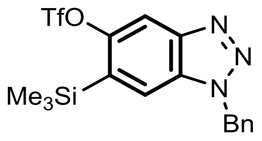

proximal-**17a**

1-Benzyl-5-(trifluoromethanesulfonyloxy)-6-(trimethylsilyl)benzotriazole (*proximal*-17a) (Table 2, entry 1-1): Following General Procedure A, a mixture of CsF (0.46 g, 3.0 mmol), benzyl azide **6b** (0.38 mL, 3.0 mmol), and 1,4-bis(trifluoromethanesulfonyloxy)-2,5-bis(trimethylsilyl)benzene **15** (0.52 g, 1.0 mmol) was stirred in MeCN (10 mL) for 40 min at rt. The crude product (*distal*-**17a**/*proximal*-**17a** = 15:85, determined by determined by 500 MHz ^{1}H NMR analysis) was purified by column chromatography on silica gel (hexane/EtOAc = 8:1) to provide the titled compound *proximal*-**17a** as a pale yellow oil (0.23 g, 52%) and its regiochemistry was determined by NOESY spectra. ^{1}H NMR (500 MHz, $CDCl_3$) δ: 0.33 (9 H, s), 5.86 (2 H, s), 7.32 (2H, d, J = 7.0 Hz), 7.34–7.40 (4 H, m), 8.04 (1 H, s). ^{13}C NMR (125 MHz, $CDCl_3$) δ: –0.96, 52.9, 110.4, 117.1, 118.4 (q, J = 319 Hz), 127.9, 128.9, 129.2, 131.6, 133.7, 133.9, 147.0, 151.1. ^{19}F NMR (376 MHz, $CDCl_3$) δ: –73.5. IR (neat): 2953, 1424 cm^{-1}. HRMS (MALDI) Calcd for $C_{17}H_{19}F_3N_3O_3SSi$ $[M+H]^+$: 430.0863, found 430.0871.

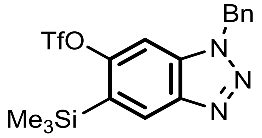

distal-**17a**

1-Benzyl-6-(trifluoromethanesulfonyloxy)-5-(trimethylsilyl)benzotriazole (*distal*-17a) (Table 2, entry 1-1) was obtained from the above-mentioned reaction mixture as a brown oil (40 mg, 9%). ^{1}H NMR (300 MHz, $CDCl_3$) δ: 0.39 (9 H, s), 5.84 (2 H, s), 7.29–7.40 (6 H, m), 8.23 (1 H, s). ^{13}C NMR (100 MHz, $CDCl_3$) δ: –0.87, 52.9, 100.7, 118.4 (q, J = 320 Hz), 127.9, 128.2, 128.9, 129.1, 129.2, 133.4, 133.6, 145.0, 153.6. ^{19}F NMR (376 MHz, $CDCl_3$) δ: –73.6. IR (neat): 2950, 1421 cm^{-1}. HRMS (MALDI) Calcd for $C_{17}H_{19}F_3N_3O_3SSi$ $[M+H]^+$: 430.0863, found 430.0867.

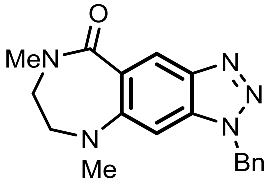

proximal-**19a**

3-Benzyl-5,8-dimethyl-5,6,7,8-tetrahydro-[1,2,3]triazolo[4',5':4,5]benzo[1,2-*e*][1,4]diazepin-9(3*H*)-one (*proximal*-18a) (Table 2, entry 1-2):

Following General Procedure B, a mixture of CsF (0.10 g, 0.69 mmol), 1,3-dimethyl-2-imidazolidinone **6l** (75 μL, 0.69 mmol), and a mixture of *distal*- and *proximal*-**17a** (0.10 g, 0.23 mmol, obtained from entry 1-1 of Table 2) was stirred in MeCN (2.3 mL) for 3 h at rt. The crude product (*distal*-**19a**/*proximal*-**19a** = 25:75, determined by 400 MHz ^{1}H NMR analysis) was purified by PTLC (CH_2Cl_2/EtOAc = 5:1) to provide the titled compound *proximal*-**19a** as a brown oil (39 mg, 53%) and its regiochemistry was determined by NOESY spectra. ^{1}H NMR (500 MHz, $CDCl_3$) δ: 2.76 (3 H, s), 3.21–3.25 (5 H, m), 3.42 (2 H, t, *J* = 5.5Hz), 5.78 (2 H, s), 6.55 (1 H, s), 7.23–7.37 (5 H, m), 8.31 (1 H, s). ^{13}C NMR (125 MHz, $CDCl_3$) δ: 34.5, 40.8, 47.8, 52.0, 57.6, 96.5, 122.3, 127.5, 128.4, 129.0, 129.7, 134.7, 135.0, 141.9, 147.3, 169.5. IR (neat): 2924, 2853, 1642, 1455 cm^{-1}. HRMS (MALDI) Calcd for $C_{18}H_{19}N_5ONa$ $[M+Na]^+$: 344.1482, found 344.1476.

Following General Procedure C, a mixture of CsF (60 mg, 0.40 mmol), benzyl azide **6b** (14 μL, 0.11 mmol), 1,3-dimethyl-2-imidazolidinone **6l** (33 μL, 0.30 mmol), and a mixture of 1,4-bis(trifluoromethanesulfonyloxy)-2,5-bis(trimethylsilyl)benzene **15** (52 mg, 0.10 mmol) was stirred in MeCN (1.0 mL) for 30 min and 14 h at rt. The crude product (*distal*-**19a**/*proximal*-**19a** = 74:26, determined by 400 MHz ^{1}H NMR analysis) was purified by PTLC (CH_2Cl_2/EtOAc = 5:1) to provide the titled compound *proximal*-**19a** with *distal*-**19a** as a brown oil (12 mg, 37%).

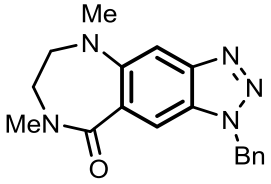

distal-**19a**

1-Benzyl-5,8-dimethyl-5,6,7,8-tetrahydro-[1,2,3]triazolo[4',5':4,5]benzo[1,2-*e*][1,4]diazepin-9(1*H*)-one (*distal*-19a) (Table 2, entry 1-2) was obtained from the above-mentioned reaction

mixture (Procedure B) as a brown oil (13 mg, 18%) and its regiochemistry was determined by NOESY spectra. ^{1}H NMR (500 MHz, $CDCl_3$) δ: 2.89 (3 H, s), 3.20–3.23 (5 H, m), 3.37 (2 H, t, J = 5.5 Hz), 5.79 (2 H, s), 7.28–7.34 (5 H, m), 7.46 (1 H, s), 7.71 (1 H, s). ^{13}C NMR (125 MHz, $CDCl_3$) δ: 34.5, 40.9, 48.1, 52.4, 57.7, 107.1, 111.4, 127.7, 128.5, 128.8, 129.0, 133.3, 134.6, 144.6, 148.3, 169.5. IR (neat): 2924, 2852, 1645, 1498 cm^{-1}. HRMS (MALDI) Calcd for $C_{18}H_{19}N_5ONa$ $[M+Na]^+$: 344.1482, found 344.1480.

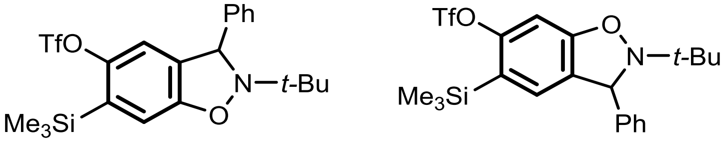

distal-**17b** *proximal*-**17b**

2-(*tert*-Butyl)-3-phenyl-6-(trimethylsilyl)-2,3-dihydrobenzo[*d*]isoxazol-5-yl trifluoromethanesulfonate (*distal*-17b) and 2-(*tert*-butyl)-3-phenyl-5-(trimethylsilyl)-2,3-dihydrobenzo[*d*]isoxazol-6-yl trifluoromethanesulfonate (*proximal*-17b) (Table 2, entry 2-1): Following General Procedure A, a mixture of CsF (0.46 g, 3.0 mmol), *N*-*tert*-butylphenyl nitrone **6d** (0.53 g, 3.0 mmol), and 1,4-bis(trifluoromethanesulfonyloxy)-3,5-bis(trimethylsilyl)benzene **15** (0.52 g, 1.0 mmol) was stirred in MeCN (10 mL) for 40 min at rt. The crude product (*distal*-**17b**/*proximal*-**17b** = 73:27, determined by 500 MHz ^{1}H NMR analysis) was purified by column chromatography on silica gel (hexane/EtOAc = 8:1) to provide a mixture of titled compounds *distal*-**17b** and *proximal*-**17b** as a pale yellow oil (0.34 g, 71%) and regiochemistry was determined by NOESY spectra. ^{1}H NMR (300 MHz, $CDCl_3$) δ: 0.25 (9/4 H, s), 0.33 (27/4 H, s), 1.17 (9/4 H, s), 1.18 (27/4 H, s), 5.57 (1/4 H, s), 5.60 (3/4 H, s), 6.80 (3/4 H, brs), 6.81 (1/4 H, s), 6.88 (3/4 H, s), 6.92 (1/4 H, brs), 7.27–7.42 (20/4 H, m). ^{13}C NMR (125 MHz, $CDCl_3$) δ: –0.83, –0.77, 25.4, 25.4, 61.2, 61.4, 66.5, 66.8, 99.2, 112.8, 115.6, 115.8, 118.3 (q, J = 319 Hz), 123.1, 127.2, 127.3, 127.7, 127.8, 128.8, 129.5, 130.1, 132.9, 133.6, 142.6, 142.9, 148.3, 154.9, 155.2, 158.3. ^{19}F NMR (376 MHz, $CDCl_3$) δ: –73.8 (proximal), –73.9 (distal). IR (neat): 2976, 1418 cm^{-1}. HRMS (MALDI) Calcd for $C_{21}H_{27}F_3NO_4SSi$ $[M+H]^+$: 474.1377, found 474.1377.

[1.4 gram scale reaction] Following General Procedure A, a mixture of CsF (0.91 g, 6.0 mmol), *N*-*tert*-butylphenyl nitrone **6d** (1.1 g, 6.0 mmol), and 1,4-bis(trifluoromethanesulfonyloxy)-3,5-bis(trimethylsilyl)benzene **15** (1.4 g, 2.0 mmol) was stirred in MeCN (20 mL) for 40 min at rt. The crude product (*distal*-**17b**/*proximal*-**17b** = 74:26, determined by 400 MHz ^{1}H NMR analysis) was

purified by column chromatography on silica gel (hexane/EtOAc = 8:1) to provide a mixture of titled compounds *distal*-**17b** and *proximal*-**17b** as a pale yellow oil (0.69 g, 73%). ^{1}H NMR spectra data were identical with those of a mixture of titled compounds *distal*-**17b** and *proximal*-**17b** obtained by the reactin in a small scale shown above.

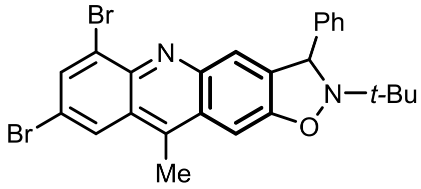

distal-**19b**

6,8-Dibromo-2-(*tert*-butyl)-10-methyl-3-phenyl-2,3-dihydroisoxazolo[5,4-*b*]acridine (*distal*-19b) (Table 2, entry 2-2): Following General Procedure B, a mixture of CsF (49 mg, 0.30 mmol), 2-amino-3,5-dibromoacetophenone **6g** (0.10 g, 0.30 mmol), and a mixture of *distal*- and *proximal*-**17b** (47 mg, 0.10 mmol, obtained from entry 2-1 of Table 2) was stirred in MeCN (1.0 mL) for 14 h at rt. The crude product (*distal*-**19b**/*proximal*-**19b** = 70:30, determined by 400 MHz ^{1}H NMR analysis) was purified by PTLC (hexane/EtOAc = 5:1) to provide the titled compound *distal*-**19b** as a yellow solid (14 mg, 40%) and its regiochemistry was determined by NOESY spectra. Mp: 207–209 °C. ^{1}H NMR (500 MHz, $CDCl_3$) δ: 1.25 (9 H, s), 2.94 (3 H, s), 5.75 (1 H, s), 7.28 (1 H, t, *J* = 7.0 Hz), 7.35 (2 H, dd, *J* = 7.0 Hz, 7.0 Hz), 7.36 (1 H, s), 7.49 (2 H, d, *J* = 7.0 Hz), 7.85 (1 H, s), 8.09 (1 H, d, *J* = 1.5 Hz), 8.29 (1 H, d, *J* = 1.5 Hz). ^{13}C NMR (125 MHz, $CDCl_3$) δ: 14.3, 25.6, 61.5, 66.4, 95.3, 118.5, 125.6, 126.2, 126.6, 126.8, 127.2, 127.4, 127.9, 128.9, 134.7, 139.4, 141.5, 141.7, 142.2, 146.3 156.0. IR (neat): 2980, 1450 cm^{-1}. HRMS (MALDI) Calcd for $C_{25}H_{23}N_2OBr_2$ $[M+H]^+$: 525.0172, found 525.0176.

[0.7 g scale reaction] Following General Procedure B, a mixture of CsF (0.67 g, 4.4 mmol), 2-amino-3,5-dibromoacetophenone **6g** (1.3 g, 4.4 mmol), and a mixture of *distal*- and *proximal*-**17b** (0.69 g, 1.5 mmol, obtained from entry 2-1 of Table 2 [1.4 g scale reaction]) was stirred in MeCN (15 mL) for 14 h at rt. The crude product (*distal*-**19b**/*proximal*-**19b** = 67:33, determined by 400 MHz ^{1}H NMR analysis) was purified by column chromatography on silica gel (hexane/CH_2Cl_2 = 2:1) to provide the mixture of titled compound *distal*-**19b** and **6g**. The mixture was purified by trituration using EtOAc to provide the pure *distal*-**19b** as a yellow solid (0.30 g, 39%). Melting point

and [1] H NMR spectra data were identical with those of *distal*-**19b** obtained by the reactin in a small scale shown above.

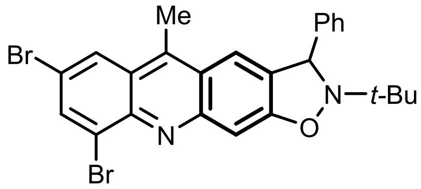

proximal-**19b**

7,9-Dibromo-2-(*tert*-butyl)-5-methyl-3-phenyl-2,3-dihydroisoxazolo[4,5-*b*]acridine (*proximal*-19b) (Table 2, entry 2-2) was obtained from the above-mentioned reaction mixture as a pale yellow oil (5.7 mg, 17%) and its regiochemistry was determined by NOESY spectra. ^{1}H NMR (500 MHz, $CDCl_3$) δ: 1.23 (9 H, s), 2.90 (3 H, s), 5.74 (1 H, s), 7.32 (1 H, t, *J* = 7.5 Hz), 7.39 (2 H, t, *J* = 7.5 Hz), 7.48 (2 H, d, *J* = 7.5 Hz), 7.52 (1 H, s), 7.71 (1 H, s), 8.15 (1 H, d, *J* = 2.0 Hz), 8.27 (1 H, d *J* = 2.0 Hz). ^{13}C NMR (125 MHz, $CDCl_3$) δ: 14.3, 25.6, 61.7, 66.3, 102.0, 117.0, 118.9, 122.7, 125.6, 126.2, 126.6, 127.4, 127.9, 129.0, 135.7, 137.7, 141.7, 142.8, 143.8, 150.7, 159.5. IR (neat): 2971, 1640 cm^{-1}. HRMS (MALDI) Calcd for $C_{25}H_{23}N_2OBr_2$ $[M+H]^+$: 525.0172, found 525.0153.

[0.7 g scale reaction] The titled compound *proximal*-**19b** was obtained from the above-mentioned reaction mixture [0.7 g scale reaction] as a yellow solid (99 mg, 13%). Mp: 224–225 °C. ^{1}H NMR spectra data were identical with those of *proximal*-**19b** obtained by the reactin in a small scale shown above.

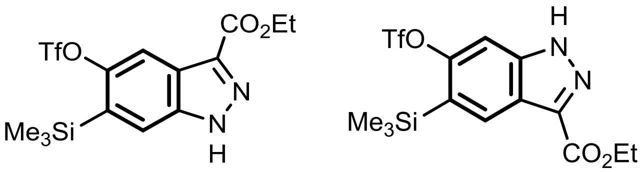

distal-**17c** *proximal*-**17c**

Ethyl 5-(trifluoromethanesulfonyloxy)-6-(trimethylsilyl)-1*H*-indazole-3-carboxylate (*distal*-17c) and ethyl 6-(trifluoromethanesulfonyloxy)-5-(trimethylsilyl)-1*H*-indazole-3-carboxylate (*proximal*-17c) (Table 2, entry 3-1): Following General Procedure A, a mixture of CsF (0.46 g, 3.0 mmol), ethyl diazoacetate **6k** (0.69 mL, 3.0 mmol), and 1,4-bis(trifluoromethanesulfonyloxy)-3,5-bis(trimethylsilyl)benzene **15** (0.53 g, 1.0 mmol) was stirred in MeCN (10 mL) for 15 min at rt. The crude product (*distal*-**17c**/*proximal*-**17c** = 24:76, determined by 400 MHz ^{1}H NMR analysis) was

purified by column chromatography on silica gel (hexane/EtOAc = 4:1) to provide the mixture of titled compounds *distal*- and *proximal*-**17c** as a colorless solid (0.27 g, 66%) and its regiochemistry was determined by HMQC and HMBC spectra. Mp: 223–226 °C. ^{1}H NMR (500 MHz, $CDCl_3$) δ: 0.46 (9/4 H, s), 0.49 (27/4 H, s), 1.54–1.59 (3 H, m), 4.57–4.64 (2 H, m), 8.14 (1/4 H, s), 8.35 (3/4 H, s), 8.37 (3/4 H, s), 8.43 (1/4 H, s). ^{13}C NMR (125 MHz, $CDCl_3$) δ: –0.8, 14.1, 14.3, 61.5, 61.6, 103.6, 111.6, 118.6 (q, J = 320 Hz), 120.7, 121.0, 122.7, 127.5, 129.8, 132.8, 136.3, 136.7, 140.3, 142.4, 151.2, 154.3, 163.0, 163.1. ^{19}F NMR (376 MHz, $CDCl_3$) δ: –73.5 (distal), –73.6 (proximal). IR (neat): 3257, 1729, 1418 cm^{-1}. HRMS (MALDI) Calcd for $C_{14}H_{17}F_3N_2O_5NaSSi$ $[M+H]^+$: 433.0472, found 433.0477.

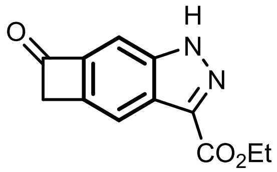

distal-**19c'**

Ethyl 6-oxo-5,6-dihydro-1*H*-cyclobuta[*f*]indazole-3-carboxylate (*distal*-19c') (Table 2, entry 3-2): A stirrer bar was placed into the flask with obtained above obtained a mixture of *distal*- and *proximal*-**17c** (20 mg, 50 μmol, obtained from entry 3-1 of Table 2). The flask was equipped with a three-way stopcock and evacuated and back-filled with Ar. MeCN (0.50 mL) and 1,1-dimethoxyethylene **6m** (13 mg, 0.15 mmol) were added into the flask via a syringe. Then, CsF (23 mg, 0.15 mmol) was quickly added to the flask. The mixture was stirred for 20 h at rt. To the mixture was added a saturated aquous $NaHCO_3$ solution and extracted with EtOAc thrice. The combined organic phase was dried over Na_2SO_4 and organic solvents were evaporated to give a crude product. The crude product with PPTS (25 mg, 0.10 mmol) was stirred for 22 h in acetone (0.50 mL). To the mixture was added a saturated aquous $NaHCO_3$ solution and extracted with EtOAc thrice. The combined organic phase was dried over Na_2SO_4 and organic solvents were evaporated to give a crude material. The mixture (*distal*-**19c'**/*proximal*-**19c'** = 76:24, determined by 300 MHz ^{1}H NMR analysis) was purified by PTLC (toluene/acetone = 5:1) to provide the titled compound *distal*-**19c'** [colorless solid (2.3 mg)] and a mixture of *distal*-**19c'** and *proximal*-**19c'** (2:1, 7.6 mg), and its regiochemistry was determined by NOESY spectra (total 83%). Mp: 222–224 °C. ^{1}H NMR (500 MHz, DMSO-d_6) δ: 1.38 (3 H, t, J = 7.0 Hz), 4.07 (2 H, s), 4.40 (2 H, q, J = 7.0 Hz),

7.78 (1 H, s), 8.17 (1 H, s). ^{13}C NMR (125 MHz, DMSO-d_6) δ: 14.4, 50.8, 60.7, 103.6, 115.5, 127.2, 141.3, 142.2, 146.3, 162.1, 190.3. IR (neat): 3274, 2922, 1761, 1717, 1471 cm^{-1}. HRMS (MALDI) Calcd for $C_{12}H_{11}N_2O_3$ [M+H]$^+$: 231.0764, found 231.0765.

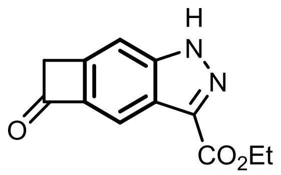

proximal-**19c’**

Ethyl 5-oxo-5,6-dihydro-1*H*-cyclobuta[*f*]indazole-3-carboxylate (*proximal*-19c’) (Table 2, entry 3-2): Following General Procedure B, a mixture of CsF (0.17 g, 1.1 mmol), 1,1-dimethoxyethylene **6m** (97 mg, 1.1 mmol), and above obtained a mixture of *distal*- and *proximal*-**17c** (0.15 g, 0.36 mmol, obtained from entry 3-1 of Table 2) was stirred in MeCN (3.0 mL) for 10 h at rt. The crude product was purified by column chromatography on silica gel (hexane/EtOAc = 1:1) to provide two fractions, Fr.1 (42 mg) containing *distal*-**19c** and *distal*-**19c’**, and Fr.2 (22 mg) containing *proximal*-**19c** and *proximal*-**19c’**. Fr.2 with PPTS (6.0 mg, 25 μmol) was stirred for 9 h in acetone (0.80 mL). To the mixture was added a saturated aquous $NaHCO_3$ solution and extracted with EtOAc thrice. The combined organic phase was dried over Na_2SO_4 and organic solvents were evaporated to give a crude material. The mixture was purified by PTLC (hexane/EtOAc = 1:1) to provide the titled compound *proximal*-**19c’** as a colorless solid (18 mg, 23%) and its regiochemistry was determined by NOESY spectra. Mp: 132–135 °C. ^{1}H NMR (500 MHz, DMSO-d_6) δ: 1.33 (3 H, t, J = 7.0 Hz), 4.01 (2 H, s), 4.35 (2 H, q, J = 7.0 Hz), 7.74 (1 H, d, J = 1.0 Hz), 8.00 (1 H, d, J = 1.0 Hz). ^{13}C NMR (125 MHz, DMSO-d_6) δ: 14.7, 50.9, 61.2, 106.5, 114.3, 123.4, 138.0, 143.3, 145.0, 147.0, 162.2, 189.7. IR (neat): 3250, 2910, 1762, 1722, 1457 cm^{-1}. HRMS (MALDI) Calcd for $C_{12}H_{11}N_2O_3$ [M+H]$^+$: 231.0764, found 231.0765.

Synthesis of 1,3-benzdiyne equivalent candidate 1a:

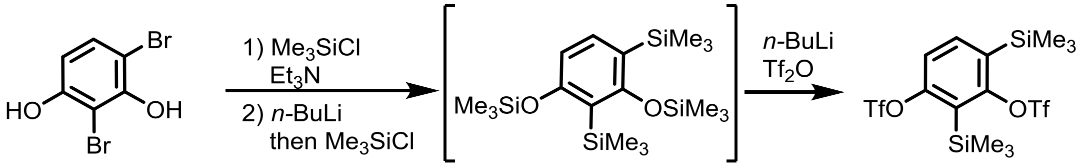

S3[13] (84% in 2 steps) **S4** (63% in 2 steps) **1a** (51%)

Scheme S2 Synthesis of **1a**.

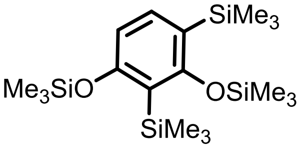

S4

2,4-Bis(trimethylsilyl)-3,5-bis(trimthylsilyloxy)benzene (S4): A round-bottom flask was charged with 2,4-dibromobenzene-1,3-diol (**S3**)[13] (3.6 g, 13 mmol) and capped with an inlet adapter with a 3-way stopcock and then evacuated and back-filled with argon. Anhydrous THF (27 mL, 0.50 M), Et_3N (5.6 mL, 40 mmol) and Me_3SiCl (5.1 mL, 40 mmol) were added via syringes and the reaction mixture was stirred for 1 h at room temperature. The reaction mixture was concentrated under reduced pressure. Hexane was added to the residue and filtrated through a celite cake and washed with hexane. The filtrate was evaporated to give 2,4-dibromo-1,3-phenylene bistrimethylsilyl ether. Without further purification of the obtained material, anhydrous THF (60 mL, 0.22 M) was added to the flask and the mixture was cooled to –78 ºC. *n*-BuLi (1.6 M hexane solution, 25 mL, 40 mmol) was added dropwise at –78 ºC and the reaction was allowed to warm to room temperature and stirred for 3 h. Me_3SiCl (6.5 mL, 54 mmol) was added to the reaction mixture and the mixture was stirred for 1 h. To the reaction mixture was added water for quenching. The mixture was extracted with hexane (this process was repeated three times) and combined organic phase was dried over anhydrous Na_2SO_4. The organic phase was filtered and concentrated under reduced pressure to provide the 2,4-bis(trimethylsilyl)-1,3-bis((trimethylsilyl)oxy)benzene (**S4**) as a colorless oil (3.5 g, 63%). This material was used for next reaction without further purification.

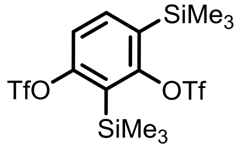

1a

2,4-Bis(trimethylsilyl)-1,3-phenylene bis(trifluoromethanesulfonate) (1a): S4 (0.31 g, 0.76 mmol), anhydrous Et_2O (4.0 mL) was added and the mixture was cooled to –78 °C. *n*-BuLi (1.6 M hexane solution, 1.0 mL, 1.6 mmol) was added dropwise at –78 °C. The reaction was allowed to warm to room temperature and stirred for 2 h. Tf_2O (0.38 mL, 2.3 mmol) was added to the reaction mixture via syringe at 0 °C and the mixture was stirred for 1 h at room temperature. To the reaction mixture was added water for quenching. The mixture was extracted with hexane (this process was repeated three times) and combined organic phase was dried over anhydrous Na_2SO_4. The organic phase was filtered and concentrated under reduced pressure. The crude product was purified by flash column chromatography (hexane) to provide the titled compound **1a** as a colorless oil (0.20 g, 51%). ^{1}H NMR (500 MHz, $CDCl_3$) δ: 0.36 (9 H, s), 0.44 (9 H, s), 7.41 (2 H, d, *J* = 8.5 Hz), 7.65 (2 H, d, *J* = 8.5 Hz). ^{13}C NMR (125 MHz, $CDCl_3$): 0.06, 0.91, 118.4 (q, *J* = 320 Hz), 119.0, 128.5, 135.5, 139.1, 154.8, 156.1. ^{19}F NMR (470 MHz, $CDCl_3$) : –73.4 (s), –71.1 (s). IR (neat): 2950, 1425, 1405 cm^{-1}. HRMS (FAB, NBA): *m*/*z* calcd for $C_{14}H_{20}F_6O_6NaS_2Si_2$ $[M+Na]^+$: 541.0036, found: 541.0048.

Synthesis of 1,3-benzdiyne equivalent 1b:

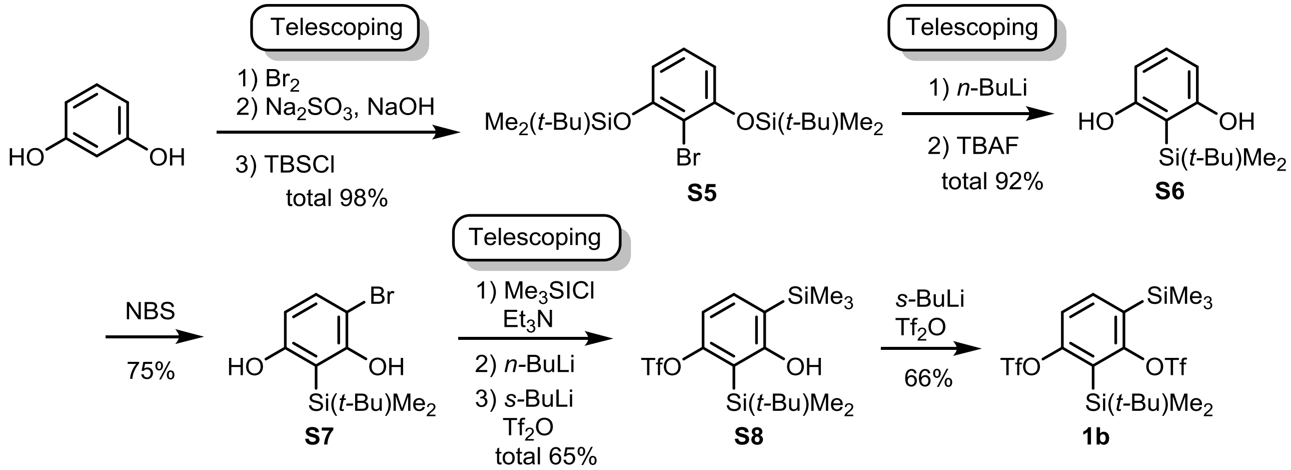

Scheme S3 Improved synthesis of 2-(*tert*-butyldimethylsilyl)benzene-1,3-diol **S6** and the following synthesis of 1,3-benzdiyne equivalent **1b**.

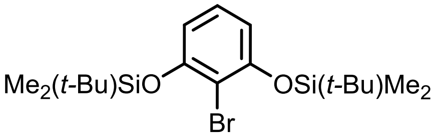

S5

1,3-Bis(*tert*-butyldimethylsilyloxy)-2-bromobenzene (S5): To a solution of resorcinol (22 g, 0.20 mol) in $CHCl_3$ (200 mL, 1.0 M) was added Br_2 (36 mL, 0.70 mol) at 0 °C. After stirring for 5 h at rt, the mixture was concentrated under the reduced pressure to give 2,4,6-tribromo-resorcinol as a white solid (73 g). Without further purification of the obtained material, MeOH (80 mL) was added to the crude material. NaOH (16 g, 0.40 mol) and Na_2SO_3 (50 g, 0.40 mol) in H_2O (400 mL) was added to the mixture at rt. After stirring for 5 min at rt, the reaction was stopped by adding 1N HCl aq. (50 mL) and the mixture was concentrated under reduced pressure and then extracted with EtOAc three times. The combined organic extracts were washed with brine, dried over $MgSO_4$ and concentrated under reduced pressure to give 2-bromoresorcinol as a white solid (41 g). Without further purification of the obtained material, DMF (1.0 L, 0.20 M) were added to the crude material (41 g). Imidazol (41 g, 0.60 mol) and TBSCl (75 g, 0.50 mol) were added to the mixture at rt. After stirring for 6 h at rt, the reaction was stopped by adding H_2O (200 mL) and the mixture was extracted with

hexane. The organic extract was washed with water, dried over $MgSO_4$ and concentrated under reduced pressure. The residue was purified by flash column chromatography on silica gel (hexane) to provide the titled compound **S5** (82 g, 98% in 3 steps) as a colourless solid. Mp: 46–48 °C. ^{1}H NMR (300 MHz, $CDCl_3$) δ: 0.23 (12 H, s), 1.04 (18 H, s), 6,50 (2 H, d, *J* = 8.5 Hz), 6.99 (1 H, t, *J* = 8.5 Hz).

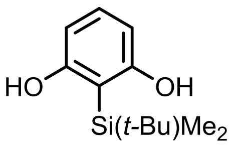

S6

2-(*tert*-Butyldimethylsilyl)benzene-1,3-diol (S6):[14] To a solution of **S5** (42 g, 0.10 mol) in THF (500 mL, 0.20 M) was added 2.5 M *n*-BuLi in hexane (44 mL, 0.11 mol) slowly at –78 °C. After stirring for 90 min, the reaction was stopped by adding a saturated aqueous solution of NH_4Cl (200 mL) and the mixture was extracted with hexane. The combined organic extracts were washed with brine, dried over $MgSO_4$ and concentrated under reduced pressure to provide 2-(*tert*-butyldimethylsilyl)-3-[(tert-butyldimethylsilyl)oxy]phenol as a brown oil (34 g). Without further purification of the obtained material, THF (1.0 L, 0.10 M) was added to the crude material. TBAF in THF (0.10 L, 0.10 mol) was slowly added to the mixture at 0 °C. After stirring for 5 min, the reaction was stopped by adding a saturated aqueous solution of NH_4Cl and the mixture was extracted with EtOAc. The combined organic extracts were washed with brine, dried over $MgSO_4$ and concentrated under reduced pressure. The residue was passed through a silicagel pad using hexane/EtOAc = 5 : 1 and solvents were removed under reduced pressure to produce solid material. The solid was recrystallized from $CHCl_3$ twice to provide the titled compound **S6** as a colorless solid (20 g, 90%).[14] The mother luquid was concentrated under reduced pressure. Hexane was added to the residue and then cooled to –10 ° to provide **S6** as a colorless solid (0.51 g, 2%). Two-step total yield was 92%. Mp: 144–147 °C. ^{1}H NMR (500 MHz, $CDCl_3$) δ: 0.41 (6 H, s), 0.94 (9 H, s), 5.00 (2 OH, brs), 6.29 (2 H, d, *J* = 8.0 Hz), 7.06 (1 H, t, *J* = 8.0 Hz).

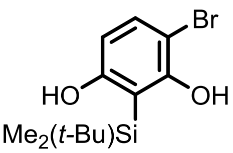

S7

4-Bromo-2-(*tert*-butyldimethylsilyl)resorcinol (S7): A round-bottom flask was charged with 2-(*tert*-butyldimethylsilyl)resorcinol (**S6**)[14] (4.2 g, 19 mmol) and evacuated and back-filled with argon. MeOH (20 mL) and CCl_4 (200 mL) were sequentially added via syringes and NBS (33 g, 19 mmol) was added portionwise to the solution over 5 min at 0 °C with rigorous stirring. After stirred for 3.5 h at 0 °C, a saturated aqueous solution of Na_2SO_3 was added to the reaction mixture for quenching. The mixture was extracted with CH_2Cl_2 (this process was repeated three times) and combined organic phase was dried over anhydrous Na_2SO_4. The organic phase was filtered and concentrated under reduced pressure. The crude product was purified by flash column chromatography (hexane/EtOAc = 6:1) to provide the titled compound **S7** as a colorless solid (4.3 g, 75%). Mp: 70–71 °C. ^{1}H NMR (500 MHz, $CDCl_3$) δ: 0.40 (6 H, s), 0.94 (9 H, s), 5.94 (OH, brs), 5.70 (OH, brs), 6.23 (1 H, d, J = 8.5 Hz), 7.28 (1 H, d, J = 8.5 Hz). ^{13}C NMR (125 MHz, $CDCl_3$) δ: –2.1, 18.5, 26.8, 101.7, 109.3, 109.7, 133.2, 157.2, 161.7. IR (neat): 3506, 2925, 1408 cm^{-1}. HRMS (MALDI): *m*/*z* calcd for $C_{12}H_{18}O_2SiBr$ $[M–H]^-$ 301.0265, found: 301.0260.

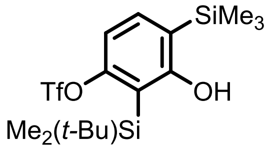

S8

2-(*tert*-Butyldimethylsilyl)-3-hydroxy-1-(trifluoromethanesulfonyloxy)-4-(trimethylsilyl)benzene (S8): An oven-dried flask was charged with 4-bromo-2-(*tert*-butyldimethylsilyl)resorcinol (**S7**) (5.0 g, 17 mmol) and evacuated and back-filled with argon. Anhydrous THF (83 mL 0.20 M) was added through the septum via syringe. Me_3SiCl (6.3 mL, 50 mmol) and Et_3N (6.9 mL, 50 mmol) were sequentially added via syringes and the reaction mixture was stirred for 1 h at room temperature. The reaction mixture was concentrated under reduced pressure. Hexane was added to the residue and filtrated through a celite cake and washed with hexane. The solution was evaporated to give 4-bromo-2-(*tert*-butyldimethylsilyl)resorcinol bis(trimethylsilyl) ether as a colorless oil (6.3 g). Without further purification of the obtained

material, anhydrous THF (0.14 L, 0.12 M) was added to the flask and the mixture was cooled to –78 ºC. *n*-BuLi (2.6 M hexane solution, 11 mL, 28 mmol) was added dropwise at –78 ºC and the reaction was allowed to warm to room temperature and stirred for 1 h. To the reaction mixture was added water for quenching. The mixture was extracted with hexane (this process was repeated three times) and combined organic phase was dried over anhydrous Na_2SO_4. The organic phase was filtered and concentrated under reduced pressure. The residue was used without further purification. Et_2O (0.14 L, 0.12 M) was added to the obtained resdure and the mixture was cooled to –78 °C. 1.1 M *s*-BuLi in hexane (13 mL, 14 mmol) was added dropwise to the mixture and stirred for 15 min at –78 °C. Tf_2O (2.1 mL, 13 mmol) was added for mixture and then mixture was wormed for rt. After stirring for 2 h at rt, the reaction was stopped by adding a saturated aquous $NaHCO_3$ solution and the mixture was extracted with hexane (this process was repeated three times). The combined organic extracts were washed with brine, dried over Na_2SO_4 and concentrated under reduced pressure. The residue was purified by flash column chromatography on silica gel (hexane/CH_2Cl_2 = 20:1) to provide the titled compound **S8** as a yellow oil (3.9 g, 65%). ^{1}H NMR (400 MHz, $CDCl_3$) δ: 0.31 (9 H, s), 0.50 (6 H, s), 0.94 (9 H, s), 5.54 (OH, brs), 6.98 (1 H, d, *J* = 8.5 Hz), 7.40 (1 H, d, *J* = 8.5 Hz). ^{13}C NMR (125 MHz, $CDCl_3$) δ: –1.9, –1.0, 18.5, 26.5, 110.6, 112.8, 118.5 (q, *J* = 320 Hz), 125.1, 138.0, 157.6, 166.9. ^{19}F NMR (376 MHz, $CDCl_3$) δ: –73.5. IR (neat): 3606, 2956 cm^{-1}. HRMS (MALDI): *m*/*z* calcd for $C_{16}H_{27}O_4F_3NaSi_2S$ $[M+Na]^+$ 451.1013, found: 451.1003.

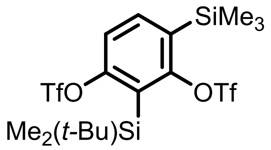

1b

2-(*tert*-Butyldimethylsilyl)-4-(trimethylsilyl)-1,3-bis(trifluoromethanesulfonyloxy)benzene (1b): To a solution of **S8** (2.9 g, 6.7 mmol) in CPME (67 mL, 0.10 M) were cooled to –78 °C then *s*-BuLi (13 mL, 13 mmol) was added dropwise and stirred for 15 min at 0 °C . Then Tf_2O (5.6 mL, 34 mmol) was added. After stirring for 4 h at 0 °C, the reaction was stopped by adding a saturated aquous $NaHCO_3$ solution (50 mL) and the mixture was extracted with hexane (this process was repeated three times). The combined organic extracts were washed with brine, dried over Na_2SO_4 and concentrated under reduced pressure. The residue was purified by flash column chromatography on silica gel (hexane) to provide the titled compound **1b** with a small amount of by-product (2.9 g).

Gel-permeation chromatography (GPC) was applied to remove the by-product completely to provide **1b** as a colorless oil (2.4 g, 66%). ^{1}H NMR (500 MHz, $CDCl_3$) δ: 0.36 (9 H, s), 0.51 (6 H, s), 0.78 (9 H, s), 7.47 (1 H, d, J = 8.5 Hz), 7.64 (1 H, d, J = 8.5 Hz). ^{13}C NMR (125 MHz, $CDCl_3$) δ: –1.7, 0.3, 19.1, 27.1, 118.4, 118.5 (q, J = 322 Hz), 125.1, 135.4, 138.9, 155.7, 156.5. ^{19}F NMR (470 MHz, $CDCl_3$) δ: –72.7, –69.6. IR (neat): 2956, 1427 cm^{-1}. HRMS (MALDI): m/z calcd for $C_{17}H_{26}O_6F_6NaSi_2S_2$ $[M+Na]^+$ 583.0506, found: 583.0506.

Syntheses of 1,4-benzdiyne equivalent 15:[15–17]

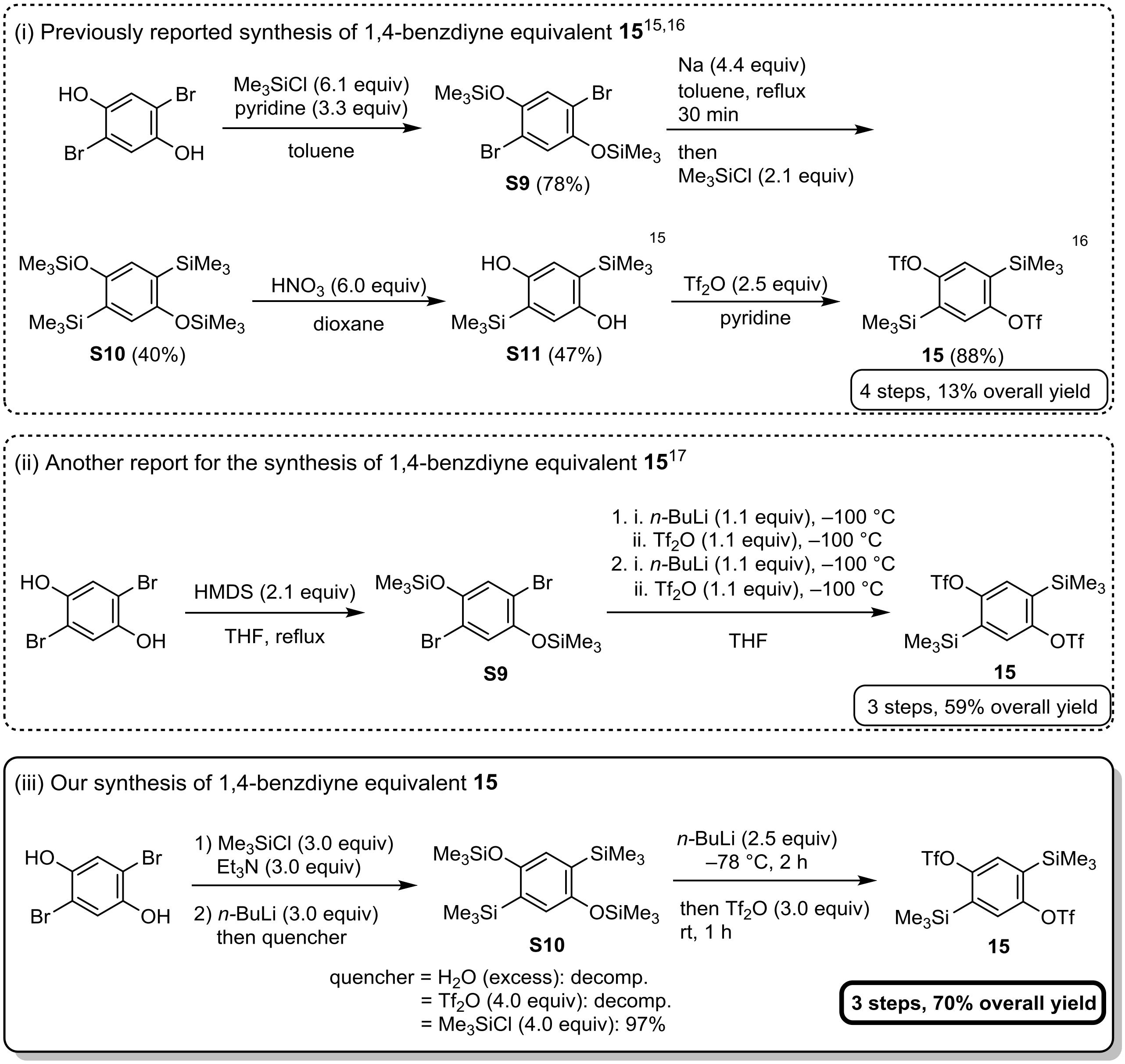

Scheme S4 Comparison among previously reported syntheses (i), (ii) and our synthesis (iii) of **15**.

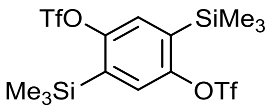

15

2,4-Bis(trimethylsilyl)-1,3-bis(trifluoromethanesulfonyloxy)benzene (15):[1,15–17] To a solution of 2,6-dibromohydroquinone (5.0 g, 19 mmol) in THF (94 mL, 0.20 M) were added Me_3SiCl (6.1 mL, 56 mmol) and Et_3N (7.8 mL, 56 mmol) at rt. After stirring for 1 h at rt, the reaction mixture was evaporated and the recidue was filtered through a celite pad with hexane. The filtrate was concentrated under reduced pressure to provide the 2,5-dibromo-1,4-bis(trimethylsilyioxy)benzene **S9**. Without further purification of the obtained material, THF (94 mL, 0.20 M) was added to the crude material. The mixture was cooled to –78 °C and then *n*-BuLi (2.5 M in hexane, 22 mL, 56 mmol) was added dropwise and it was stirred for 1 h. Then Me_3SiCl (9.5 mL, 74 mmol) was added the mixture and the mixture was wormed to room temperature. After 1 h, the reaction was stopped for water and extracted with hexane (this process was repeated three times). The combined organic extracts were washed with brine, dried over Na_2SO_4 and concentrated under reduced pressure to provide **S10**. Without further purification of the obtained material, Et_2O (94 mL, 0.20 M) was added to the crude material. The mixture was cooled to –78 °C and then *n*-BuLi (2.5 M in hexane, 16 mL, 41 mmol) was added dropwise and it was stirred for 2 h at rt. Then the mixture was cooled to 0 °C and Tf_2O (9.4 mL, 56 mmol) was added the mixture and sirred for 1 h at rt. The reaction was quenched by adding water and extracted with hexane (this process was repeated three times). The combined organic extracts were washed with brine, dried over Na_2SO_4 and concentrated under reduced pressure. The residue was purified by flash column chromatography on silica gel (hexane) and then recrystllized from hexane to provide the titled compound **15** as a colorless solid (6.8 g, 70%). Mp: 105–107 °C. ^1H NMR (300 MHz, $CDCl_3$) δ: 0.38 (18 H, s), 7.44 (2 H, s). ^{13}C NMR (125 MHz, $CDCl_3$) δ: –1.28, 118.4 (q, *J* = 322 Hz), 126.9, 137.4, 153.0. ^{19}F NMR (470 MHz, $CDCl_3$) δ: –81.7. IR (neat): 2964, 1418 cm^{-1}. HRMS (FAB, NBA): *m*/*z* calcd for $C_{14}H_{20}O_6F_6NaSi_2S_2$ $[M+Na]^+$ 541.0036, found: 541.0062 .

Cartesian coordinates of optimized structures of 4,5-benzotriazolyne 5b and 4,5-indolyne 5b' by DFT calculation [B3LYP/6-31G(d)]:

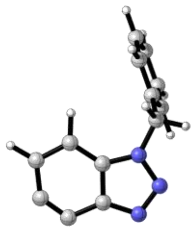

5b

1	C	2.3484460	2.3419730	-0.0399380
2	C	1.3396390	1.4822160	-0.4942080
3	C	1.4759020	0.0894790	-0.2865460
4	C	2.5904930	-0.5152420	0.3750240
5	C	3.5443220	0.4186870	0.7904050
6	C	3.3810980	1.6361680	0.5810600
7	H	2.2877920	3.4161970	-0.1720430
8	H	0.4611040	1.8906510	-0.9835090
9	N	1.2837160	-2.1422850	-0.1698670
10	N	2.4091070	-1.8700980	0.4139500
11	N	0.6982580	-0.9861870	-0.6061380
12	C	-0.5853580	-1.0492630	-1.2955540
13	H	-0.4836100	-0.5619990	-2.2717590
14	H	-0.7542580	-2.1161760	-1.4712090
15	C	-1.7276770	-0.4313490	-0.5109220
16	C	-1.9799990	-0.8298620	0.8084110
17	C	-2.5587700	0.5226200	-1.1060870
18	C	-3.0456230	-0.2792270	1.5177440
19	H	-1.3363470	-1.5698670	1.2774210
20	C	-3.6305960	1.0712370	-0.3976700
21	H	-2.3717530	0.8355990	-2.1313910
22	C	-3.8743200	0.6722670	0.9161170
23	H	-3.2316630	-0.5939450	2.5409580

24	H	-4.2691870	1.8114970	-0.8719660
25	H	-4.7049990	1.0998140	1.4708760

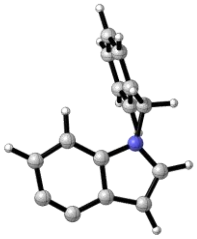

5b'

1	C	3.5263030	0.6319310	0.7016330
2	C	3.2966670	1.8297510	0.4435120
3	C	2.1932010	2.4024690	-0.1915520
4	C	1.2547520	1.4260890	-0.5626250
5	C	1.4956820	0.0626440	-0.2848650
6	C	2.6761940	-0.4288350	0.3868750
7	H	2.0330440	3.4567500	-0.3888170
8	H	0.3334060	1.7313490	-1.0504620
9	N	0.7149840	-1.0462710	-0.5713090
10	C	-0.5651610	-1.0375020	-1.2619440
11	H	-0.4517640	-0.5096690	-2.2170250
12	H	-0.7933870	-2.0813080	-1.5089960
13	C	-1.7126610	-0.4261320	-0.4716260
14	C	-2.7126210	0.2864430	-1.1427080
15	C	-1.8164000	-0.6002400	0.9132900
16	C	-3.8040910	0.8086010	-0.4464500
17	H	-2.6377820	0.4347240	-2.2182930
18	C	-2.9032900	-0.0741830	1.6113190
19	H	-1.0374370	-1.1382250	1.4466140
20	C	-3.9016950	0.6293120	0.9338330
21	H	-4.5715480	1.3615520	-0.9815240

22	H	-2.9689500	-0.2118330	2.6873070
23	H	-4.7467840	1.0398960	1.4796740
24	C	1.3617300	-2.1765840	-0.1042620
25	H	0.9050050	-3.1475000	-0.2431510
26	C	2.5563860	-1.8464990	0.4863730
27	H	3.2613490	-2.5354240	0.9290060

Experimental references:

(1) (a) H. M. Duong, M. Bendikov, H. Steiger, Q. Zhang, G. Sonmez, G. Yamada and G. Wudl, *Org. Lett.*, 2003, **5**, 4433–4436; (b) D. Rodríguez-Lojo, A. Cobas, D. Peña, D. Pérez and E. Guitián, *Org. Lett.*, 2012, **14**, 1363–1365; (c) B. Schuler, S. Collazos, L. Gross, G. Meyer, D. Pérez, E. Guitián and D. Peña, *Angew. Chem. Int. Ed.*, 2014, **53**, 9004–9006. (d) D. Rodríguez-Lojo, D. Peña, D. Pérez and E. Guitián, *Synlett*, 2015**, 26**, 1633–1637.

(2) Houk and Garg et al. have reported that distortions of arynes are useful to predict and understand the regioselectivities of aryne reactions, see: (a) P. H.-Y. Cheong, R. S. Paton, S. M. Bronner, G.-Y. J. Im, N. K. Garg and K. N. Houk, *J. Am. Chem. Soc.*, 2010, **132**, 1267–1269; (b) G.-Y. J. Im, S. M. Bronner, A. E. Goetz, R. S. Paton, P. H.-Y. Cheong, K. N. Houk and N. K. Garg, *J. Am. Chem. Soc.*, 2010, **132**, 17933–17944; (c) S. M. Bronner, J. L. Mackey, K. N. Houk and N. K. Garg, *J. Am. Chem. Soc.*, 2012, **134**, 13966–13969.

(3) For a natural bond orbital (NBO) method, see: (a) K. B. Wiberg, *Tetrahedron*, 1968, **24**, 1083–1096; (b) J. P. Foster and F. Weinhold, *J. Am. Chem. Soc.*, 1980, **102**, 7211–7218; c) A. E. Reed, R. B. Weinstock and F. Weinhold, *J. Chem. Phys.*, 1985, **83**, 735–746.

(4) For recent reports on the NBO analysis of benzynes, see: (a) A. Takagi, T. Ikawa, Y. Kurita, K. Saito, K. Azechi, M. Egi, Y. Itoh, H. Tokiwa, Y. Kita and S. Akai, *Tetrahedron*, 2013, **69**, 4338–4352; (b) A. Takagi, T. Ikawa, K. Saito, S. Masuda, T. Ito and S. Akai, *Org. Biomol. Chem.*, 2013, **11**, 8145–8150.

(5) The natural "localized" molecular orbital (NLMO) based on natural bond orbital (NBO) interpretations could give chemists actual chemical and even quantitative insights without a methodology/basis set-dependency. For a review on NBO analysis, see: A. E. Reed, L. A. Curtiss and F. Weinhold, *Chem. Rev.*, 1988, **88**, 899–926.

(6) Electron density ρ^{i}_{CA} of the ith (reacting π) NBO of carbon atom C_A (A = 1 or 2) was calculated as

$$\rho^{i}_{CA} = n_i \times d_{CA}$$

where n^i is occupancy of the ith NBO and d_{CA} is percentage contribution from each carbon atom C_A for the ith NBO (Fig. S3).

(7) All calculations were performed using the Gaussian 09, Revision D.01, M. J. Frisch, G. W. Trucks, H. B. Schlegel, G. E. Scuseria, M. A. Robb, J. R. Cheeseman, G. Scalmani, V.

Barone, B. Mennucci, G. A. Petersson, H. Nakatsuji, M. Caricato, X. Li, H. P. Hratchian, A. F. Izmaylov, J. Bloino, G. Zheng, J. L. Sonnenberg, M. Hada, M. Ehara, K. Toyota, R. Fukuda, J. Hasegawa, M. Ishida, T. Nakajima, Y. Honda, O. Kitao, H. Nakai, T. Vreven, J. A. Montgomery, Jr., J. E. Peralta, F. Ogliaro, M. Bearpark, J. J. Heyd, E. Brothers, K. N. Kudin, V. N. Staroverov, R. Kobayashi, J. Normand, K. Raghavachari, A. Rendell, J. C. Burant, S. S. Iyengar, J. Tomasi, M. Cossi, N. Rega, J. M. Millam, M. Klene, J. E. Knox, J. B. Cross, V. Bakken, C. Adamo, J. Jaramillo, R. Gomperts, R. E. Stratmann, O. Yazyev, A. J. Austin, R. Cammi, C. Pomelli, J. W. Ochterski, R. L. Martin, K. Morokuma, V. G. Zakrzewski, G. A. Voth, P. Salvador, J. J. Dannenberg, S. Dapprich, A. D. Daniels, Ö. Farkas, J. B. Foresman, J. V. Ortiz, J. Cioslowski, and D. J. Fox, Gaussian, Inc., Wallingford CT, 2009.

(8) The graphics for Fig. S3 were prepared with CYLview, 1.0b: C. Y. Legault, Université de Sherbrooke, 2009 (http://www.cylview.org).

(9) (a) A. R. Evans and G. A. Taylor, *J. Chem. Soc., Perkin Trans. 1*, 1983, 979–983. (b) J. W. Bode, Y. Hachisu, T. Matsuura and K. Suzuki, *Tetrahedron Lett.*, 2003, **44**, 3555–3558; (c) J. W. Bode, Y. Hachisu, T. Matsuura and K. Suzuki, *Org. Lett.*, 2003, **5**, 391–394; (d) T. Matsuura, J. W. Bode, Y. Hachisu and K. Suzuki, *Synlett*, 2003, 1746–1748.

(10) A. Rodriguez and W. J. Moran, *Synthesis*, 2009, 650–654.

(11) M. M. Maluleka and M. J. Mphahlele, *Tetrahedron*, 2013, **69**, 699–704.

(12) W. C. Still, M. Kahn and A. Mitra, *J. Org. Chem.*, 1978, **43**, 2923–2925.

(13) E. Kiehlmann and R. W. Lauener, *Can. J. Chem.*, 1989, **67**, 335–344.

(14) T. Ikawa, H. Kaneko, S. Masuda, E. Ishitsubo, H. Tokiwa and S. Akai, *Org. Biomol. Chem.*, 2015, **13**, 520–526.

(15) H. Bock, S. Nick and K. Z. Ruppert, *Naturforsch*, 1995, **50**, 595–604.

(16) H. M. Duong, M. Bendikov, D. Steiger, Q. Zhang, G. Sonmez, J. Yamada and F. Wudl, *Org. Lett.*, 2003, **5**, 4433–4436.

(17) Very recently, a modified one-pot procedure was reporeted. This method might be better than before;[16] however, careful temperature control appeared to be important around –100 °C and the yield was 59%; see, N. Pavliček, B. Schuler, S. Collazos, N. Moll, D. Pérez, E. Guitián, G. Meyer, D. Peña and L. Gross, *Nat. Chem.*, 2015, **7**, 623–628.

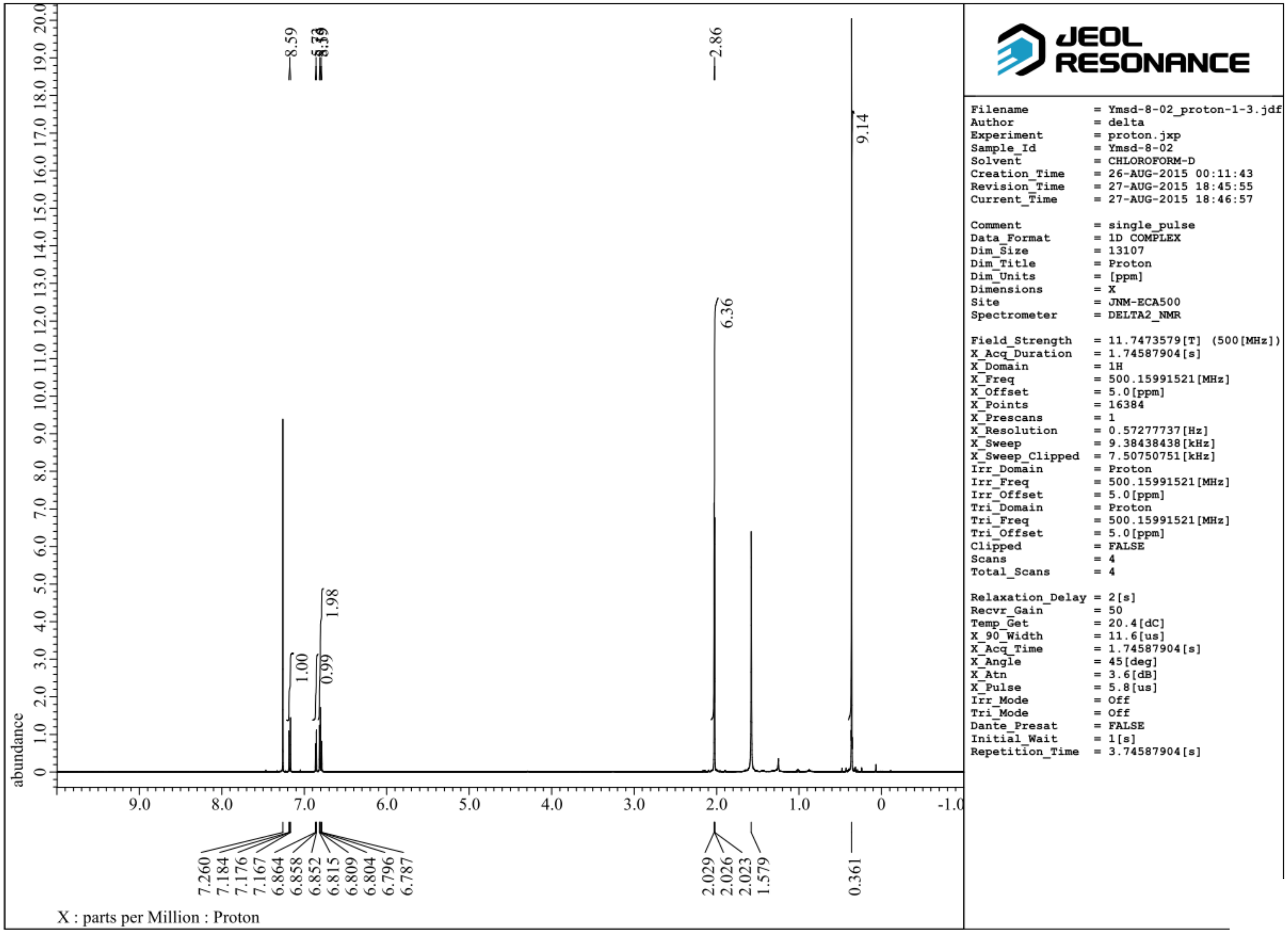

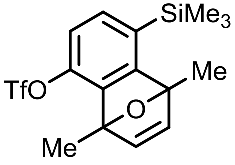

8

You created this PDF from an application that is not licensed to print to novaPDF printer (http://www.novapdf.com)

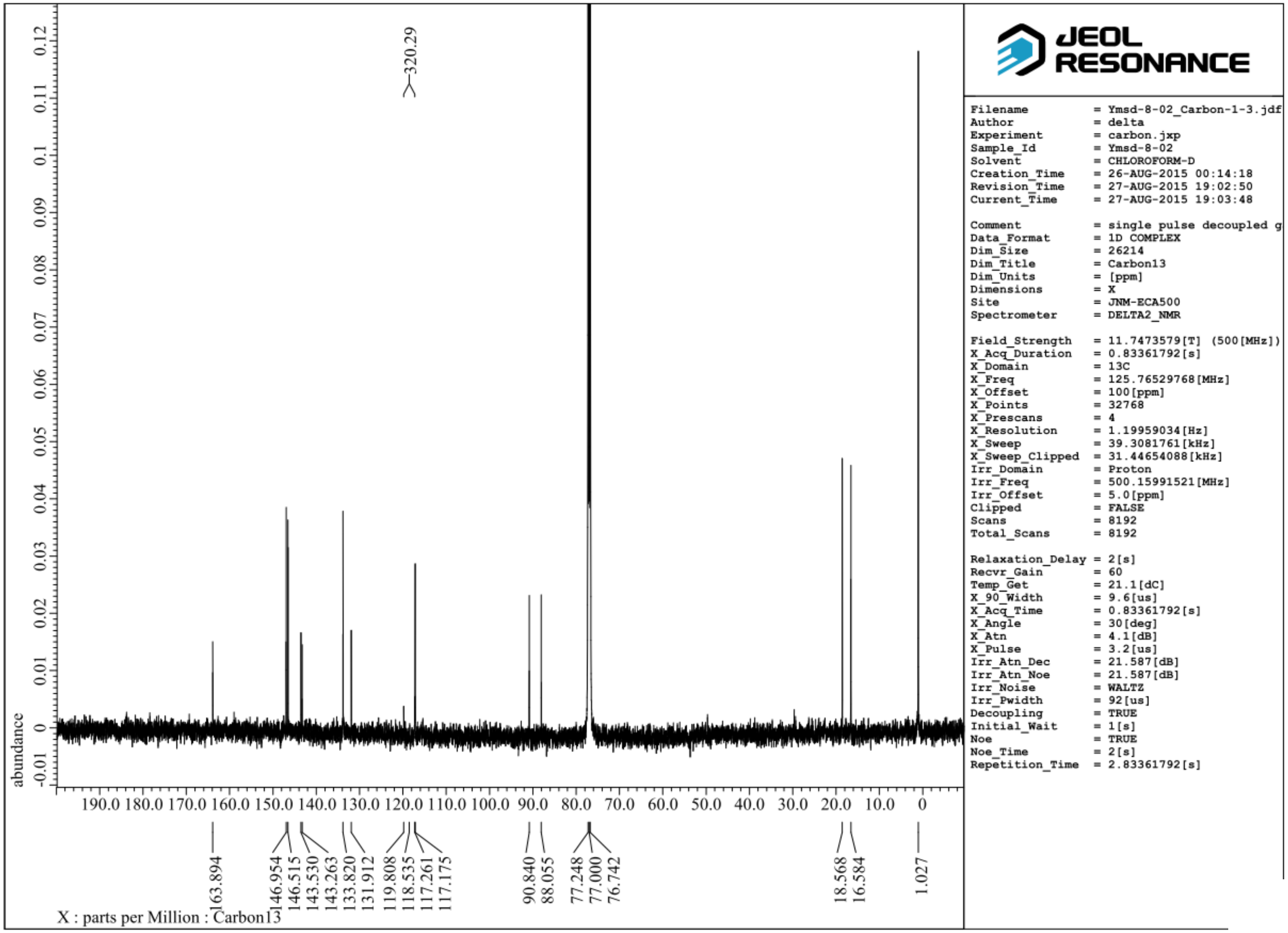

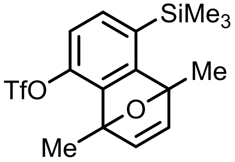

8

You created this PDF from an application that is not licensed to print to novaPDF printer (http://www.novapdf.com)

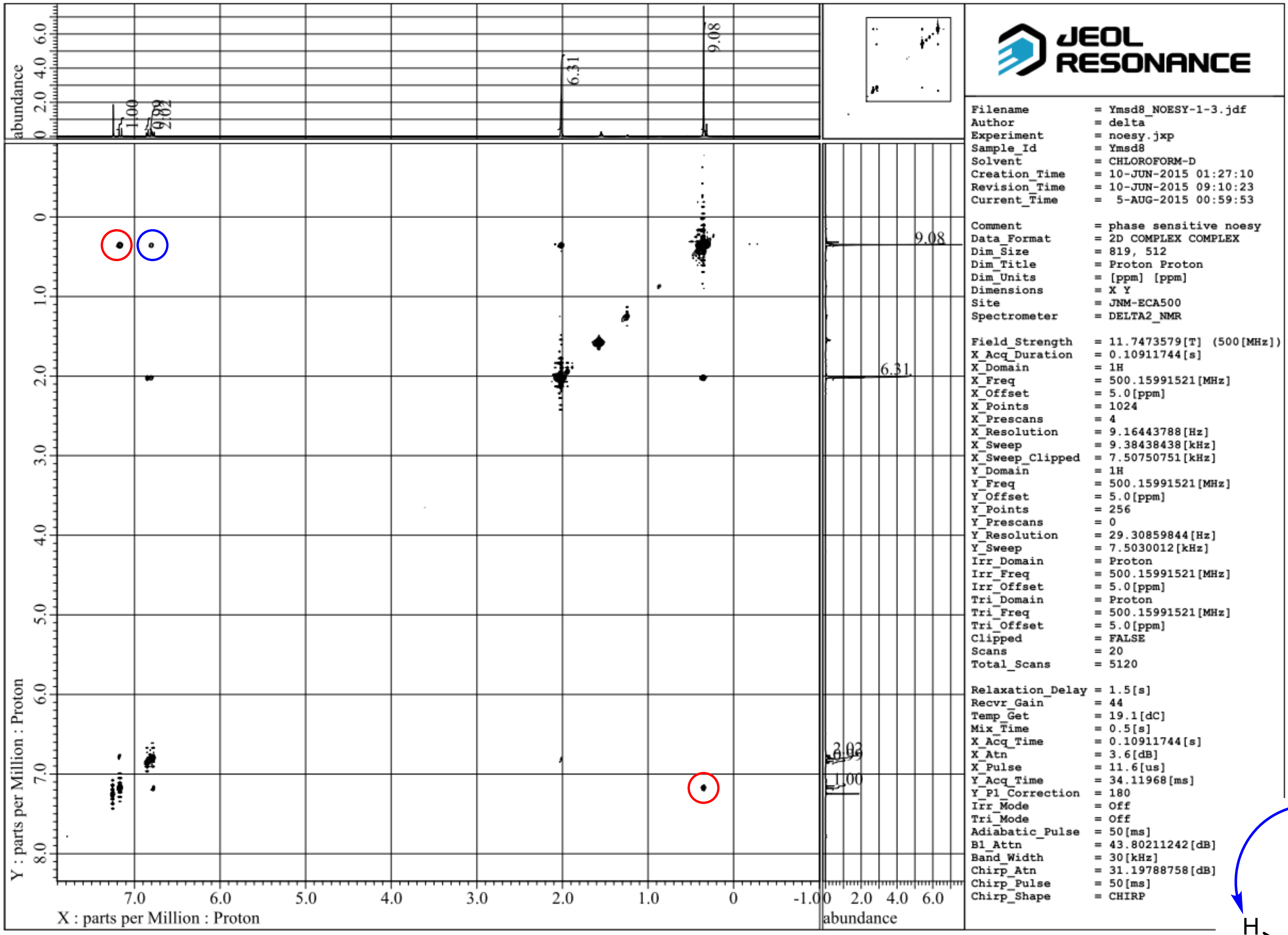

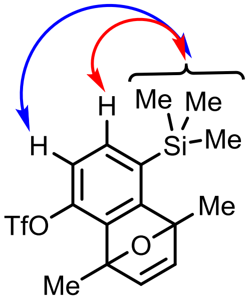

8

You created this PDF from an application that is not licensed to print to novaPDF printer (http://www.novapdf.com)

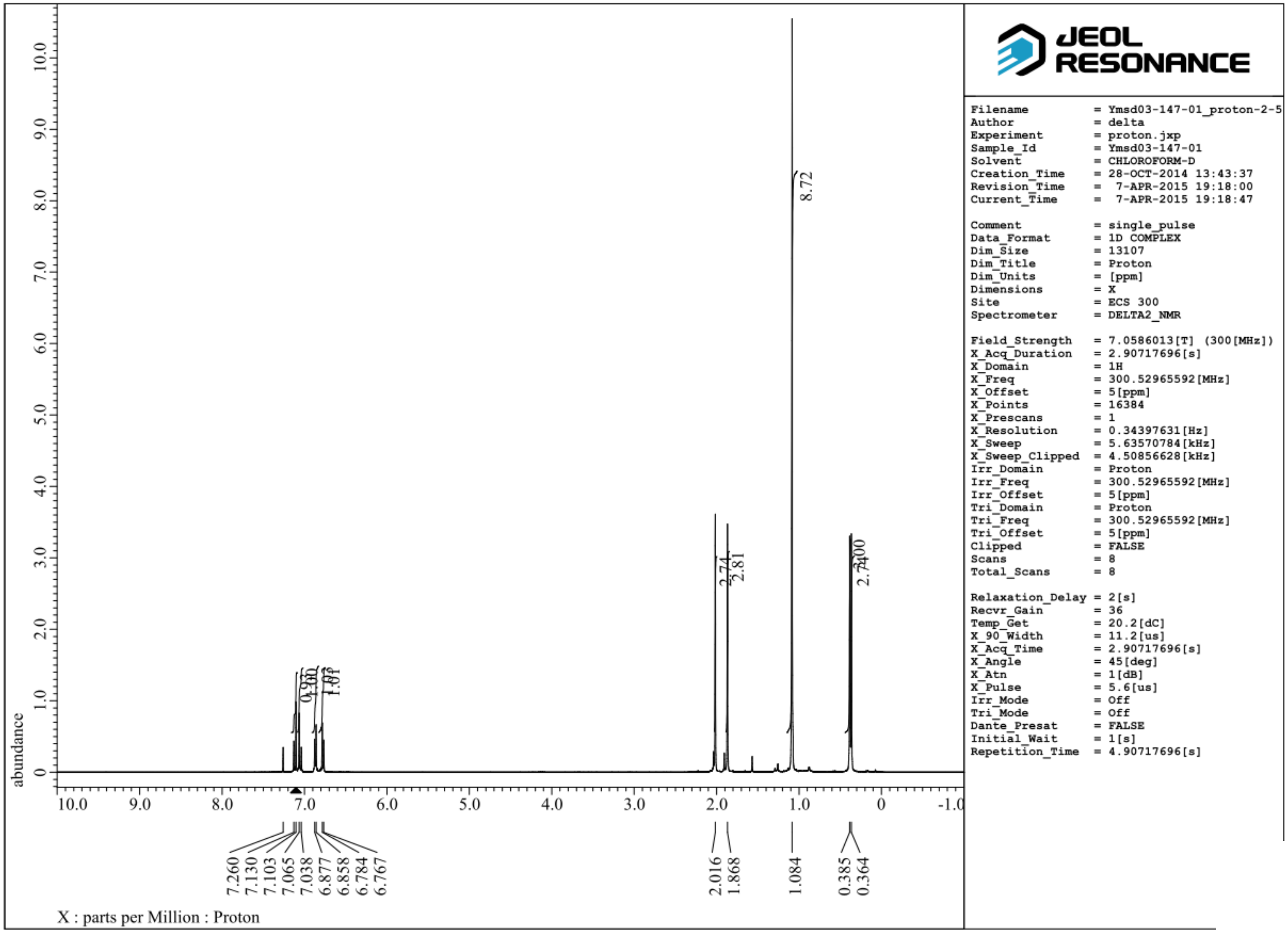

You created this PDF from an application that is not licensed to print to novaPDF printer (http://www.novapdf.com)

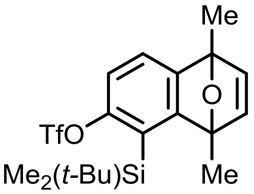

10a

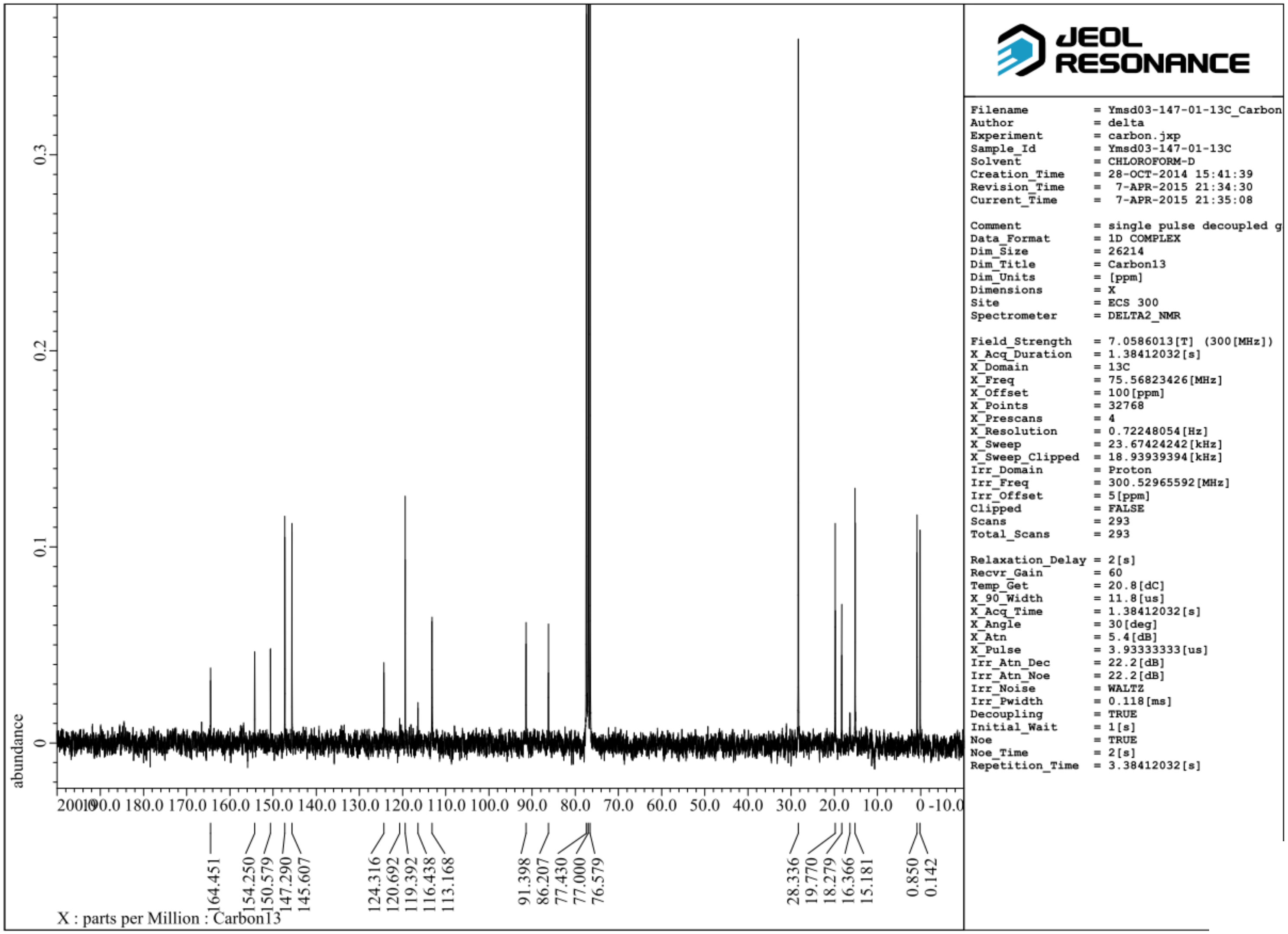

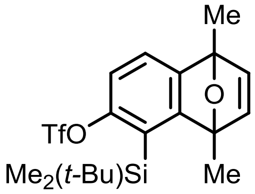

10a

You created this PDF from an application that is not licensed to print to novaPDF printer (http://www.novapdf.com)

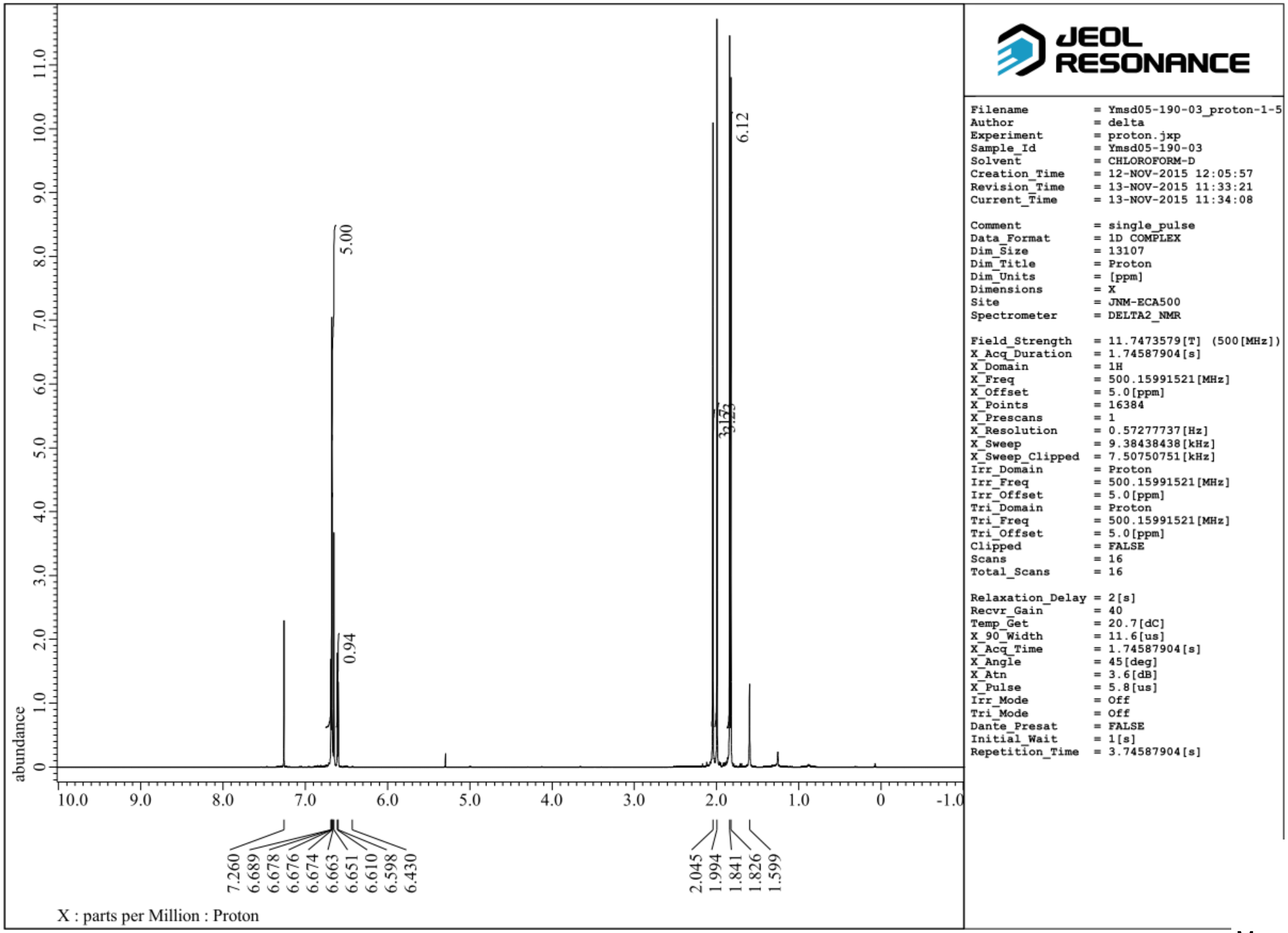

JEOL RESONANCE
Filename = Ymsd05-190-03_proton-1-5
Author = delta
Experiment = proton.jxp
Sample_Id = Ymsd05-190-03
Solvent = CHLOROFORM-D
Creation_Time = 12-NOV-2015 12:05:57
Revision_Time = 13-NOV-2015 11:33:21
Current_Time = 13-NOV-2015 11:34:08
Comment = single_pulse
Data_Format = 1D COMPLEX
Dim_Size = 13107
Dim_Title = Proton
Dim_Units = [ppm]
Dimensions = X
Site = JNM-ECA500
Spectrometer = DELTA2_NMR
Field_Strength = 11.7473579[T] (500[MHz])
X_Acq_Duration = 1.74587904[s]
X_Domain = 1H
X_Freq = 500.15991521[MHz]
X_Offset = 5.0[ppm]
X_Points = 16384
X_Prescans = 1
X_Resolution = 0.57277737[Hz]
X_Sweep = 9.38438438[kHz]
X_Sweep_Clipped = 7.50750751[kHz]
Irr_Domain = Proton
Irr_Freq = 500.15991521[MHz]
Irr_Offset = 5.0[ppm]
Tri_Domain = Proton
Tri_Freq = 500.15991521[MHz]
Tri_Offset = 5.0[ppm]
Clipped = FALSE
Scans = 16
Total_Scans = 16
Relaxation_Delay = 2[s]
Recvr_Gain = 40
Temp_Get = 20.7[dC]
X_90_Width = 11.6[us]
X_Acq_Time = 1.74587904[s]
X_Angle = 45[deg]
X_Atn = 3.6[dB]
X_Pulse = 5.8[us]
Irr_Mode = Off
Tri_Mode = Off
Dante_Presat = FALSE
Initial_Wait = 1[s]
Repetition_Time = 3.74587904[s]
abundance
X : parts per Million : Proton
5.00
0.94
6.12
3.15
3.23
7.260
6.689
6.678
6.676
6.674
6.663
6.651
6.610
6.598
6.430
2.045
1.994
1.841
1.826
1.599

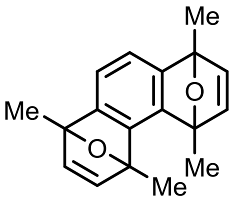

Me
Me
O
O
Me
Me

3a

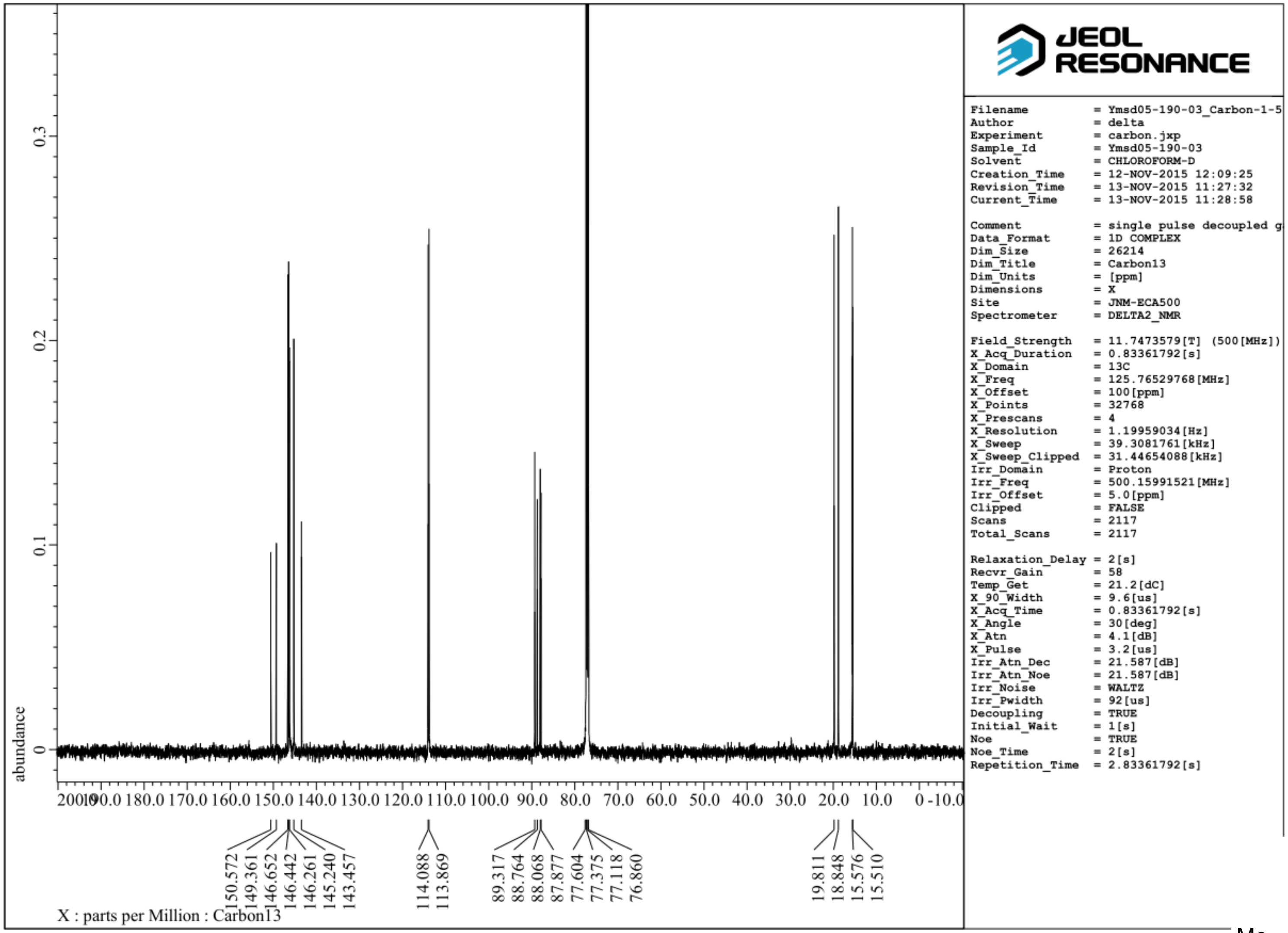
JEOL RESONANCE
Filename = Ymsd05-190-03_Carbon-1-5
Author = delta
Experiment = carbon.jxp
Sample_Id = Ymsd05-190-03
Solvent = CHLOROFORM-D
Creation_Time = 12-NOV-2015 12:09:25
Revision_Time = 13-NOV-2015 11:27:32
Current_Time = 13-NOV-2015 11:28:58
Comment = single pulse decoupled g
Data_Format = 1D COMPLEX
Dim_Size = 26214
Dim_Title = Carbon13
Dim_Units = [ppm]
Dimensions = X
Site = JNM-ECA500
Spectrometer = DELTA2_NMR
Field_Strength = 11.7473579[T] (500[MHz])
X_Acq_Duration = 0.83361792[s]
X_Domain = 13C
X_Freq = 125.76529768[MHz]
X_Offset = 100[ppm]
X_Points = 32768
X_Prescans = 4
X_Resolution = 1.19959034[Hz]
X_Sweep = 39.3081761[kHz]
X_Sweep_Clipped = 31.44654088[kHz]
Irr_Domain = Proton
Irr_Freq = 500.15991521[MHz]
Irr_Offset = 5.0[ppm]
Clipped = FALSE
Scans = 2117
Total_Scans = 2117
Relaxation_Delay = 2[s]
Recvr_Gain = 58
Temp_Get = 21.2[dC]
X_90_Width = 9.6[us]
X_Acq_Time = 0.83361792[s]
X_Angle = 30[deg]
X_Atn = 4.1[dB]
X_Pulse = 3.2[us]
Irr_Atn_Dec = 21.587[dB]
Irr_Atn_Noe = 21.587[dB]
Irr_Noise = WALTZ
Irr_Pwidth = 92[us]
Decoupling = TRUE
Initial_Wait = 1[s]
Noe = TRUE
Noe_Time = 2[s]
Repetition_Time = 2.83361792[s]
abundance
0.3
0.2
0.1
0
200190.0 180.0 170.0 160.0 150.0 140.0 130.0 120.0 110.0 100.0 90.0 80.0 70.0 60.0 50.0 40.0 30.0 20.0 10.0 0 -10.0
150.572
149.361
146.652
146.442
146.261
145.240
143.457
114.088
113.869
89.317
88.764
88.068
87.877
77.604
77.375
77.118
76.860
19.811
18.848
15.576
15.510
X : parts per Million : Carbon13

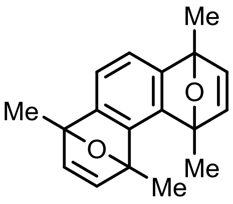
Me
O
Me
O
Me
Me

3a

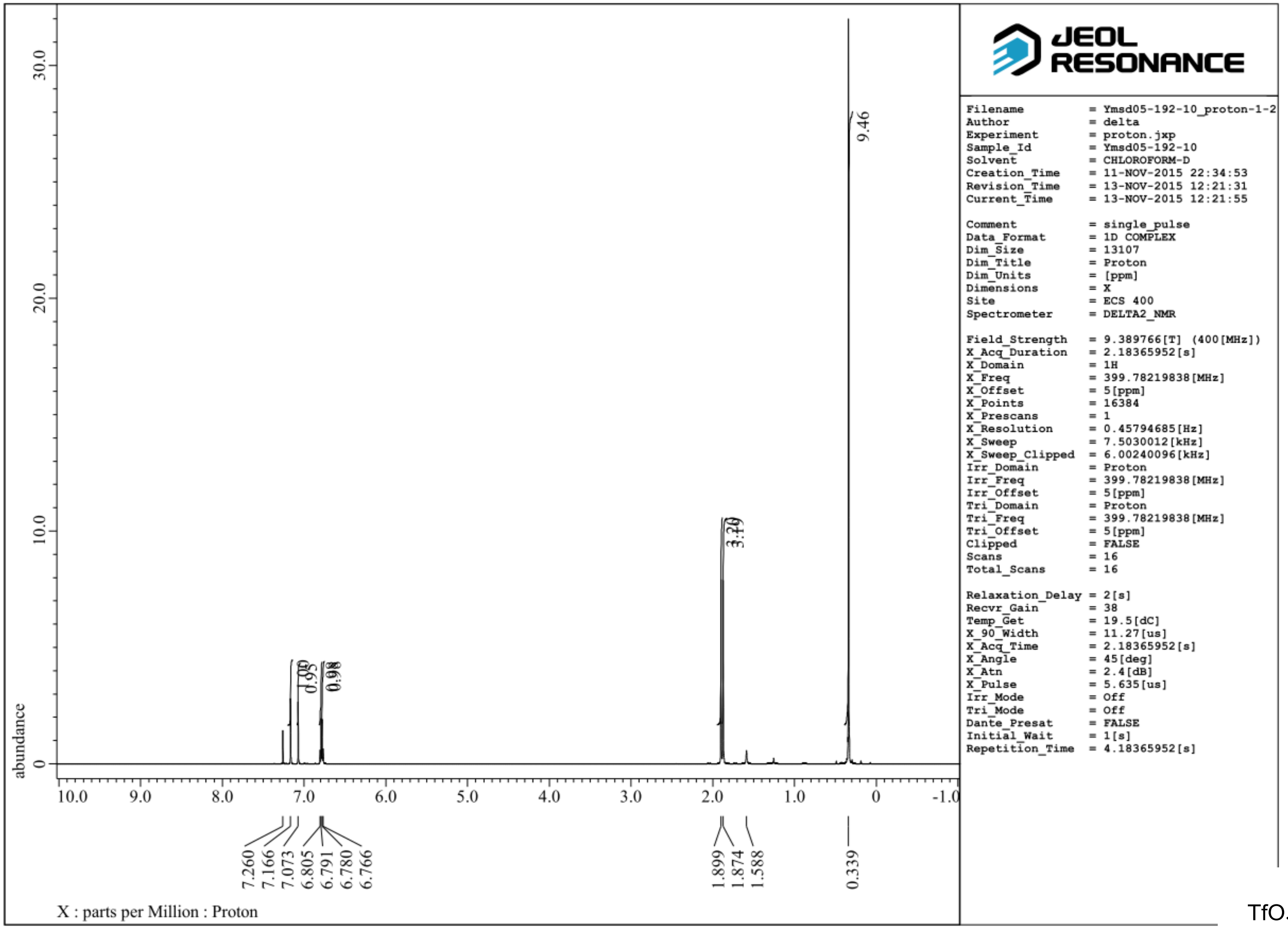

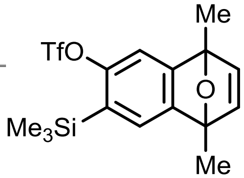

S1

You created this PDF from an application that is not licensed to print to novaPDF printer (http://www.novapdf.com)

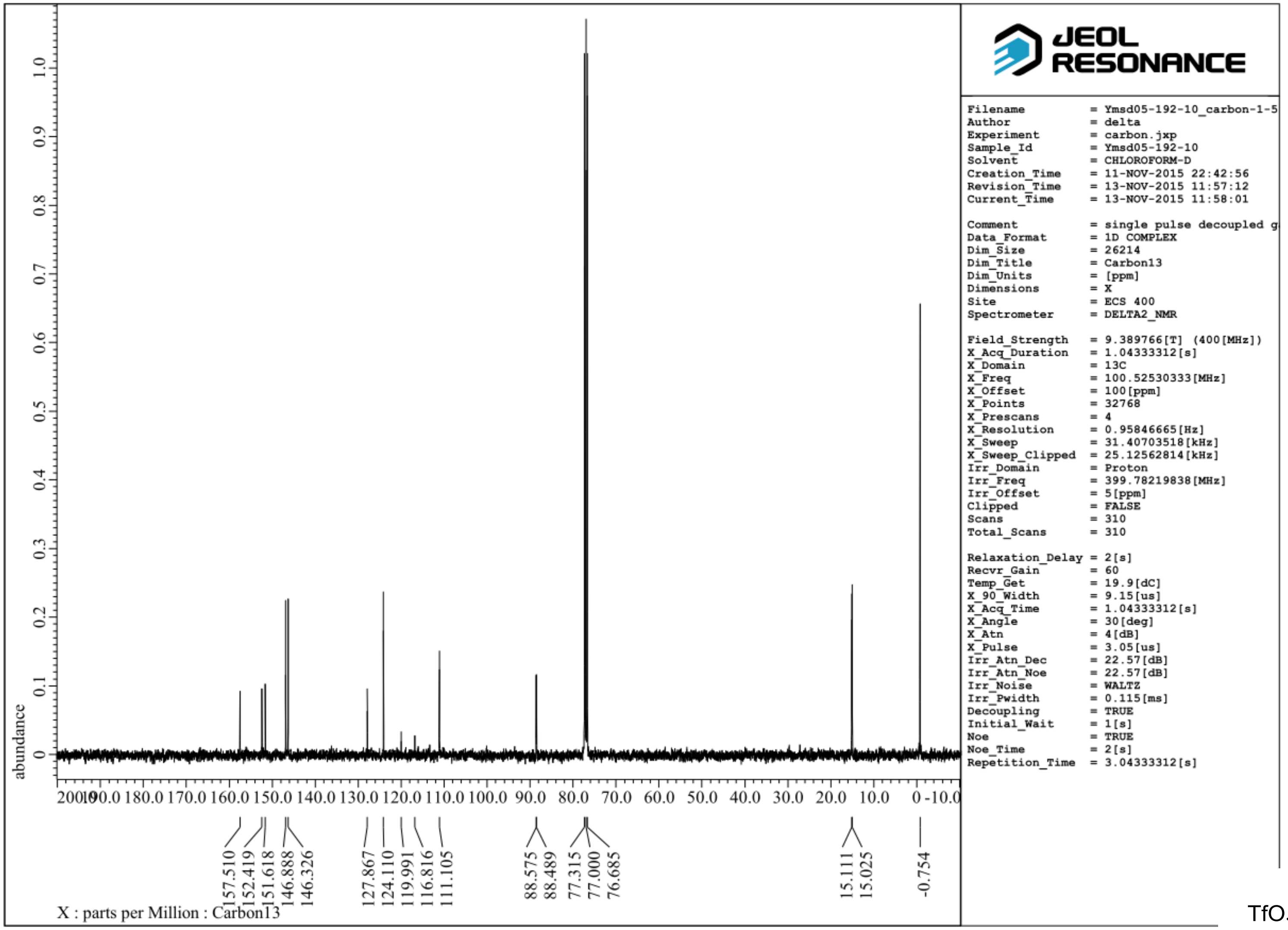

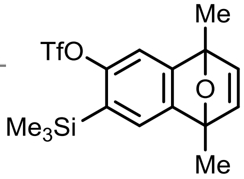

S1

You created this PDF from an application that is not licensed to print to novaPDF printer (http://www.novapdf.com)

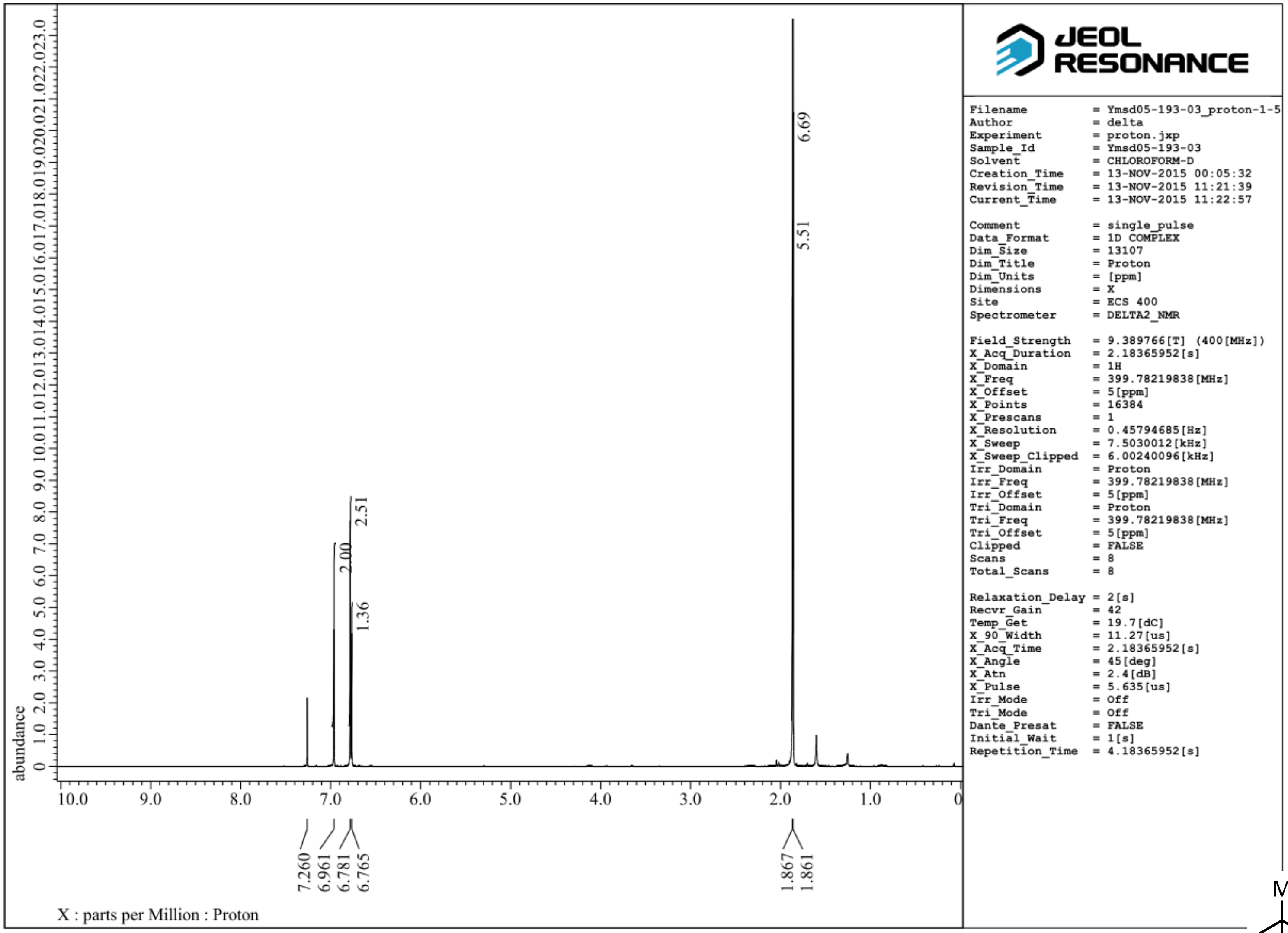

abundance
X : parts per Million : Proton
7.260
6.961
6.781
6.765
1.867
1.861
2.00
2.51
1.36
6.69
5.51
JEOL RESONANCE
Filename = Ymsd05-193-03_proton-1-5
Author = delta
Experiment = proton.jxp
Sample_Id = Ymsd05-193-03
Solvent = CHLOROFORM-D
Creation_Time = 13-NOV-2015 00:05:32
Revision_Time = 13-NOV-2015 11:21:39
Current_Time = 13-NOV-2015 11:22:57
Comment = single_pulse
Data_Format = 1D COMPLEX
Dim_Size = 13107
Dim_Title = Proton
Dim_Units = [ppm]
Dimensions = X
Site = ECS 400
Spectrometer = DELTA2_NMR
Field_Strength = 9.389766[T] (400[MHz])
X_Acq_Duration = 2.18365952[s]
X_Domain = 1H
X_Freq = 399.78219838[MHz]
X_Offset = 5[ppm]
X_Points = 16384
X_Prescans = 1
X_Resolution = 0.45794685[Hz]
X_Sweep = 7.5030012[kHz]
X_Sweep_Clipped = 6.00240096[kHz]
Irr_Domain = Proton
Irr_Freq = 399.78219838[MHz]
Irr_Offset = 5[ppm]
Tri_Domain = Proton
Tri_Freq = 399.78219838[MHz]
Tri_Offset = 5[ppm]
Clipped = FALSE
Scans = 8
Total_Scans = 8
Relaxation_Delay = 2[s]
Recvr_Gain = 42
Temp_Get = 19.7[dC]
X_90_Width = 11.27[us]
X_Acq_Time = 2.18365952[s]
X_Angle = 45[deg]
X_Atn = 2.4[dB]
X_Pulse = 5.635[us]
Irr_Mode = Off
Tri_Mode = Off
Dante_Presat = FALSE
Initial_Wait = 1[s]
Repetition_Time = 4.18365952[s]

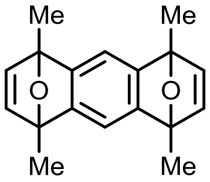

Me
Me
O
O
Me
Me

S2

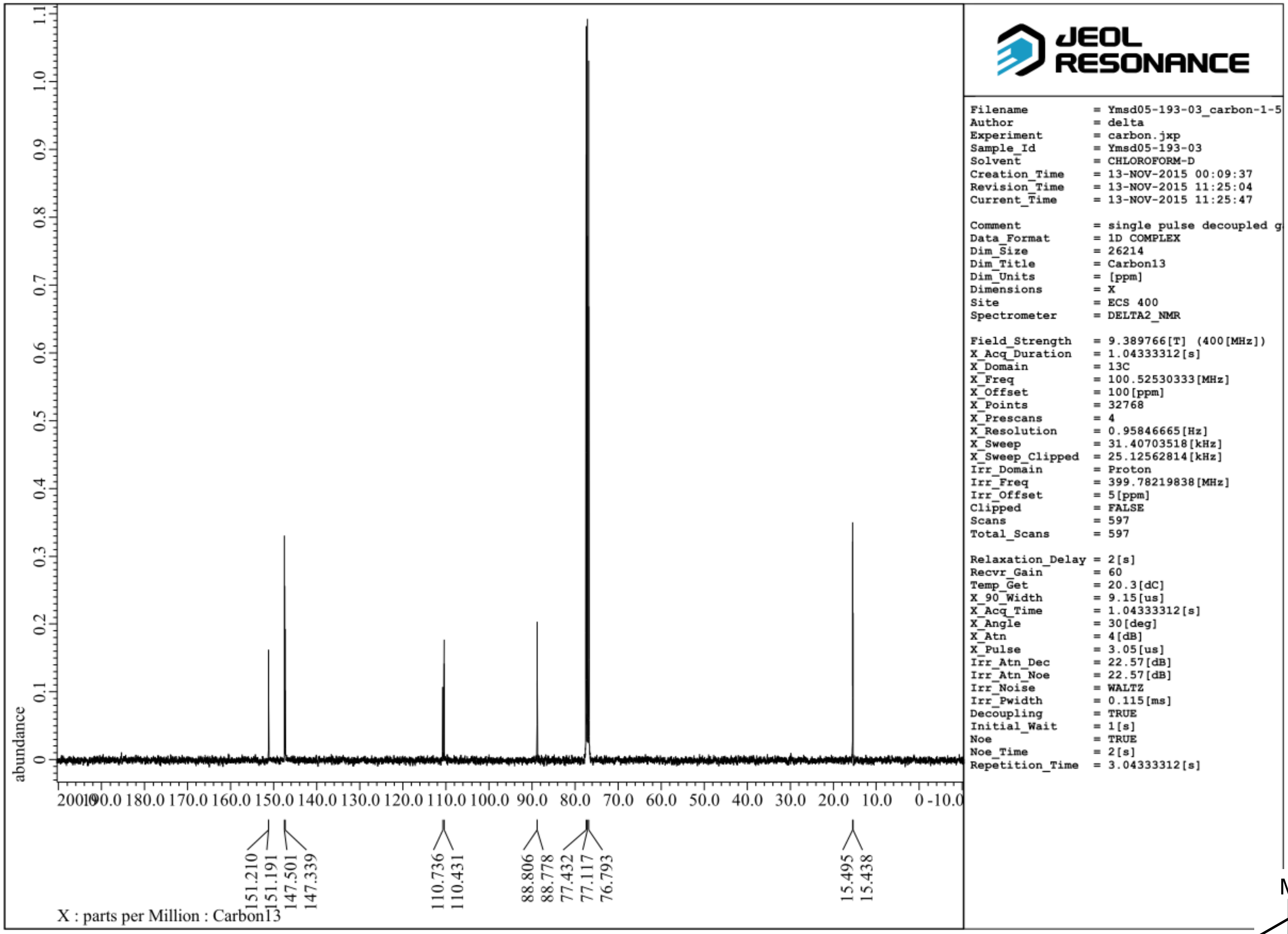
JEOL RESONANCE
Filename = Ymsd05-193-03_carbon-1-5
Author = delta
Experiment = carbon.jxp
Sample_Id = Ymsd05-193-03
Solvent = CHLOROFORM-D
Creation_Time = 13-NOV-2015 00:09:37
Revision_Time = 13-NOV-2015 11:25:04
Current_Time = 13-NOV-2015 11:25:47
Comment = single pulse decoupled g
Data_Format = 1D COMPLEX
Dim_Size = 26214
Dim_Title = Carbon13
Dim_Units = [ppm]
Dimensions = X
Site = ECS 400
Spectrometer = DELTA2_NMR
Field_Strength = 9.389766[T] (400[MHz])
X_Acq_Duration = 1.04333312[s]
X_Domain = 13C
X_Freq = 100.52530333[MHz]
X_Offset = 100[ppm]
X_Points = 32768
X_Prescans = 4
X_Resolution = 0.95846665[Hz]
X_Sweep = 31.40703518[kHz]
X_Sweep_Clipped = 25.12562814[kHz]
Irr_Domain = Proton
Irr_Freq = 399.78219838[MHz]
Irr_Offset = 5[ppm]
Clipped = FALSE
Scans = 597
Total_Scans = 597
Relaxation_Delay = 2[s]
Recvr_Gain = 60
Temp_Get = 20.3[dC]
X_90_Width = 9.15[us]
X_Acq_Time = 1.04333312[s]
X_Angle = 30[deg]
X_Atn = 4[dB]
X_Pulse = 3.05[us]
Irr_Atn_Dec = 22.57[dB]
Irr_Atn_Noe = 22.57[dB]
Irr_Noise = WALTZ
Irr_Pwidth = 0.115[ms]
Decoupling = TRUE
Initial_Wait = 1[s]
Noe = TRUE
Noe_Time = 2[s]
Repetition_Time = 3.04333312[s]
abundance
0 0.1 0.2 0.3 0.4 0.5 0.6 0.7 0.8 0.9 1.0 1.1
200190.0 180.0 170.0 160.0 150.0 140.0 130.0 120.0 110.0 100.0 90.0 80.0 70.0 60.0 50.0 40.0 30.0 20.0 10.0 0 -10.0
151.210
151.191
147.501
147.339
110.736
110.431
88.806
88.778
77.432
77.117
76.793
15.495
15.438
X : parts per Million : Carbon13

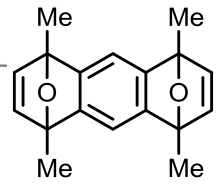
Me
Me
O
O
Me
Me

S2

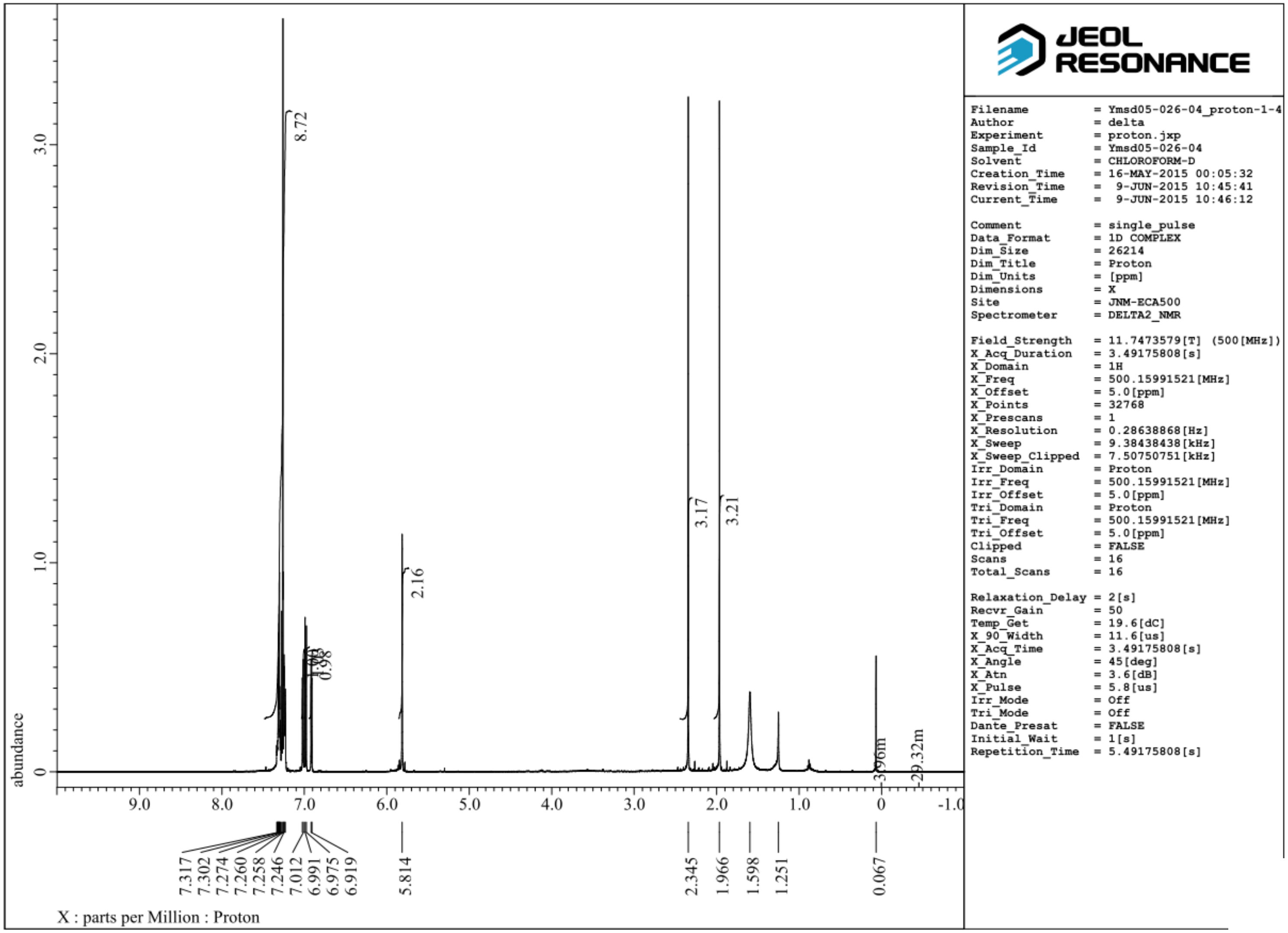

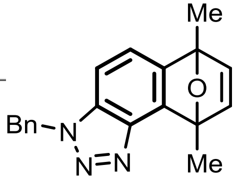

distal-**3b**

You created this PDF from an application that is not licensed to print to novaPDF printer (http://www.novapdf.com)

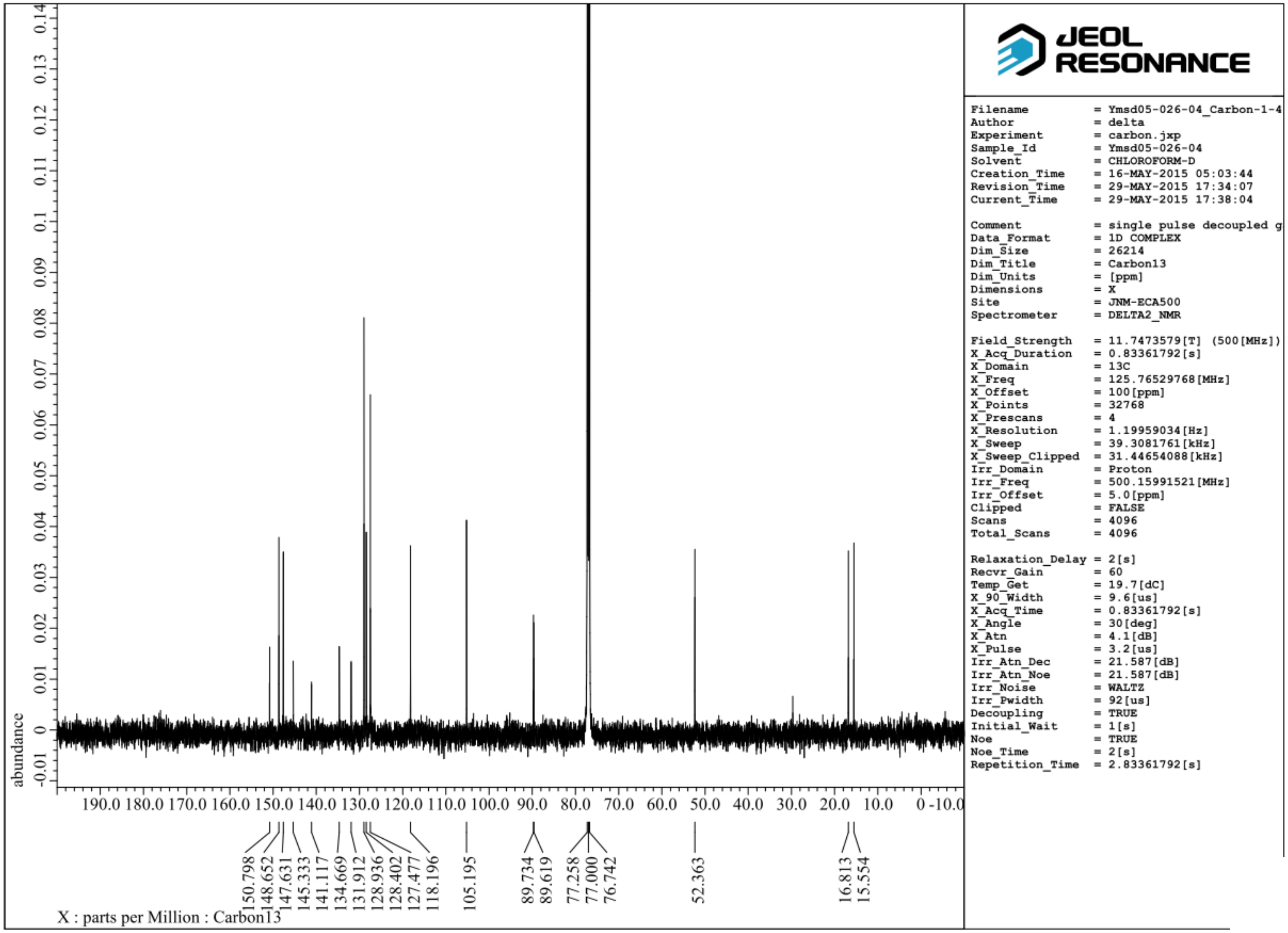

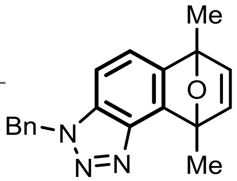

distal-**3b**

You created this PDF from an application that is not licensed to print to novaPDF printer (http://www.novapdf.com)

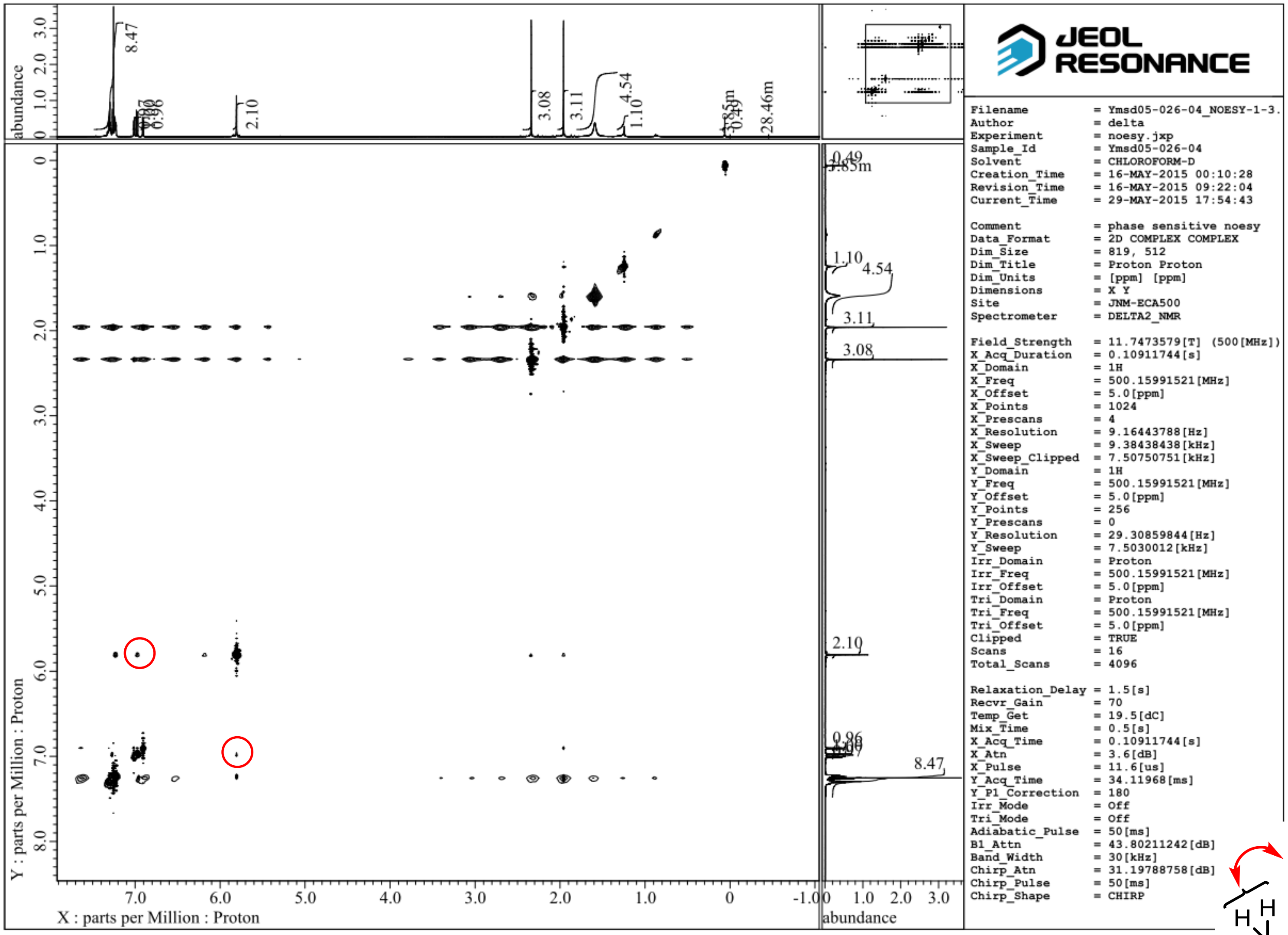

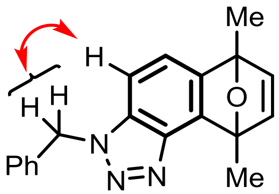

distal-**3b**

You created this PDF from an application that is not licensed to print to novaPDF printer (http://www.novapdf.com)

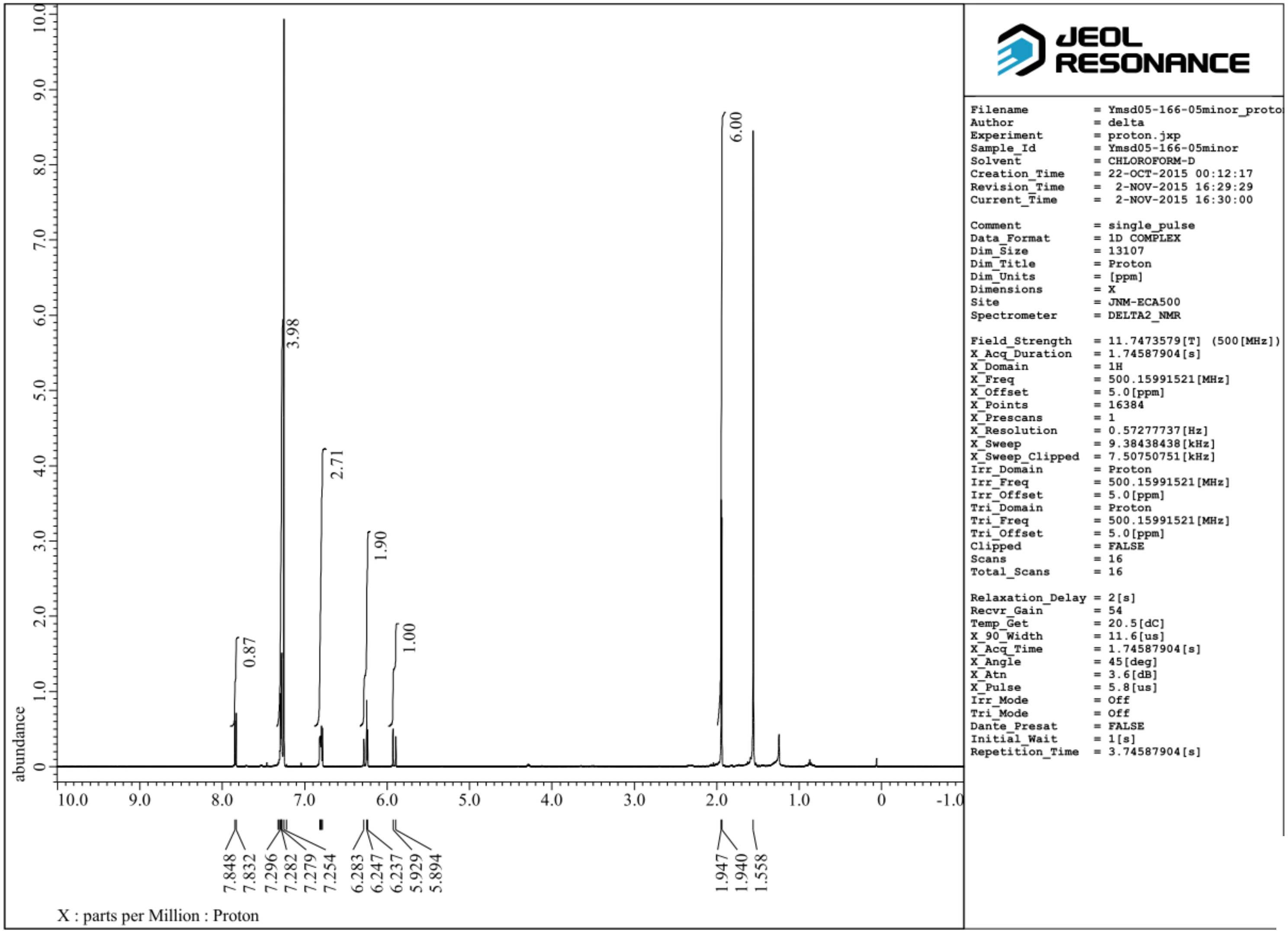

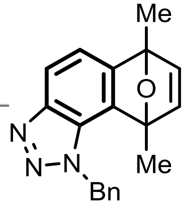

proximal-**3b**

You created this PDF from an application that is not licensed to print to novaPDF printer (http://www.novapdf.com)

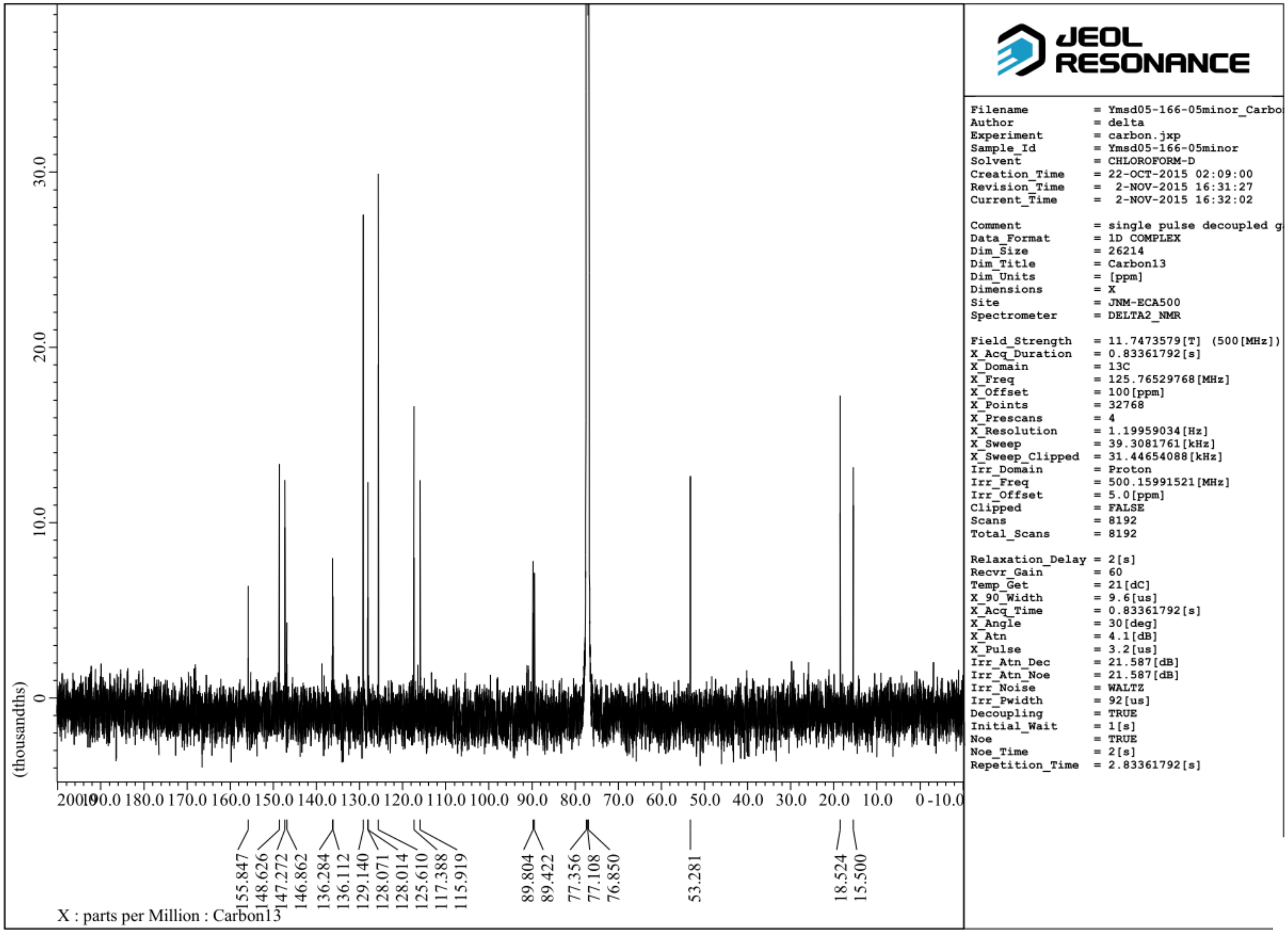

You created this PDF from an application that is not licensed to print to novaPDF printer (http://www.novapdf.com)

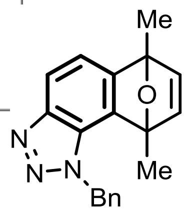

proximal-**3b**

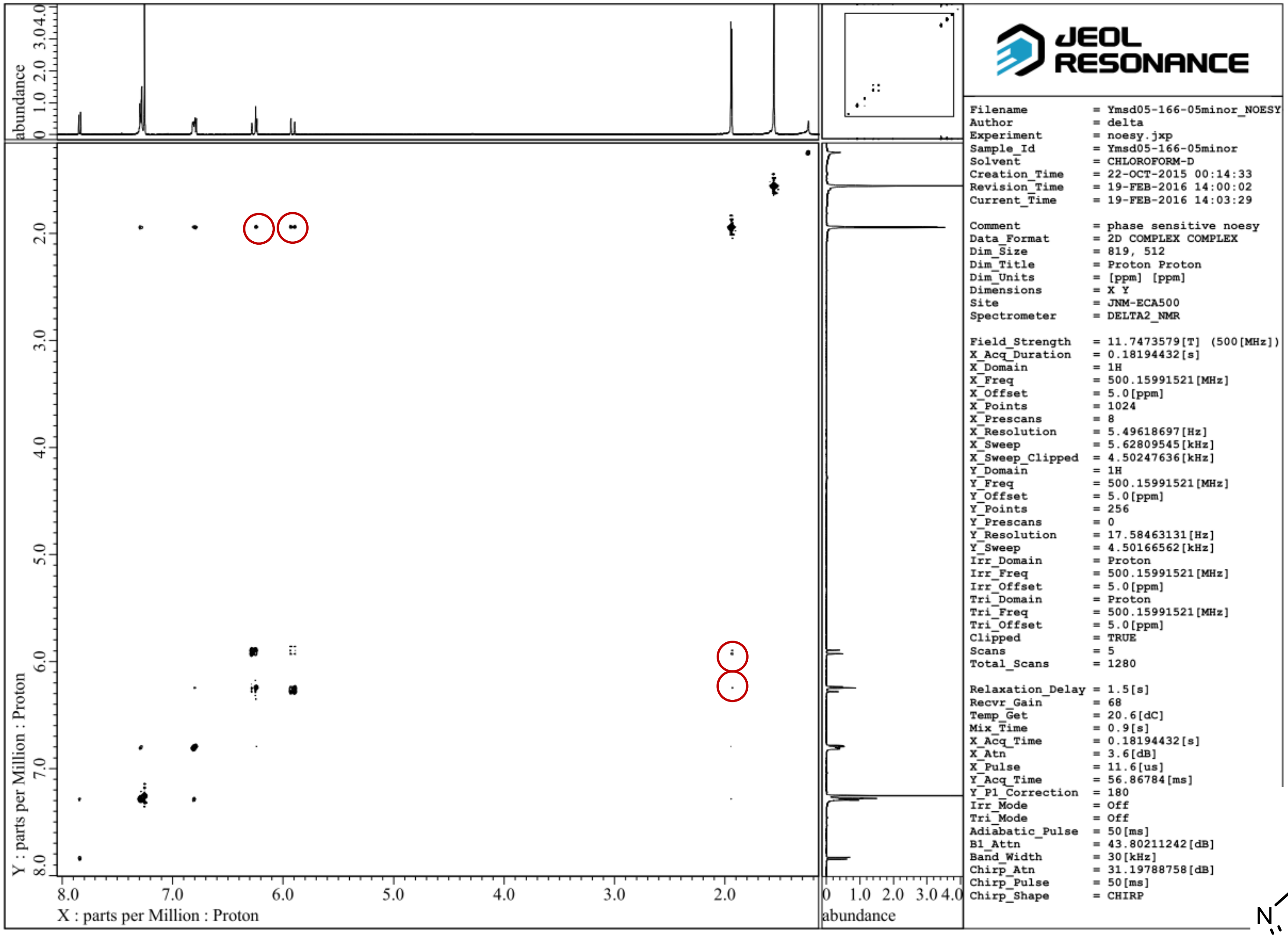

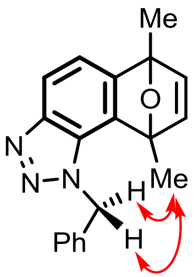

proximal-**3b**

You created this PDF from an application that is not licensed to print to novaPDF printer (http://www.novapdf.com)

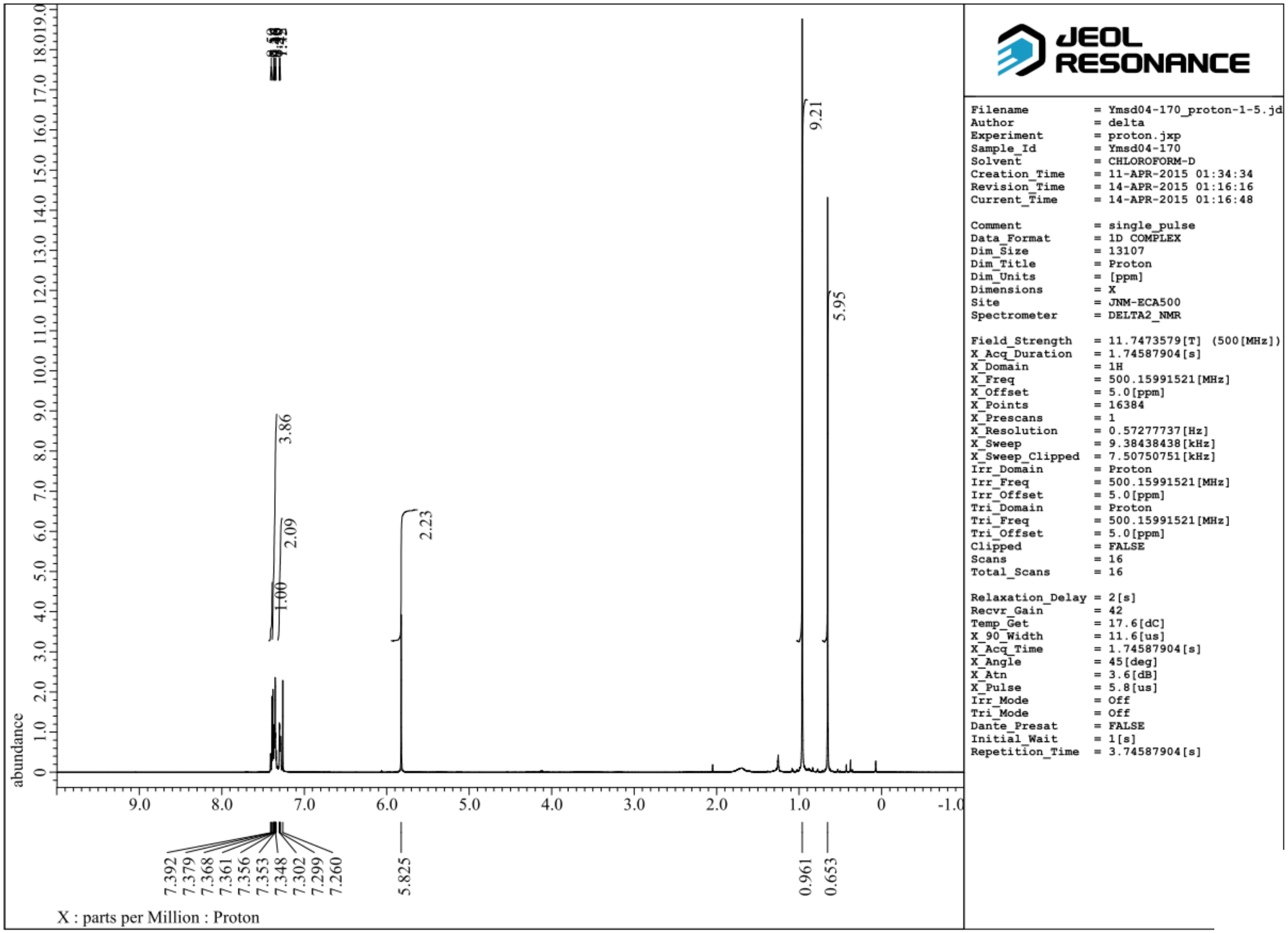

You created this PDF from an application that is not licensed to print to novaPDF printer (http://www.novapdf.com)

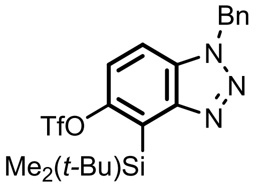

distal-**10b**

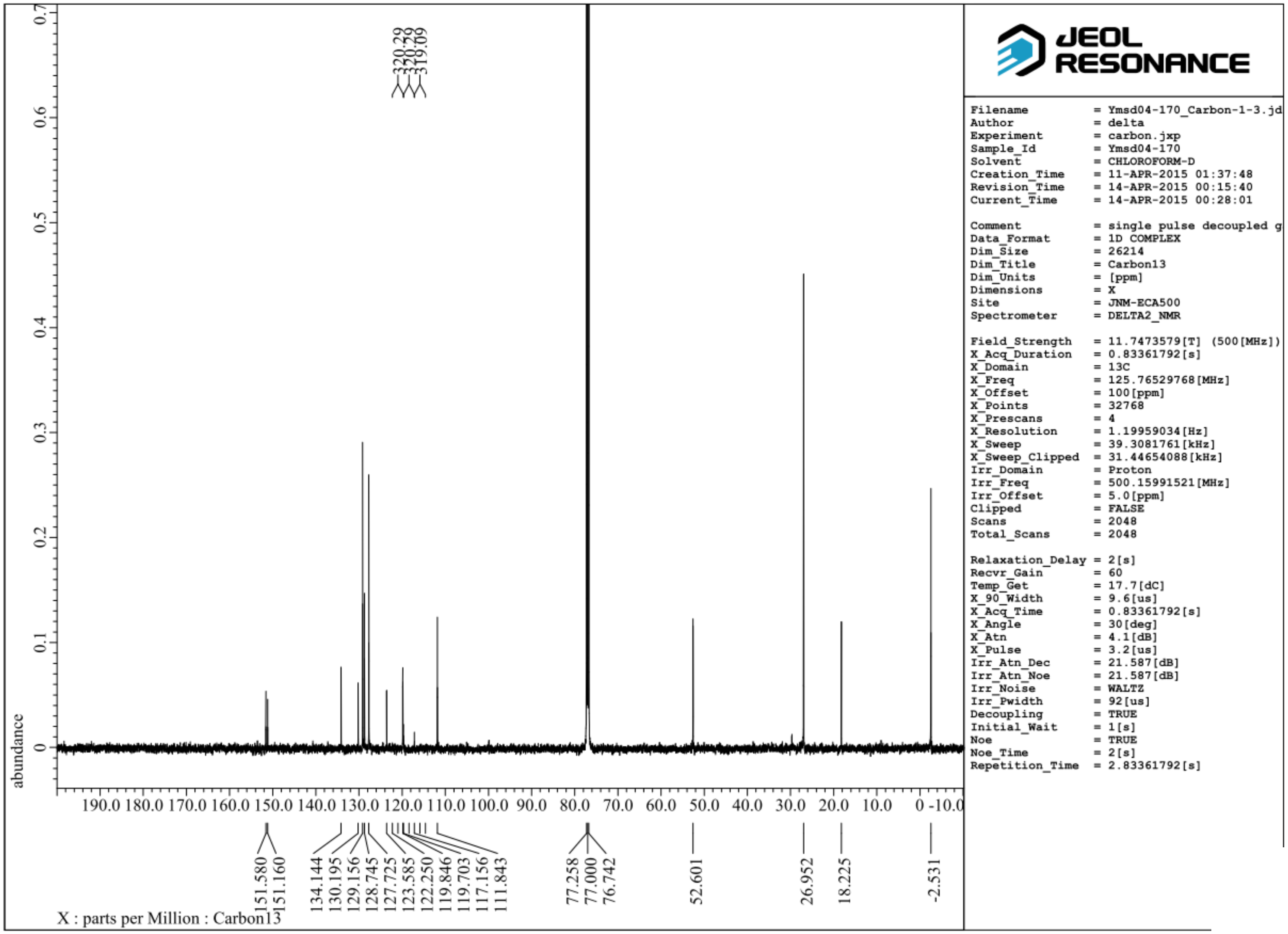

You created this PDF from an application that is not licensed to print to novaPDF printer (http://www.novapdf.com)

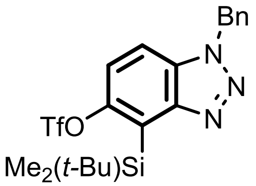

distal-**10b**

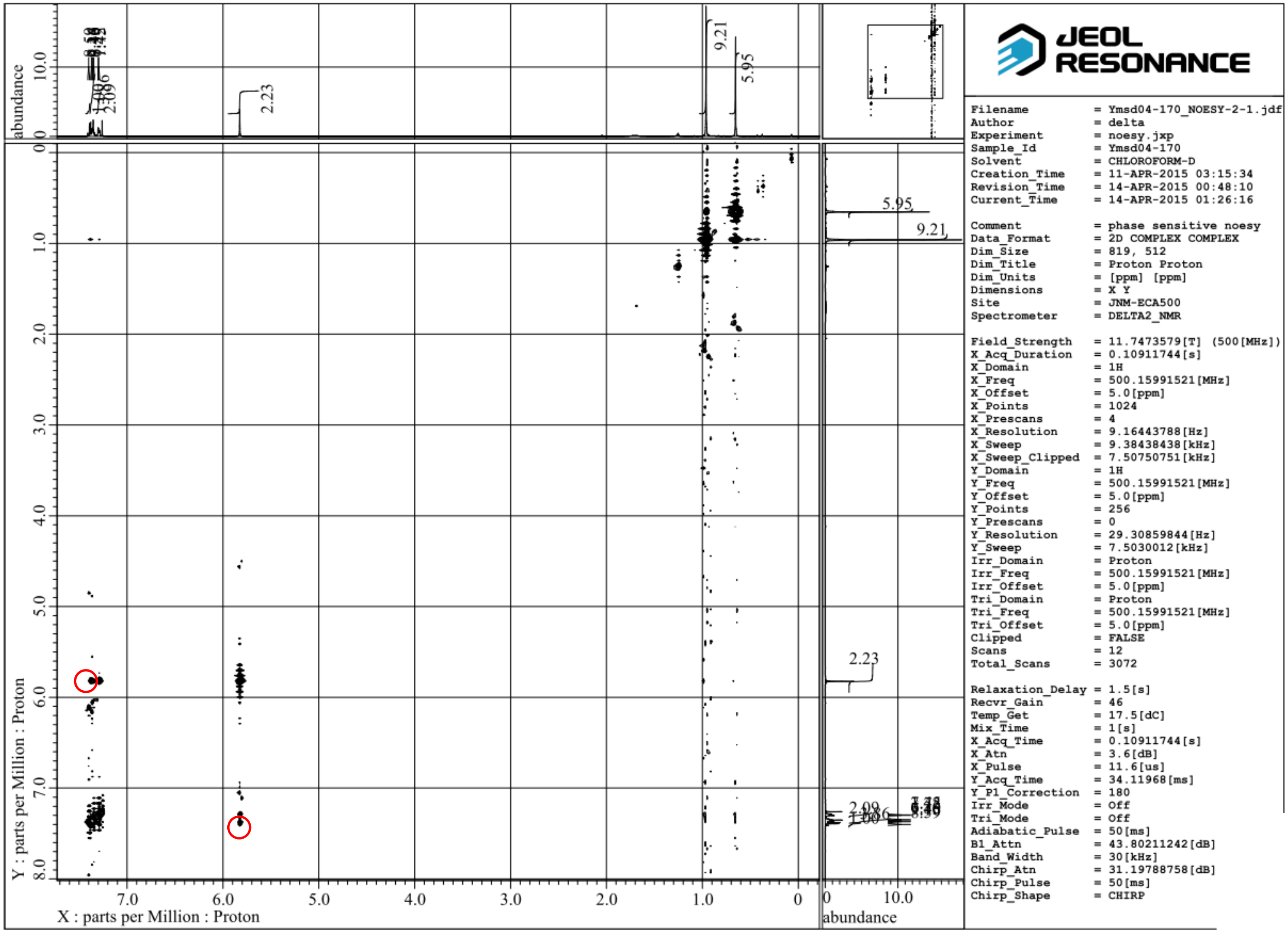

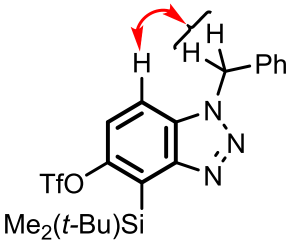

distal-**10b**

You created this PDF from an application that is not licensed to print to novaPDF printer (http://www.novapdf.com)

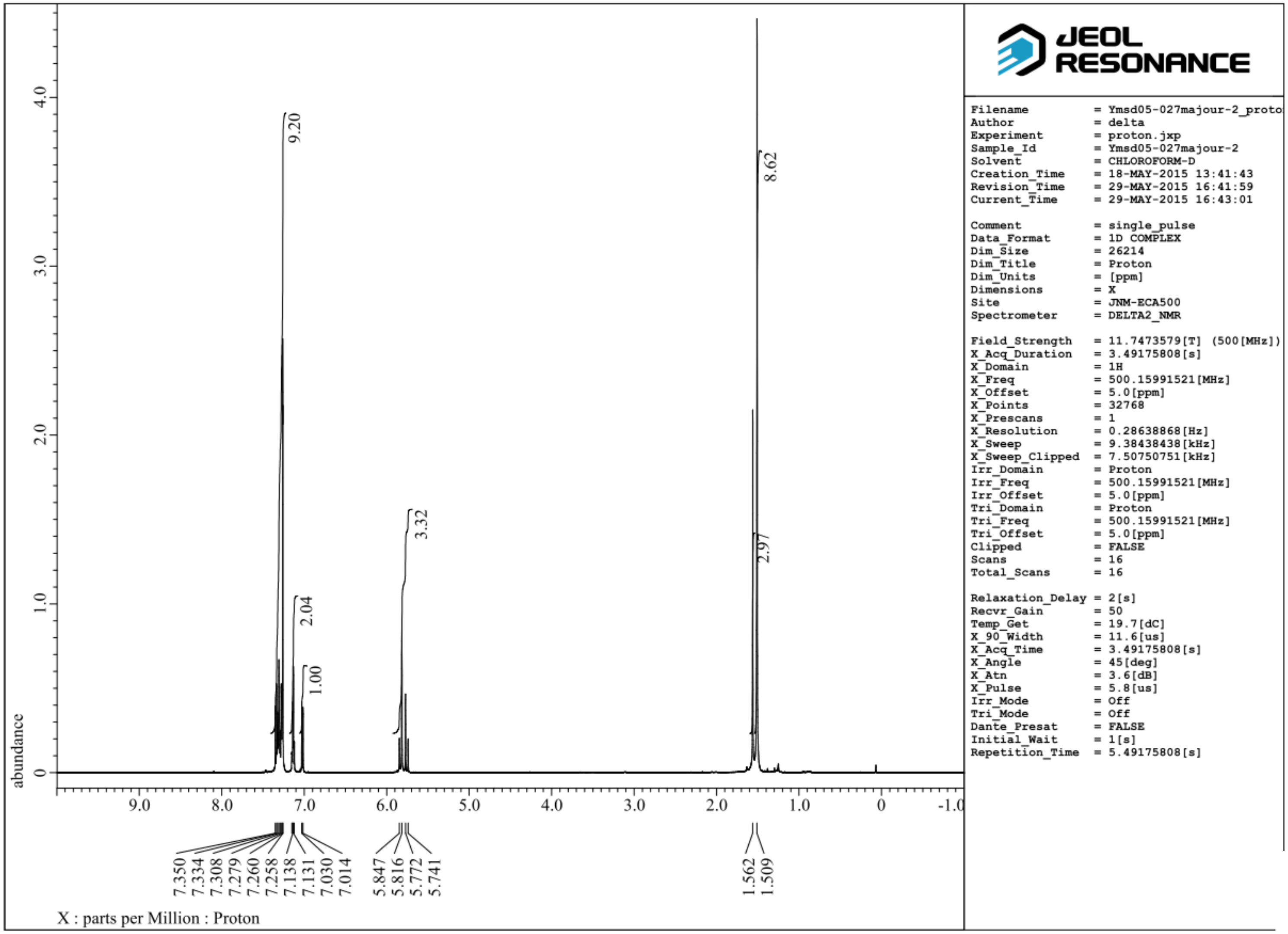

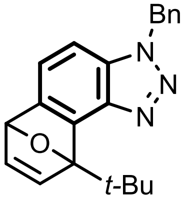

proximal-**3c**

You created this PDF from an application that is not licensed to print to novaPDF printer (http://www.novapdf.com)

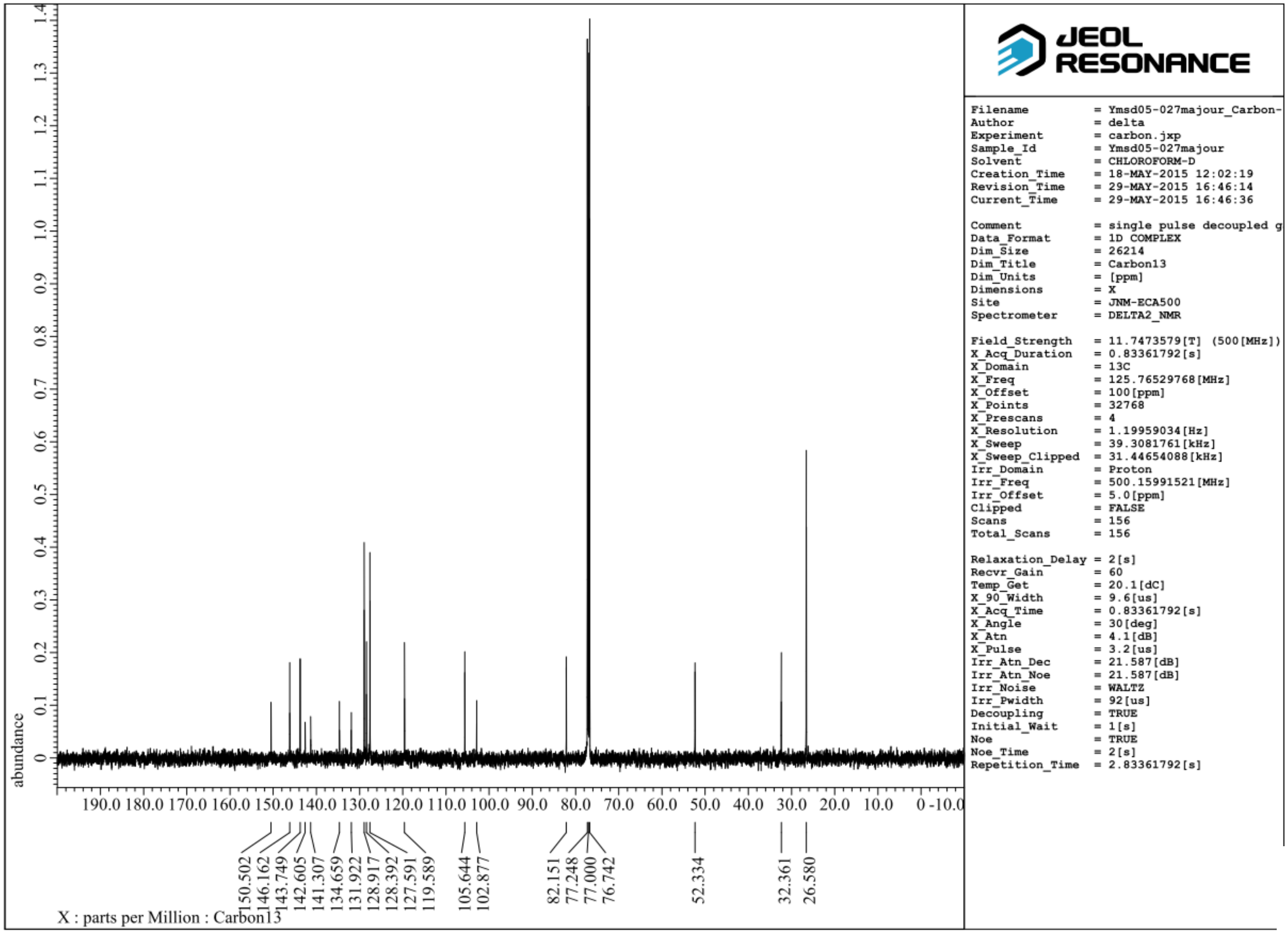

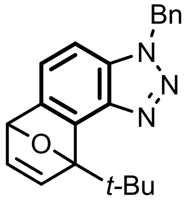

proximal-**3c**

You created this PDF from an application that is not licensed to print to novaPDF printer (http://www.novapdf.com)

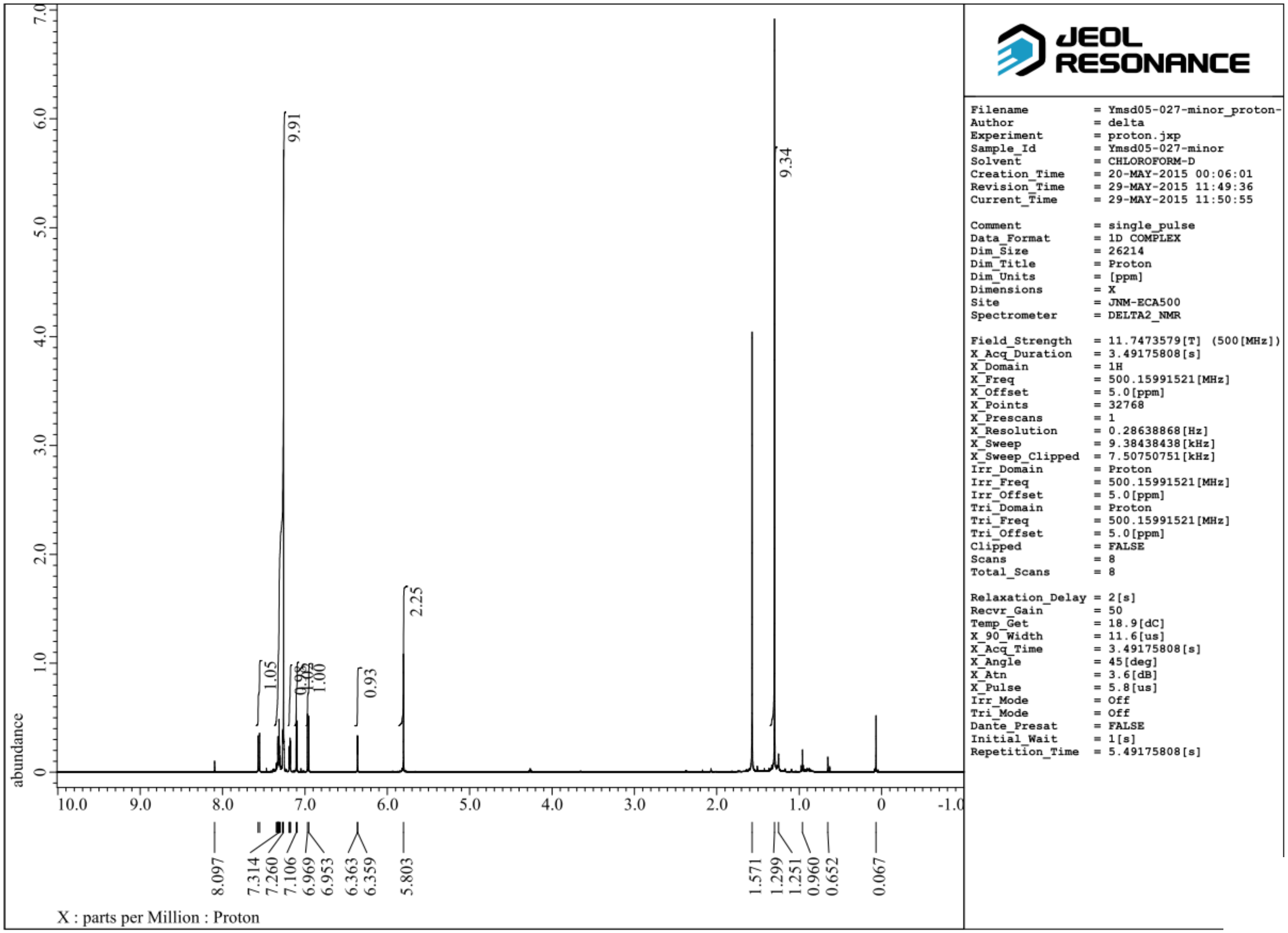

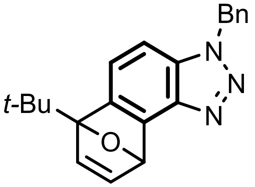

distal-**3c**

You created this PDF from an application that is not licensed to print to novaPDF printer (http://www.novapdf.com)

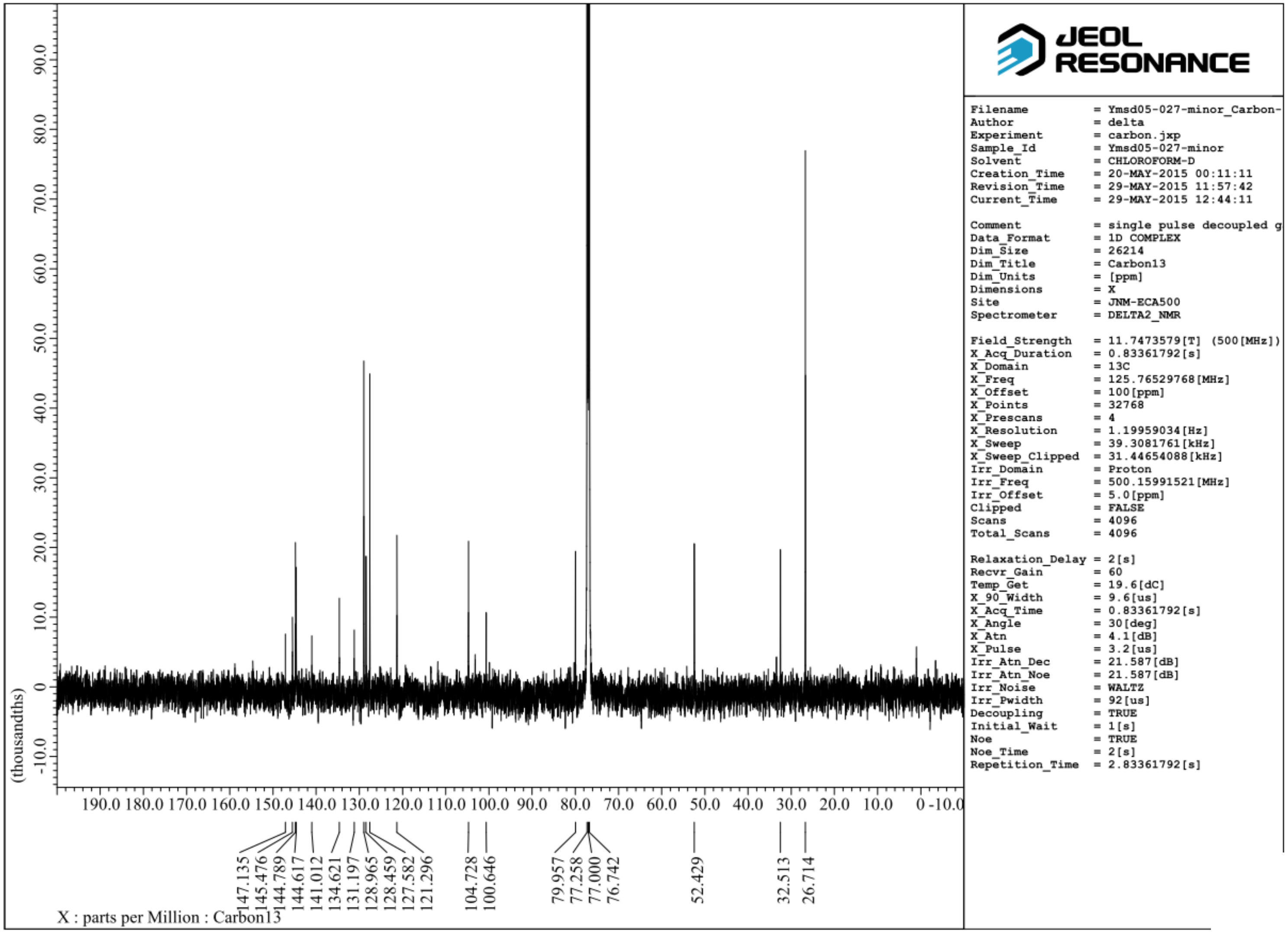

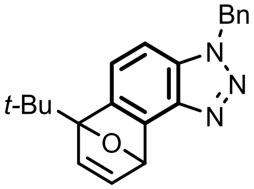

distal-**3c**

You created this PDF from an application that is not licensed to print to novaPDF printer (http://www.novapdf.com)

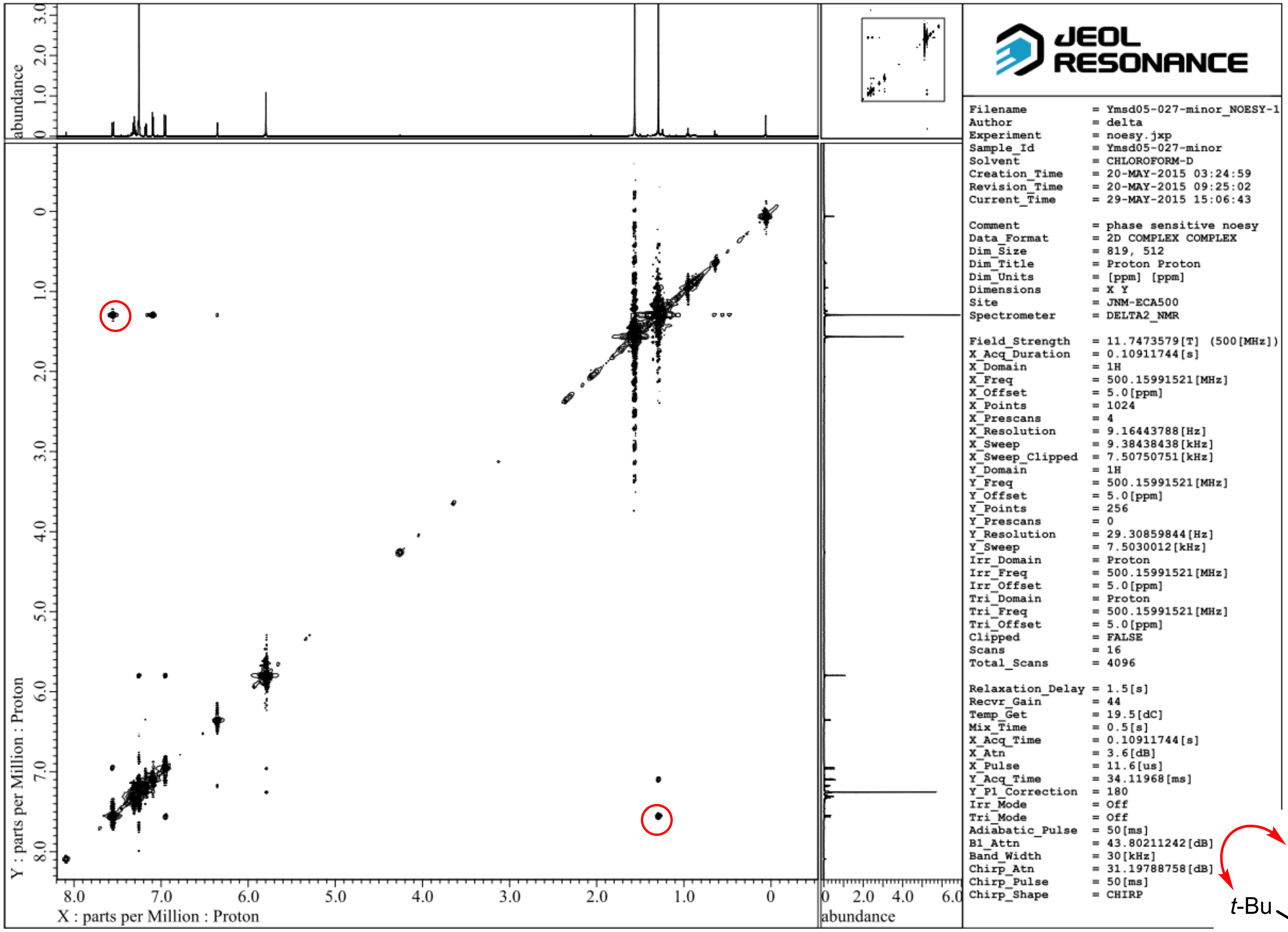

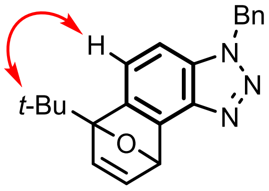

distal-**3c**

You created this PDF from an application that is not licensed to print to novaPDF printer (http://www.novapdf.com)

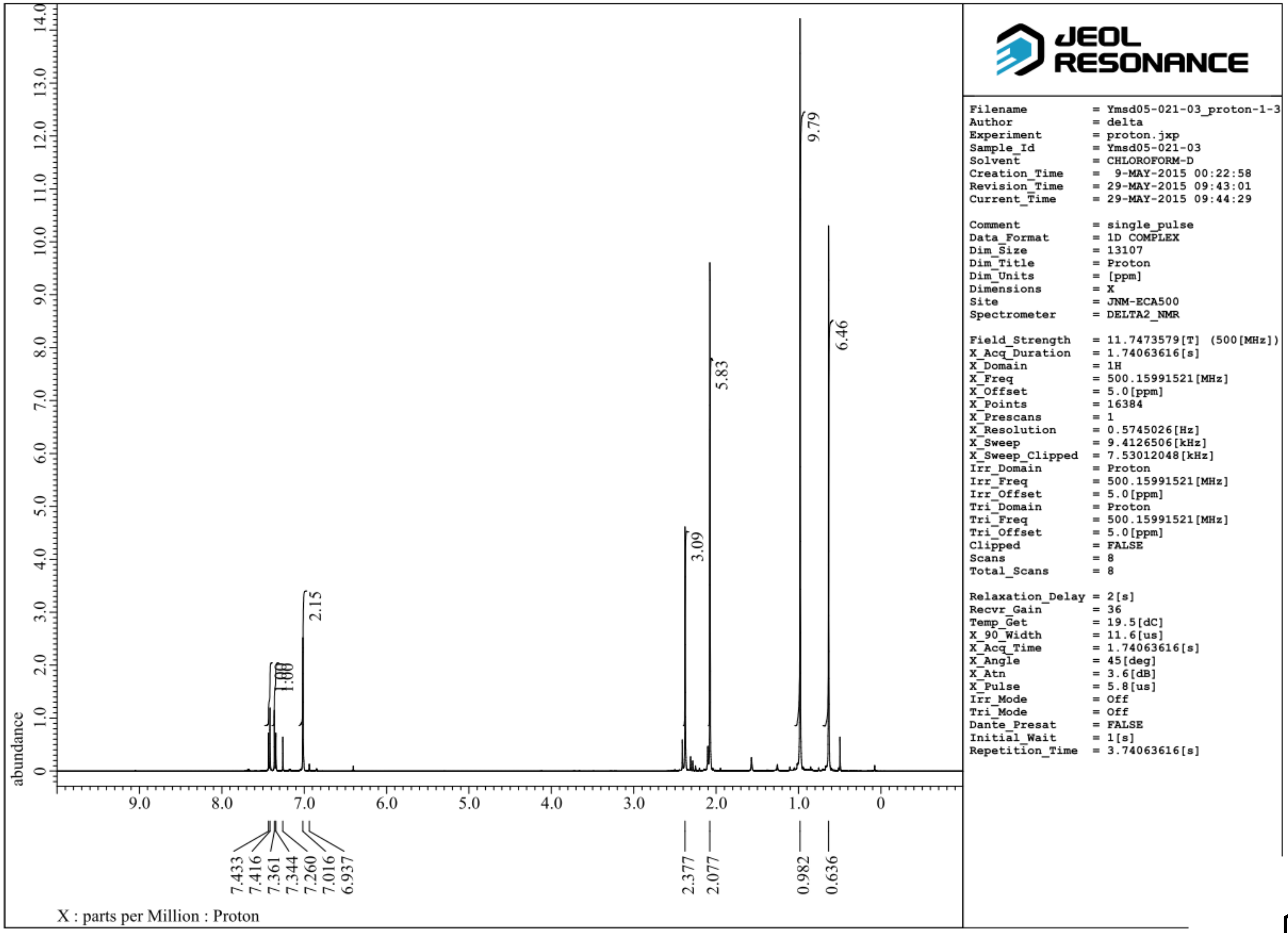
JEOL RESONANCE
Filename = Ymsd05-021-03_proton-1-3
Author = delta
Experiment = proton.jxp
Sample_Id = Ymsd05-021-03
Solvent = CHLOROFORM-D
Creation_Time = 9-MAY-2015 00:22:58
Revision_Time = 29-MAY-2015 09:43:01
Current_Time = 29-MAY-2015 09:44:29
Comment = single_pulse
Data_Format = 1D COMPLEX
Dim_Size = 13107
Dim_Title = Proton
Dim_Units = [ppm]
Dimensions = X
Site = JNM-ECA500
Spectrometer = DELTA2_NMR
Field_Strength = 11.7473579[T] (500[MHz])
X_Acq_Duration = 1.74063616[s]
X_Domain = 1H
X_Freq = 500.15991521[MHz]
X_Offset = 5.0[ppm]
X_Points = 16384
X_Prescans = 1
X_Resolution = 0.5745026[Hz]
X_Sweep = 9.4126506[kHz]
X_Sweep_Clipped = 7.53012048[kHz]
Irr_Domain = Proton
Irr_Freq = 500.15991521[MHz]
Irr_Offset = 5.0[ppm]
Tri_Domain = Proton
Tri_Freq = 500.15991521[MHz]
Tri_Offset = 5.0[ppm]
Clipped = FALSE
Scans = 8
Total_Scans = 8
Relaxation_Delay = 2[s]
Recvr_Gain = 36
Temp_Get = 19.5[dC]
X_90_Width = 11.6[us]
X_Acq_Time = 1.74063616[s]
X_Angle = 45[deg]
X_Atn = 3.6[dB]
X_Pulse = 5.8[us]
Irr_Mode = Off
Tri_Mode = Off
Dante_Presat = FALSE
Initial_Wait = 1[s]
Repetition_Time = 3.74063616[s]
abundance
9.79
6.46
5.83
3.09
2.15
1.00
1.00
7.433
7.416
7.361
7.344
7.260
7.016
6.937
2.377
2.077
0.982
0.636
X : parts per Million : Proton

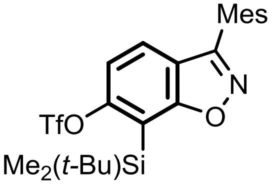
Mes
N
O
TfO
Me2(t-Bu)Si

distal-**10c**

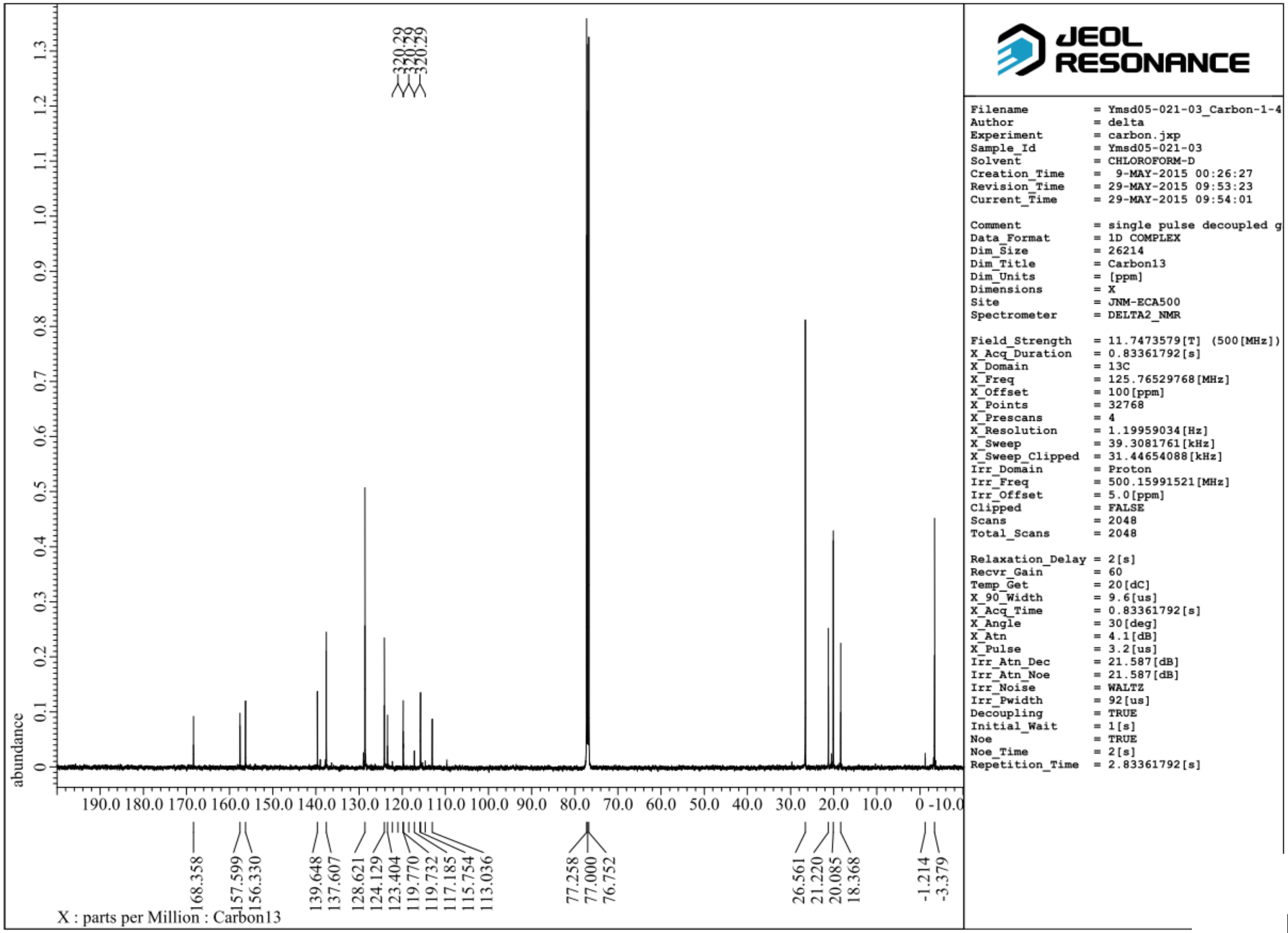
JEOL RESONANCE
Filename = Ymsd05-021-03_Carbon-1-4
Author = delta
Experiment = carbon.jxp
Sample_Id = Ymsd05-021-03
Solvent = CHLOROFORM-D
Creation_Time = 9-MAY-2015 00:26:27
Revision_Time = 29-MAY-2015 09:53:23
Current_Time = 29-MAY-2015 09:54:01
Comment = single pulse decoupled g
Data_Format = 1D COMPLEX
Dim_Size = 26214
Dim_Title = Carbon13
Dim_Units = [ppm]
Dimensions = X
Site = JNM-ECA500
Spectrometer = DELTA2_NMR
Field_Strength = 11.7473579[T] (500[MHz])
X_Acq_Duration = 0.83361792[s]
X_Domain = 13C
X_Freq = 125.76529768[MHz]
X_Offset = 100[ppm]
X_Points = 32768
X_Prescans = 4
X_Resolution = 1.19959034[Hz]
X_Sweep = 39.3081761[kHz]
X_Sweep_Clipped = 31.44654088[kHz]
Irr_Domain = Proton
Irr_Freq = 500.15991521[MHz]
Irr_Offset = 5.0[ppm]
Clipped = FALSE
Scans = 2048
Total_Scans = 2048
Relaxation_Delay = 2[s]
Recvr_Gain = 60
Temp_Get = 20[dC]
X_90_Width = 9.6[us]
X_Acq_Time = 0.83361792[s]
X_Angle = 30[deg]
X_Atn = 4.1[dB]
X_Pulse = 3.2[us]
Irr_Atn_Dec = 21.587[dB]
Irr_Atn_Noe = 21.587[dB]
Irr_Noise = WALTZ
Irr_Pwidth = 92[us]
Decoupling = TRUE
Initial_Wait = 1[s]
Noe = TRUE
Noe_Time = 2[s]
Repetition_Time = 2.83361792[s]
320.29
320.29
320.29
320.29
168.358
157.599
156.330
139.648
137.607
128.621
124.129
123.404
119.770
119.732
117.185
115.754
113.036
77.258
77.000
76.752
26.561
21.220
20.085
18.368
-1.214
-3.379
abundance
X : parts per Million : Carbon13

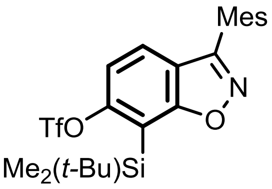
Mes
N
O
TfO
Me2(t-Bu)Si

distal-**10c**

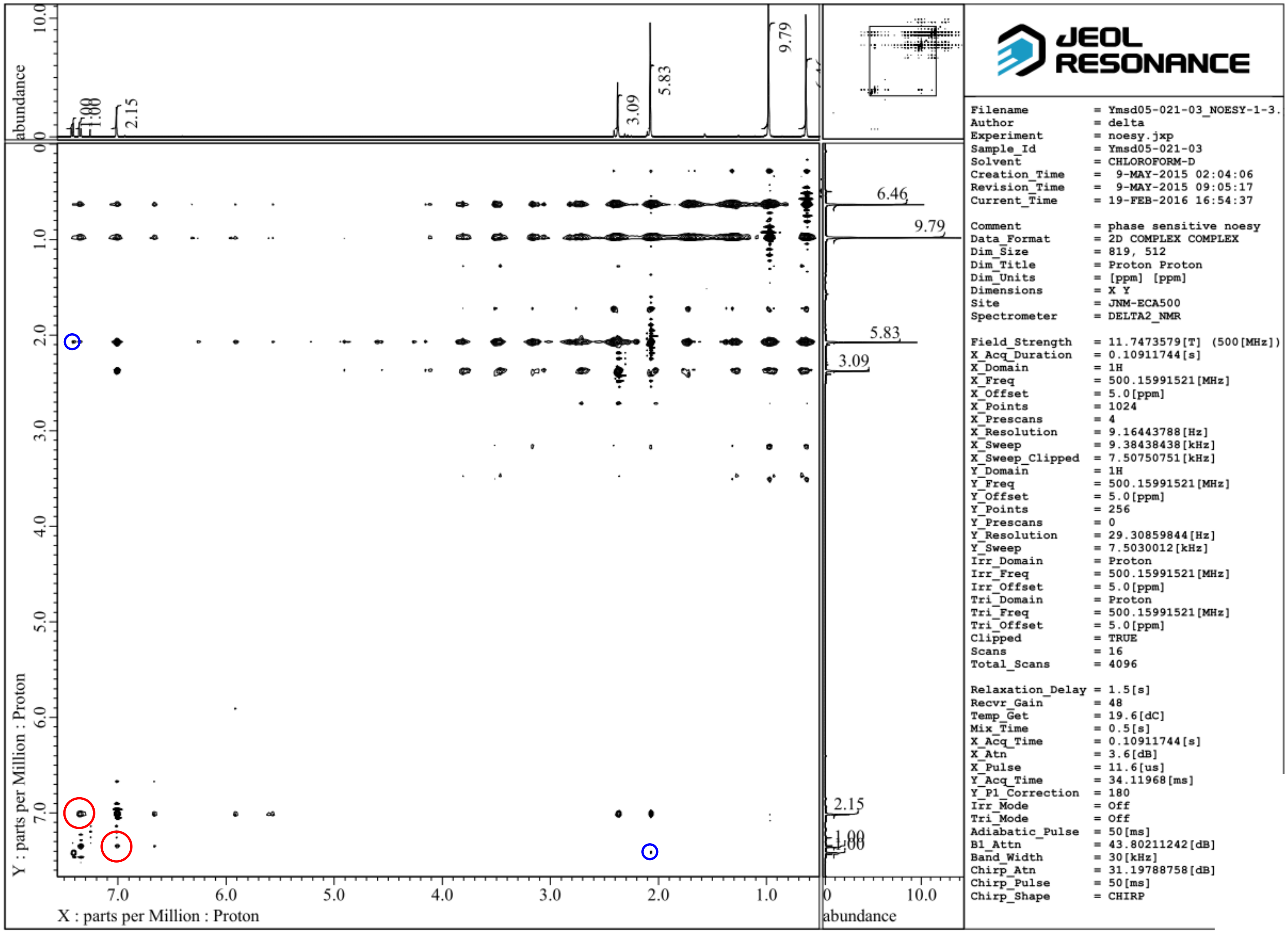

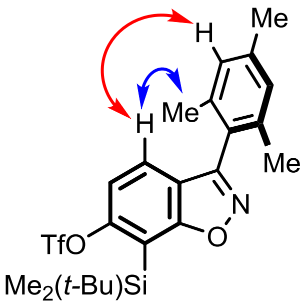

distal-**10c**

You created this PDF from an application that is not licensed to print to novaPDF printer (http://www.novapdf.com)

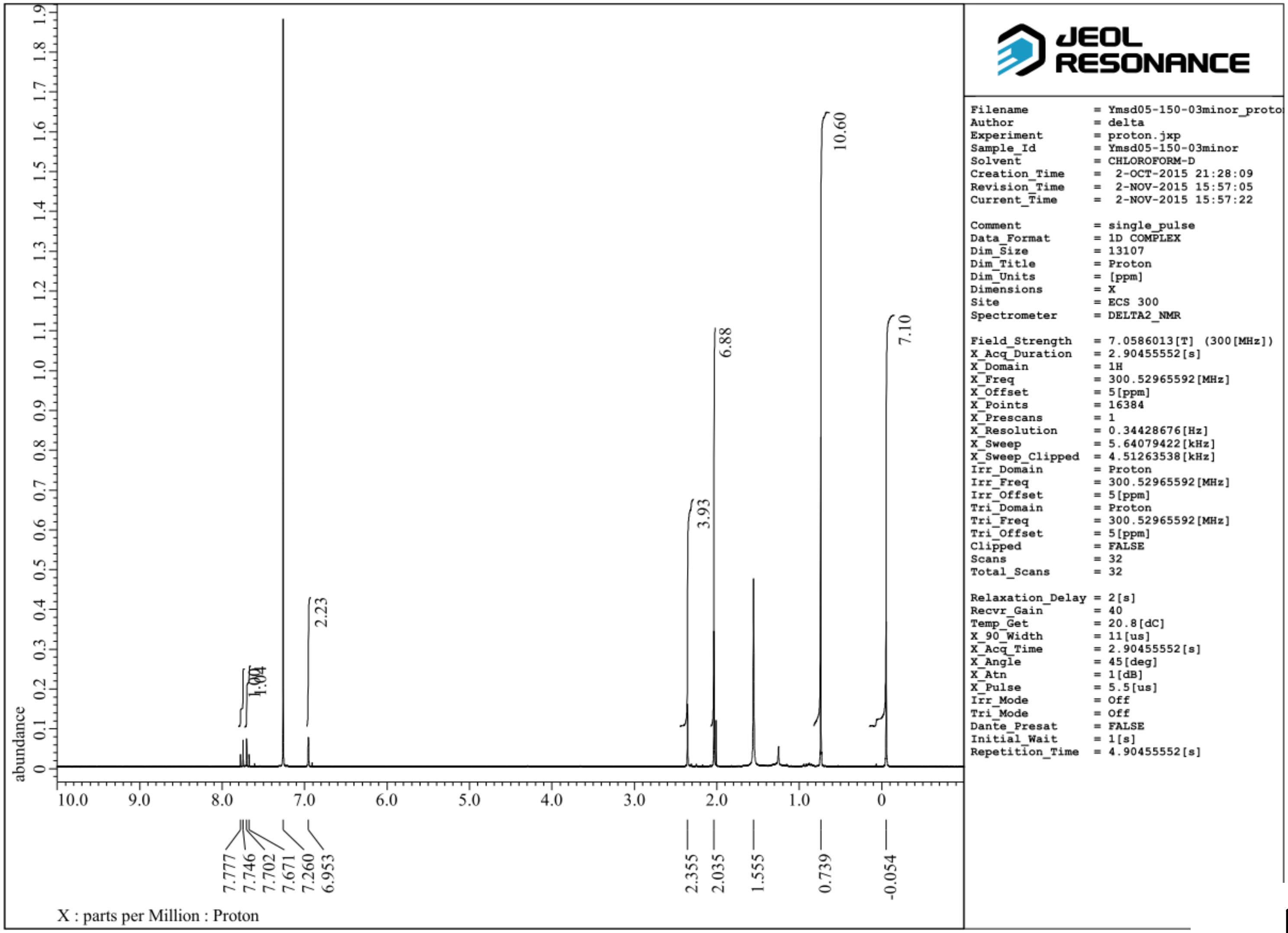

You created this PDF from an application that is not licensed to print to novaPDF printer (http://www.novapdf.com)

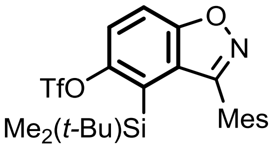

proximal-**10c**

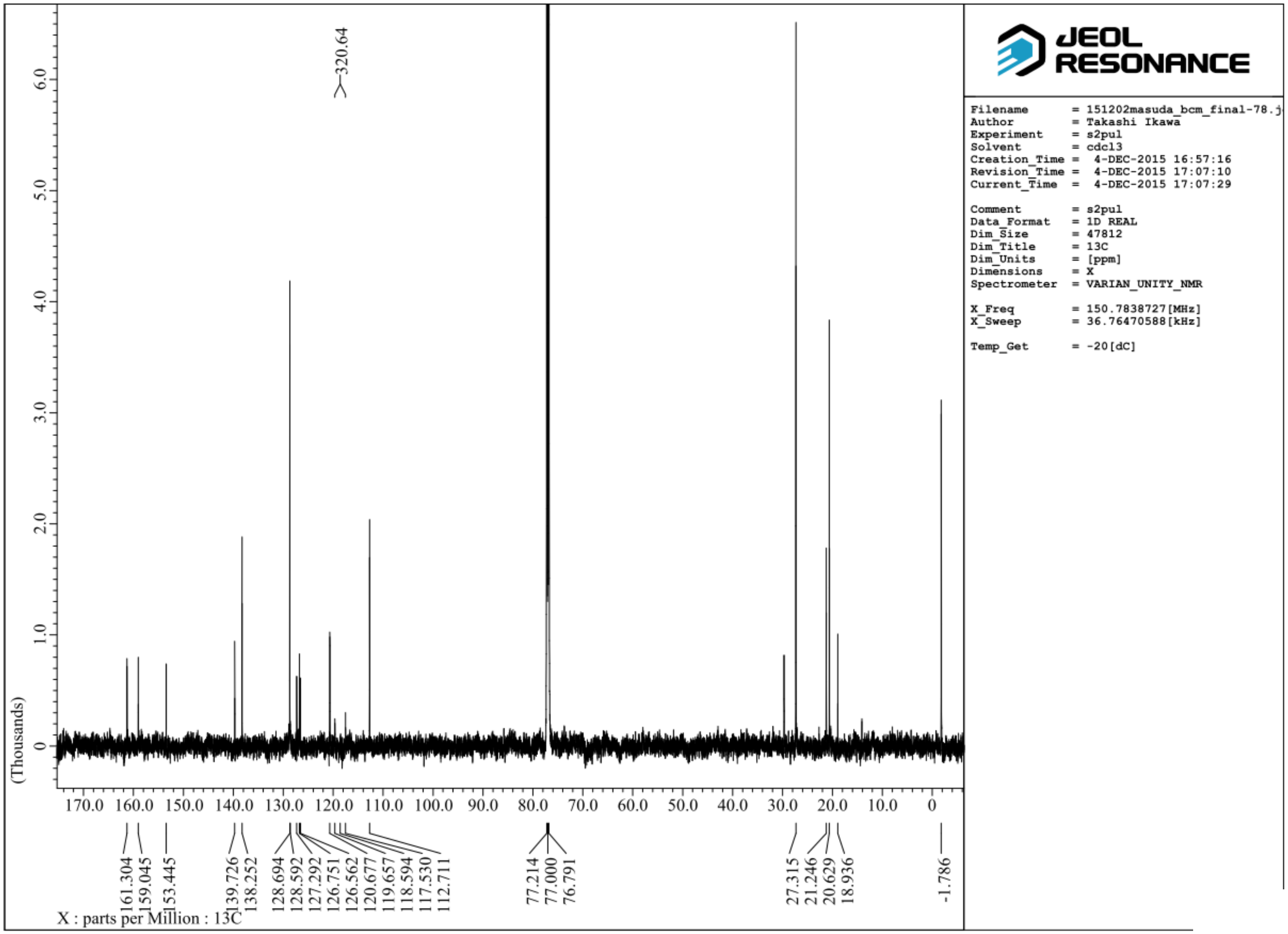

You created this PDF from an application that is not licensed to print to novaPDF printer (http://www.novapdf.com)

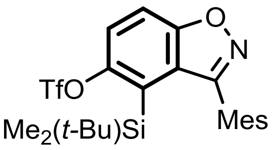

proximal-**10c**

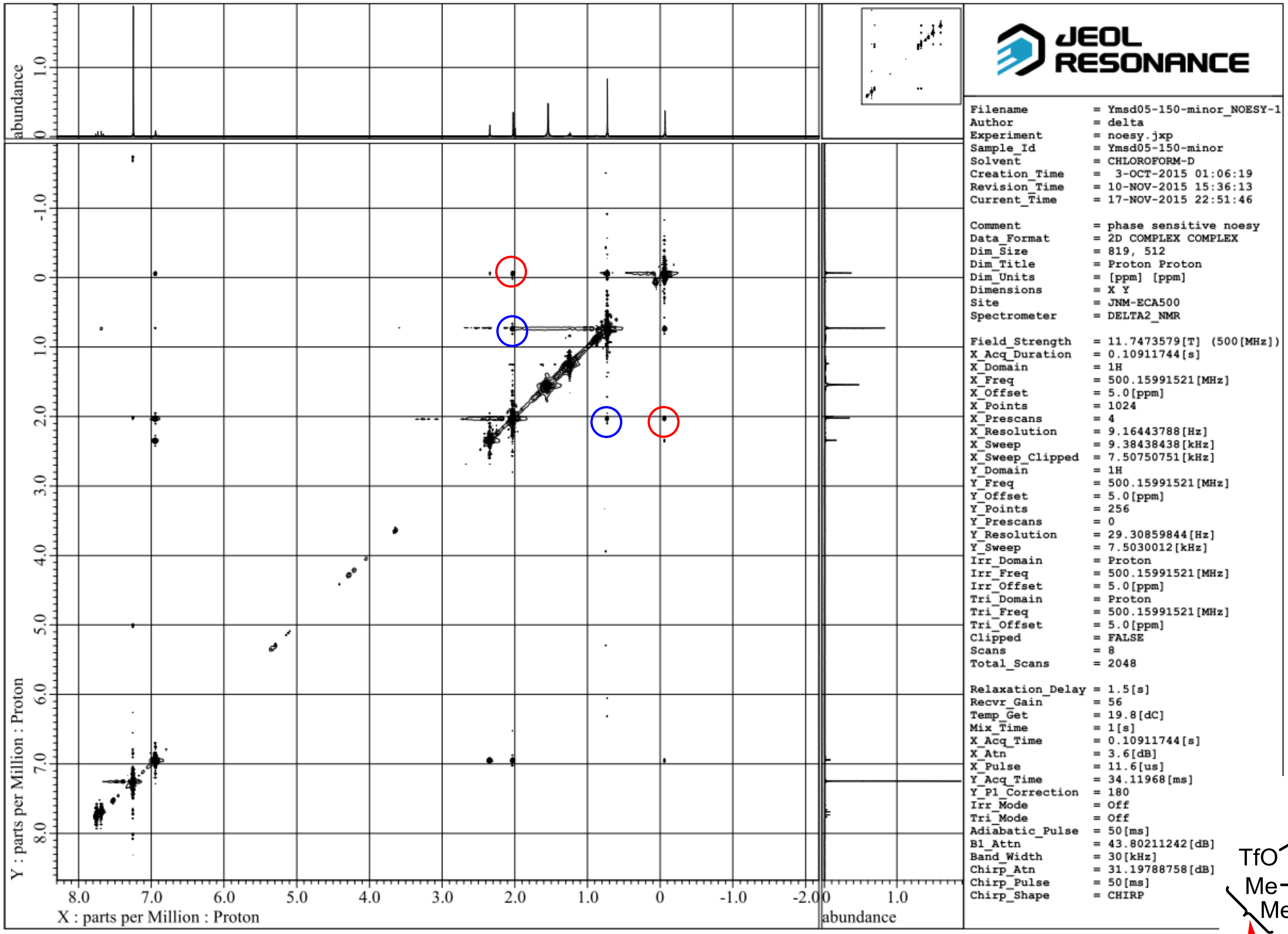

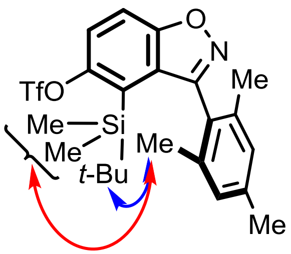

proximal-**10c**

You created this PDF from an application that is not licensed to print to novaPDF printer (http://www.novapdf.com)

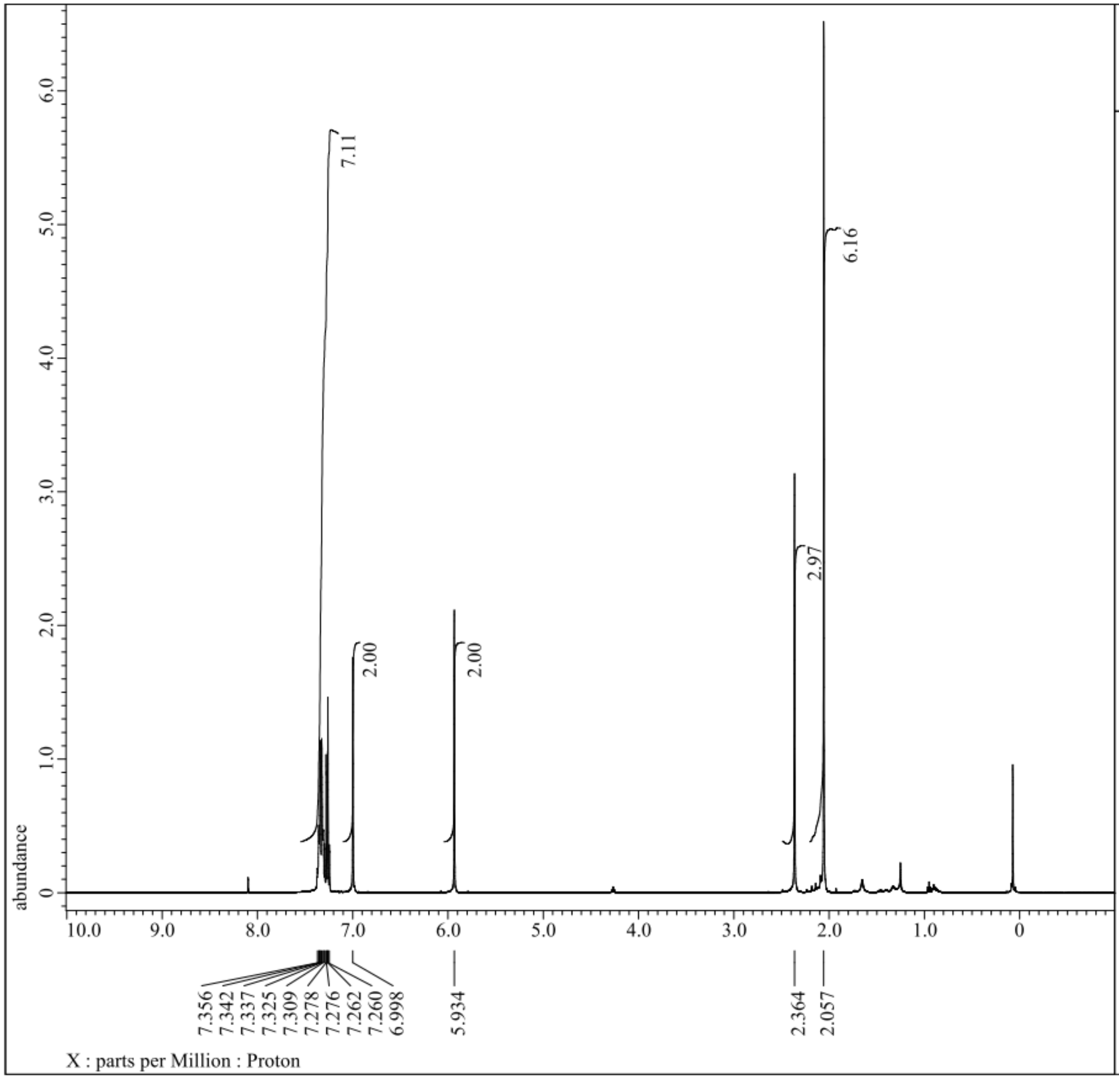

JEOL RESONANCE

```
Filename             = Ymsd05-028-02_proton-1-3
Author               = delta
Experiment           = proton.jxp
Sample_Id            = Ymsd05-028-02
Solvent              = CHLOROFORM-D
Creation_Time        = 20-MAY-2015 11:53:29
Revision_Time        =  5-AUG-2015 10:19:13
Current_Time         =  5-AUG-2015 10:19:33

Comment              = single_pulse
Data_Format          = 1D COMPLEX
Dim_Size             = 26214
Dim_Title            = Proton
Dim_Units            = [ppm]
Dimensions           = X
Site                 = JNM-ECA500
Spectrometer         = DELTA2_NMR

Field_Strength       = 11.7473579[T] (500[MHz])
X_Acq_Duration       = 3.49175808[s]
X_Domain             = 1H
X_Freq               = 500.15991521[MHz]
X_Offset             = 5.0[ppm]
X_Points             = 32768
X_Prescans           = 1
X_Resolution         = 0.28638868[Hz]
X_Sweep              = 9.38438438[kHz]
X_Sweep_Clipped      = 7.50750751[kHz]
Irr_Domain           = Proton
Irr_Freq             = 500.15991521[MHz]
Irr_Offset           = 5.0[ppm]
Tri_Domain           = Proton
Tri_Freq             = 500.15991521[MHz]
Tri_Offset           = 5.0[ppm]
Clipped              = FALSE
Scans                = 8
Total_Scans          = 8

Relaxation_Delay     = 2[s]
Recvr_Gain           = 40
Temp_Get             = 19.4[dC]
X_90_Width           = 11.6[us]
X_Acq_Time           = 3.49175808[s]
X_Angle              = 45[deg]
X_Atn                = 3.6[dB]
X_Pulse              = 5.8[us]
Irr_Mode             = Off
Tri_Mode             = Off
Dante_Presat         = FALSE
Initial_Wait         = 1[s]
Repetition_Time      = 5.49175808[s]
```

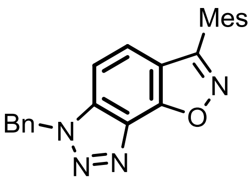

distal-**3d**

You created this PDF from an application that is not licensed to print to novaPDF printer (http://www.novapdf.com)

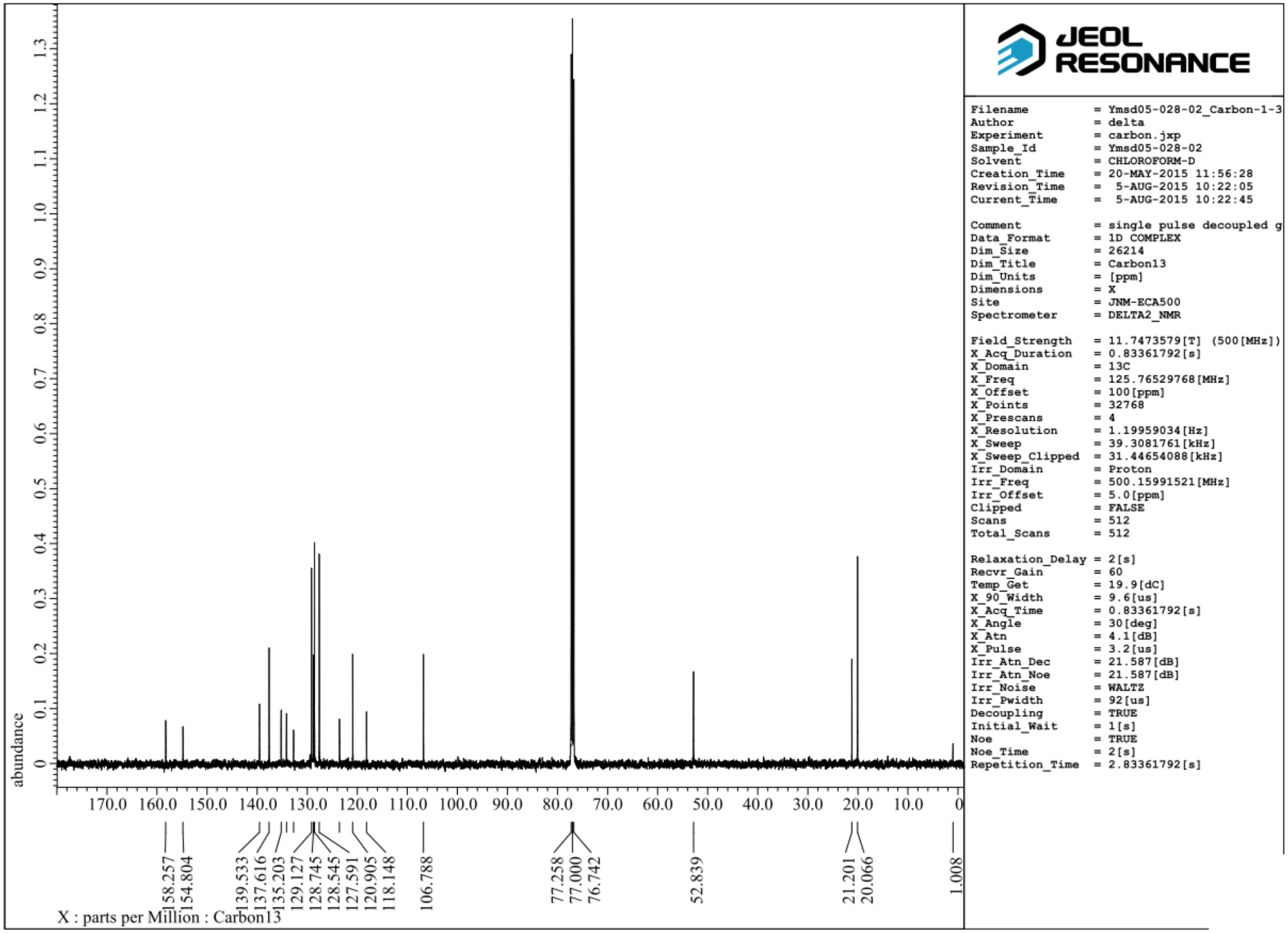

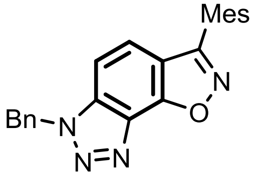

distal-**3d**

You created this PDF from an application that is not licensed to print to novaPDF printer (http://www.novapdf.com)

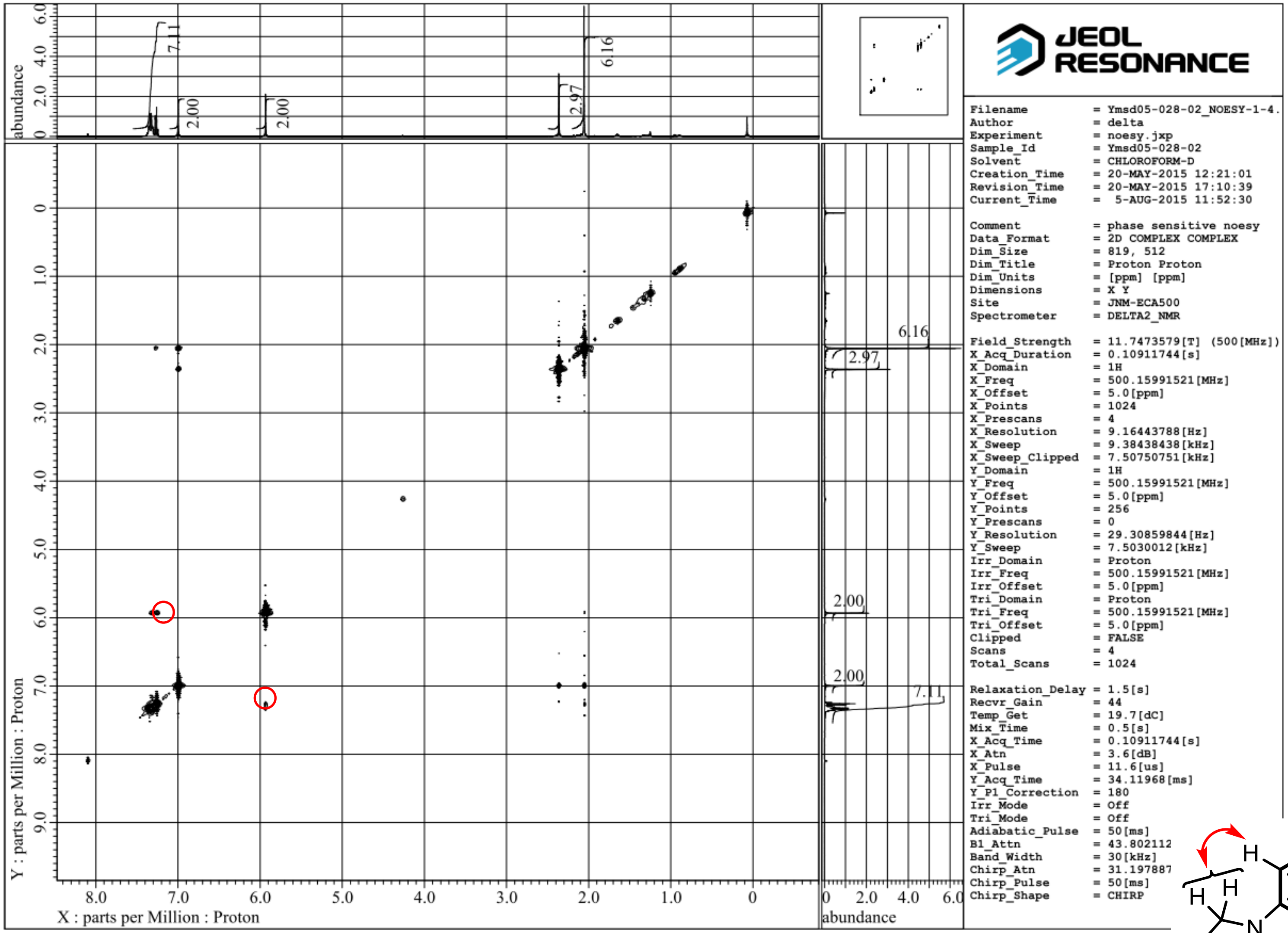

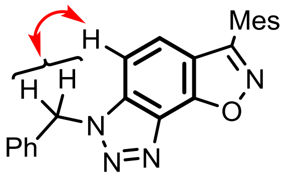

distal-**3d**

You created this PDF from an application that is not licensed to print to novaPDF printer (http://www.novapdf.com)

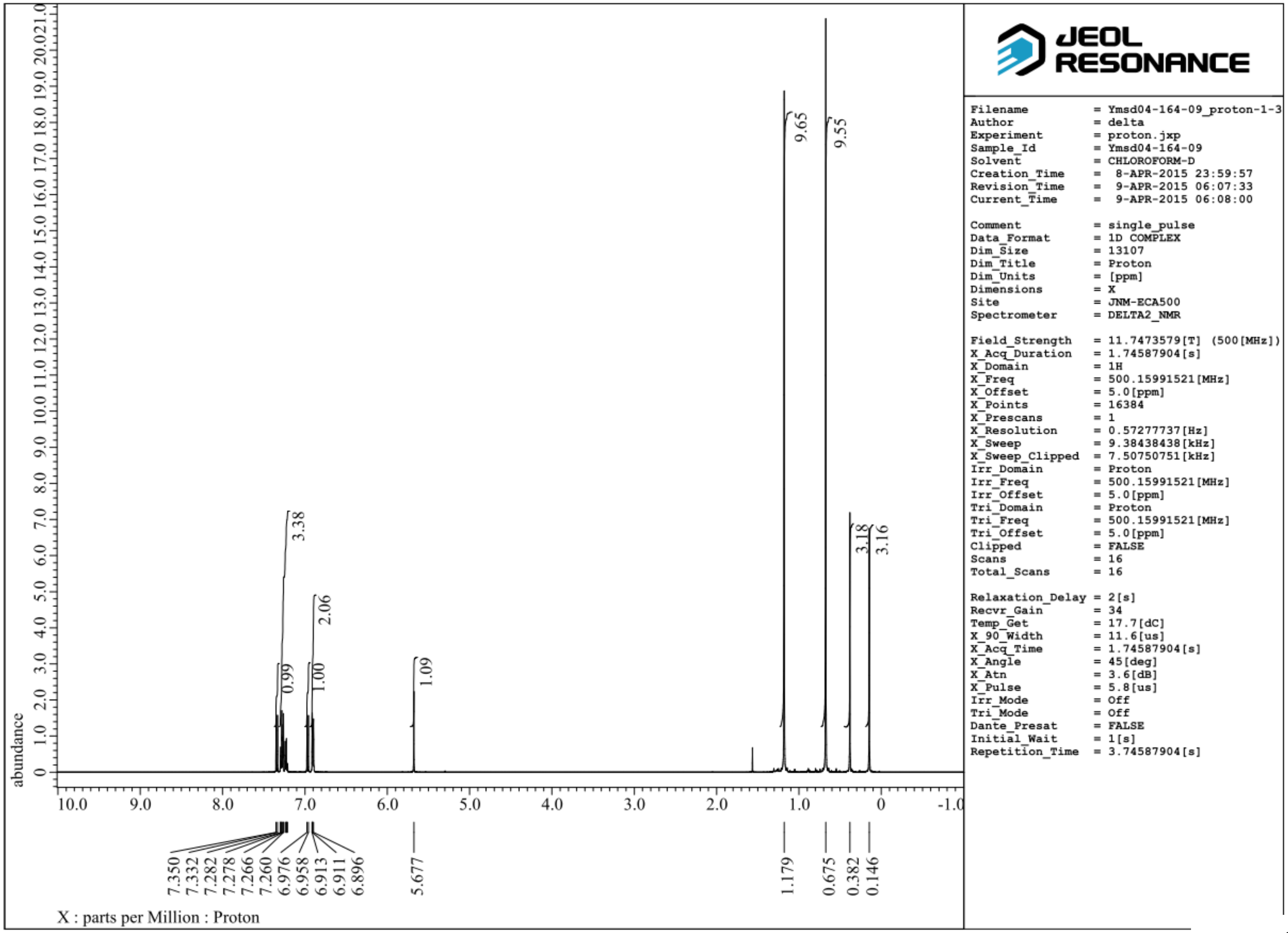

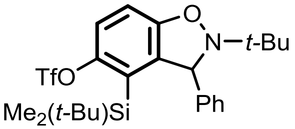

proximal-**10d**

You created this PDF from an application that is not licensed to print to novaPDF printer (http://www.novapdf.com)

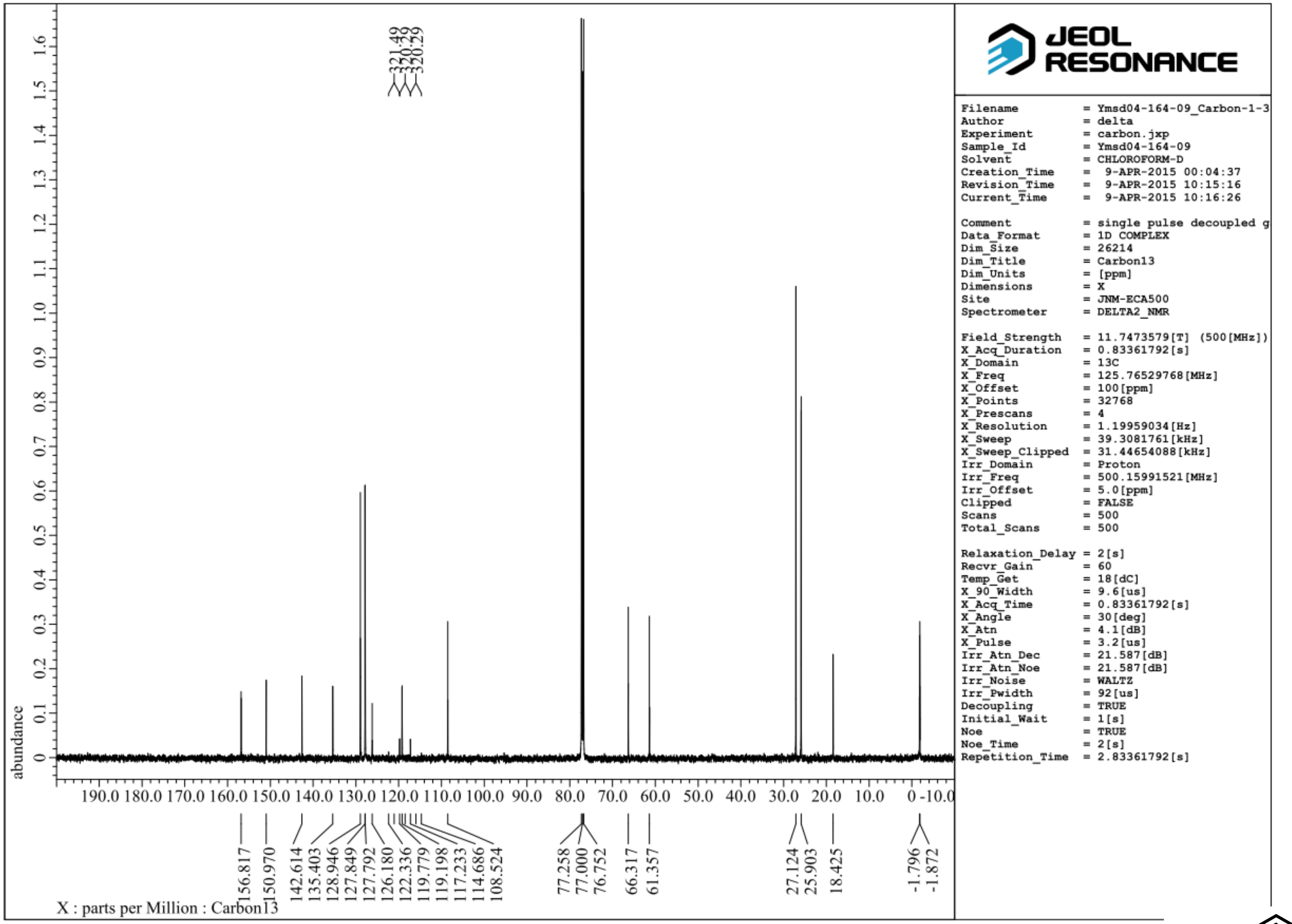

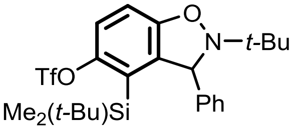

proximal-**10d**

You created this PDF from an application that is not licensed to print to novaPDF printer (http://www.novapdf.com)

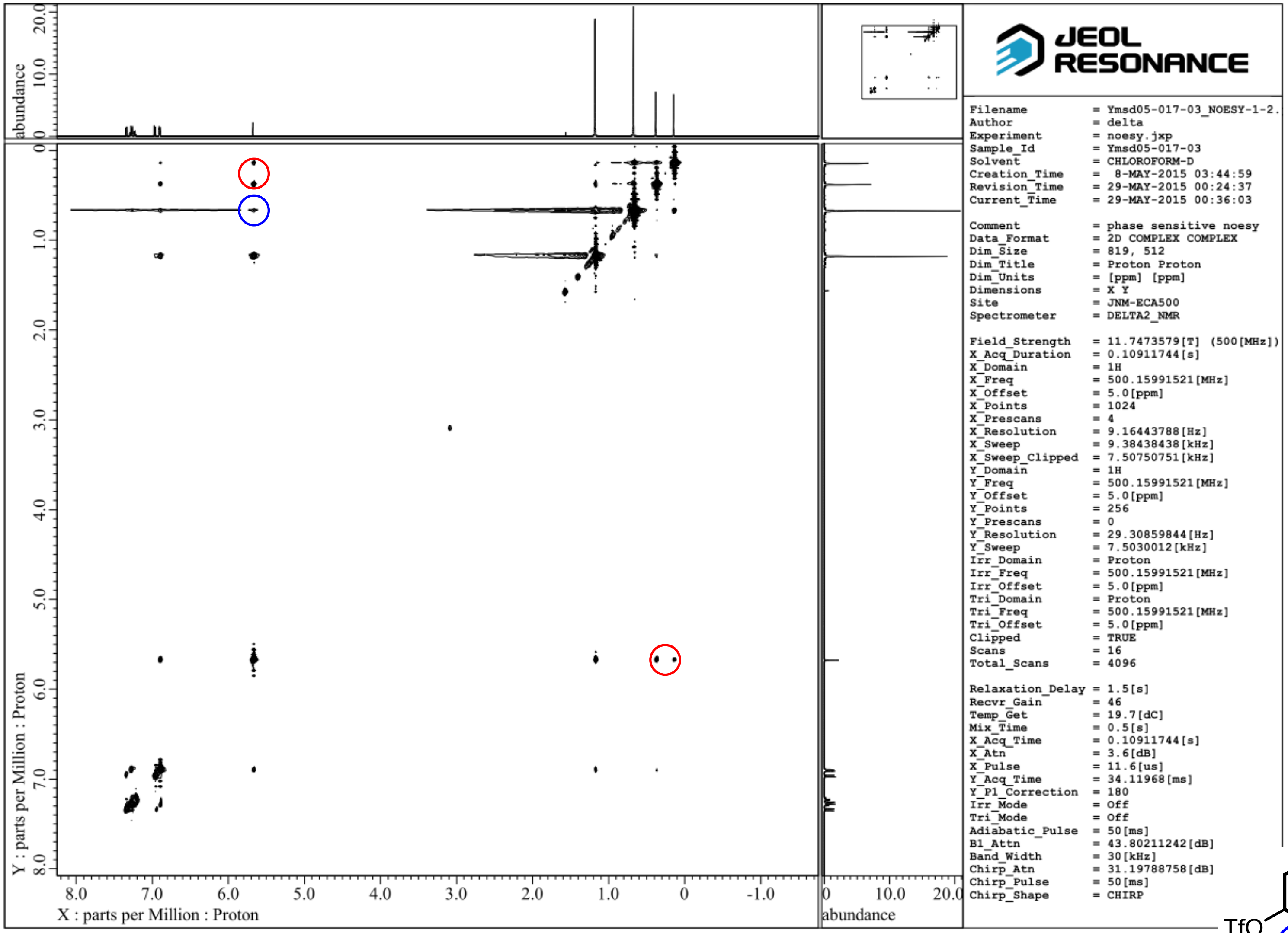

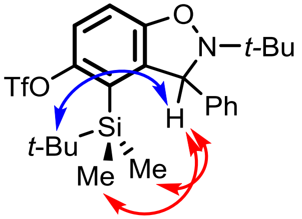

proximal-**10d**

You created this PDF from an application that is not licensed to print to novaPDF printer (http://www.novapdf.com)

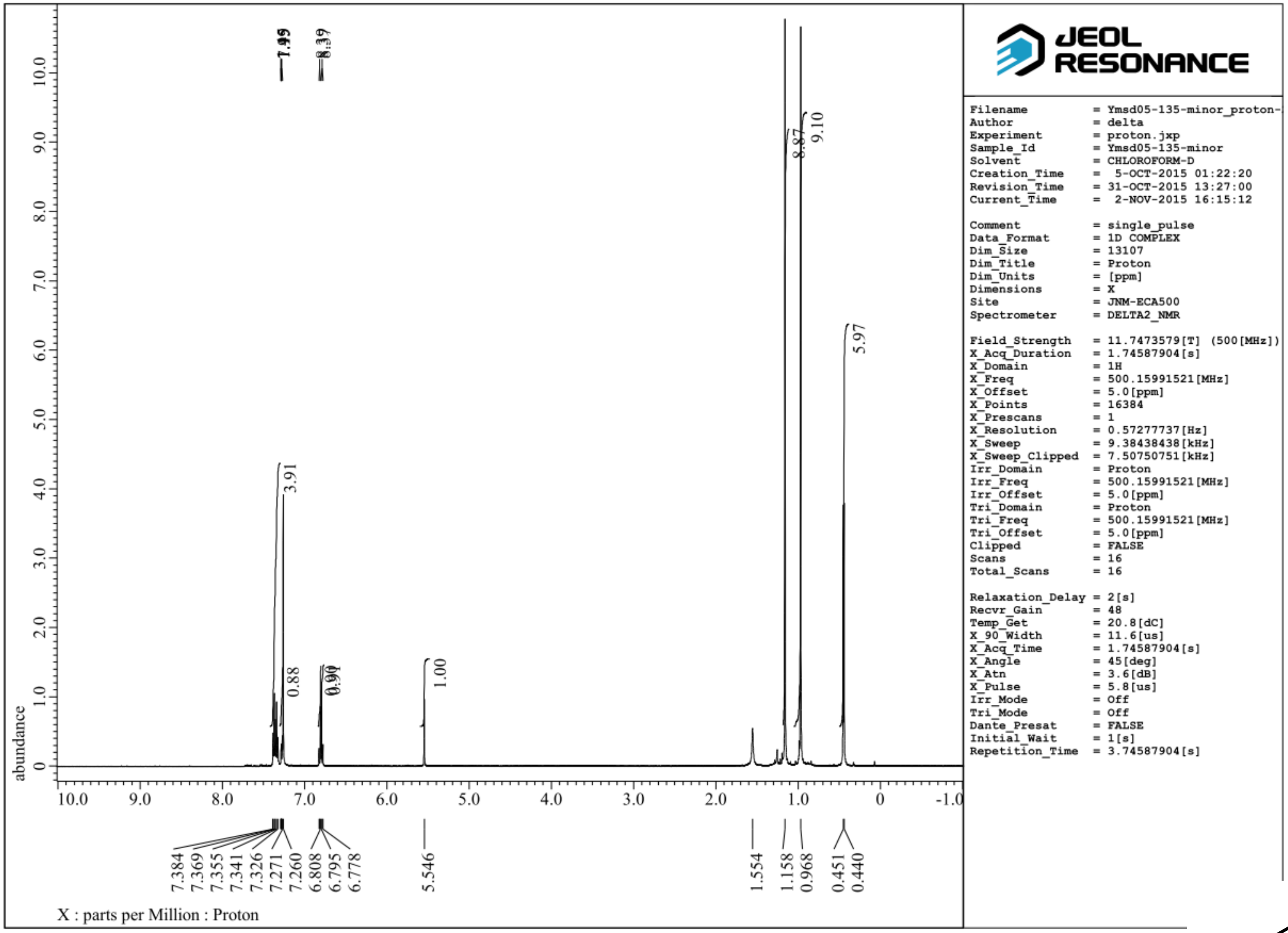

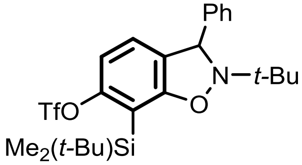

distal-**10d**

You created this PDF from an application that is not licensed to print to novaPDF printer (http://www.novapdf.com)

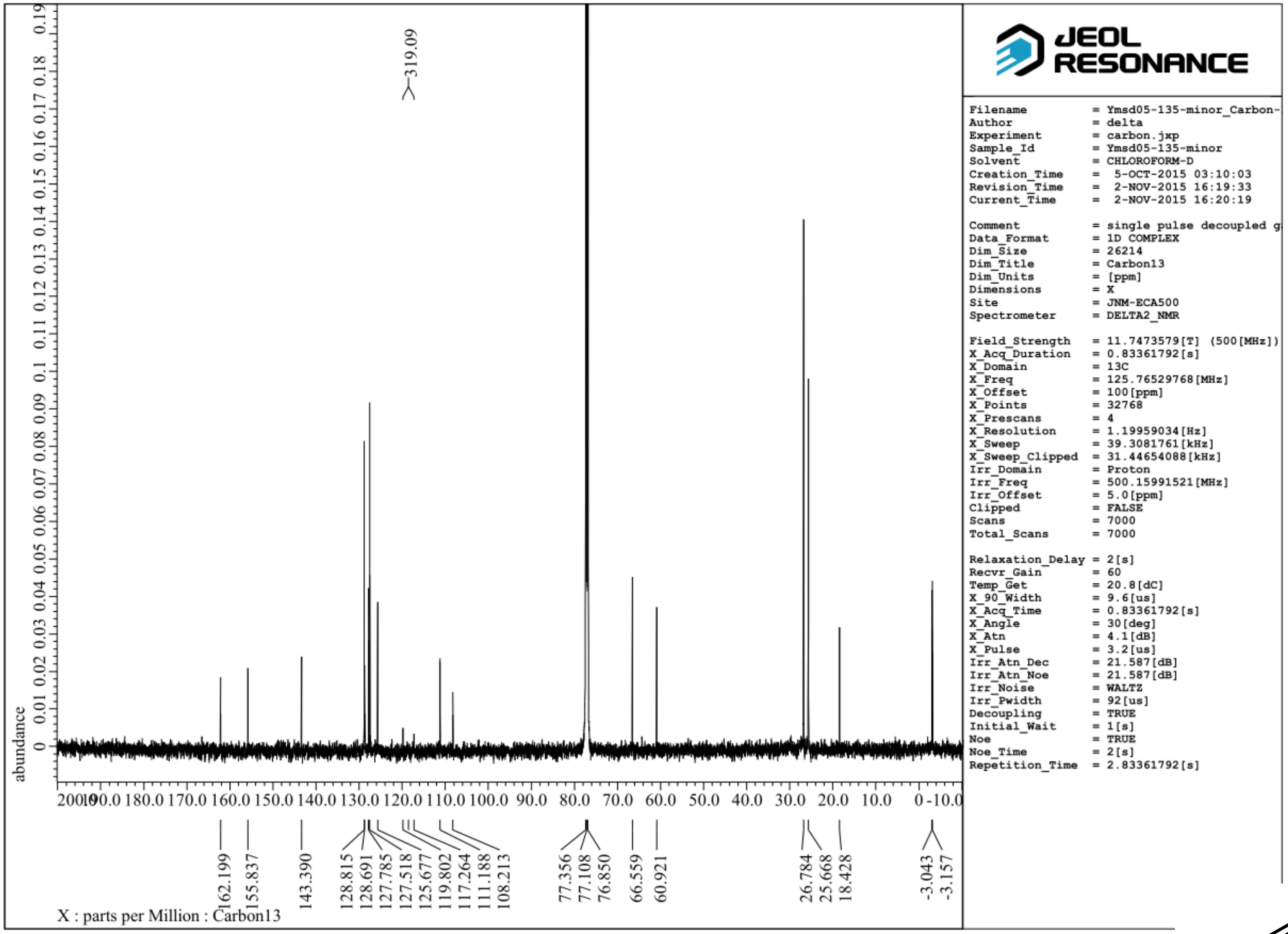

You created this PDF from an application that is not licensed to print to novaPDF printer (http://www.novapdf.com)

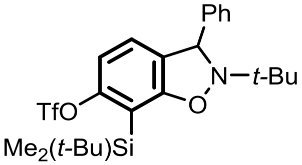

distal-**10d**

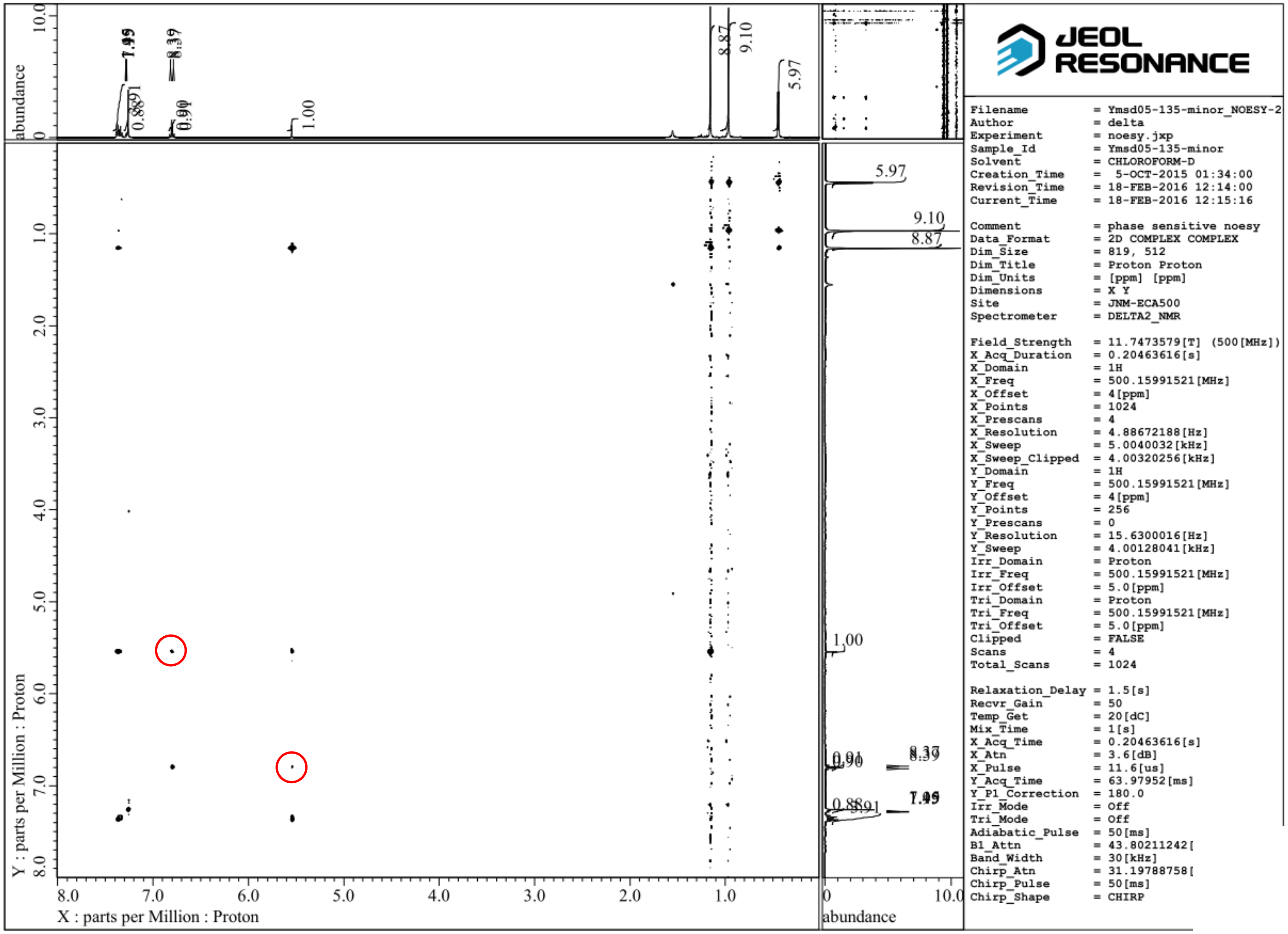

abundance
X : parts per Million : Proton
Y : parts per Million : Proton
JEOL RESONANCE
Filename = Ymsd05-135-minor_NOESY-2
Author = delta
Experiment = noesy.jxp
Sample_Id = Ymsd05-135-minor
Solvent = CHLOROFORM-D
Creation_Time = 5-OCT-2015 01:34:00
Revision_Time = 18-FEB-2016 12:14:00
Current_Time = 18-FEB-2016 12:15:16
Comment = phase sensitive noesy
Data_Format = 2D COMPLEX COMPLEX
Dim_Size = 819, 512
Dim_Title = Proton Proton
Dim_Units = [ppm] [ppm]
Dimensions = X Y
Site = JNM-ECA500
Spectrometer = DELTA2_NMR
Field_Strength = 11.7473579[T] (500[MHz])
X_Acq_Duration = 0.20463616[s]
X_Domain = 1H
X_Freq = 500.15991521[MHz]
X_Offset = 4[ppm]
X_Points = 1024
X_Prescans = 4
X_Resolution = 4.88672188[Hz]
X_Sweep = 5.0040032[kHz]
X_Sweep_Clipped = 4.00320256[kHz]
Y_Domain = 1H
Y_Freq = 500.15991521[MHz]
Y_Offset = 4[ppm]
Y_Points = 256
Y_Prescans = 0
Y_Resolution = 15.6300016[Hz]
Y_Sweep = 4.00128041[kHz]
Irr_Domain = Proton
Irr_Freq = 500.15991521[MHz]
Irr_Offset = 5.0[ppm]
Tri_Domain = Proton
Tri_Freq = 500.15991521[MHz]
Tri_Offset = 5.0[ppm]
Clipped = FALSE
Scans = 4
Total_Scans = 1024
Relaxation_Delay = 1.5[s]
Recvr_Gain = 50
Temp_Get = 20[dC]
Mix_Time = 1[s]
X_Acq_Time = 0.20463616[s]
X_Atn = 3.6[dB]
X_Pulse = 11.6[us]
Y_Acq_Time = 63.97952[ms]
Y_P1_Correction = 180.0
Irr_Mode = Off
Tri_Mode = Off
Adiabatic_Pulse = 50[ms]
B1_Attn = 43.80211242[
Band_Width = 30[kHz]
Chirp_Atn = 31.19788758[
Chirp_Pulse = 50[ms]
Chirp_Shape = CHIRP

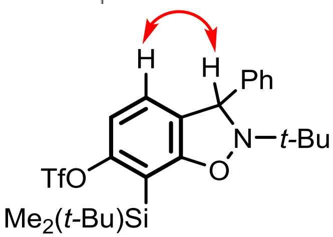

H
H
Ph
N
t-Bu
O
TfO
Me2(t-Bu)Si

distal-**10d**

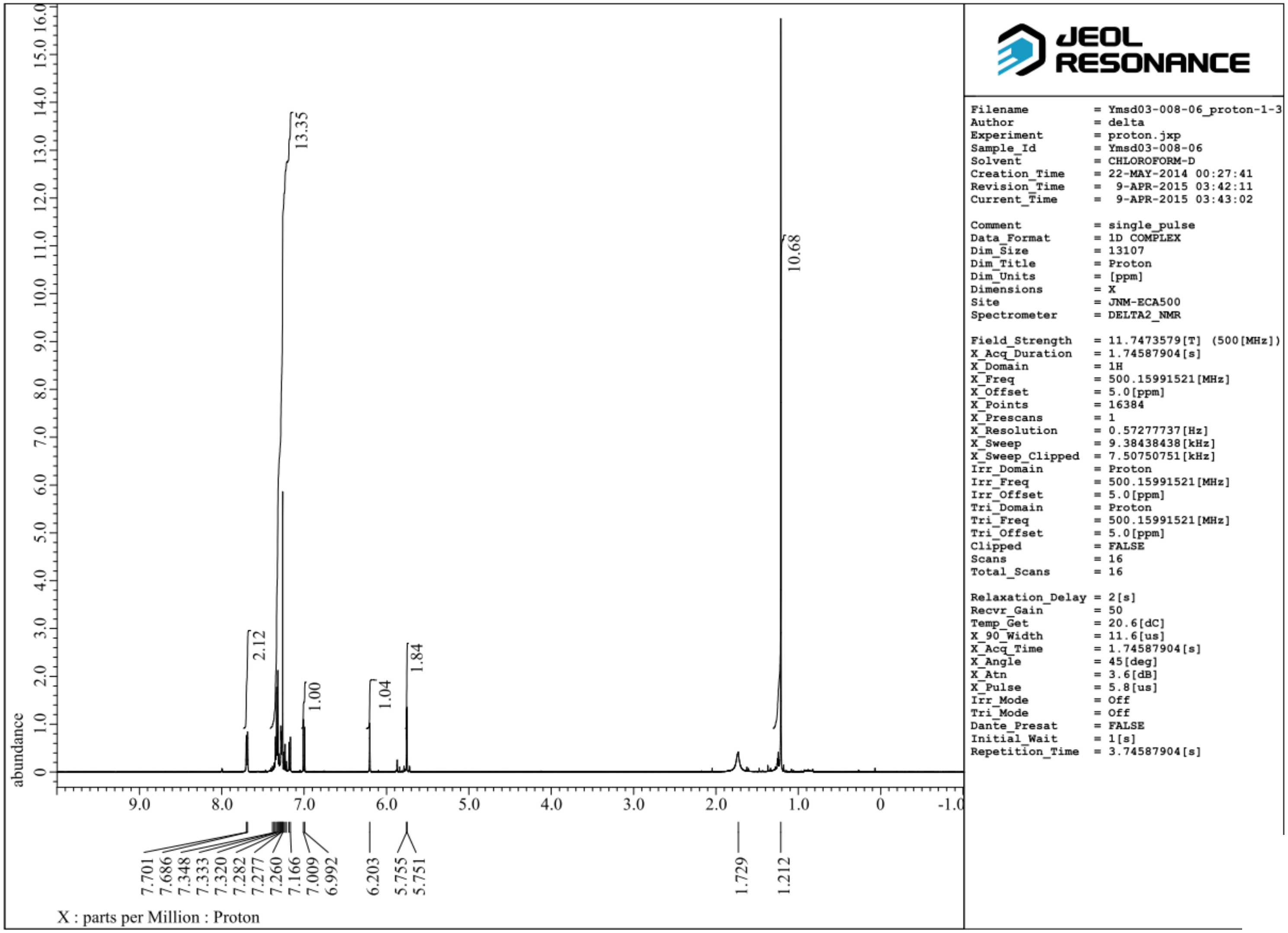

You created this PDF from an application that is not licensed to print to novaPDF printer (http://www.novapdf.com)

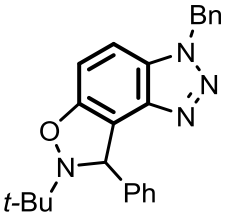

distal-**3e**

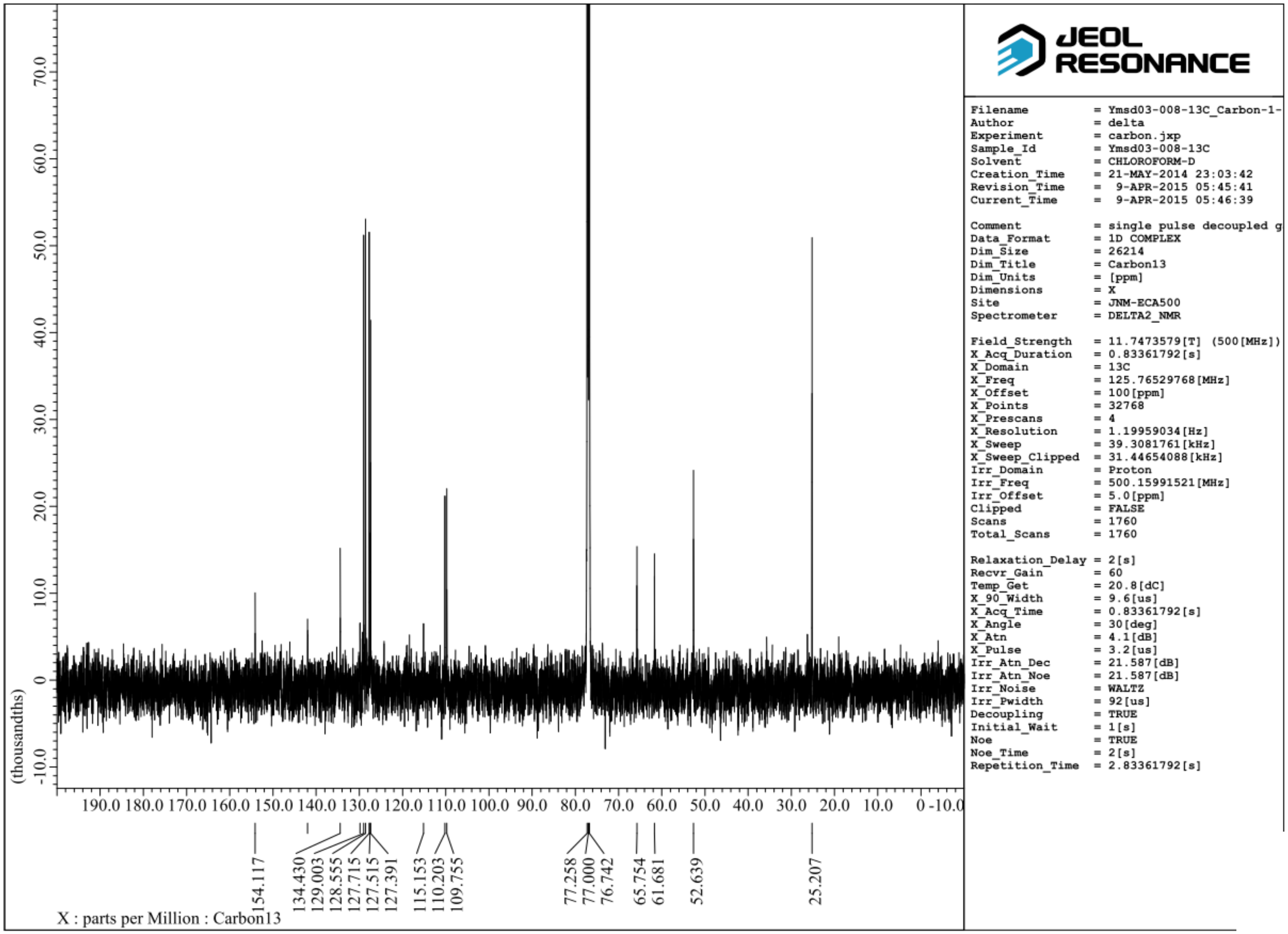

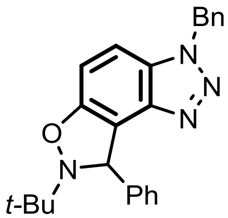

distal-**3e**

You created this PDF from an application that is not licensed to print to novaPDF printer (http://www.novapdf.com)

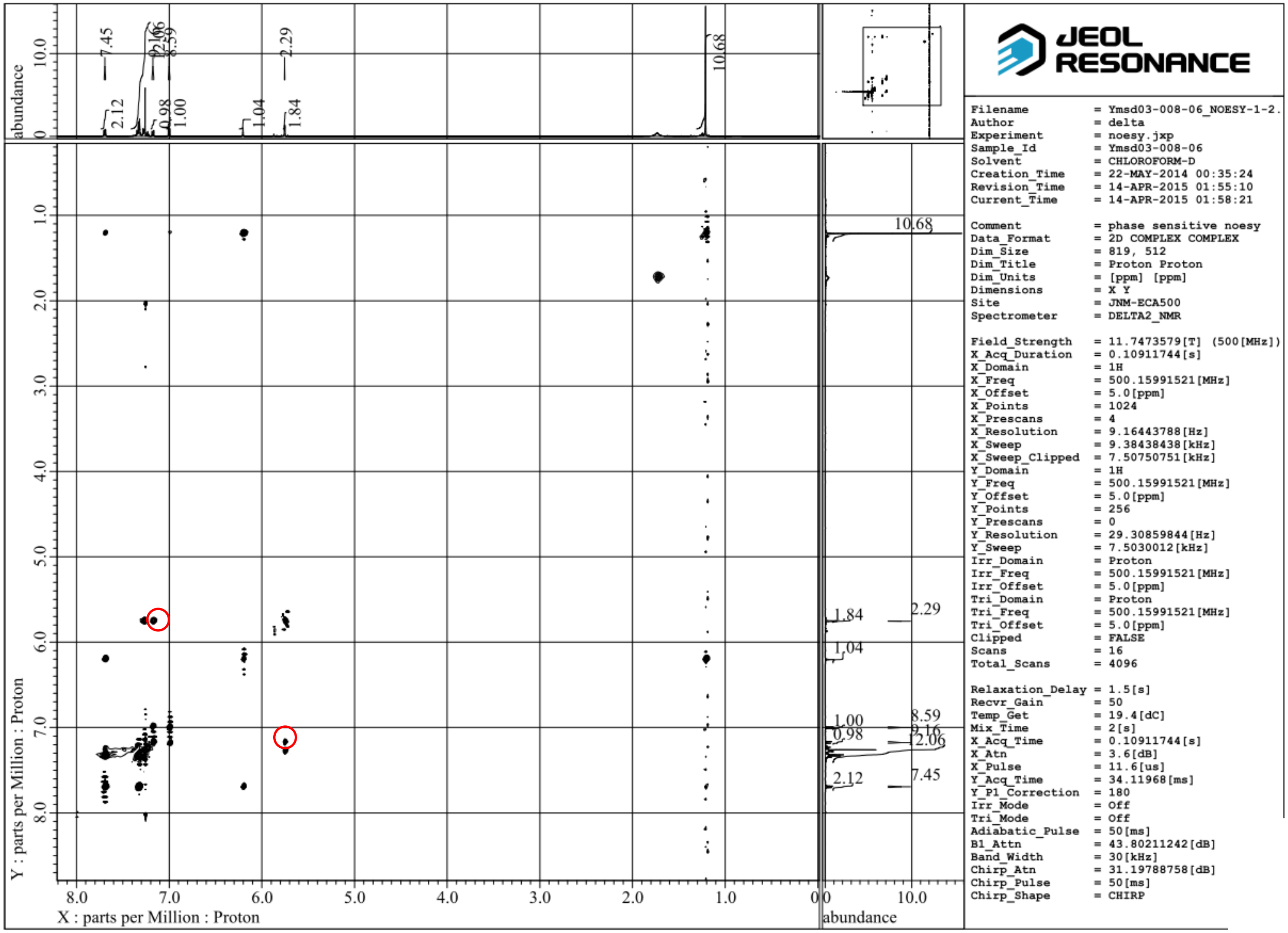

You created this PDF from an application that is not licensed to print to novaPDF printer (http://www.novapdf.com)

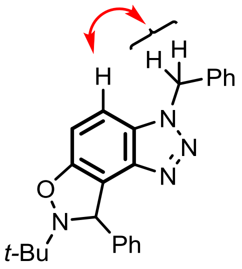

distal-**3e**

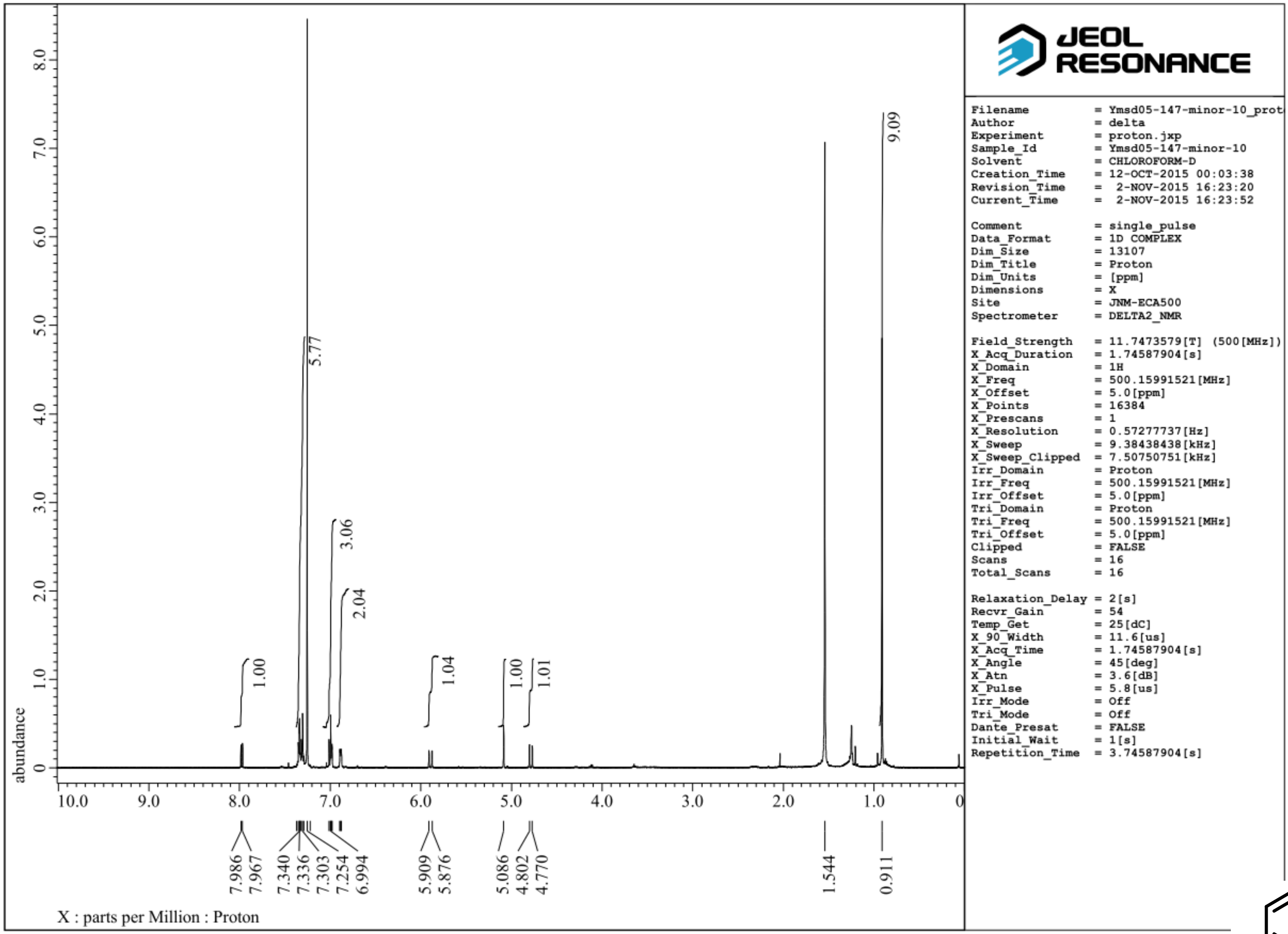

You created this PDF from an application that is not licensed to print to novaPDF printer (http://www.novapdf.com)

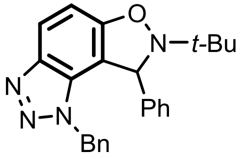

proximal-**3e**

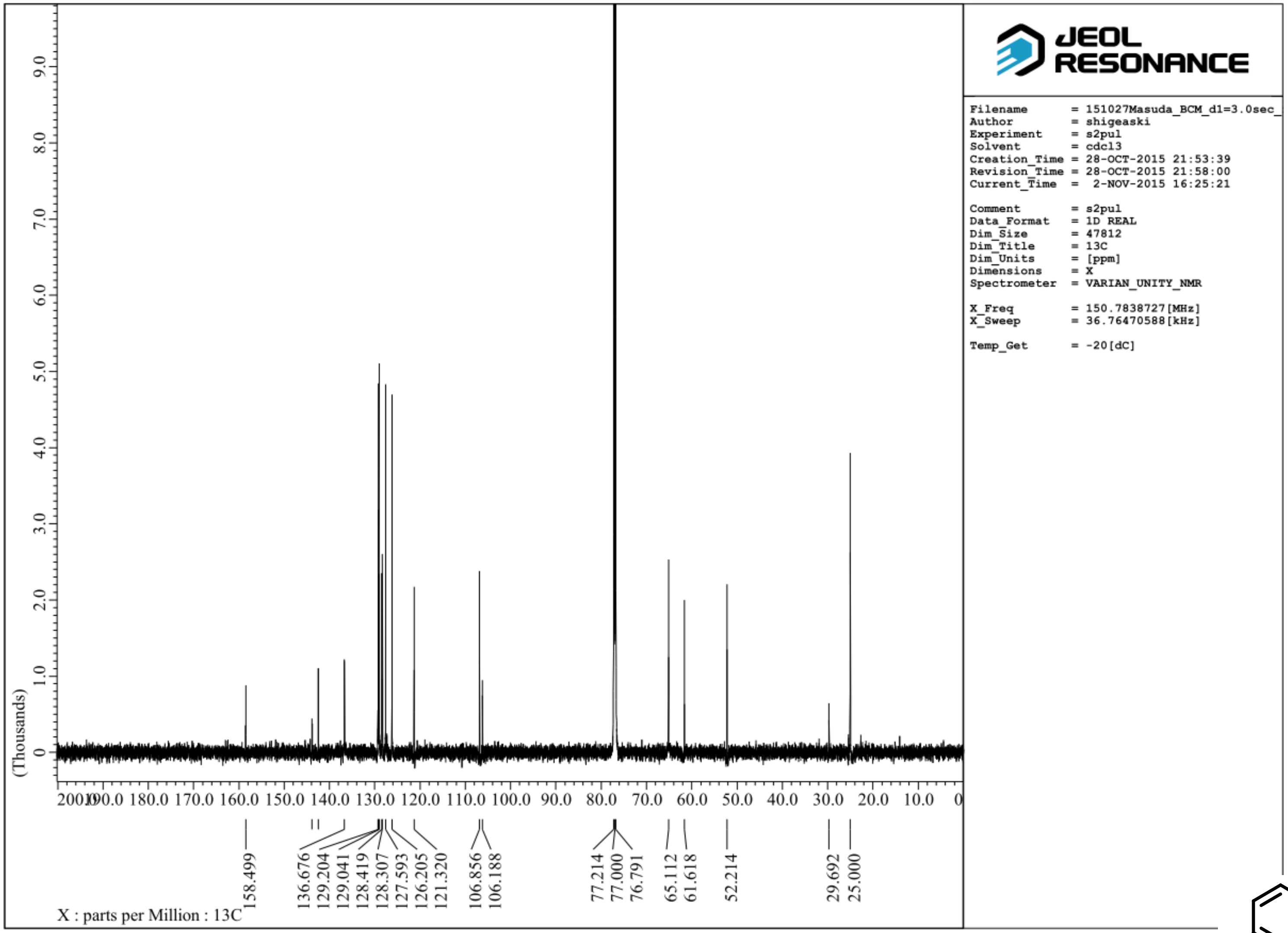

You created this PDF from an application that is not licensed to print to novaPDF printer (http://www.novapdf.com)

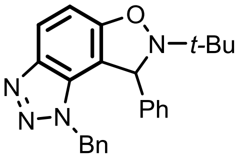

proximal-**3e**

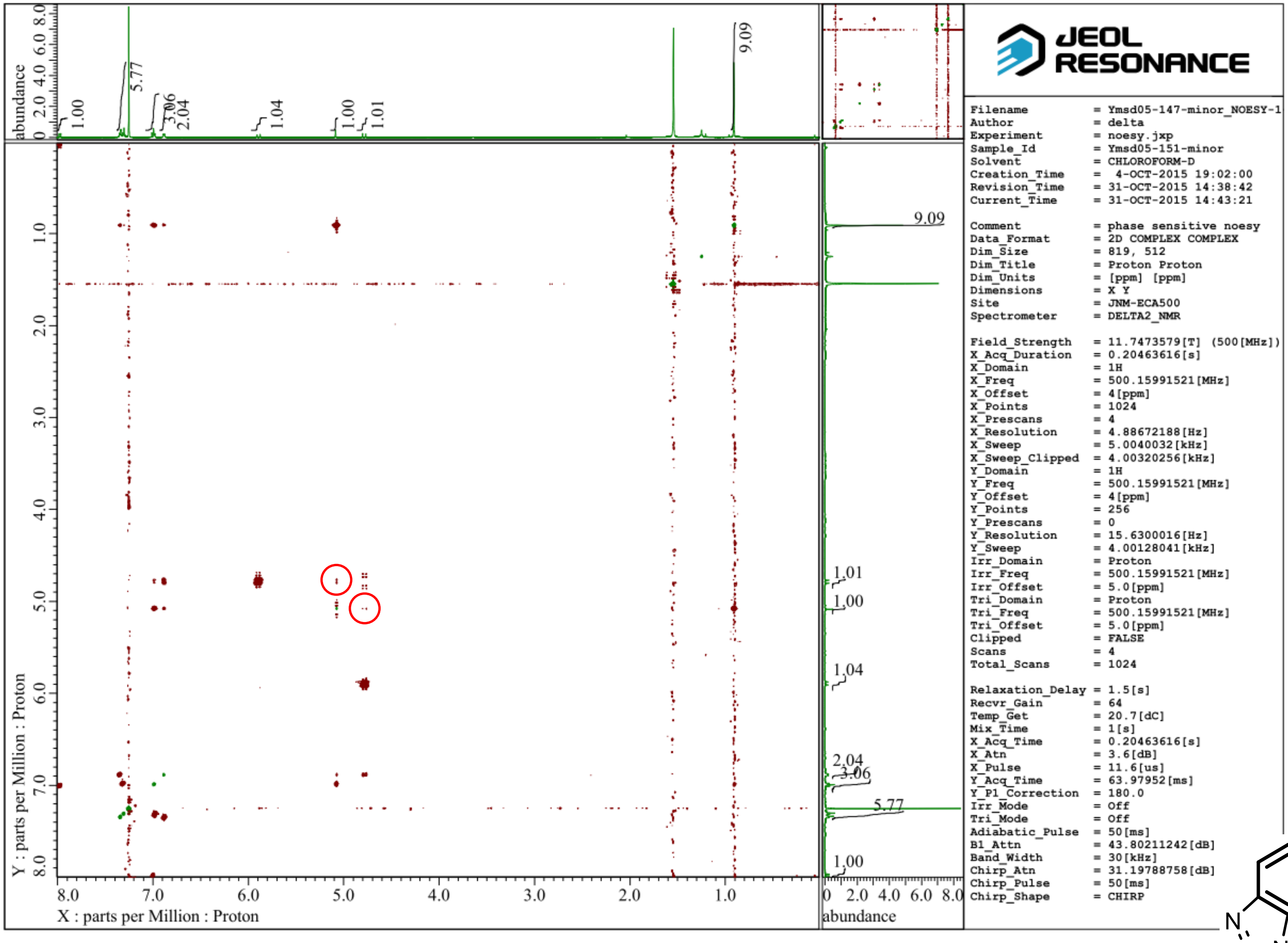

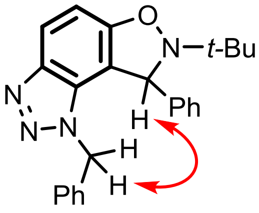

proximal-**3e**

You created this PDF from an application that is not licensed to print to novaPDF printer (http://www.novapdf.com)

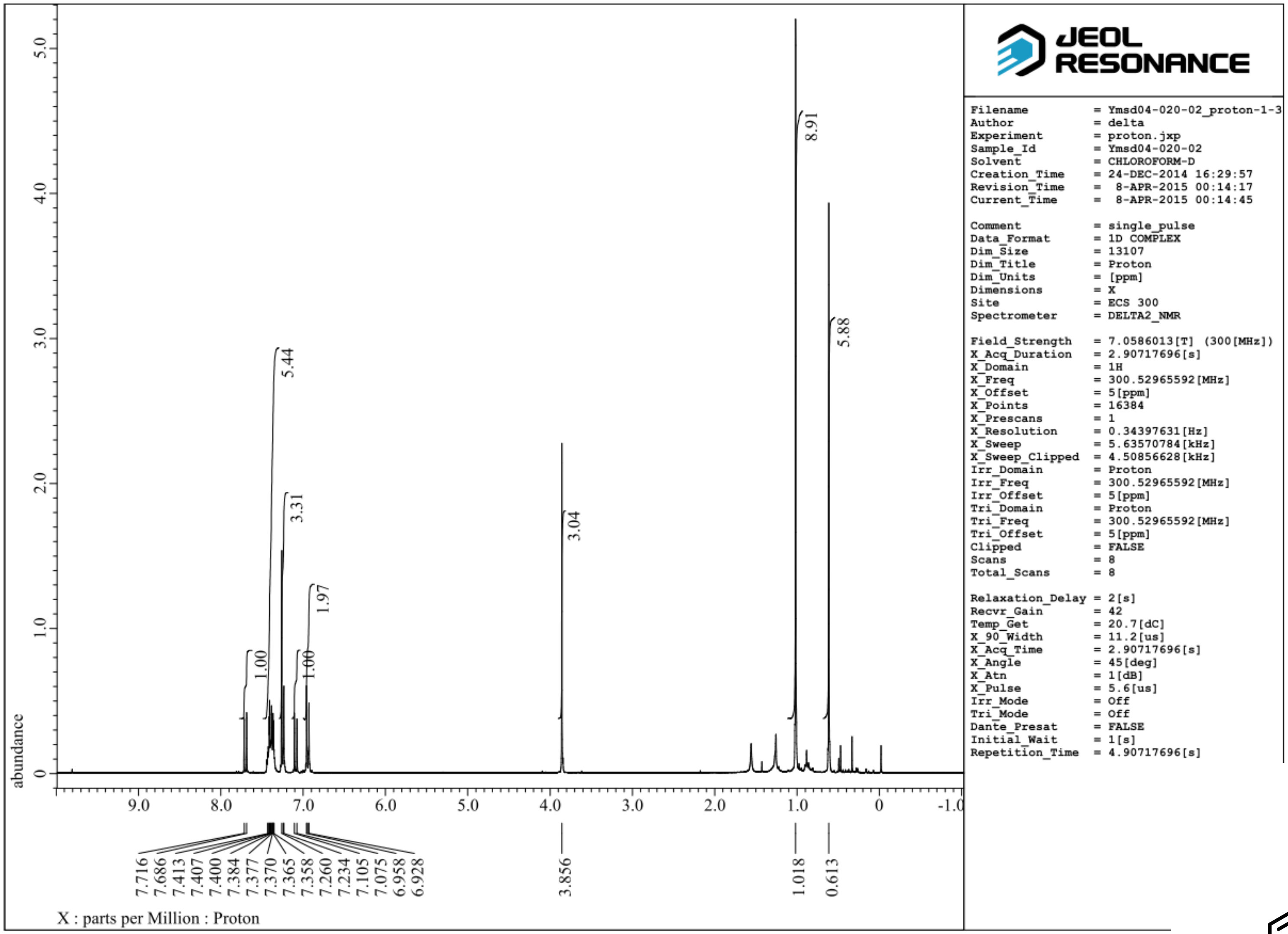

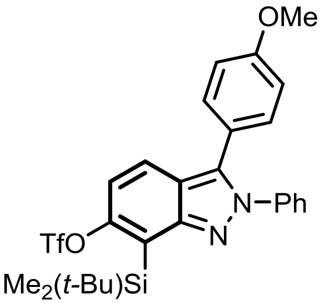

distal-**10e**

You created this PDF from an application that is not licensed to print to novaPDF printer (http://www.novapdf.com)

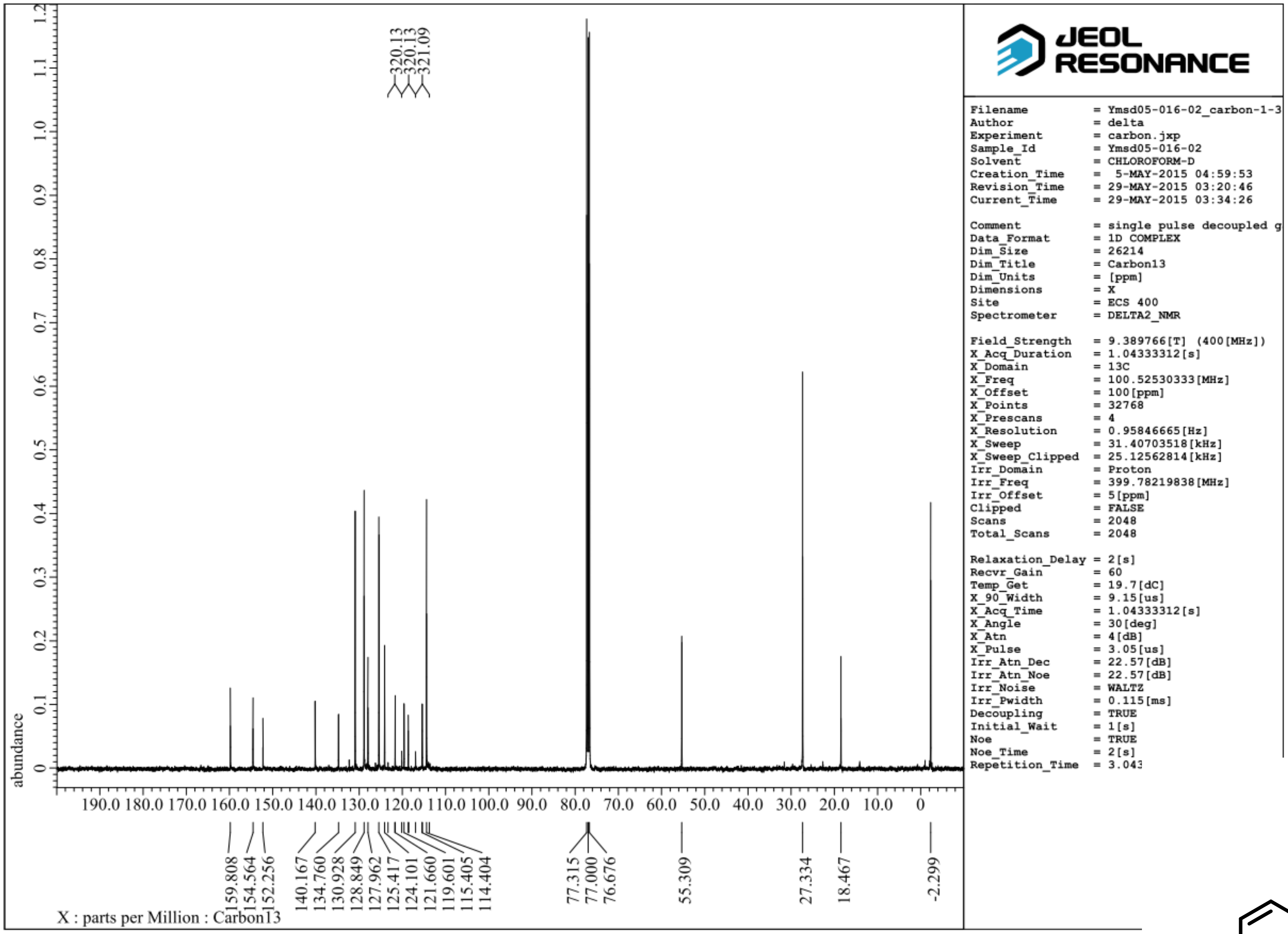

You created this PDF from an application that is not licensed to print to novaPDF printer (http://www.novapdf.com)

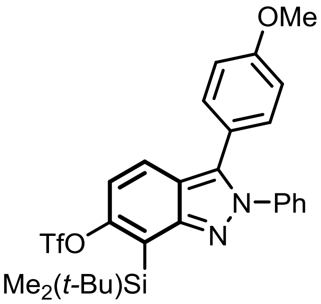

distal-**10e**

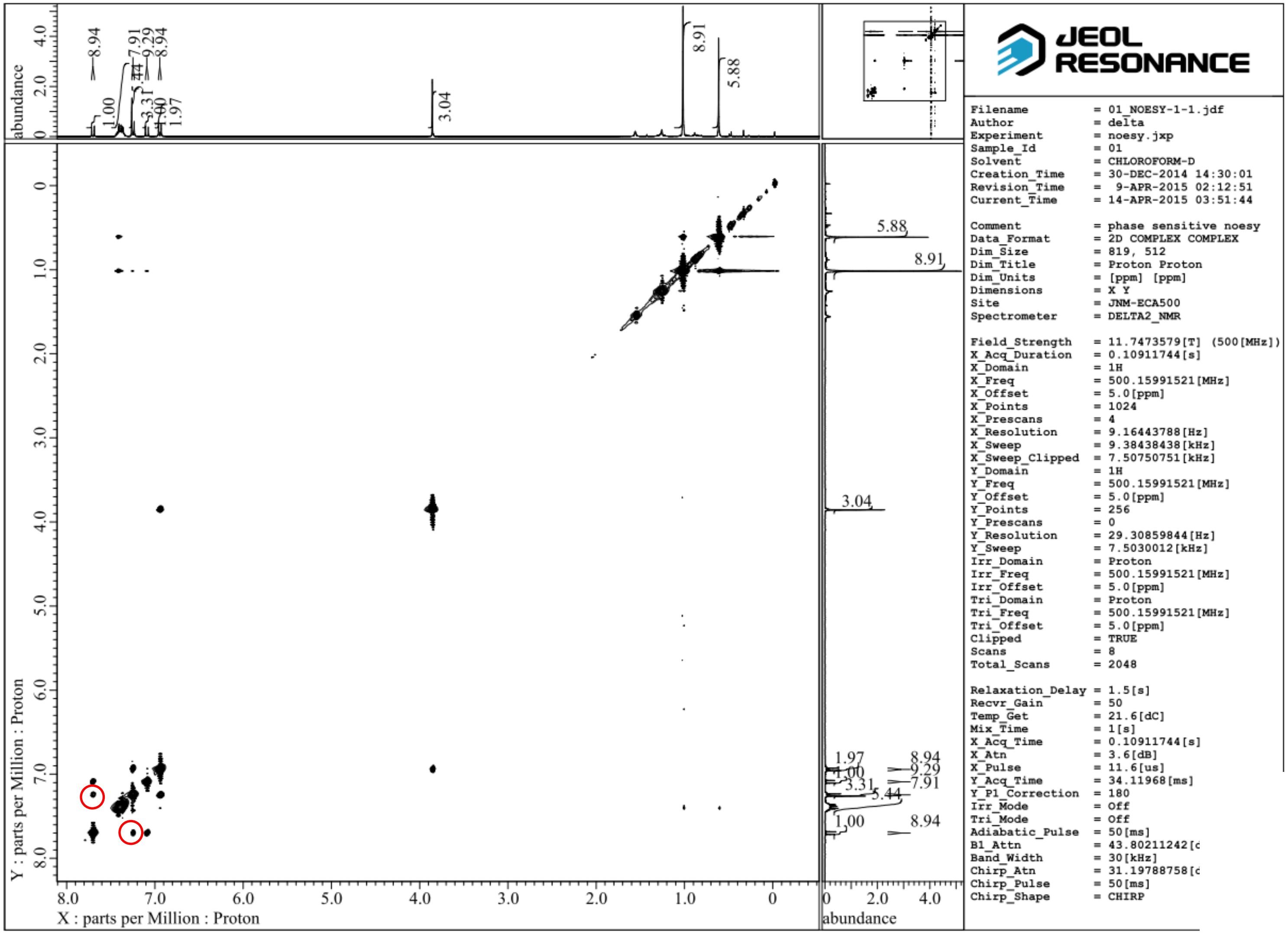

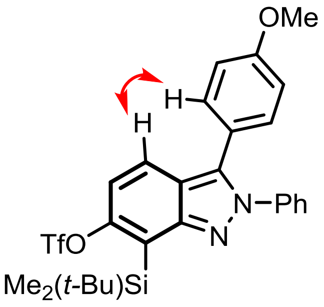

distal-**10e**

You created this PDF from an application that is not licensed to print to novaPDF printer (http://www.novapdf.com)

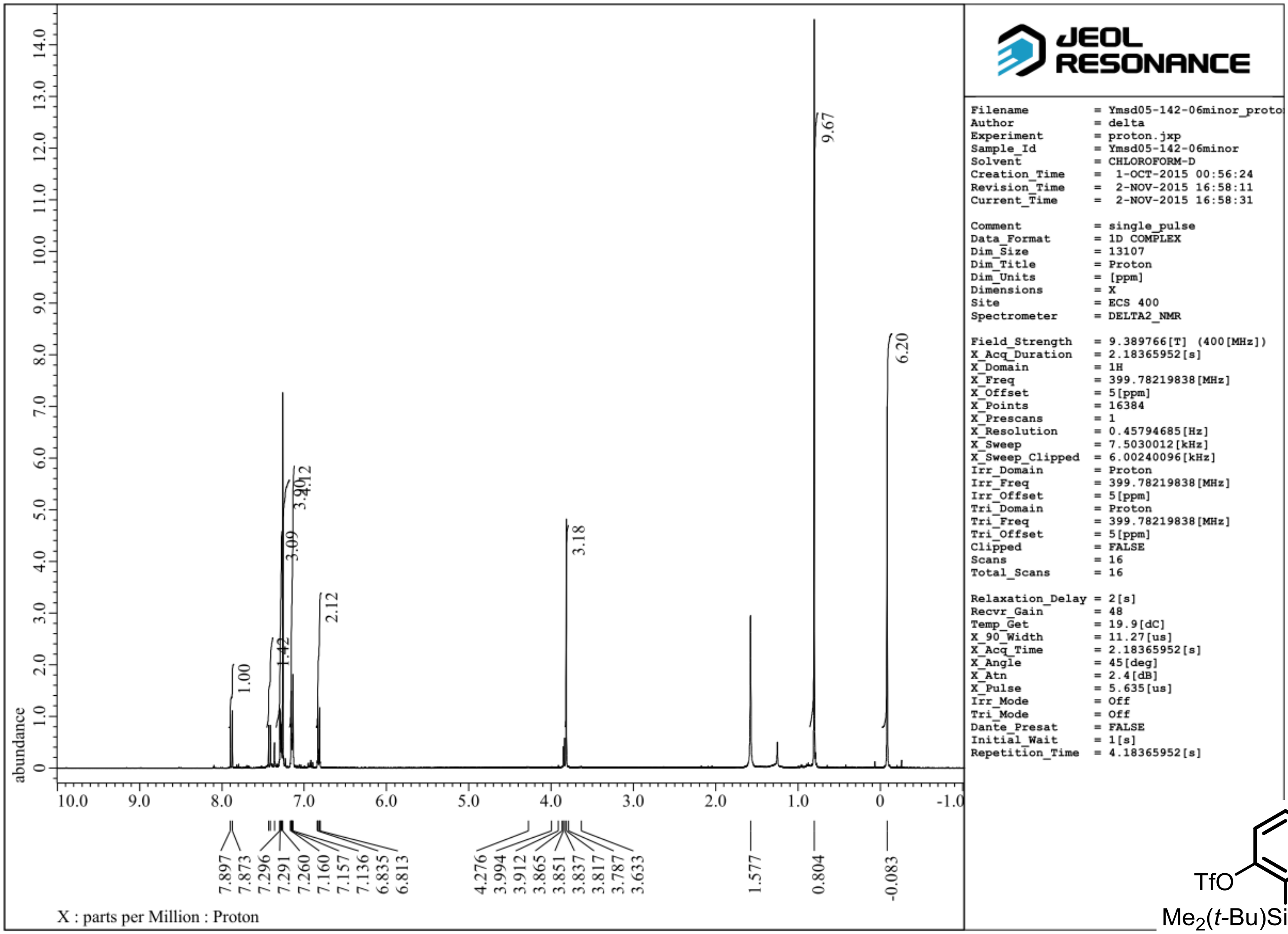

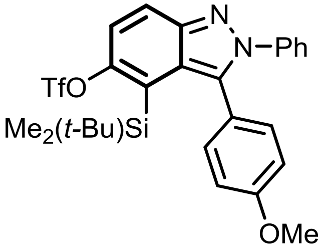

proximal-**10e**

You created this PDF from an application that is not licensed to print to novaPDF printer (http://www.novapdf.com)

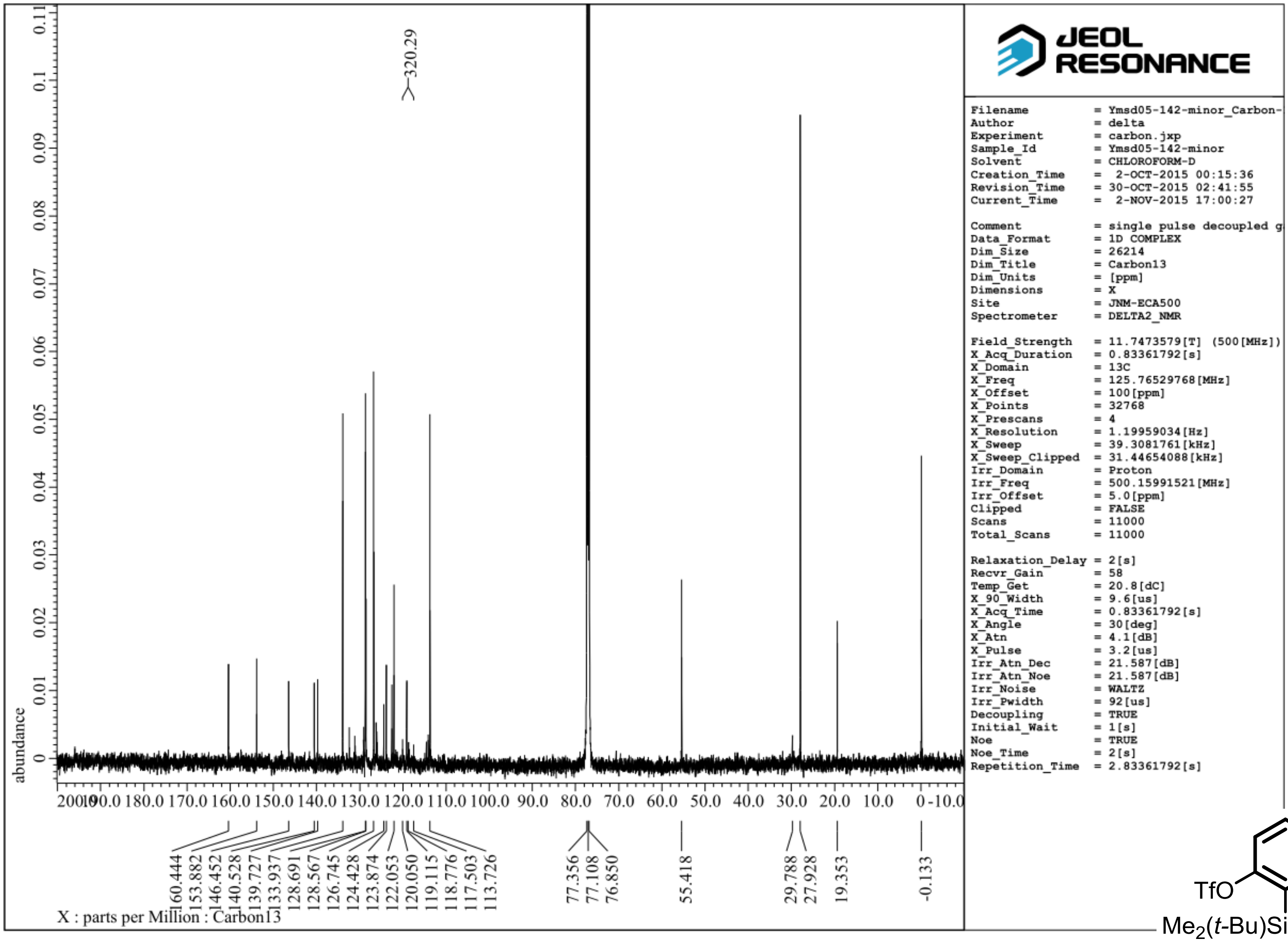

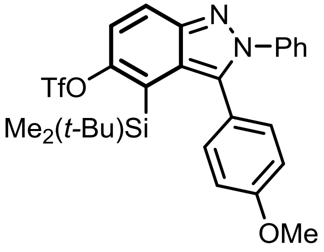

proximal-**10e**

You created this PDF from an application that is not licensed to print to novaPDF printer (http://www.novapdf.com)

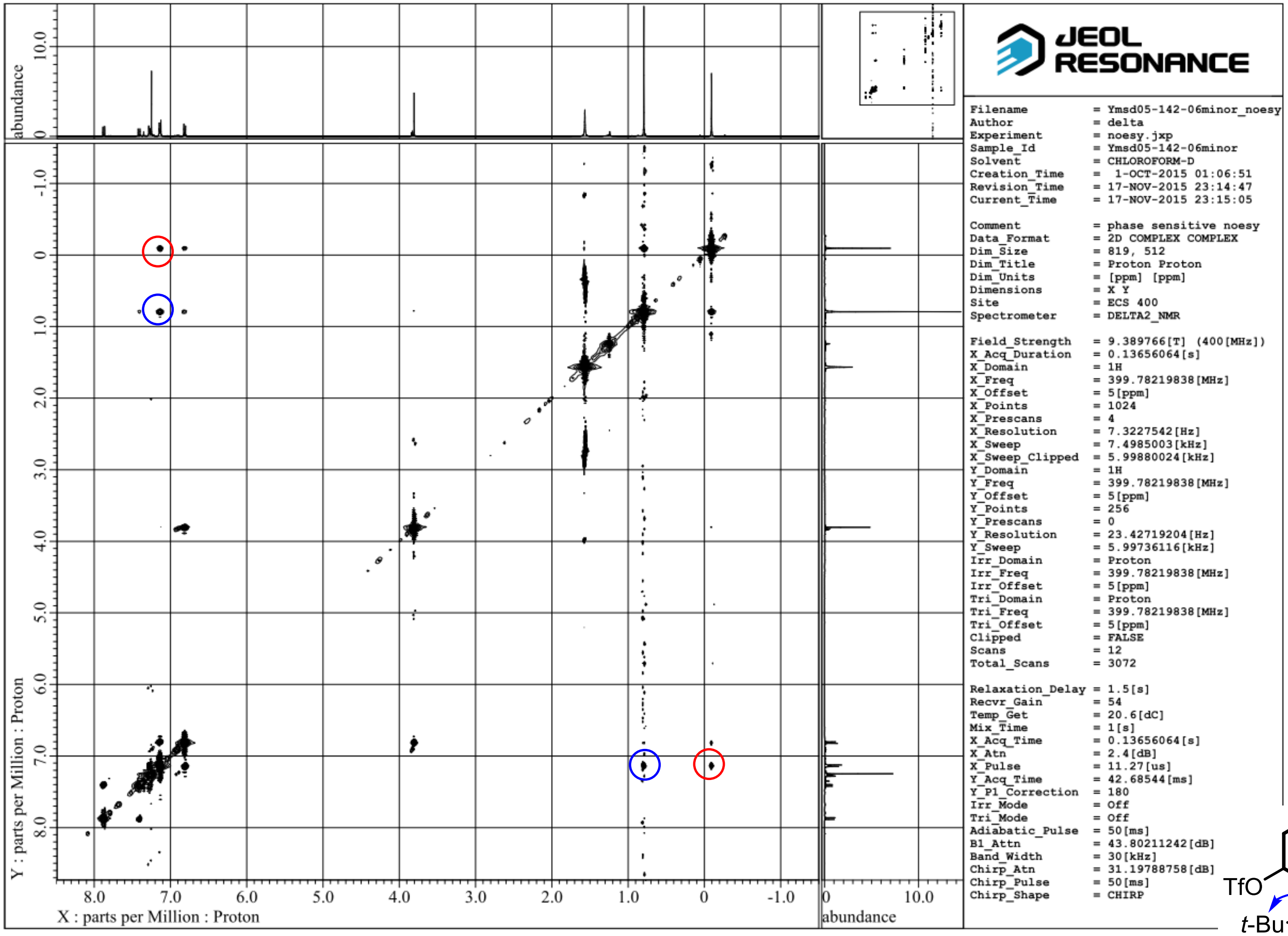

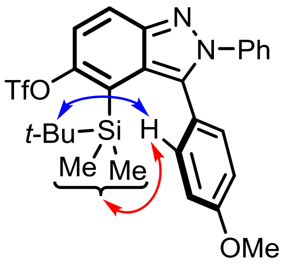

proximal-**10e**

You created this PDF from an application that is not licensed to print to novaPDF printer (http://www.novapdf.com)

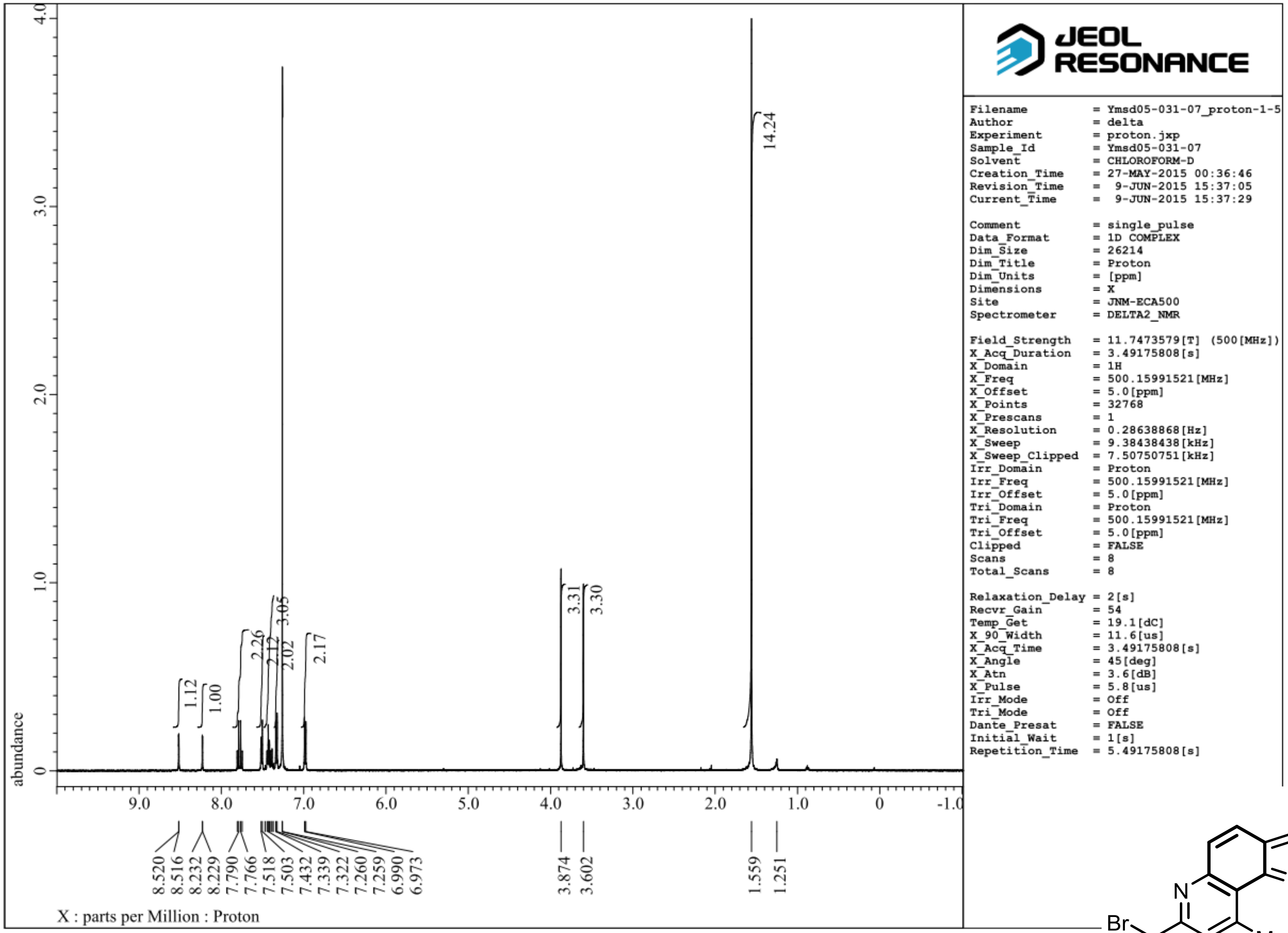

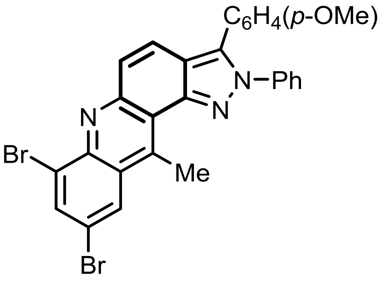

proximal-**3f**

You created this PDF from an application that is not licensed to print to novaPDF printer (http://www.novapdf.com)

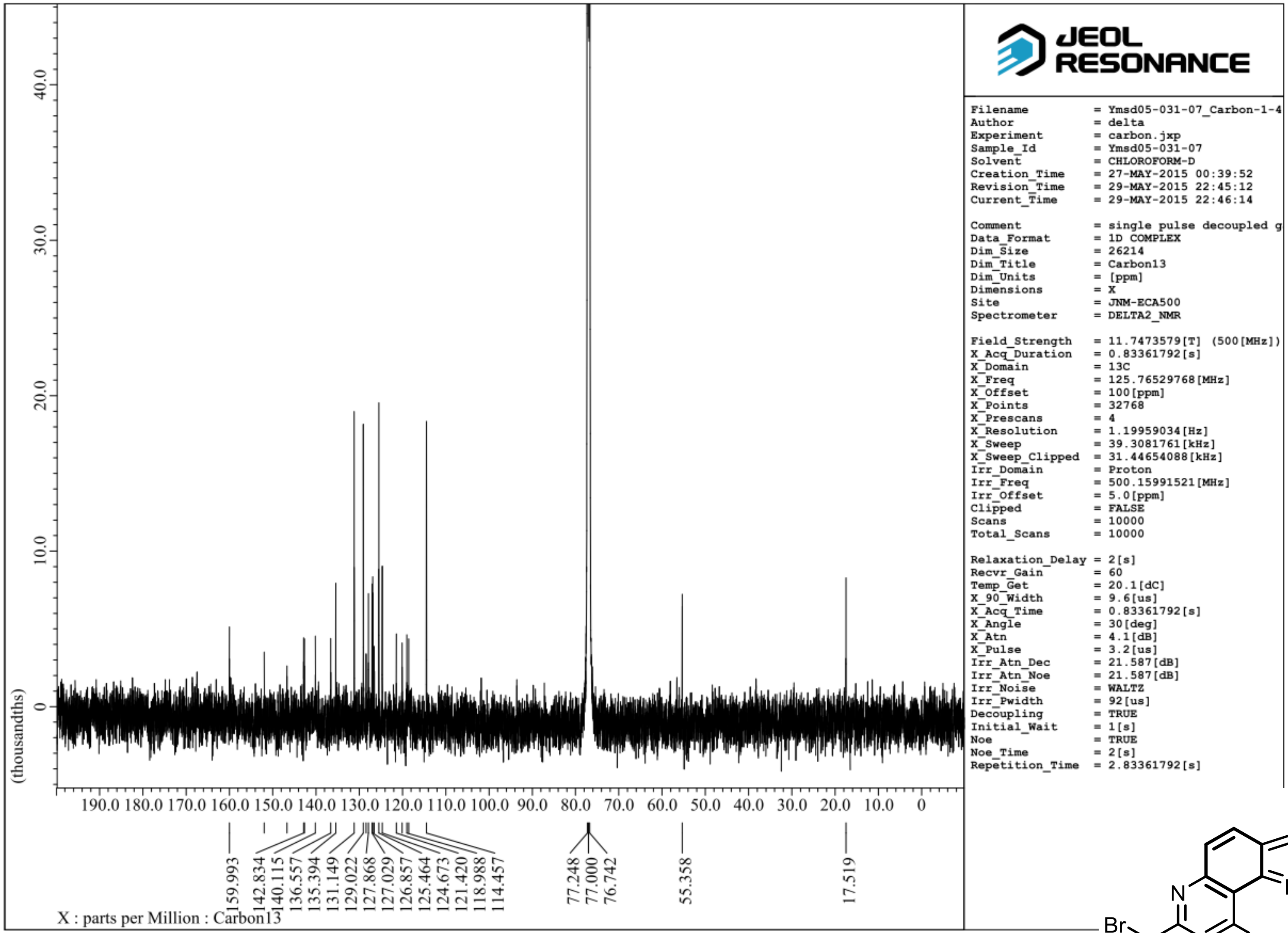

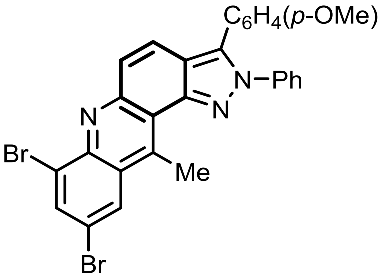

proximal-**3f**

You created this PDF from an application that is not licensed to print to novaPDF printer (http://www.novapdf.com)

Ymsd05-031-06

exp518 TOCSY

```
      SAMPLE                  FLAGS
date    Jun  5 2015   hs                  nn
solvent       cdcl3   sspul                y
sample                PFGflg               y
   ACQUISITION        hsglvl            5760
sw           9615.4        SPECIAL
at            0.150   temp              30.0
np             2884   gain                48
fb             4000   spin          not used
ss               32     F2 PROCESSING
d1            1.000   gf               0.034
nt               32   gfs           not used
  2D ACQUISITION      fn                4096
sw1          9615.4     F1 PROCESSING
ni              256   gf1              0.015
   TRANSMITTER        gfs1          not used
tn               H1   proc1               lp
sfrq        599.927   fn1               4096
tof           600.0        DISPLAY
tpwr             55   sp              4118.7
pw            7.350   wp               986.0
      TOCSY           sp1             4090.6
mix       undefined   wp1             1070.5
slpw      undefined   rfl             1243.0
slpwr     undefined   rfp                  0
trim         0.0020   rfl1            1243.0
  PRESATURATION       rfp1                 0
satmode           n          PLOT
wet               n   wc               107.2
    DECOUPLER         sc                 6.7
dn              C13   wc2              134.2
dm              nnn   sc2                  0
                      vs                6024
                      th                   2
                      ai   cdc   ph
```

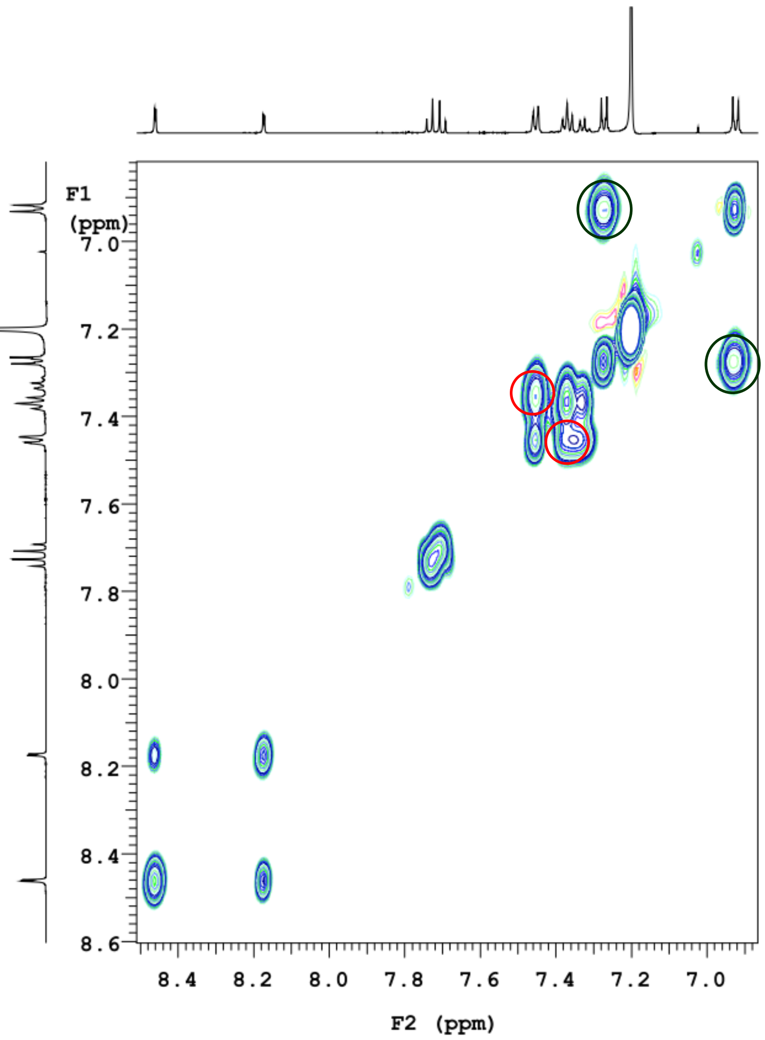

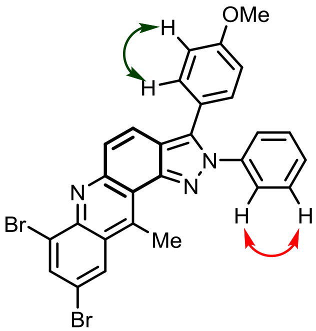

proximal-**3f**

Ymsd05-031-06

exp519 ROESYAD

```
        SAMPLE
date      Jun  5 2015
solvent         cdcl3
sample
     ACQUISITION
sw             9615.4
at              0.150
np               2884
fb               4000
ss                 32
d1              1.000
nt                 64
   2D ACQUISITION
sw1            9615.4
ni                256
     TRANSMITTER
tn                 H1
sfrq          599.927
tof             600.0
tpwr               55
pw              7.350
        TOCSY
mixR            0.200
slpwrR             43
slpwR          32.426
trim           0.0020
   PRESATURATION
satmode             n
wet                 n
      DECOUPLER
dn                C13
dm                nnn
```

```
        FLAGS
hs                 nn
sspul               y
PFGflg              y
hsglvl           5760
       SPECIAL
temp             30.0
gain               48
spin         not used
  F2 PROCESSING
gf              0.033
gfs          not used
fn               4096
  F1 PROCESSING
gf1             0.012
gfs1         not used
proc1              lp
fn1              4096
       DISPLAY
sp             1851.0
wp             2878.0
sp1            1851.0
wp1            2878.0
rfl            1243.0
rfp                 0
rfl1           1243.0
rfp1                0
        PLOT
wc              107.2
sc                6.7
wc2             134.2
sc2                 0
vs              70955
th                  1
ai      ph
```

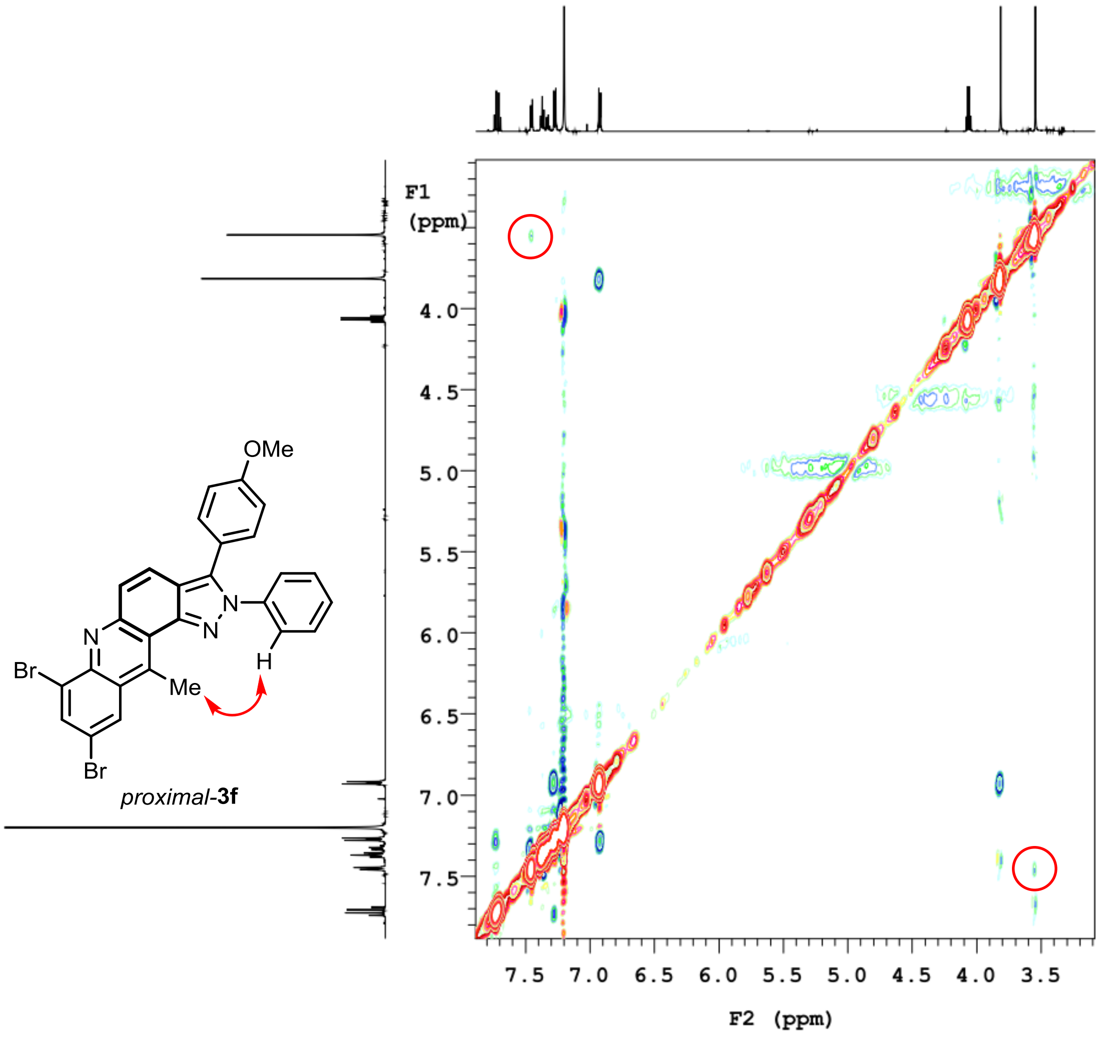

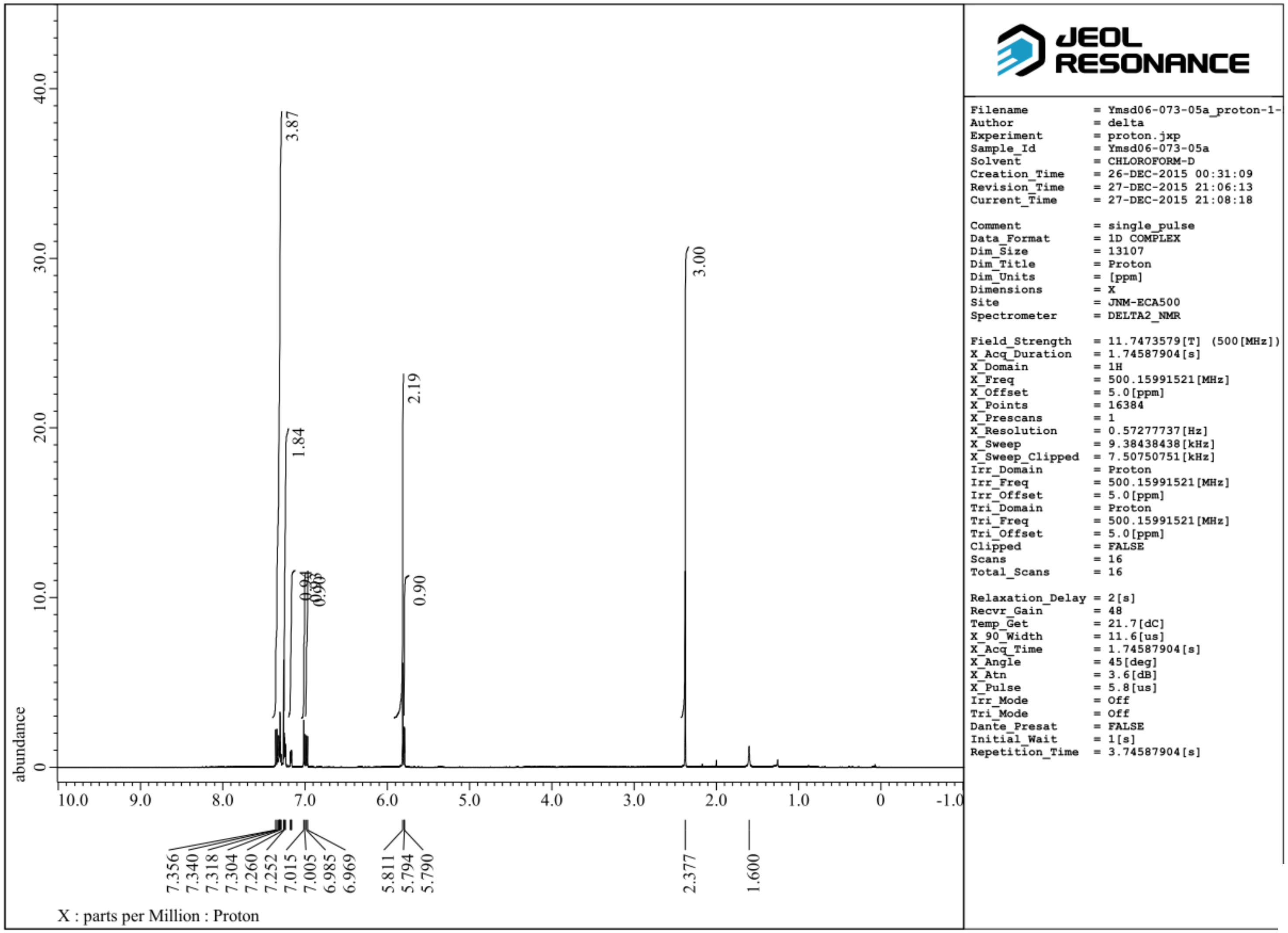

JEOL RESONANCE
Filename = Ymsd06-073-05a_proton-1-
Author = delta
Experiment = proton.jxp
Sample_Id = Ymsd06-073-05a
Solvent = CHLOROFORM-D
Creation_Time = 26-DEC-2015 00:31:09
Revision_Time = 27-DEC-2015 21:06:13
Current_Time = 27-DEC-2015 21:08:18
Comment = single_pulse
Data_Format = 1D COMPLEX
Dim_Size = 13107
Dim_Title = Proton
Dim_Units = [ppm]
Dimensions = X
Site = JNM-ECA500
Spectrometer = DELTA2_NMR
Field_Strength = 11.7473579[T] (500[MHz])
X_Acq_Duration = 1.74587904[s]
X_Domain = 1H
X_Freq = 500.15991521[MHz]
X_Offset = 5.0[ppm]
X_Points = 16384
X_Prescans = 1
X_Resolution = 0.57277737[Hz]
X_Sweep = 9.38438438[kHz]
X_Sweep_Clipped = 7.50750751[kHz]
Irr_Domain = Proton
Irr_Freq = 500.15991521[MHz]
Irr_Offset = 5.0[ppm]
Tri_Domain = Proton
Tri_Freq = 500.15991521[MHz]
Tri_Offset = 5.0[ppm]
Clipped = FALSE
Scans = 16
Total_Scans = 16
Relaxation_Delay = 2[s]
Recvr_Gain = 48
Temp_Get = 21.7[dC]
X_90_Width = 11.6[us]
X_Acq_Time = 1.74587904[s]
X_Angle = 45[deg]
X_Atn = 3.6[dB]
X_Pulse = 5.8[us]
Irr_Mode = Off
Tri_Mode = Off
Dante_Presat = FALSE
Initial_Wait = 1[s]
Repetition_Time = 3.74587904[s]
abundance
X : parts per Million : Proton
3.87
1.84
2.19
0.90
3.00
7.356
7.340
7.318
7.304
7.260
7.252
7.015
7.005
6.985
6.969
5.811
5.794
5.790
2.377
1.600

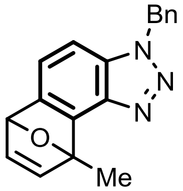

Bn
N
N
N
O
Me

proximal-**3g**

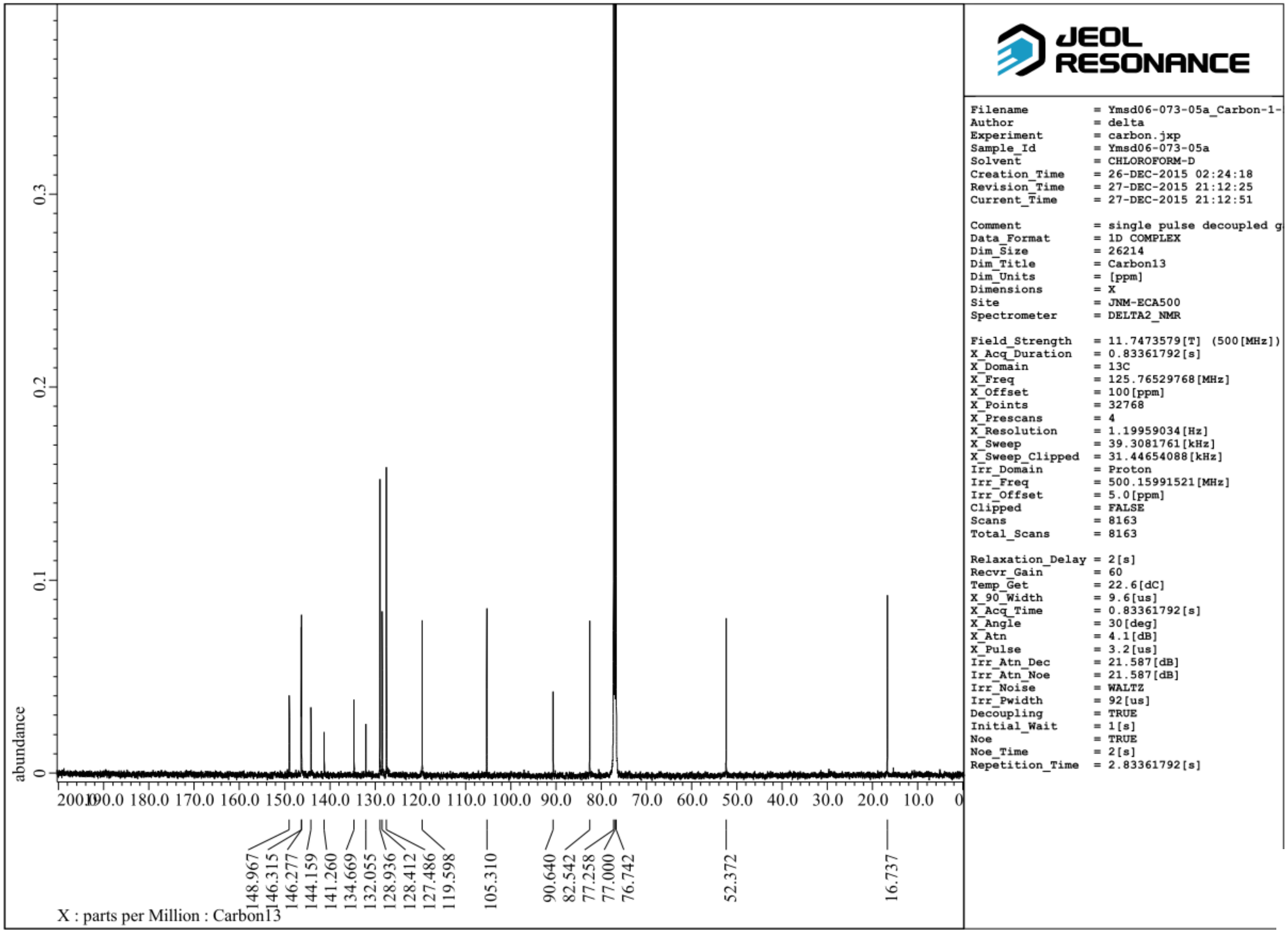

JEOL RESONANCE
Filename = Ymsd06-073-05a_Carbon-1-
Author = delta
Experiment = carbon.jxp
Sample_Id = Ymsd06-073-05a
Solvent = CHLOROFORM-D
Creation_Time = 26-DEC-2015 02:24:18
Revision_Time = 27-DEC-2015 21:12:25
Current_Time = 27-DEC-2015 21:12:51
Comment = single pulse decoupled g
Data_Format = 1D COMPLEX
Dim_Size = 26214
Dim_Title = Carbon13
Dim_Units = [ppm]
Dimensions = X
Site = JNM-ECA500
Spectrometer = DELTA2_NMR
Field_Strength = 11.7473579[T] (500[MHz])
X_Acq_Duration = 0.83361792[s]
X_Domain = 13C
X_Freq = 125.76529768[MHz]
X_Offset = 100[ppm]
X_Points = 32768
X_Prescans = 4
X_Resolution = 1.19959034[Hz]
X_Sweep = 39.3081761[kHz]
X_Sweep_Clipped = 31.44654088[kHz]
Irr_Domain = Proton
Irr_Freq = 500.15991521[MHz]
Irr_Offset = 5.0[ppm]
Clipped = FALSE
Scans = 8163
Total_Scans = 8163
Relaxation_Delay = 2[s]
Recvr_Gain = 60
Temp_Get = 22.6[dC]
X_90_Width = 9.6[us]
X_Acq_Time = 0.83361792[s]
X_Angle = 30[deg]
X_Atn = 4.1[dB]
X_Pulse = 3.2[us]
Irr_Atn_Dec = 21.587[dB]
Irr_Atn_Noe = 21.587[dB]
Irr_Noise = WALTZ
Irr_Pwidth = 92[us]
Decoupling = TRUE
Initial_Wait = 1[s]
Noe = TRUE
Noe_Time = 2[s]
Repetition_Time = 2.83361792[s]
abundance
0.3
0.2
0.1
0
200.0 190.0 180.0 170.0 160.0 150.0 140.0 130.0 120.0 110.0 100.0 90.0 80.0 70.0 60.0 50.0 40.0 30.0 20.0 10.0 0
148.967
146.315
146.277
144.159
141.260
134.669
132.055
128.936
128.412
127.486
119.598
105.310
90.640
82.542
77.258
77.000
76.742
52.372
16.737
X : parts per Million : Carbon13

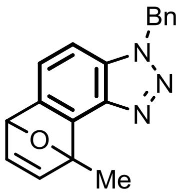

Bn
N
N
N
O
Me

proximal-**3g**

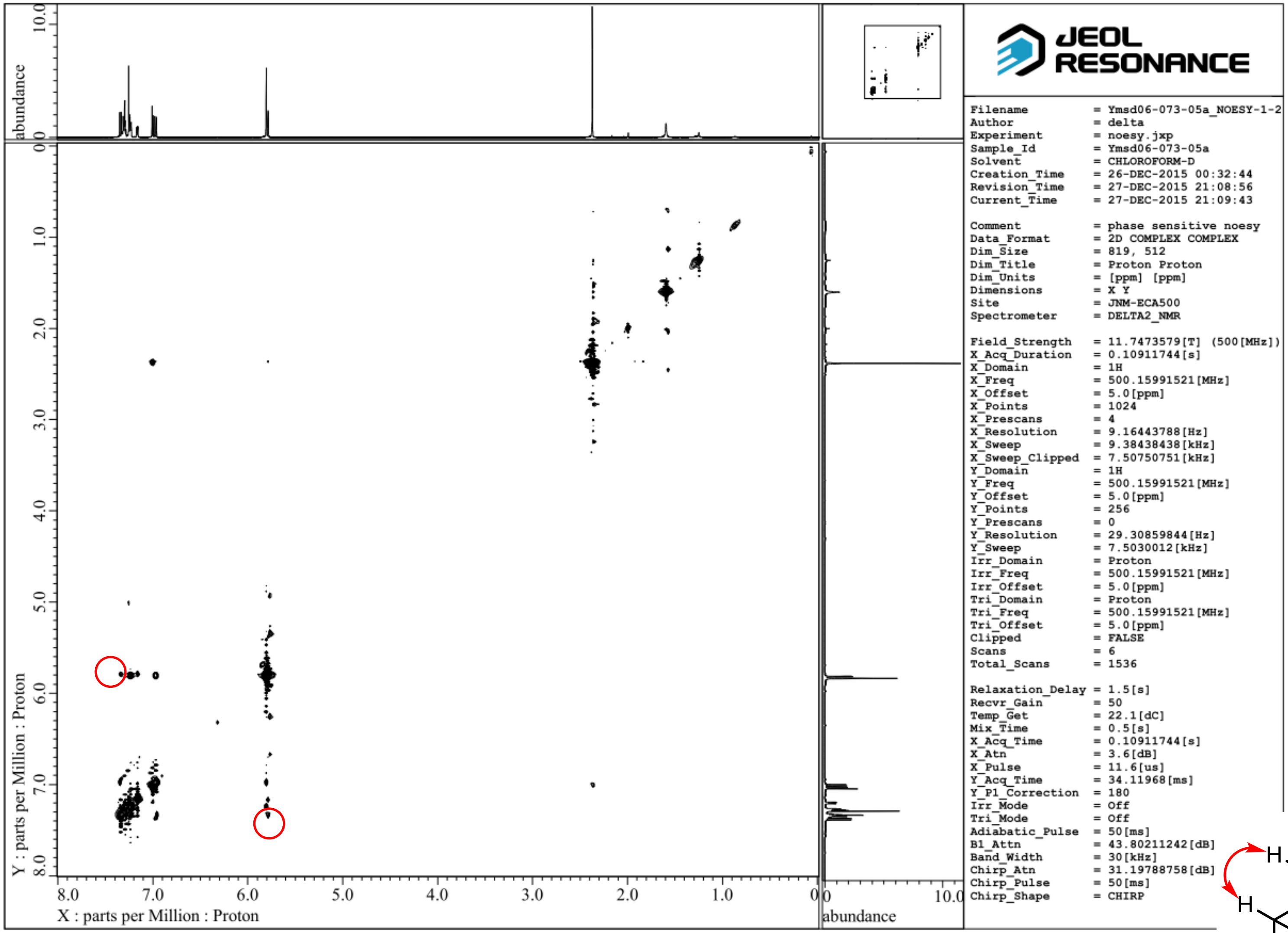
JEOL RESONANCE
abundance
X : parts per Million : Proton
Y : parts per Million : Proton
Filename = Ymsd06-073-05a_NOESY-1-2
Author = delta
Experiment = noesy.jxp
Sample_Id = Ymsd06-073-05a
Solvent = CHLOROFORM-D
Creation_Time = 26-DEC-2015 00:32:44
Revision_Time = 27-DEC-2015 21:08:56
Current_Time = 27-DEC-2015 21:09:43
Comment = phase sensitive noesy
Data_Format = 2D COMPLEX COMPLEX
Dim_Size = 819, 512
Dim_Title = Proton Proton
Dim_Units = [ppm] [ppm]
Dimensions = X Y
Site = JNM-ECA500
Spectrometer = DELTA2_NMR
Field_Strength = 11.7473579[T] (500[MHz])
X_Acq_Duration = 0.10911744[s]
X_Domain = 1H
X_Freq = 500.15991521[MHz]
X_Offset = 5.0[ppm]
X_Points = 1024
X_Prescans = 4
X_Resolution = 9.16443788[Hz]
X_Sweep = 9.38438438[kHz]
X_Sweep_Clipped = 7.50750751[kHz]
Y_Domain = 1H
Y_Freq = 500.15991521[MHz]
Y_Offset = 5.0[ppm]
Y_Points = 256
Y_Prescans = 0
Y_Resolution = 29.30859844[Hz]
Y_Sweep = 7.5030012[kHz]
Irr_Domain = Proton
Irr_Freq = 500.15991521[MHz]
Irr_Offset = 5.0[ppm]
Tri_Domain = Proton
Tri_Freq = 500.15991521[MHz]
Tri_Offset = 5.0[ppm]
Clipped = FALSE
Scans = 6
Total_Scans = 1536
Relaxation_Delay = 1.5[s]
Recvr_Gain = 50
Temp_Get = 22.1[dC]
Mix_Time = 0.5[s]
X_Acq_Time = 0.10911744[s]
X_Atn = 3.6[dB]
X_Pulse = 11.6[us]
Y_Acq_Time = 34.11968[ms]
Y_P1_Correction = 180
Irr_Mode = Off
Tri_Mode = Off
Adiabatic_Pulse = 50[ms]
B1_Attn = 43.80211242[dB]
Band_Width = 30[kHz]
Chirp_Atn = 31.19788758[dB]
Chirp_Pulse = 50[ms]
Chirp_Shape = CHIRP

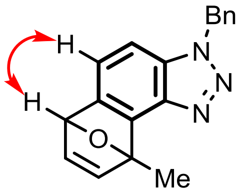
Bn
N
N
N
H
H
O
Me

proximal-**3g**

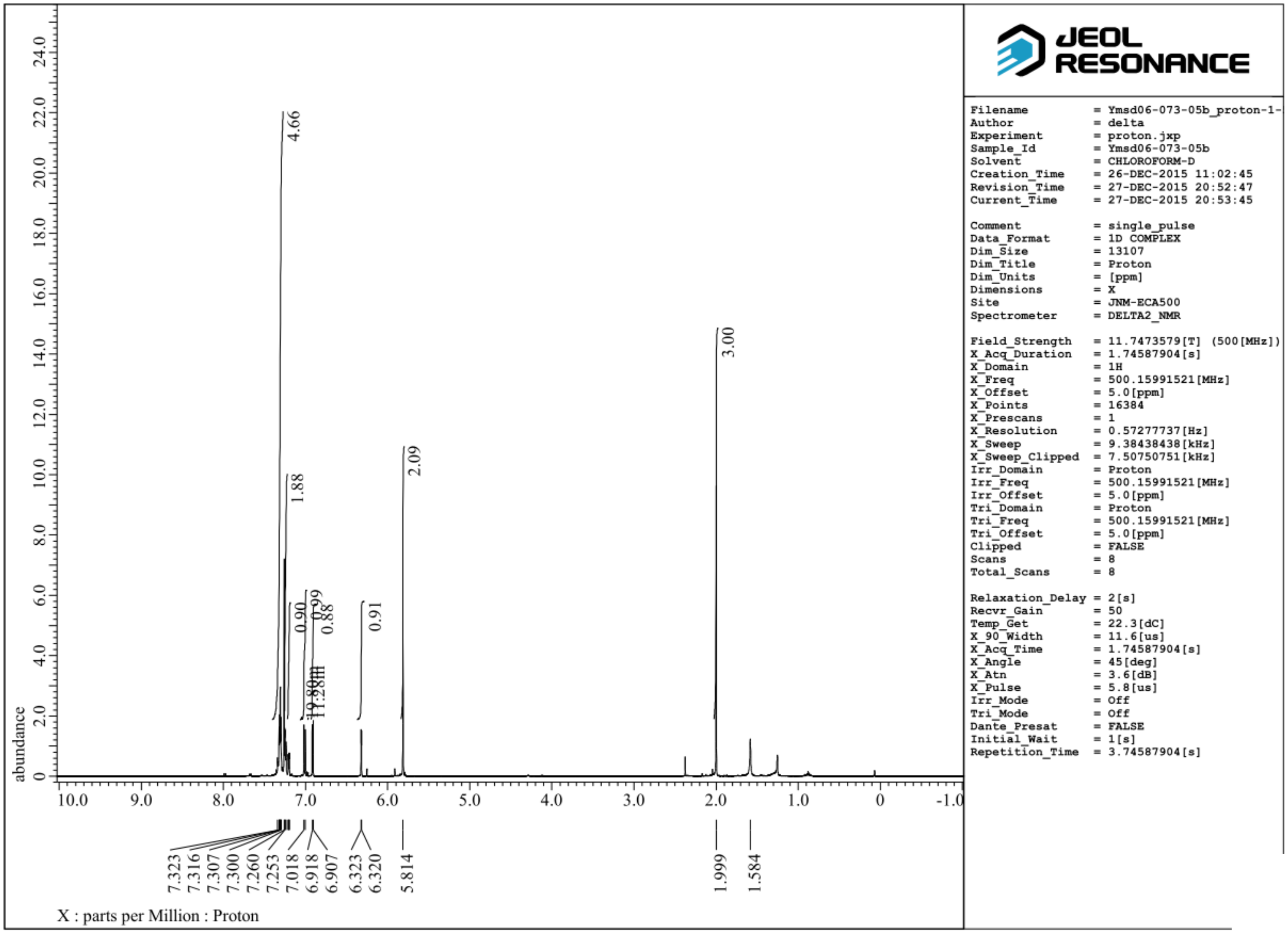
JEOL RESONANCE
Filename = Ymsd06-073-05b_proton-1-
Author = delta
Experiment = proton.jxp
Sample_Id = Ymsd06-073-05b
Solvent = CHLOROFORM-D
Creation_Time = 26-DEC-2015 11:02:45
Revision_Time = 27-DEC-2015 20:52:47
Current_Time = 27-DEC-2015 20:53:45
Comment = single_pulse
Data_Format = 1D COMPLEX
Dim_Size = 13107
Dim_Title = Proton
Dim_Units = [ppm]
Dimensions = X
Site = JNM-ECA500
Spectrometer = DELTA2_NMR
Field_Strength = 11.7473579[T] (500[MHz])
X_Acq_Duration = 1.74587904[s]
X_Domain = 1H
X_Freq = 500.15991521[MHz]
X_Offset = 5.0[ppm]
X_Points = 16384
X_Prescans = 1
X_Resolution = 0.57277737[Hz]
X_Sweep = 9.38438438[kHz]
X_Sweep_Clipped = 7.50750751[kHz]
Irr_Domain = Proton
Irr_Freq = 500.15991521[MHz]
Irr_Offset = 5.0[ppm]
Tri_Domain = Proton
Tri_Freq = 500.15991521[MHz]
Tri_Offset = 5.0[ppm]
Clipped = FALSE
Scans = 8
Total_Scans = 8
Relaxation_Delay = 2[s]
Recvr_Gain = 50
Temp_Get = 22.3[dC]
X_90_Width = 11.6[us]
X_Acq_Time = 1.74587904[s]
X_Angle = 45[deg]
X_Atn = 3.6[dB]
X_Pulse = 5.8[us]
Irr_Mode = Off
Tri_Mode = Off
Dante_Presat = FALSE
Initial_Wait = 1[s]
Repetition_Time = 3.74587904[s]
abundance
0
2.0
4.0
6.0
8.0
10.0
12.0
14.0
16.0
18.0
20.0
22.0
24.0
4.66
1.88
0.90
0.99
0.88
0.91
2.09
3.00
10.0
9.0
8.0
7.0
6.0
5.0
4.0
3.0
2.0
1.0
0
-1.0
7.323
7.316
7.307
7.300
7.260
7.253
7.018
6.918
6.907
6.323
6.320
5.814
1.999
1.584
X : parts per Million : Proton

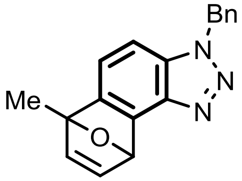
Bn
N
N
N
Me
O

distal-**3g**

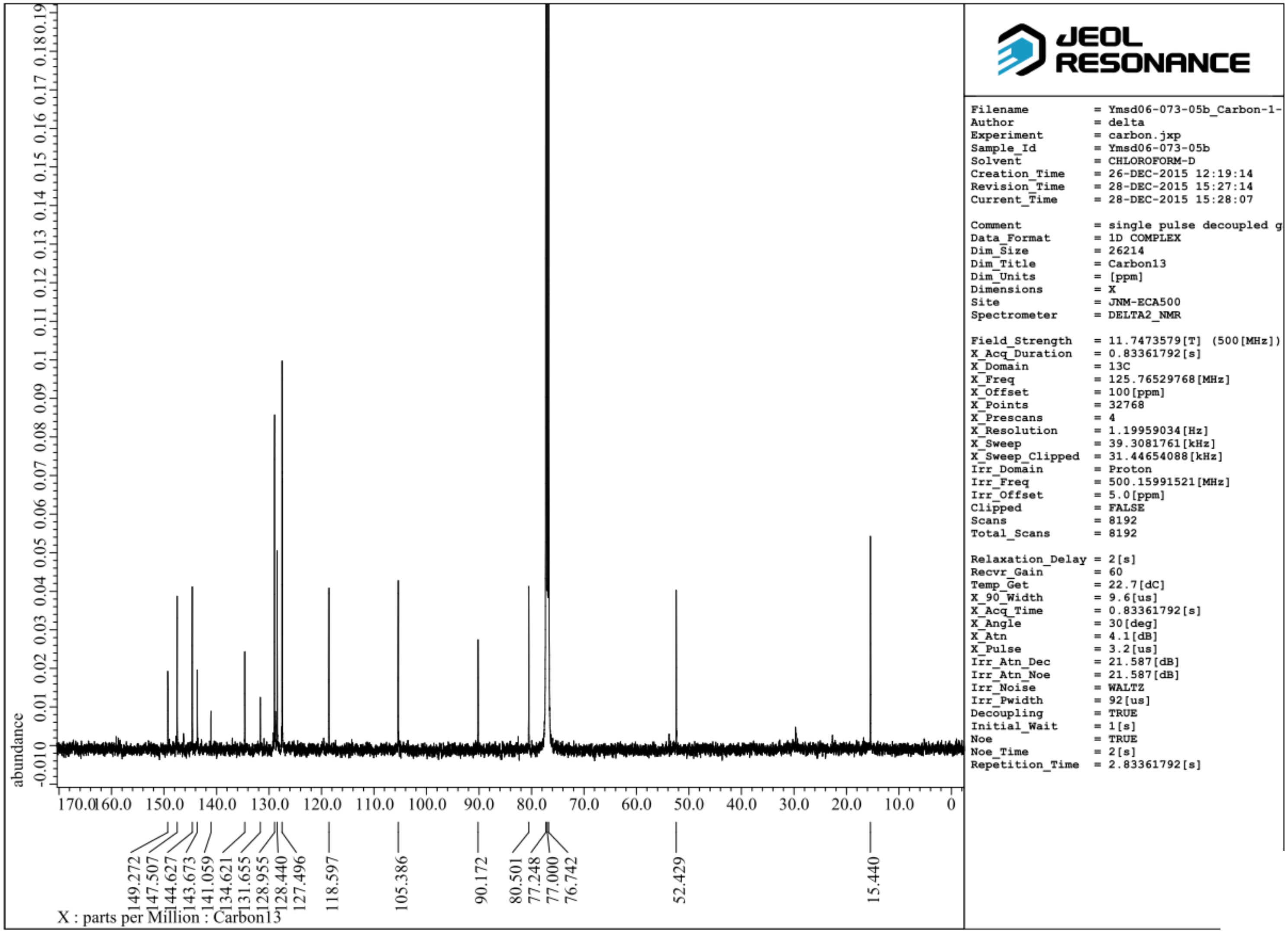

You created this PDF from an application that is not licensed to print to novaPDF printer (http://www.novapdf.com)

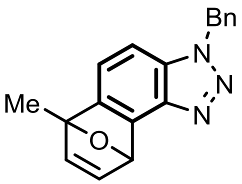

distal-**3g**

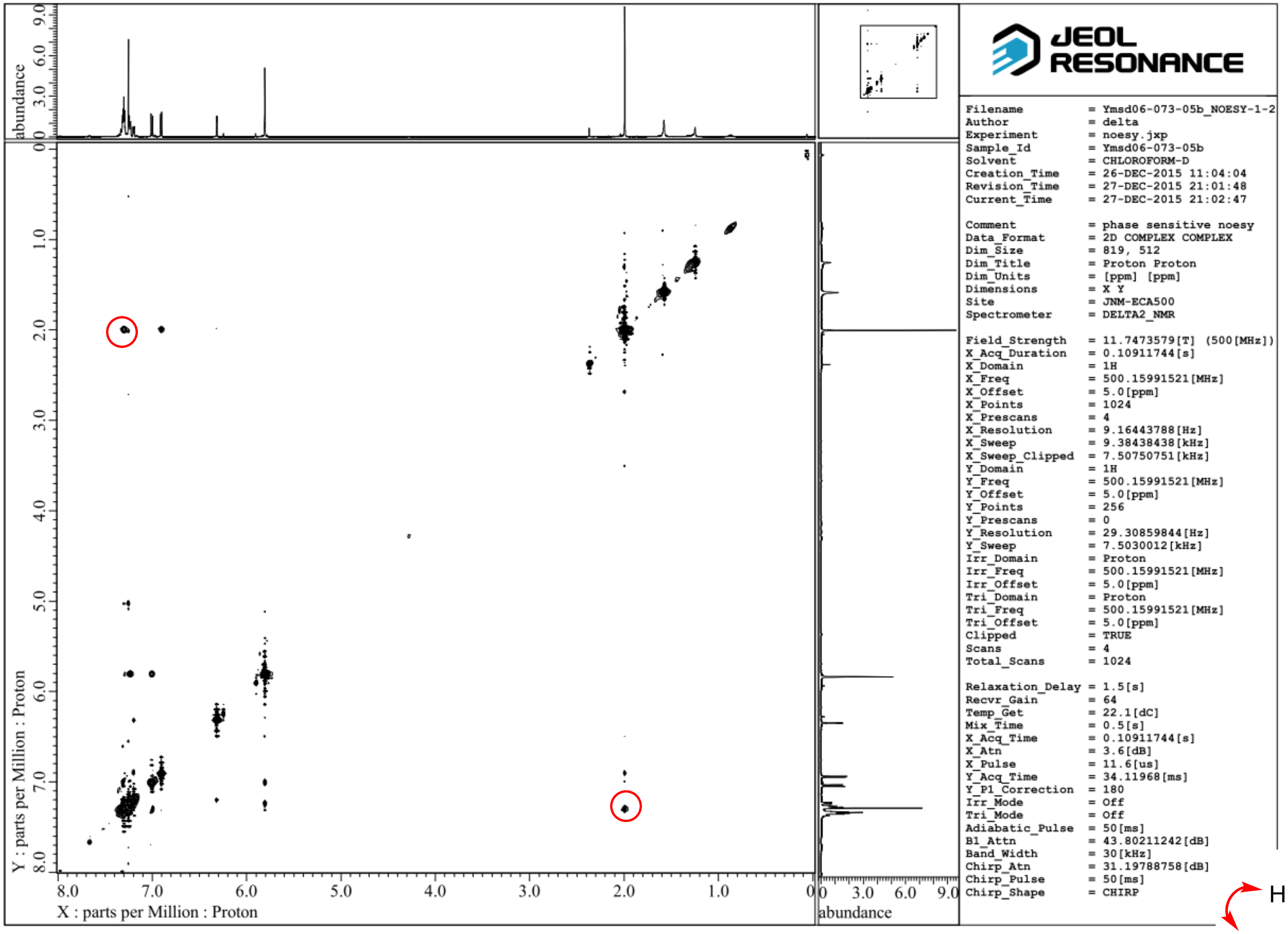
JEOL RESONANCE
Filename = Ymsd06-073-05b_NOESY-1-2
Author = delta
Experiment = noesy.jxp
Sample_Id = Ymsd06-073-05b
Solvent = CHLOROFORM-D
Creation_Time = 26-DEC-2015 11:04:04
Revision_Time = 27-DEC-2015 21:01:48
Current_Time = 27-DEC-2015 21:02:47
Comment = phase sensitive noesy
Data_Format = 2D COMPLEX COMPLEX
Dim_Size = 819, 512
Dim_Title = Proton Proton
Dim_Units = [ppm] [ppm]
Dimensions = X Y
Site = JNM-ECA500
Spectrometer = DELTA2_NMR
Field_Strength = 11.7473579[T] (500[MHz])
X_Acq_Duration = 0.10911744[s]
X_Domain = 1H
X_Freq = 500.15991521[MHz]
X_Offset = 5.0[ppm]
X_Points = 1024
X_Prescans = 4
X_Resolution = 9.16443788[Hz]
X_Sweep = 9.38438438[kHz]
X_Sweep_Clipped = 7.50750751[kHz]
Y_Domain = 1H
Y_Freq = 500.15991521[MHz]
Y_Offset = 5.0[ppm]
Y_Points = 256
Y_Prescans = 0
Y_Resolution = 29.30859844[Hz]
Y_Sweep = 7.5030012[kHz]
Irr_Domain = Proton
Irr_Freq = 500.15991521[MHz]
Irr_Offset = 5.0[ppm]
Tri_Domain = Proton
Tri_Freq = 500.15991521[MHz]
Tri_Offset = 5.0[ppm]
Clipped = TRUE
Scans = 4
Total_Scans = 1024
Relaxation_Delay = 1.5[s]
Recvr_Gain = 64
Temp_Get = 22.1[dC]
Mix_Time = 0.5[s]
X_Acq_Time = 0.10911744[s]
X_Atn = 3.6[dB]
X_Pulse = 11.6[us]
Y_Acq_Time = 34.11968[ms]
Y_P1_Correction = 180
Irr_Mode = Off
Tri_Mode = Off
Adiabatic_Pulse = 50[ms]
B1_Attn = 43.80211242[dB]
Band_Width = 30[kHz]
Chirp_Atn = 31.19788758[dB]
Chirp_Pulse = 50[ms]
Chirp_Shape = CHIRP
abundance
X : parts per Million : Proton
Y : parts per Million : Proton

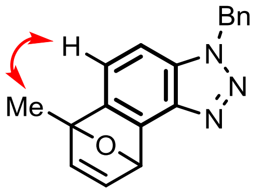
H
Me
O
N
N
N
Bn

distal-**3g**

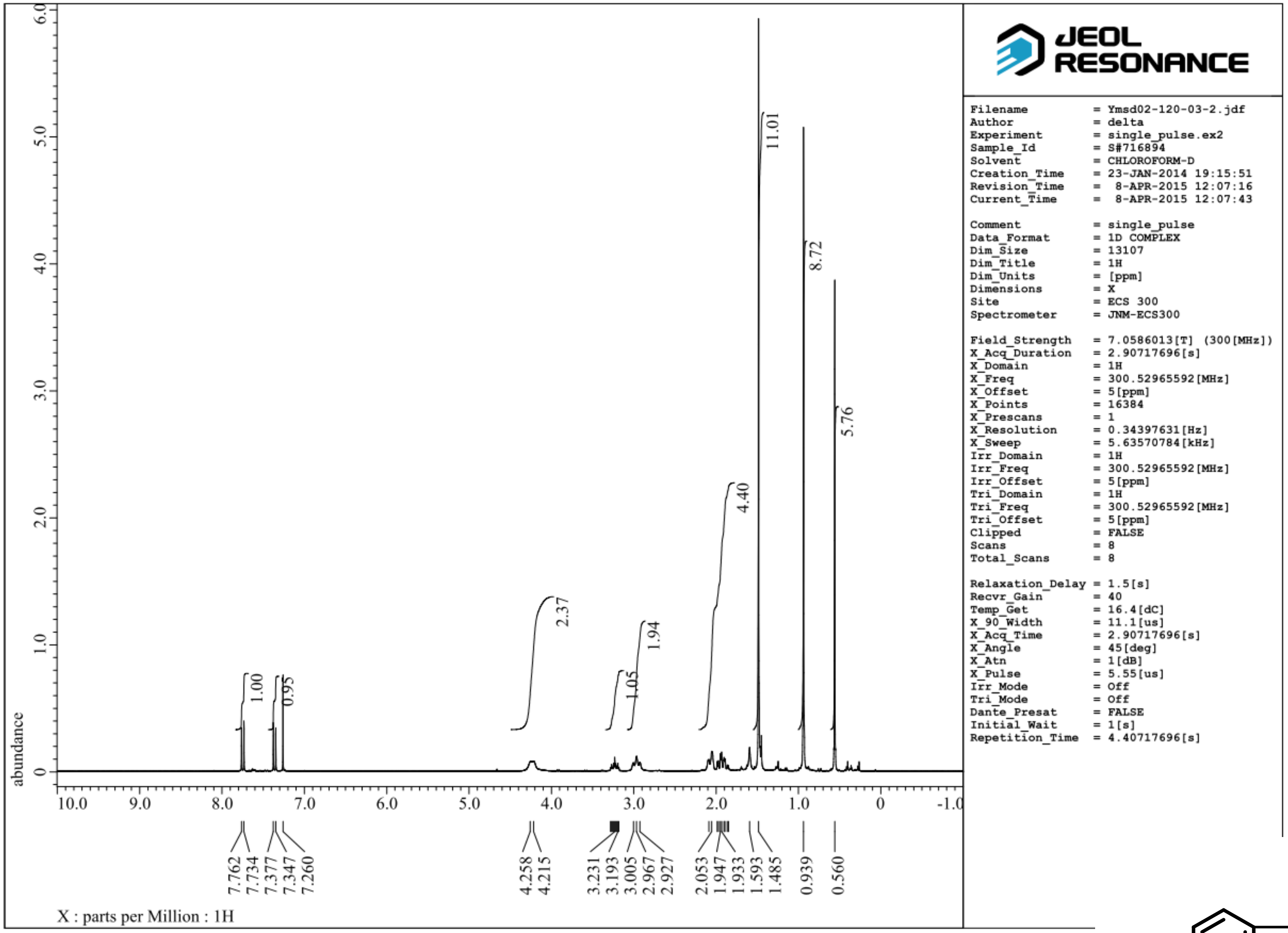

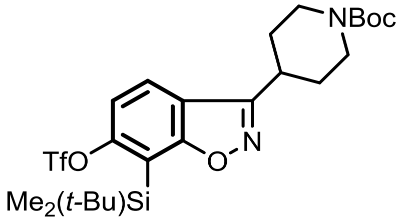

distal-**10f**

You created this PDF from an application that is not licensed to print to novaPDF printer (http://www.novapdf.com)

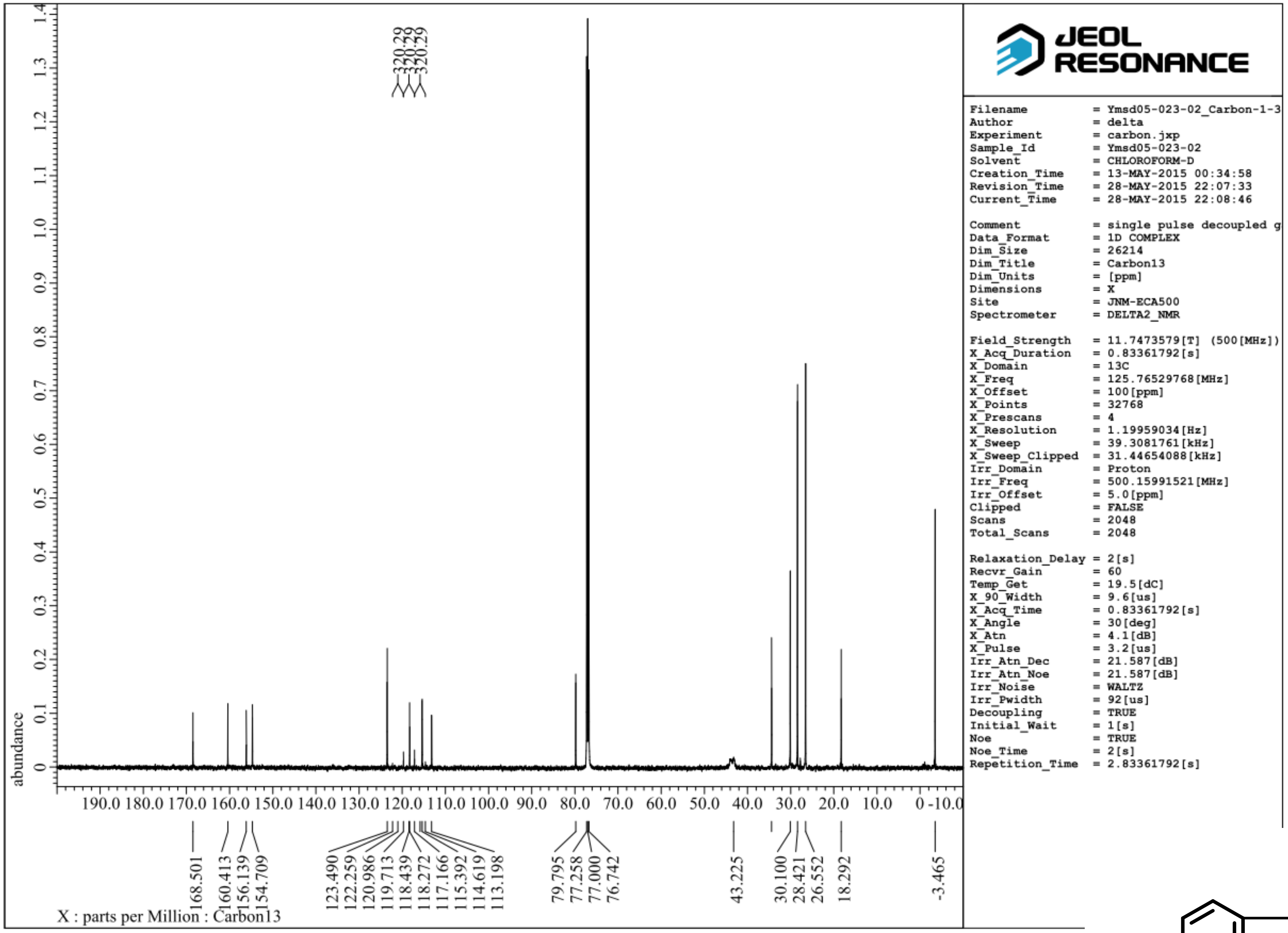

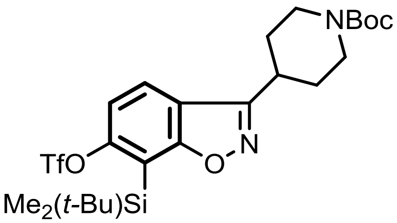

distal-**10f**

You created this PDF from an application that is not licensed to print to novaPDF printer (http://www.novapdf.com)

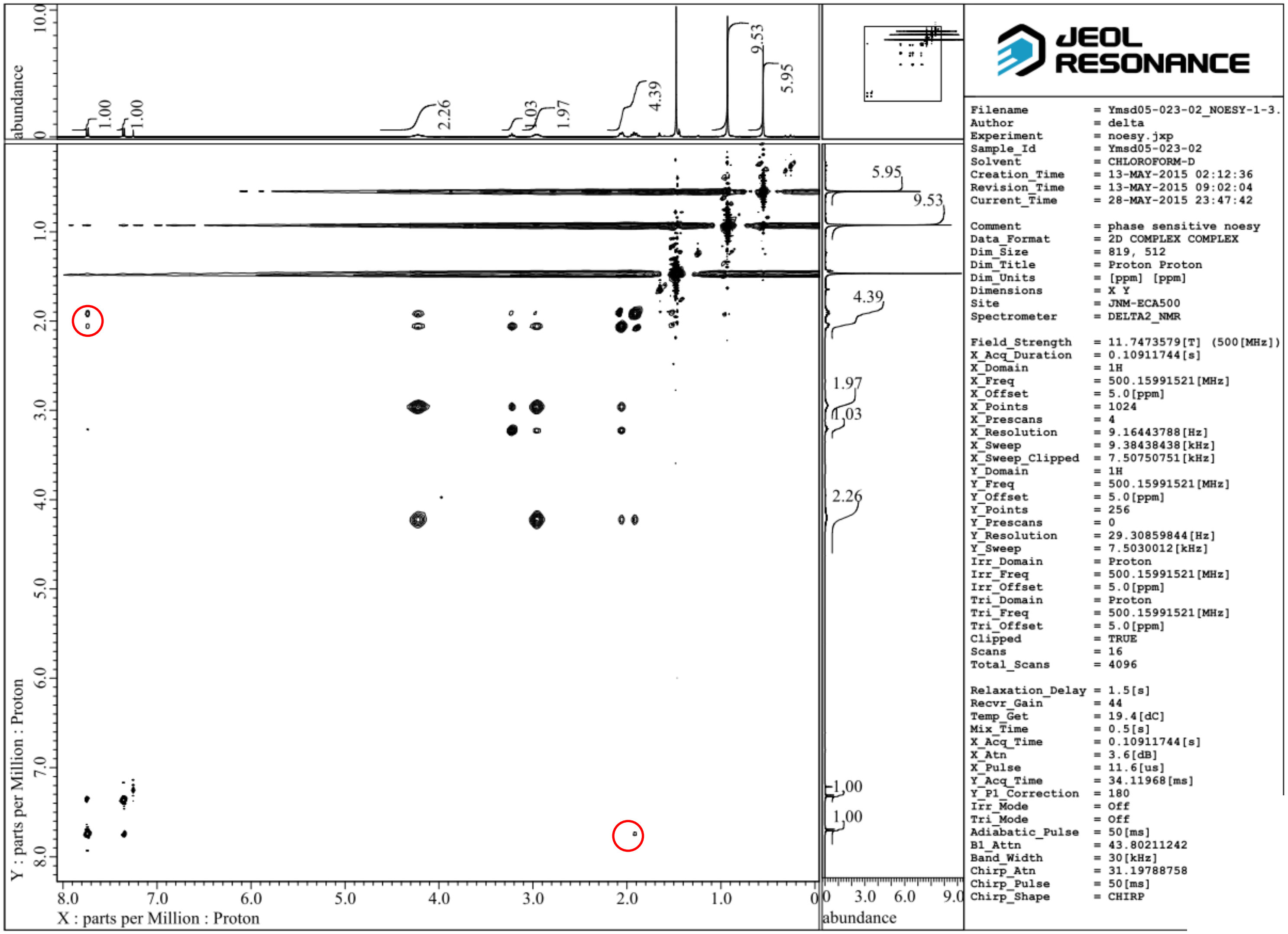

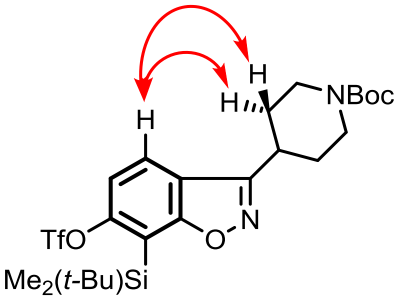

distal-**10f**

You created this PDF from an application that is not licensed to print to novaPDF printer (http://www.novapdf.com)

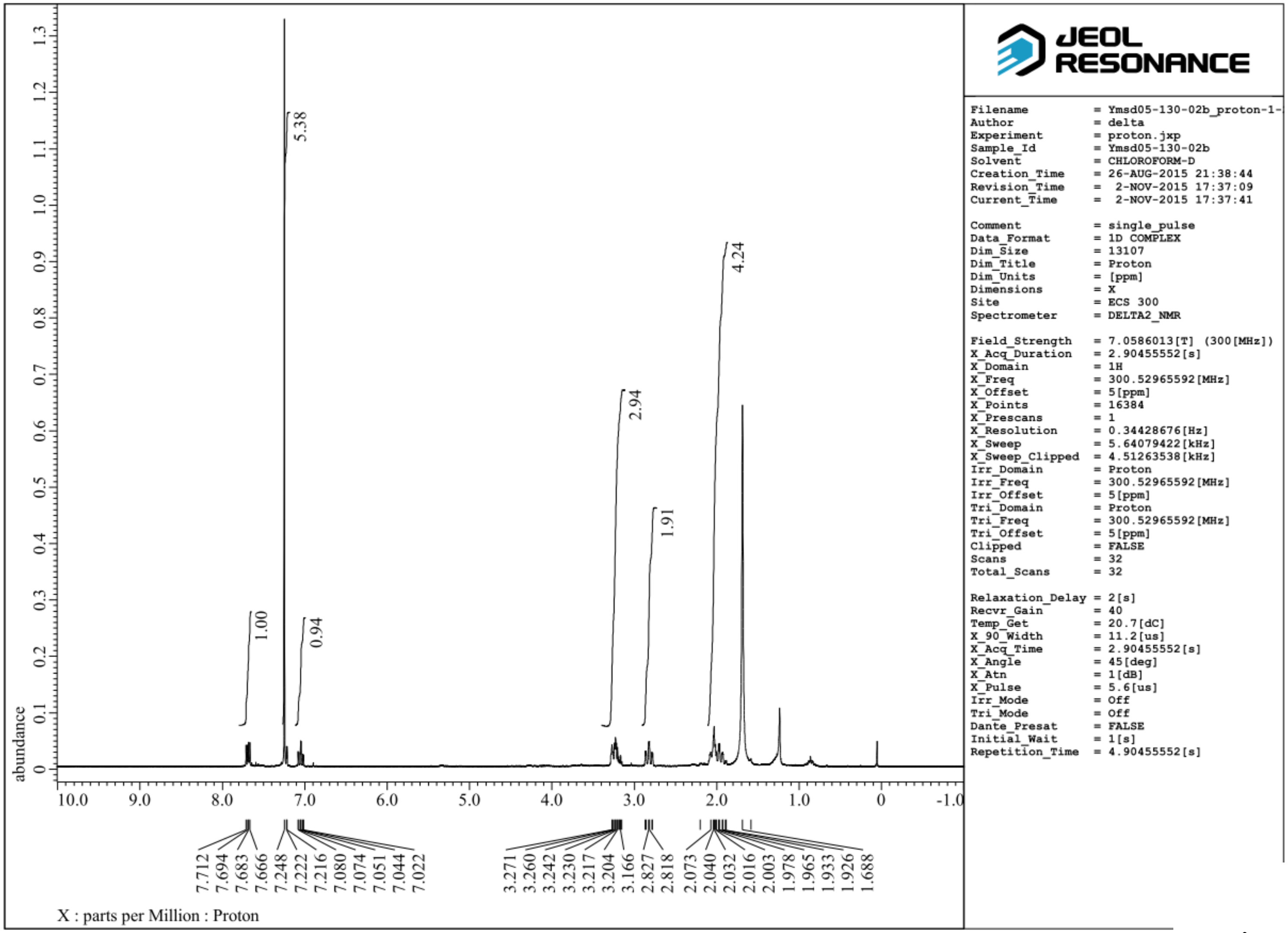

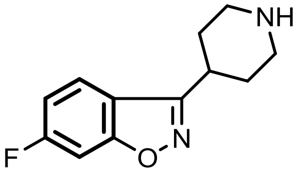

12

You created this PDF from an application that is not licensed to print to novaPDF printer (http://www.novapdf.com)

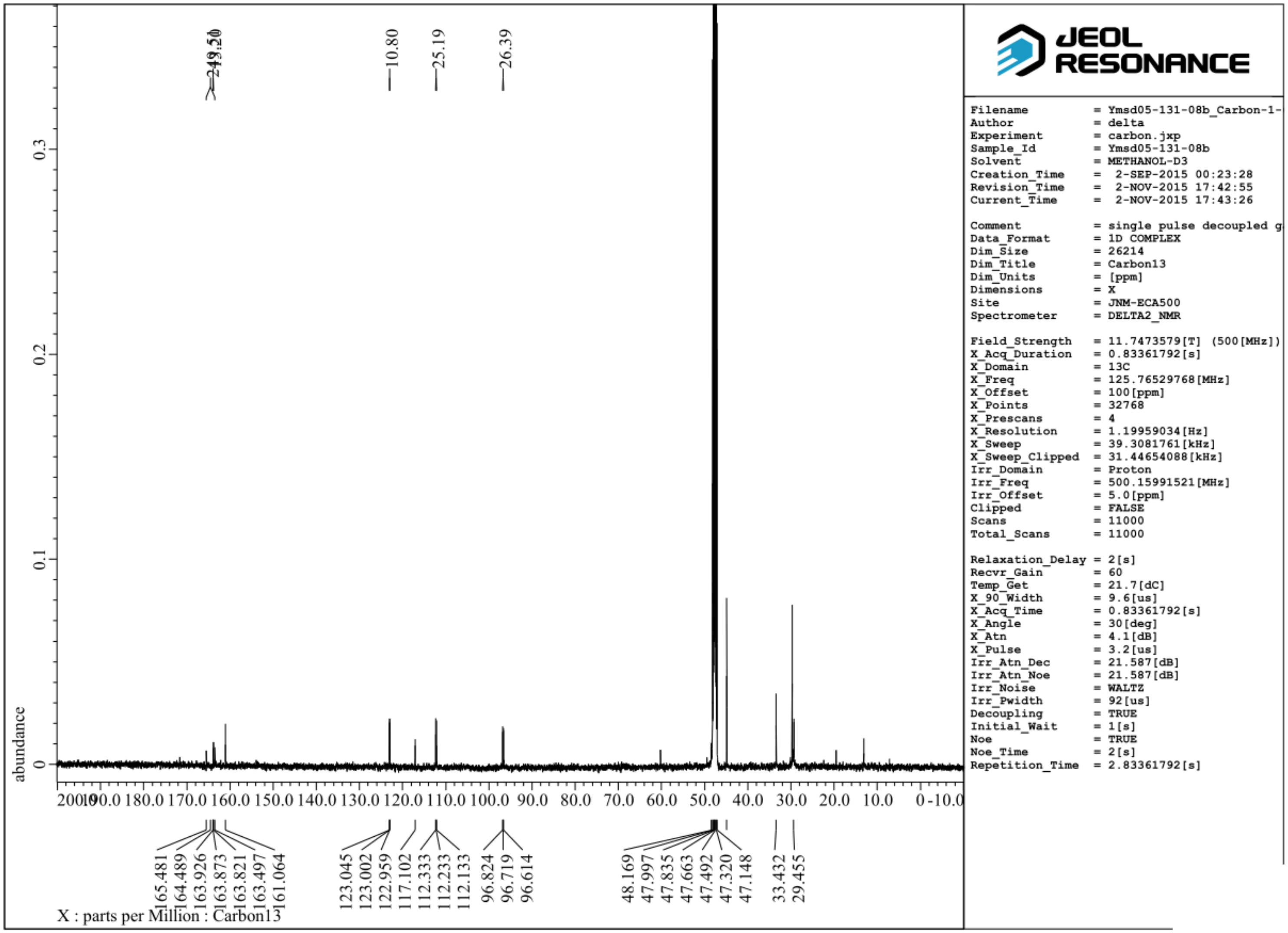

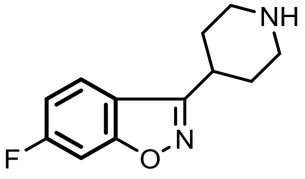

12

You created this PDF from an application that is not licensed to print to novaPDF printer (http://www.novapdf.com)

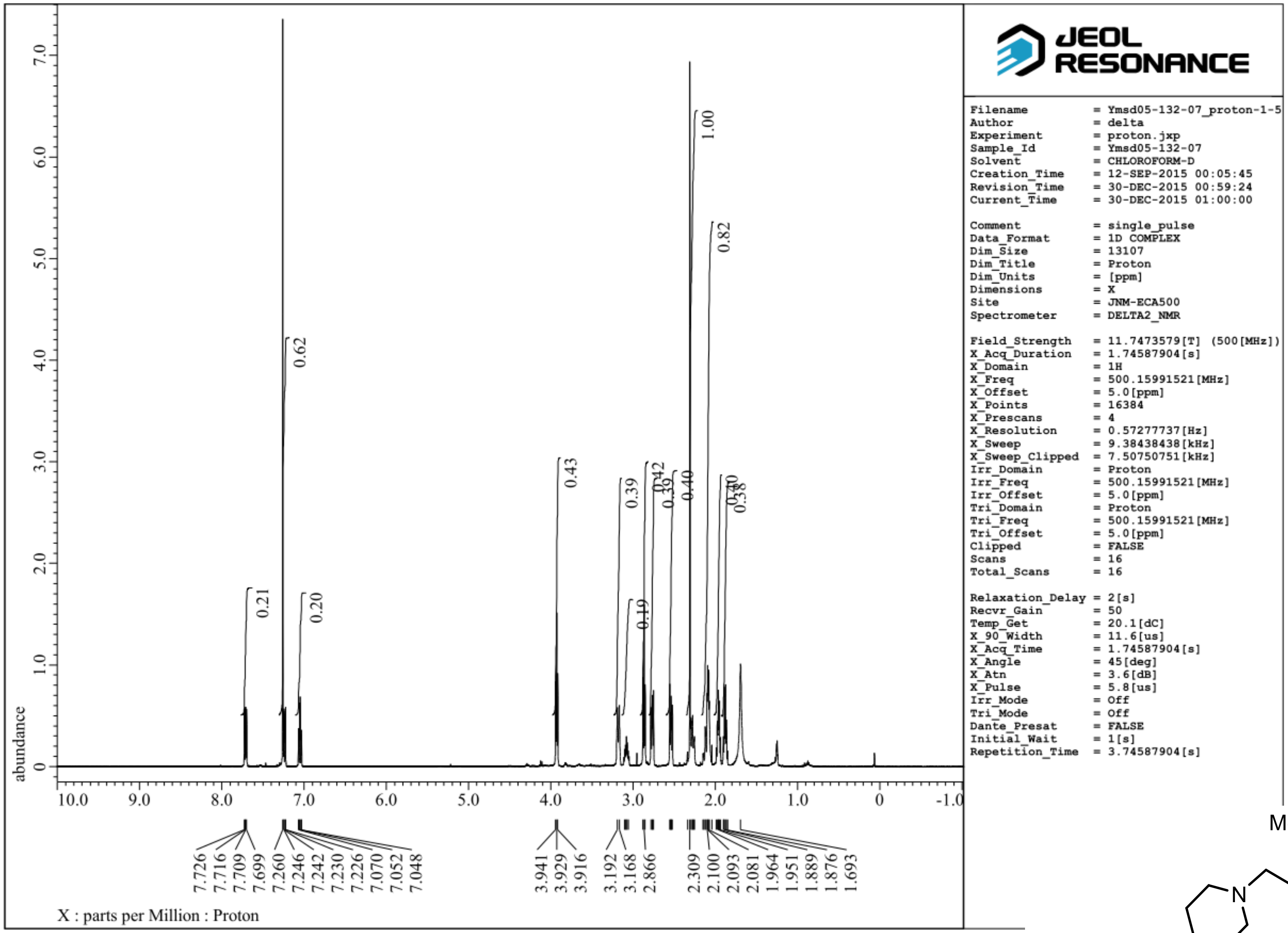

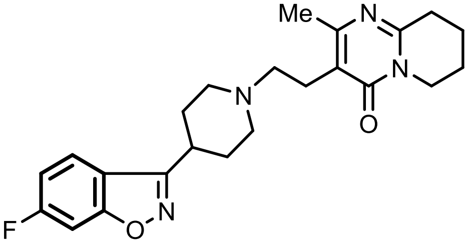

14

You created this PDF from an application that is not licensed to print to novaPDF printer (http://www.novapdf.com)

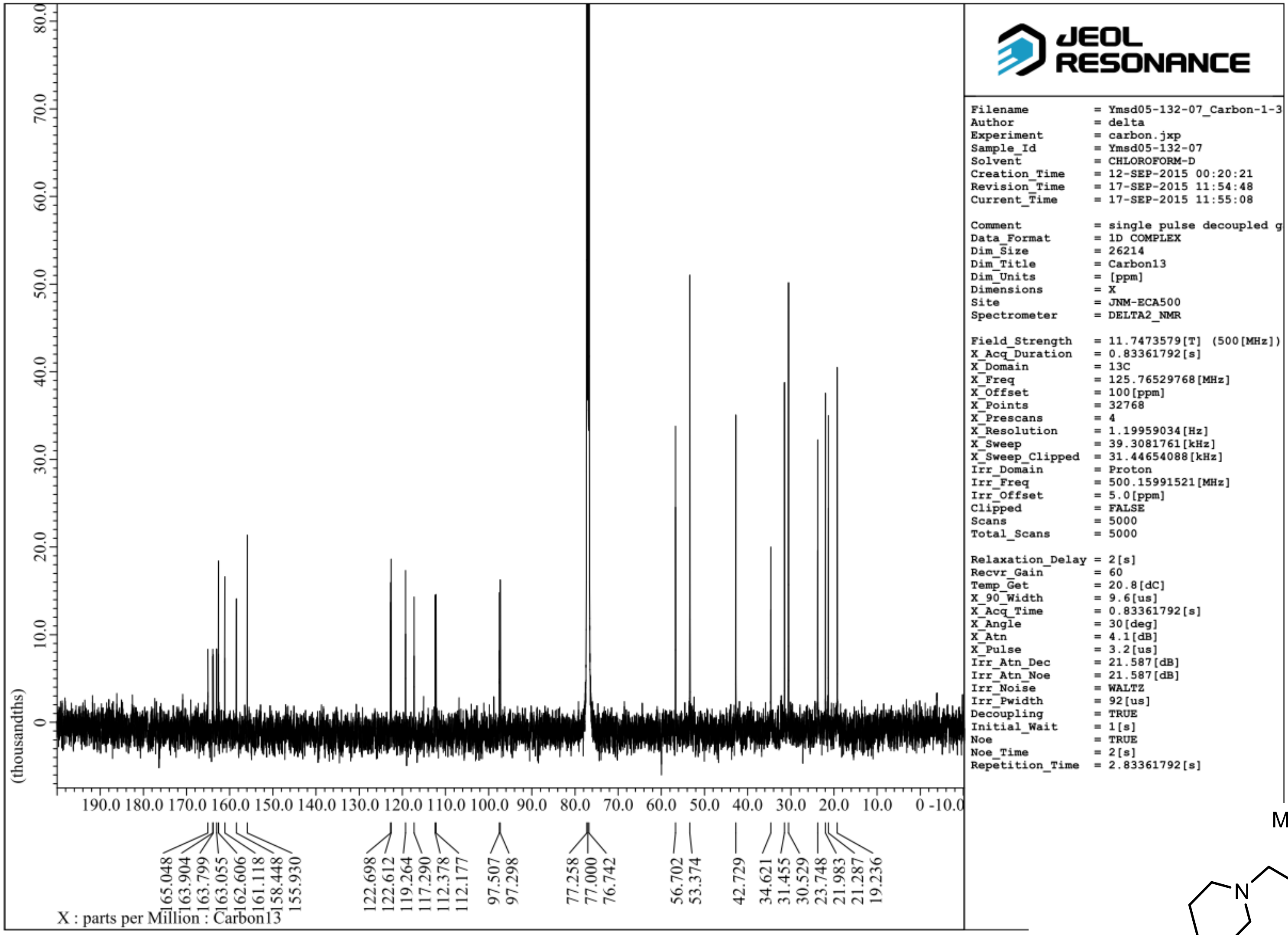
JEOL RESONANCE
Filename = Ymsd05-132-07_Carbon-1-3
Author = delta
Experiment = carbon.jxp
Sample_Id = Ymsd05-132-07
Solvent = CHLOROFORM-D
Creation_Time = 12-SEP-2015 00:20:21
Revision_Time = 17-SEP-2015 11:54:48
Current_Time = 17-SEP-2015 11:55:08
Comment = single pulse decoupled g
Data_Format = 1D COMPLEX
Dim_Size = 26214
Dim_Title = Carbon13
Dim_Units = [ppm]
Dimensions = X
Site = JNM-ECA500
Spectrometer = DELTA2_NMR
Field_Strength = 11.7473579[T] (500[MHz])
X_Acq_Duration = 0.83361792[s]
X_Domain = 13C
X_Freq = 125.76529768[MHz]
X_Offset = 100[ppm]
X_Points = 32768
X_Prescans = 4
X_Resolution = 1.19959034[Hz]
X_Sweep = 39.3081761[kHz]
X_Sweep_Clipped = 31.44654088[kHz]
Irr_Domain = Proton
Irr_Freq = 500.15991521[MHz]
Irr_Offset = 5.0[ppm]
Clipped = FALSE
Scans = 5000
Total_Scans = 5000
Relaxation_Delay = 2[s]
Recvr_Gain = 60
Temp_Get = 20.8[dC]
X_90_Width = 9.6[us]
X_Acq_Time = 0.83361792[s]
X_Angle = 30[deg]
X_Atn = 4.1[dB]
X_Pulse = 3.2[us]
Irr_Atn_Dec = 21.587[dB]
Irr_Atn_Noe = 21.587[dB]
Irr_Noise = WALTZ
Irr_Pwidth = 92[us]
Decoupling = TRUE
Initial_Wait = 1[s]
Noe = TRUE
Noe_Time = 2[s]
Repetition_Time = 2.83361792[s]
(thousandths)
80.0 70.0 60.0 50.0 40.0 30.0 20.0 10.0 0
190.0 180.0 170.0 160.0 150.0 140.0 130.0 120.0 110.0 100.0 90.0 80.0 70.0 60.0 50.0 40.0 30.0 20.0 10.0 0 -10.0
165.048 163.904 163.799 163.055 162.606 161.118 158.448 155.930
122.698 122.612 119.264 117.290 112.378 112.177
97.507 97.298
77.258 77.000 76.742
56.702 53.374
42.729 34.621 31.455 30.529 23.748 21.983 21.287 19.236
X : parts per Million : Carbon13

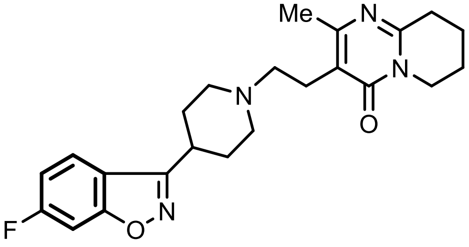
Me
N
N
O
N
F
O
N

14

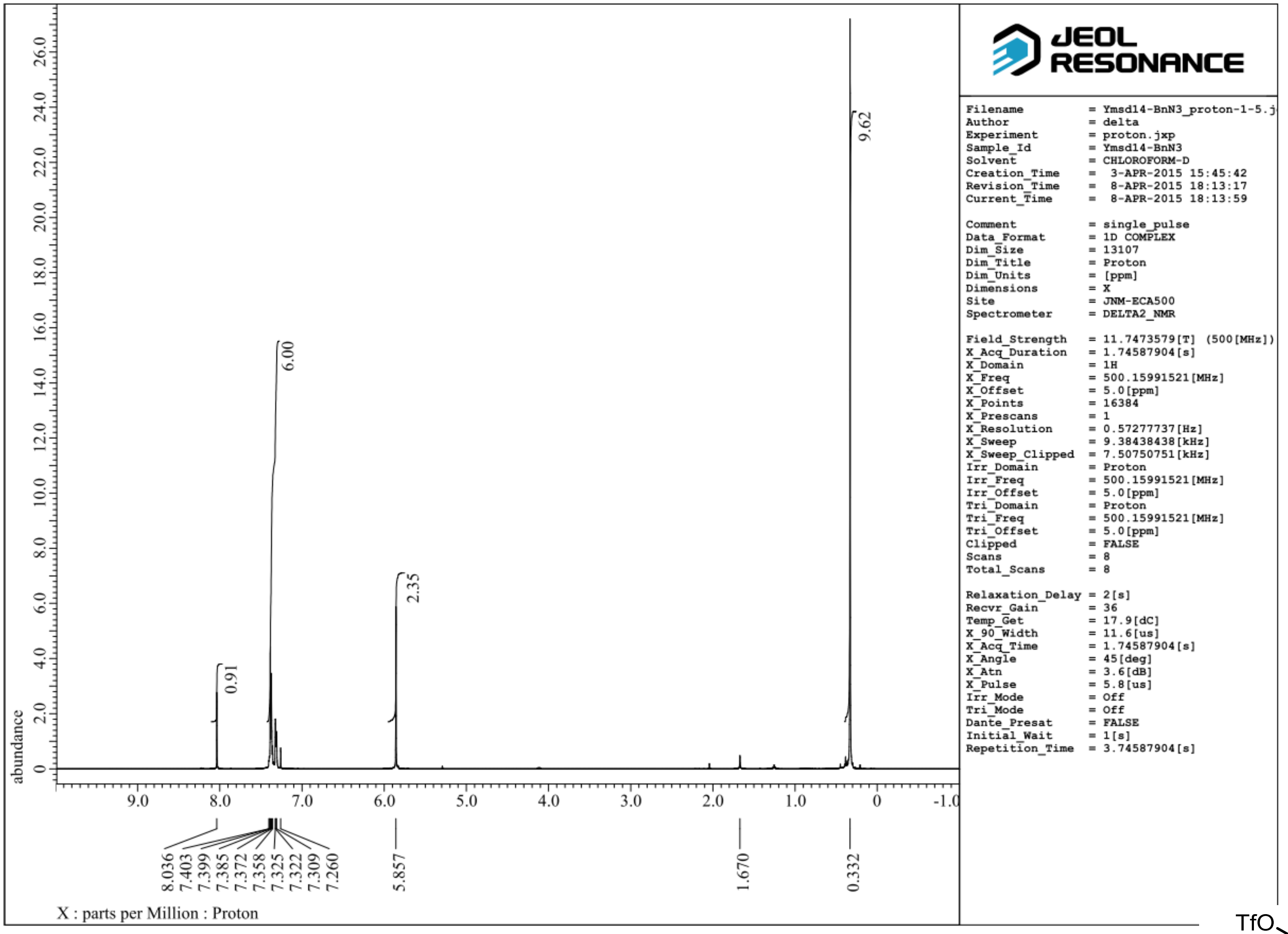

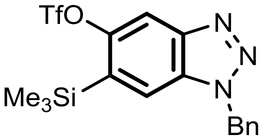

proximal-**17a**

You created this PDF from an application that is not licensed to print to novaPDF printer (http://www.novapdf.com)

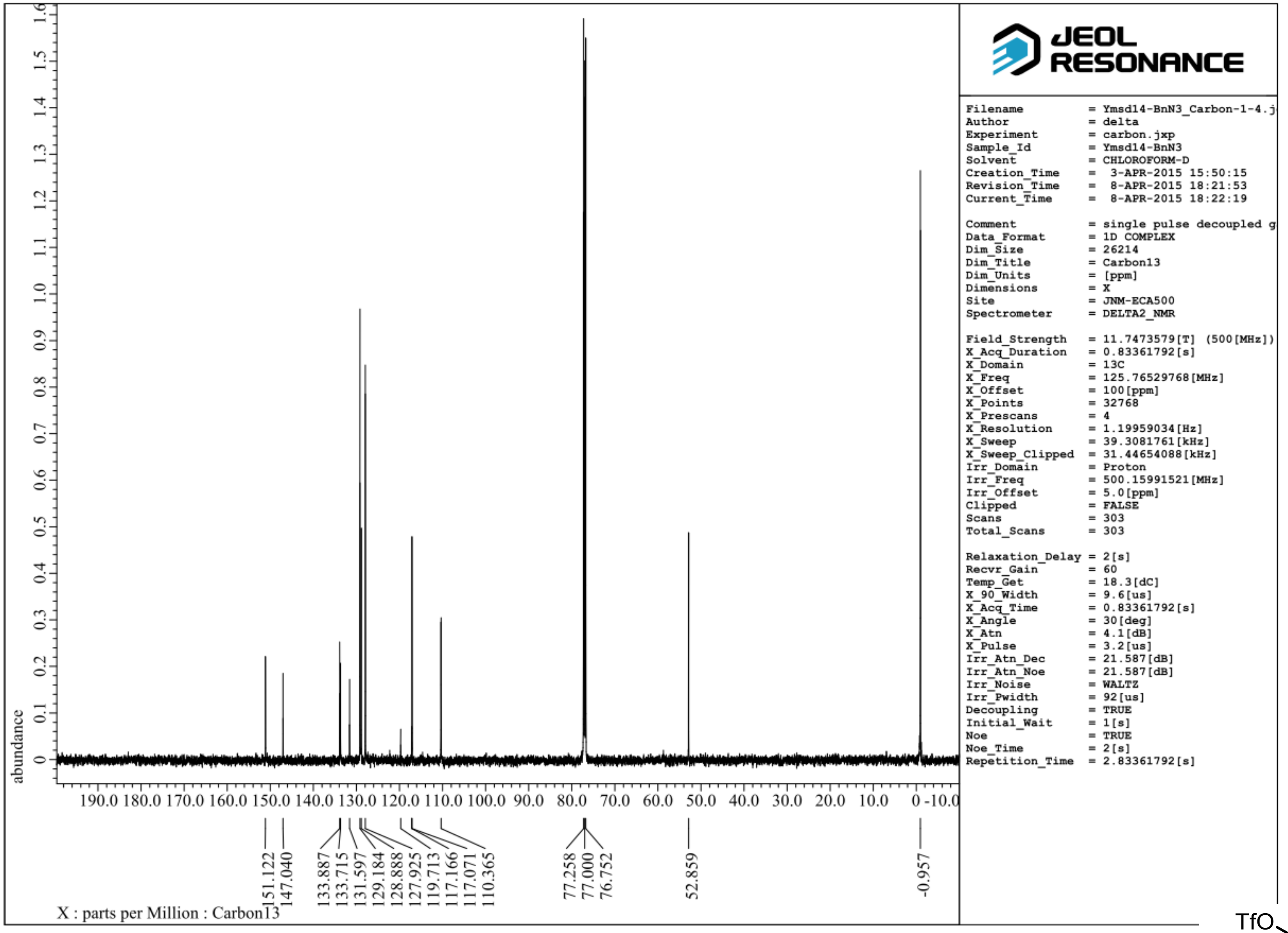

You created this PDF from an application that is not licensed to print to novaPDF printer (http://www.novapdf.com)

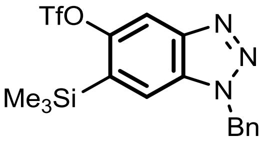

proximal-**17a**

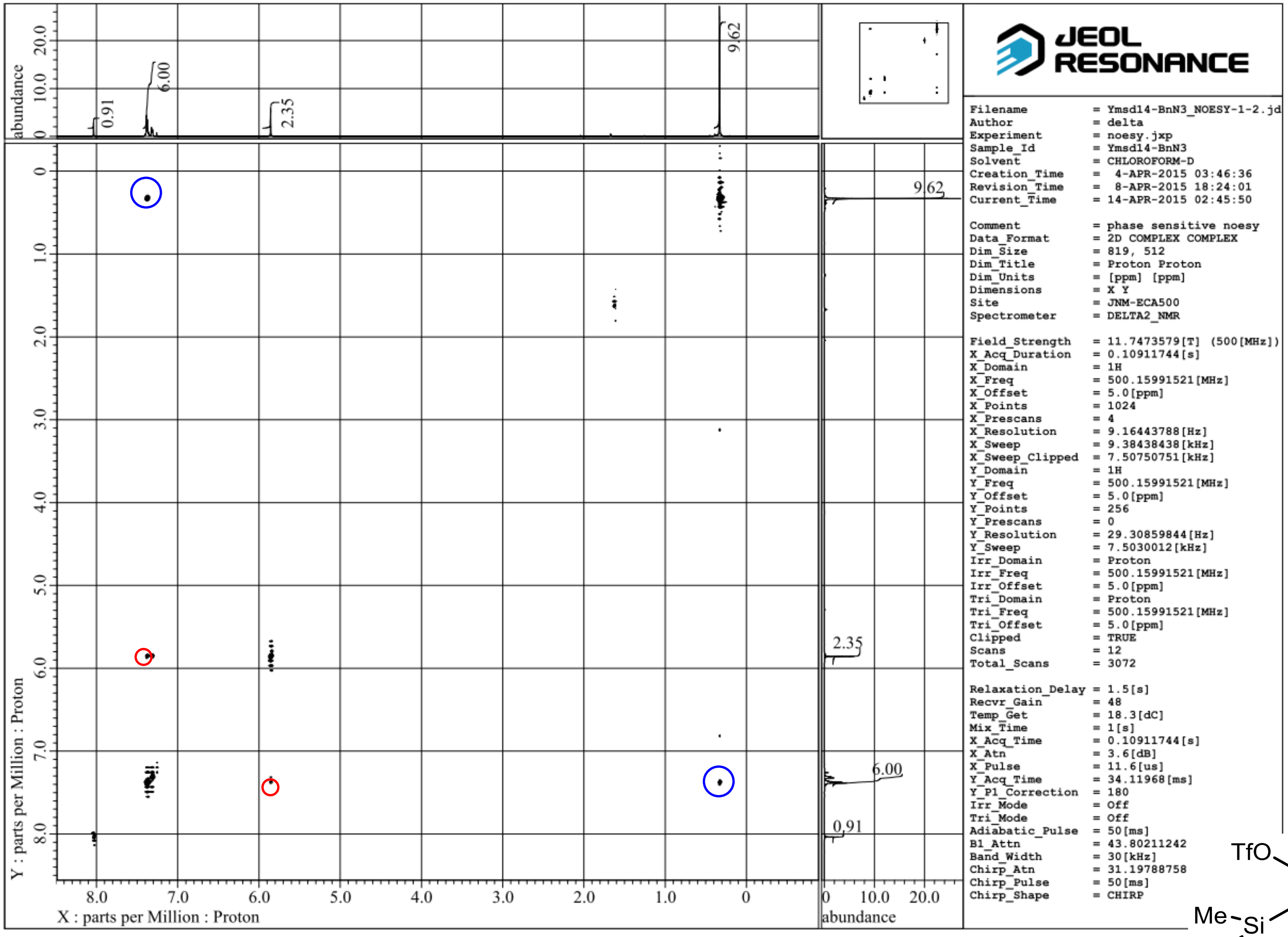

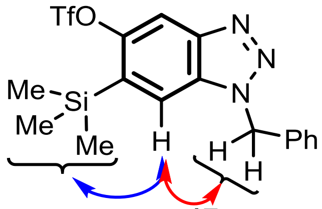

proximal-**17a**

You created this PDF from an application that is not licensed to print to novaPDF printer (http://www.novapdf.com)

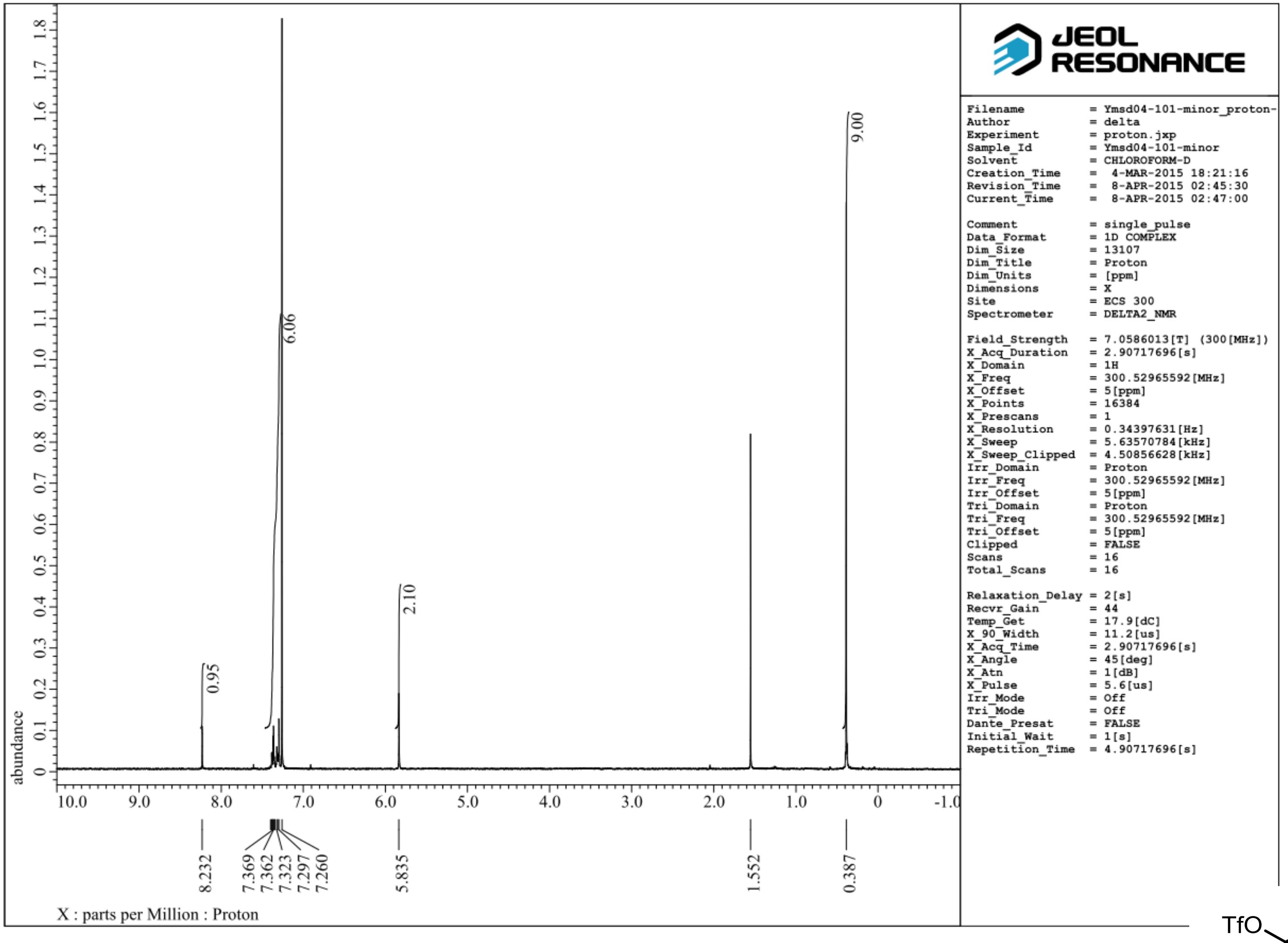

You created this PDF from an application that is not licensed to print to novaPDF printer (http://www.novapdf.com)

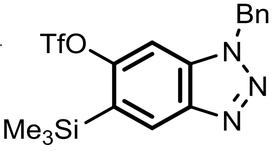

distal-**17a**

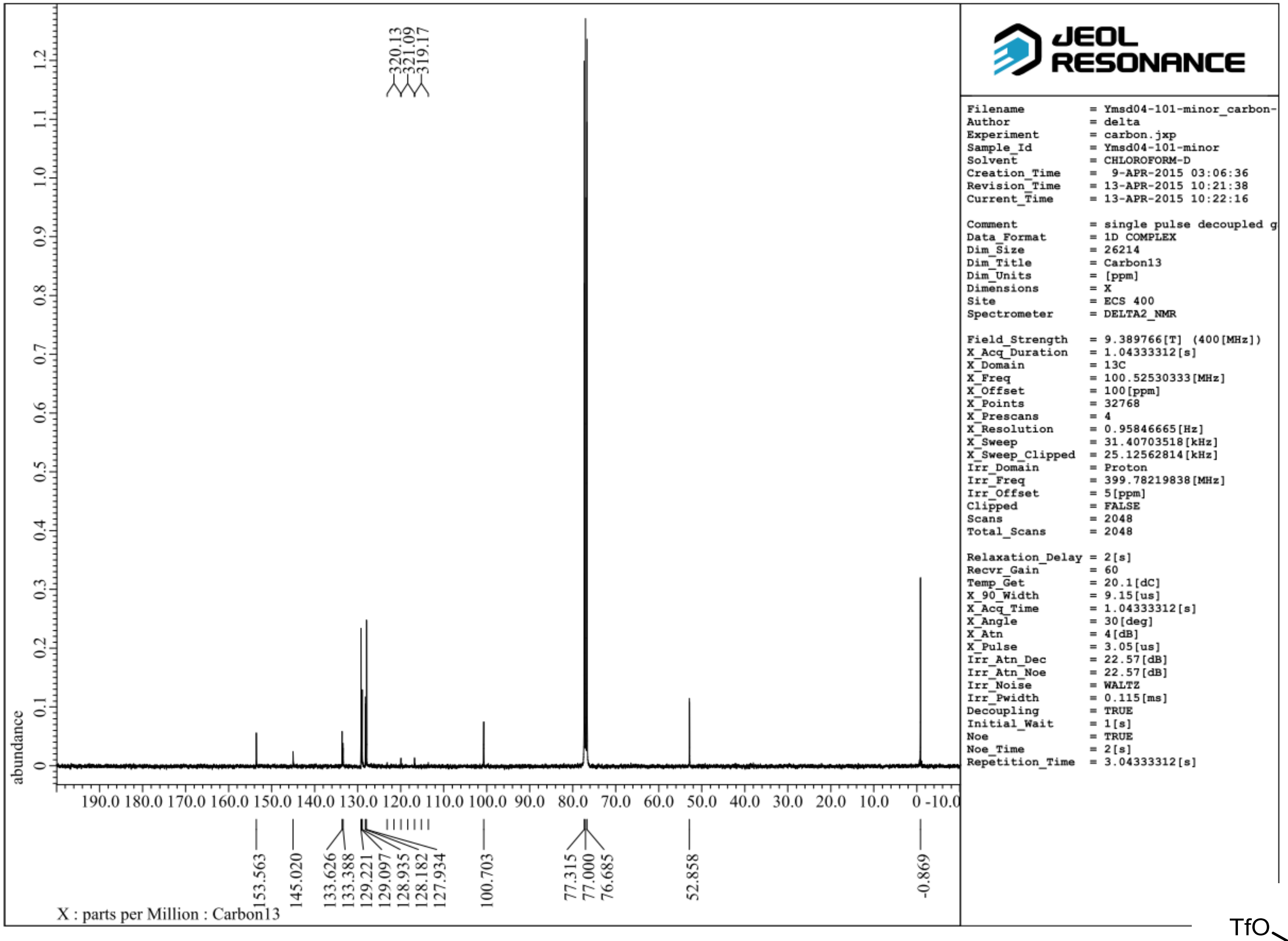

You created this PDF from an application that is not licensed to print to novaPDF printer (http://www.novapdf.com)

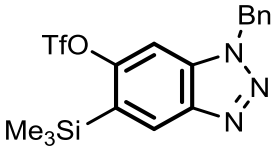

distal-**17a**

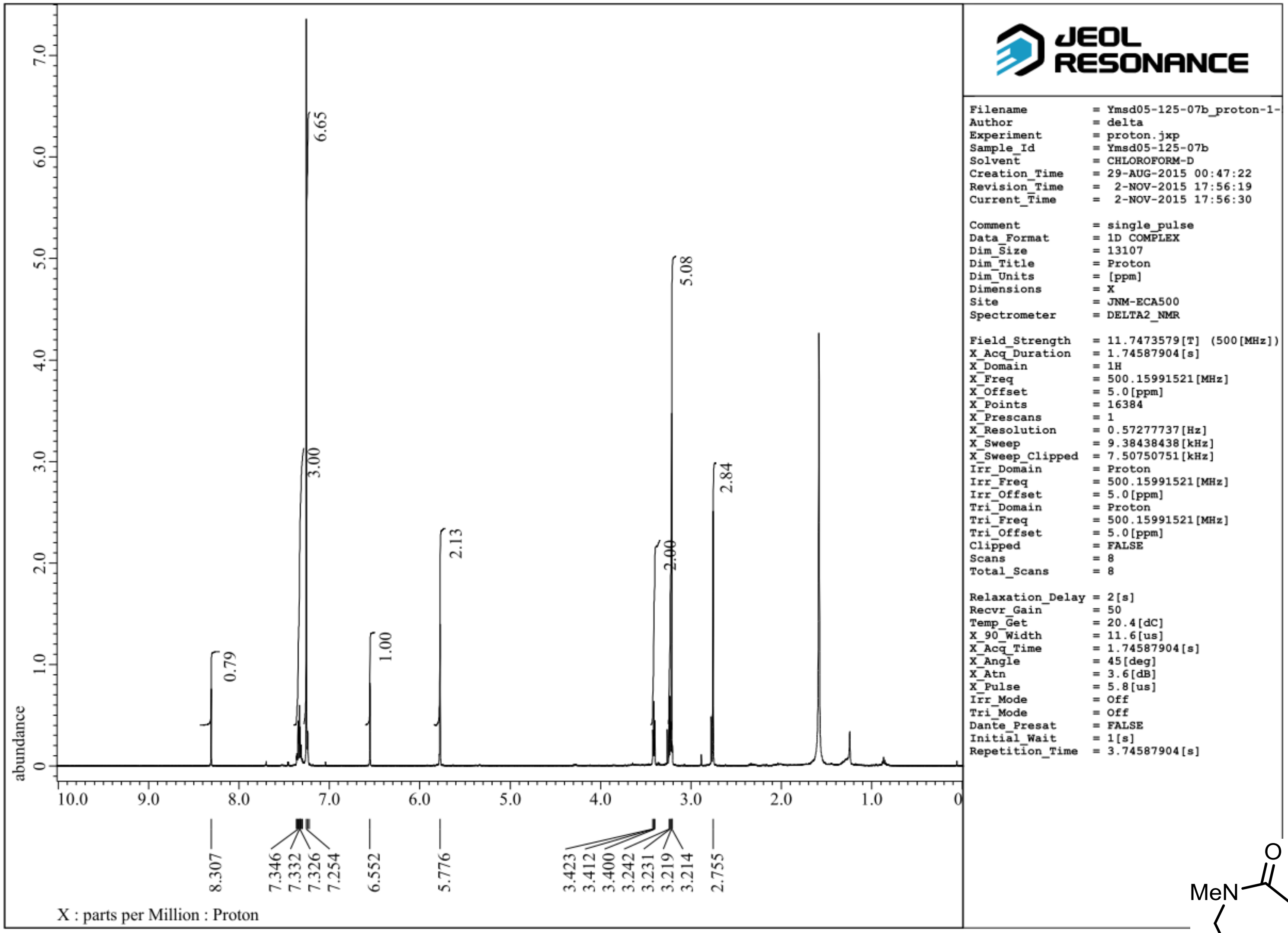

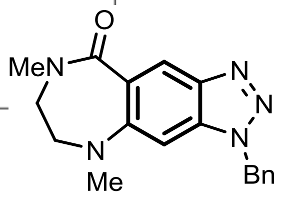

proximal-**19a**

You created this PDF from an application that is not licensed to print to novaPDF printer (http://www.novapdf.com)

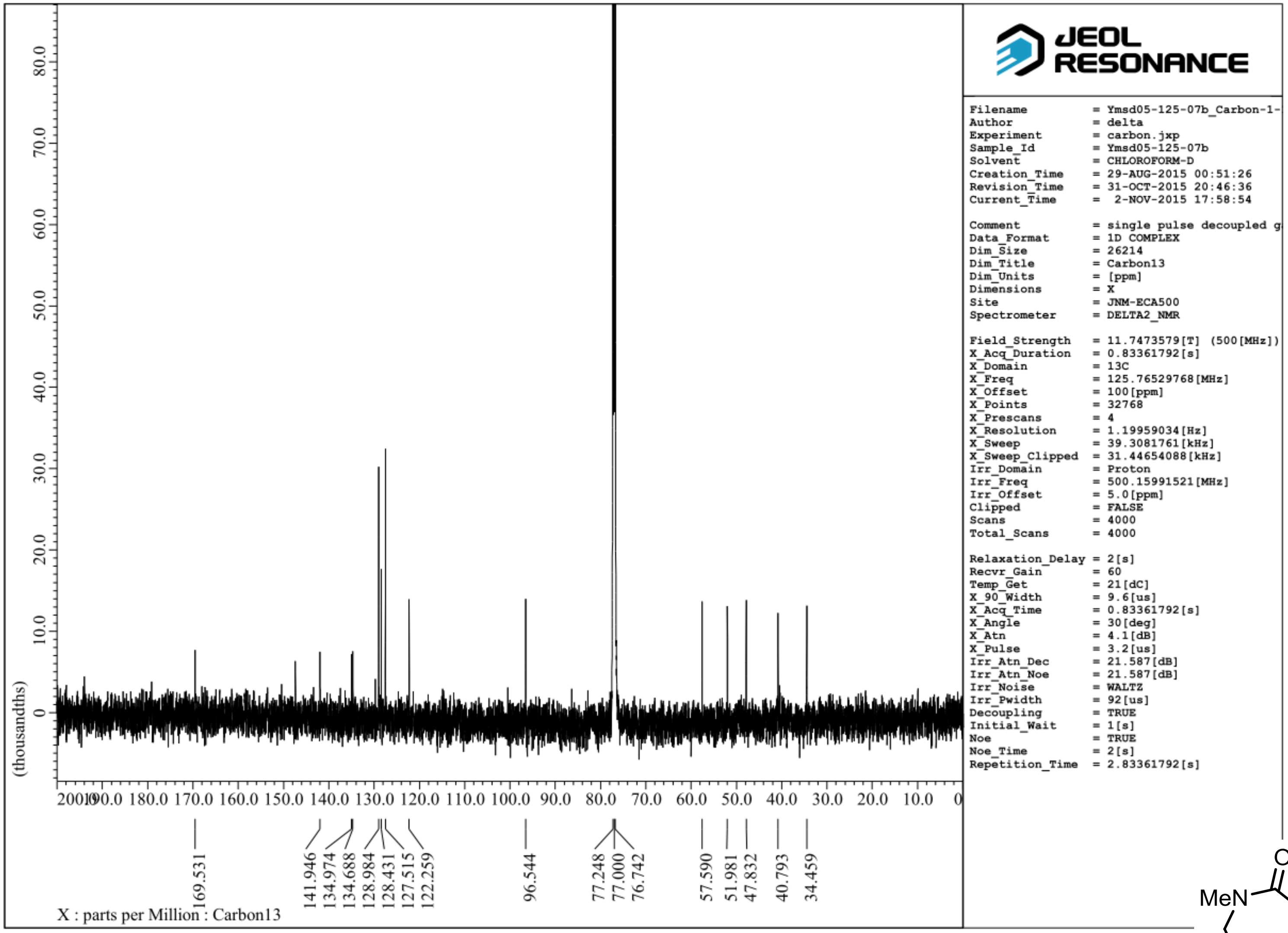

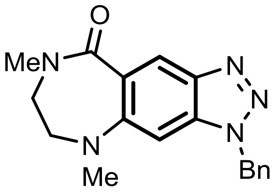

proximal-**19a**

You created this PDF from an application that is not licensed to print to novaPDF printer (http://www.novapdf.com)

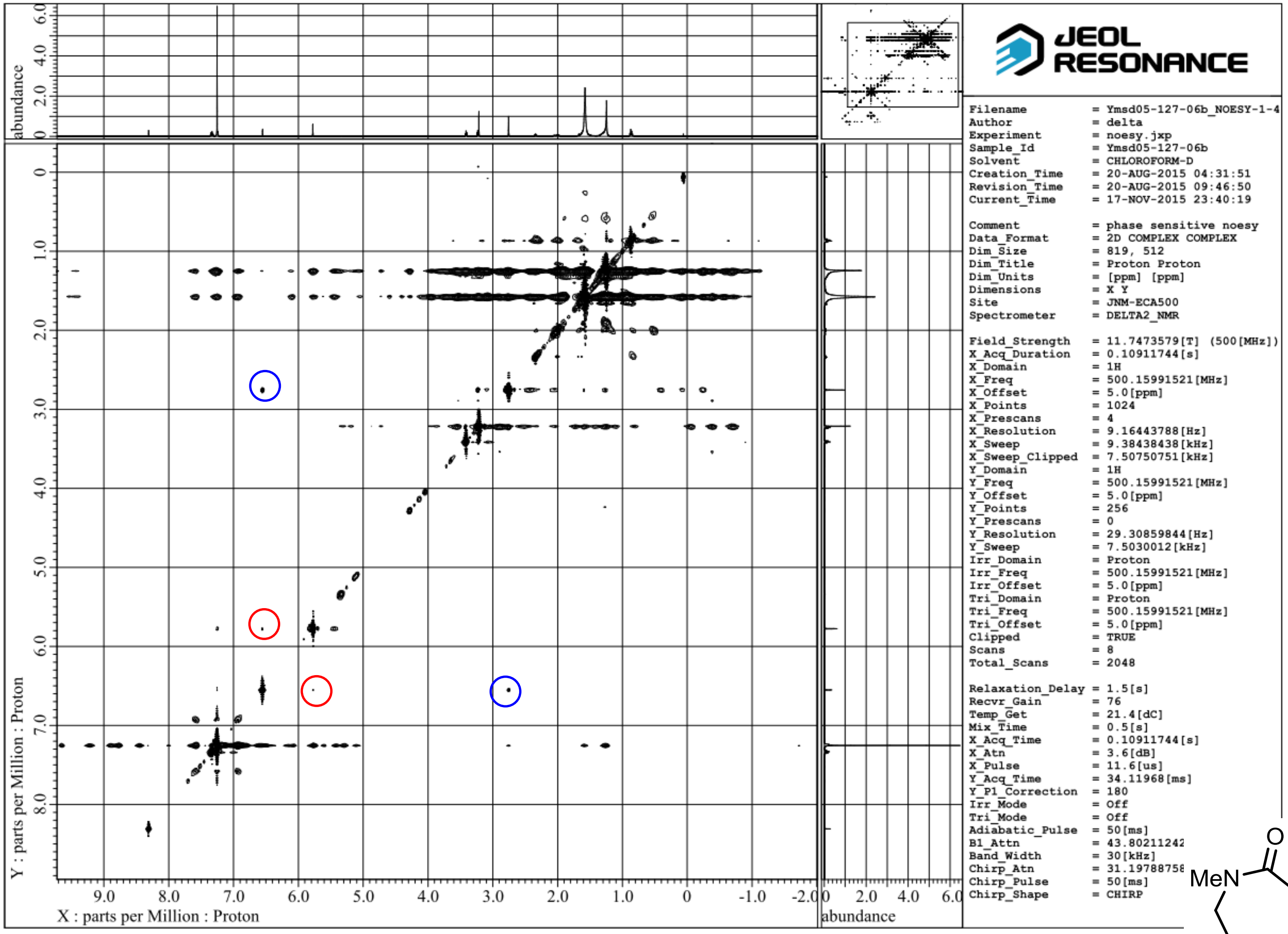

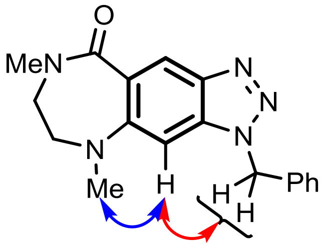

proximal-**19a**

You created this PDF from an application that is not licensed to print to novaPDF printer (http://www.novapdf.com)

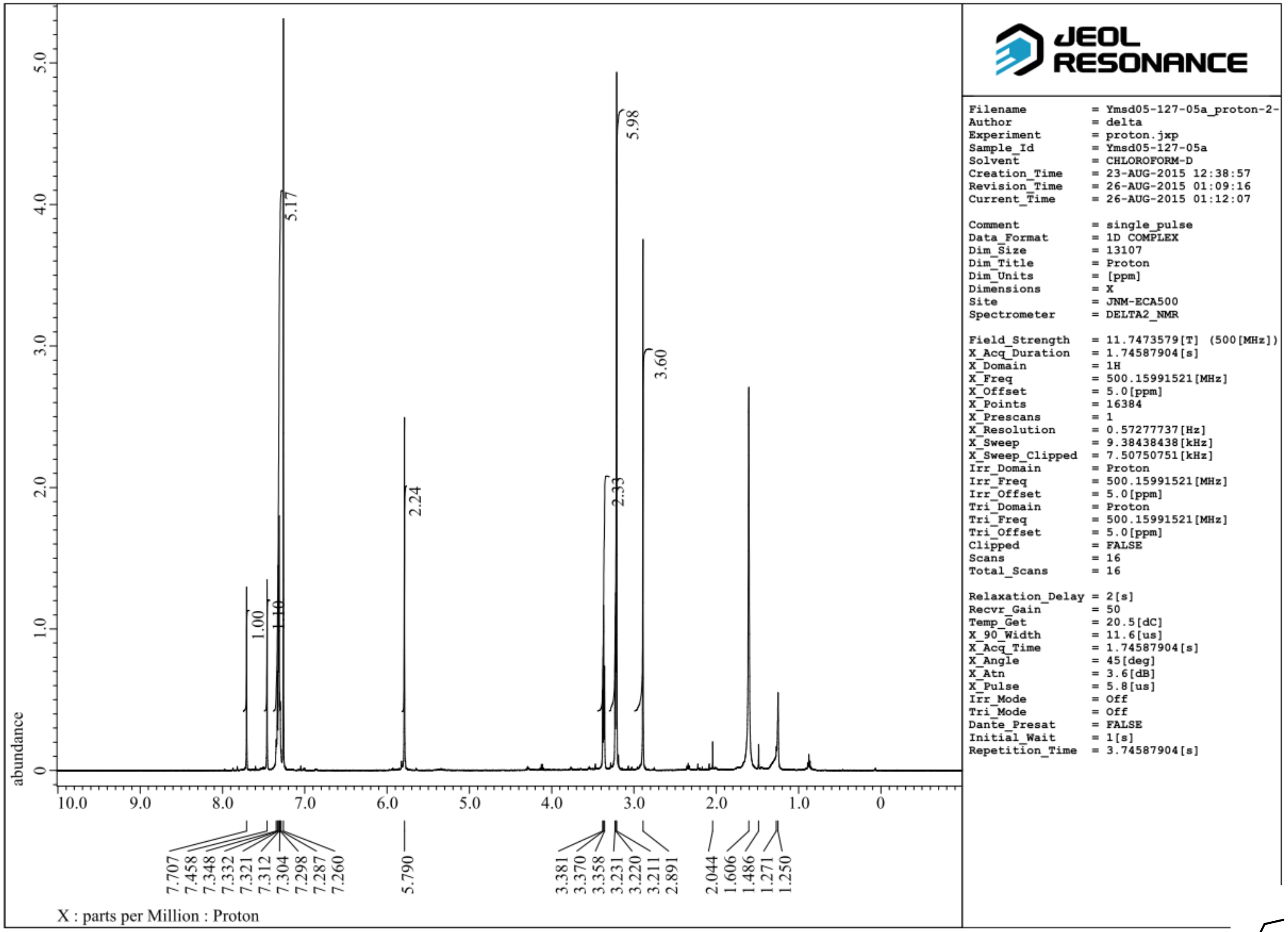

You created this PDF from an application that is not licensed to print to novaPDF printer (http://www.novapdf.com)

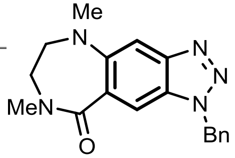

distal-**19a**

distal-**19a**

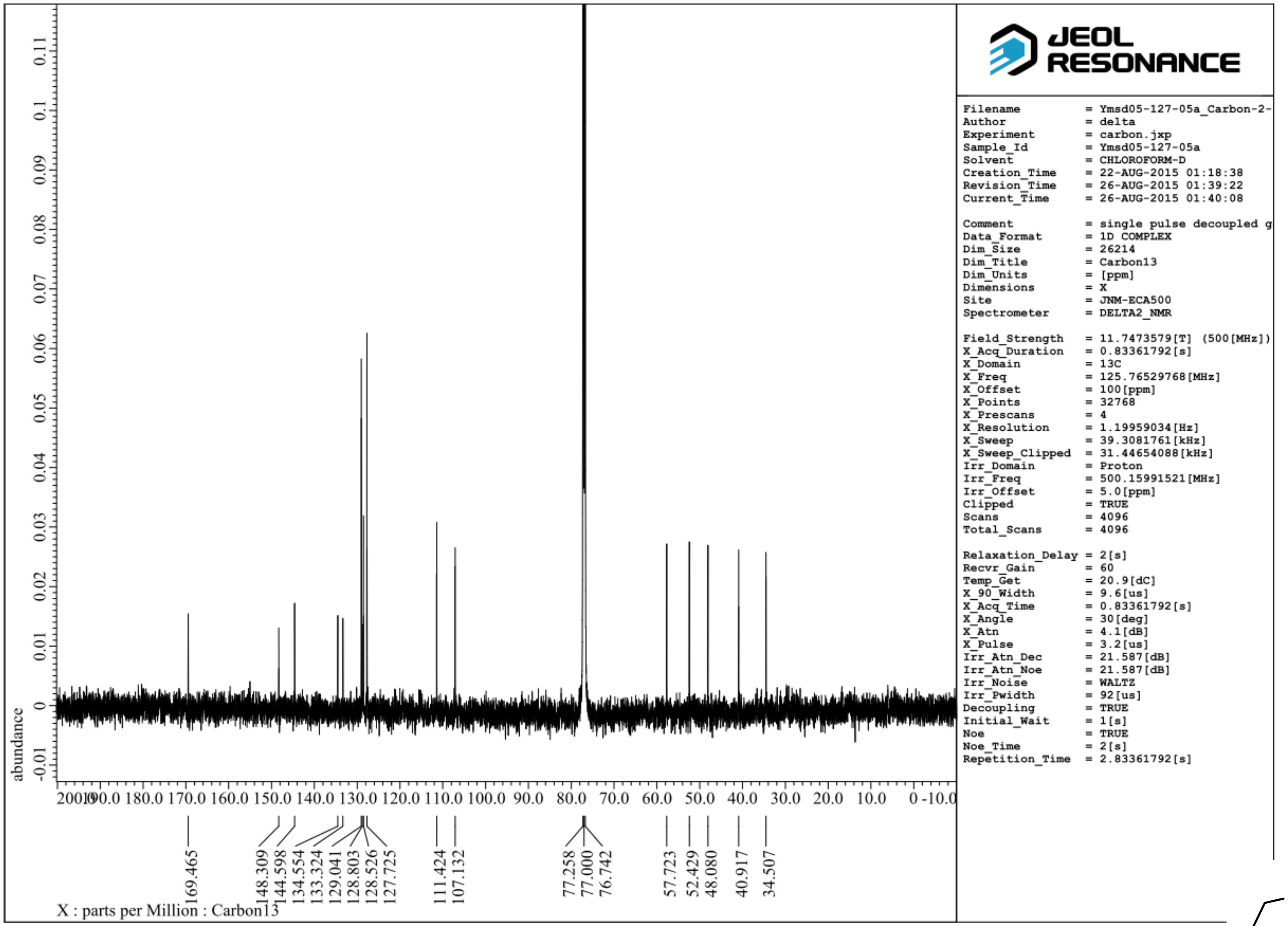

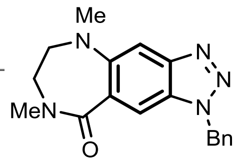

distal-**19a**

You created this PDF from an application that is not licensed to print to novaPDF printer (http://www.novapdf.com)

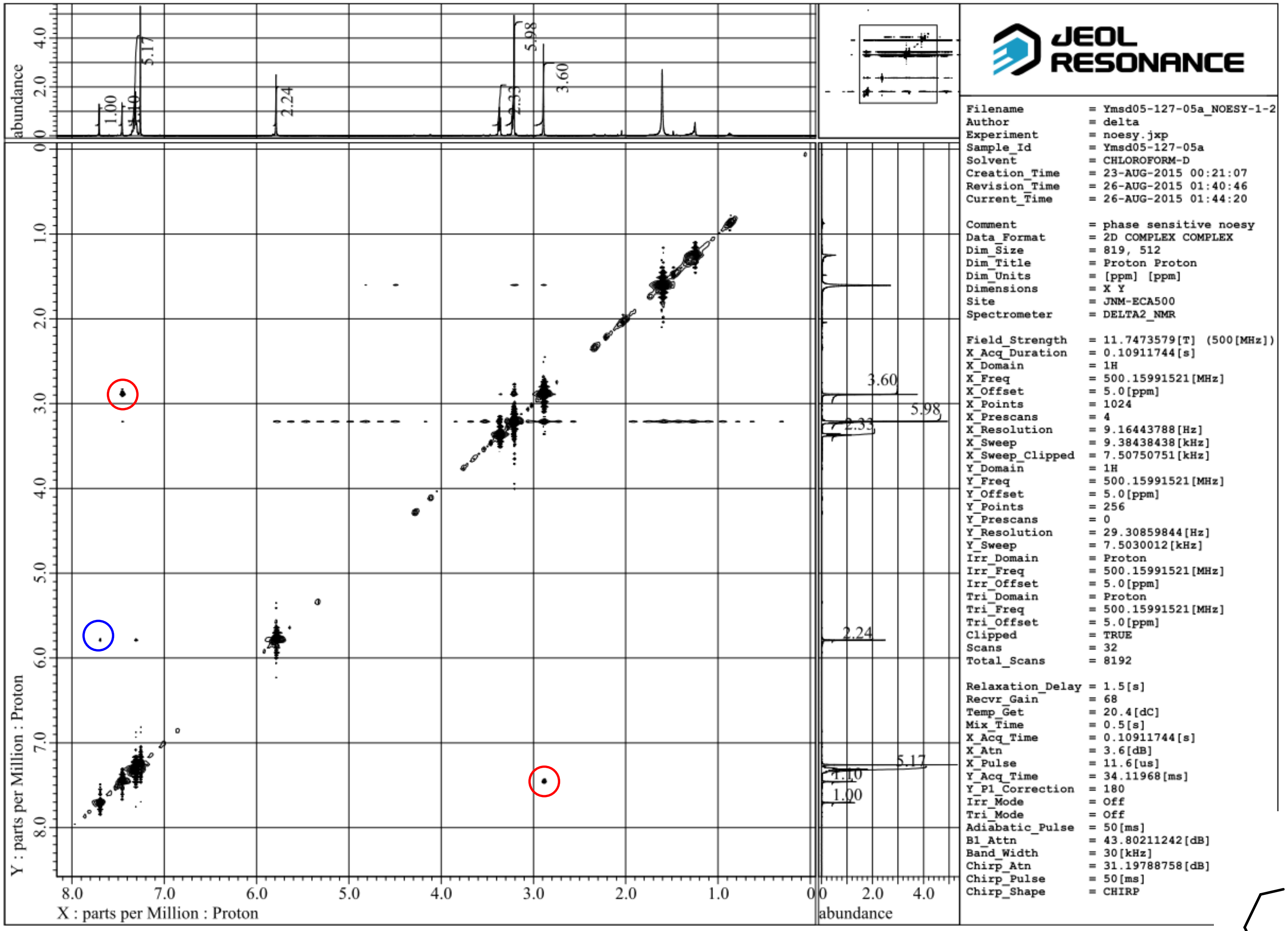

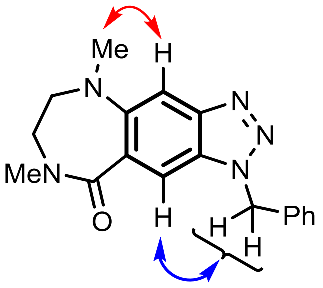

distal-**19a**

You created this PDF from an application that is not licensed to print to novaPDF printer (http://www.novapdf.com)

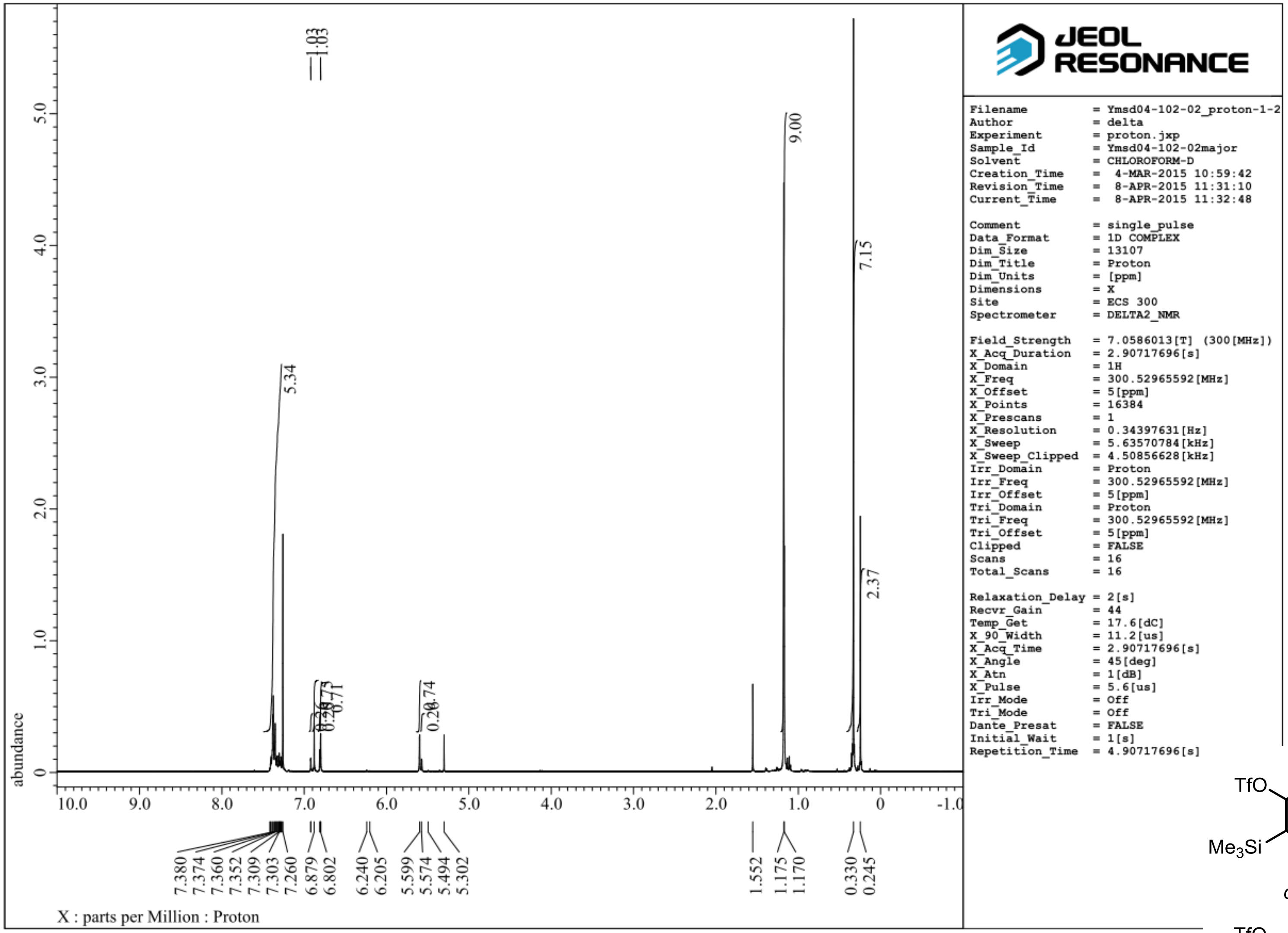

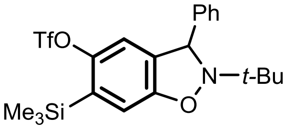

distal-**17b**

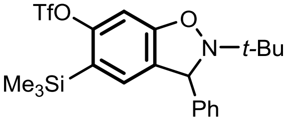

proximal-**17b**

You created this PDF from an application that is not licensed to print to novaPDF printer (http://www.novapdf.com)

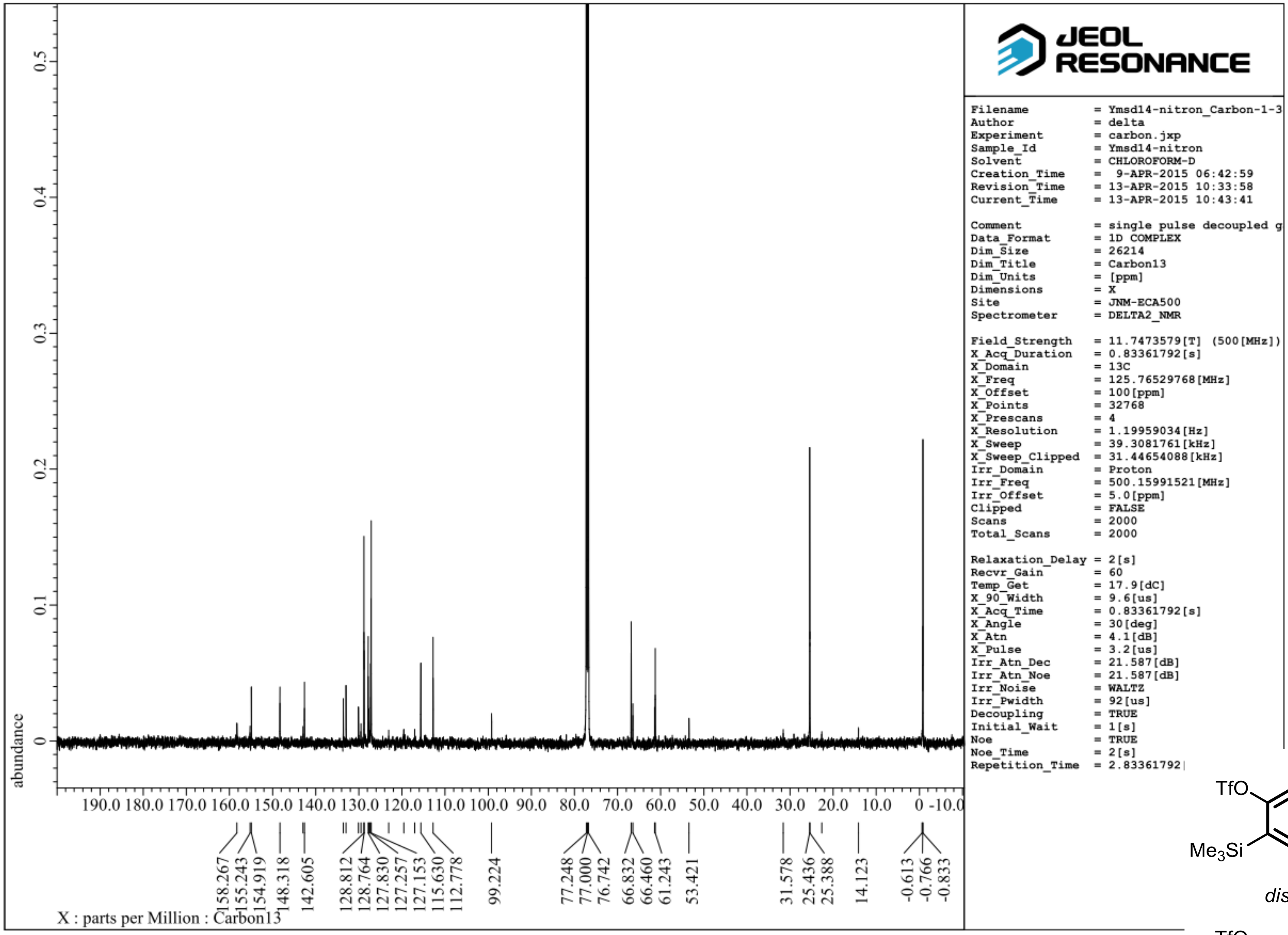

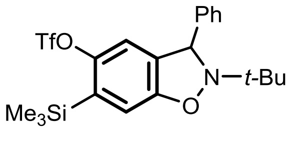

distal-**17b**

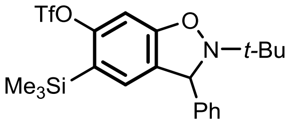

proximal-**17b**

You created this PDF from an application that is not licensed to print to novaPDF printer (http://www.novapdf.com)

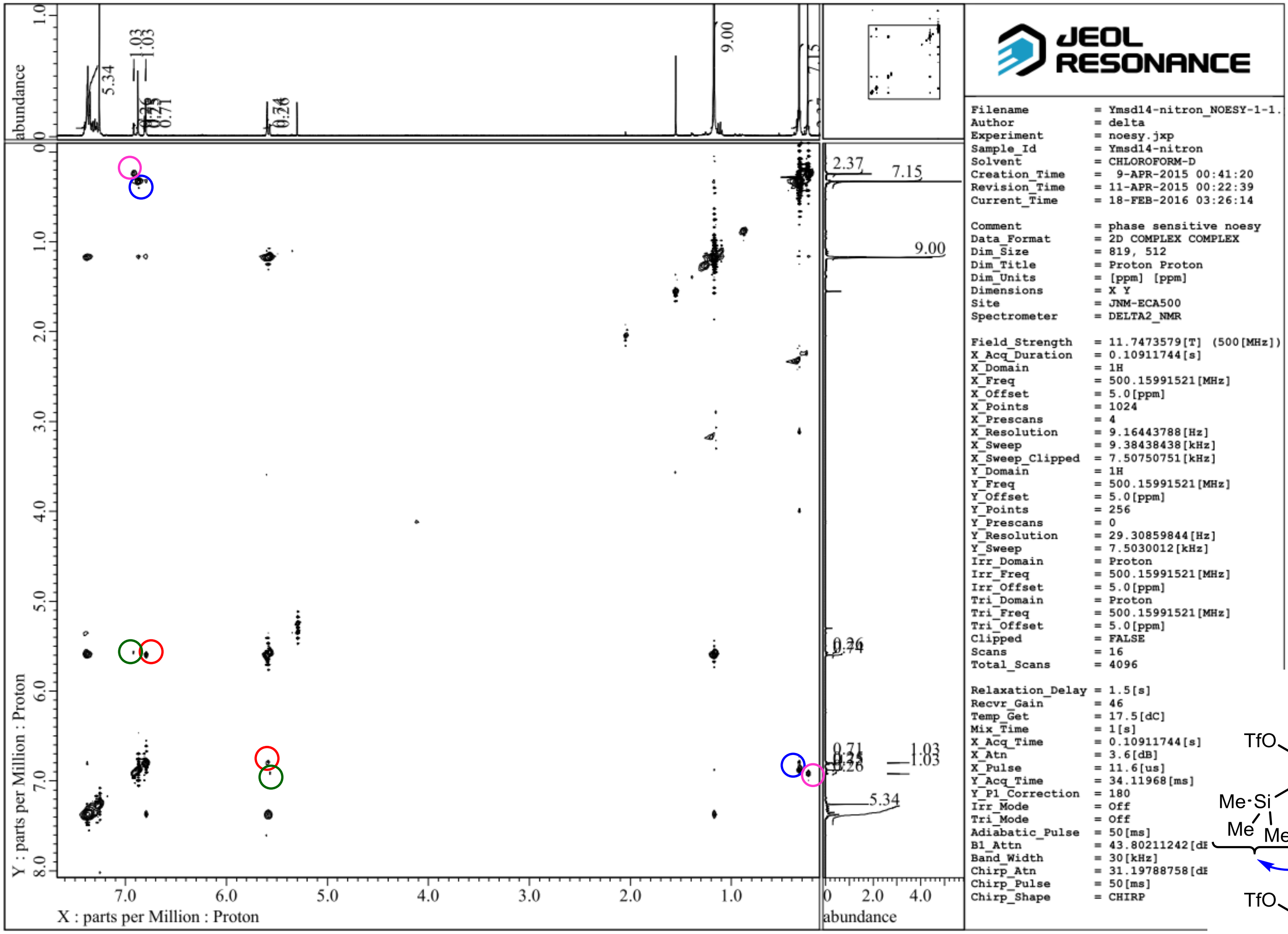

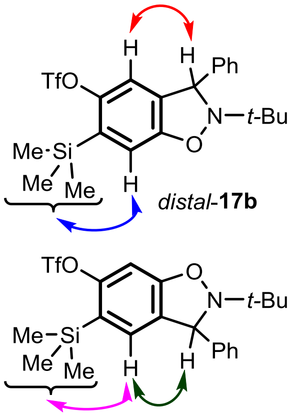

distal-**17b**

proximal-**17b**

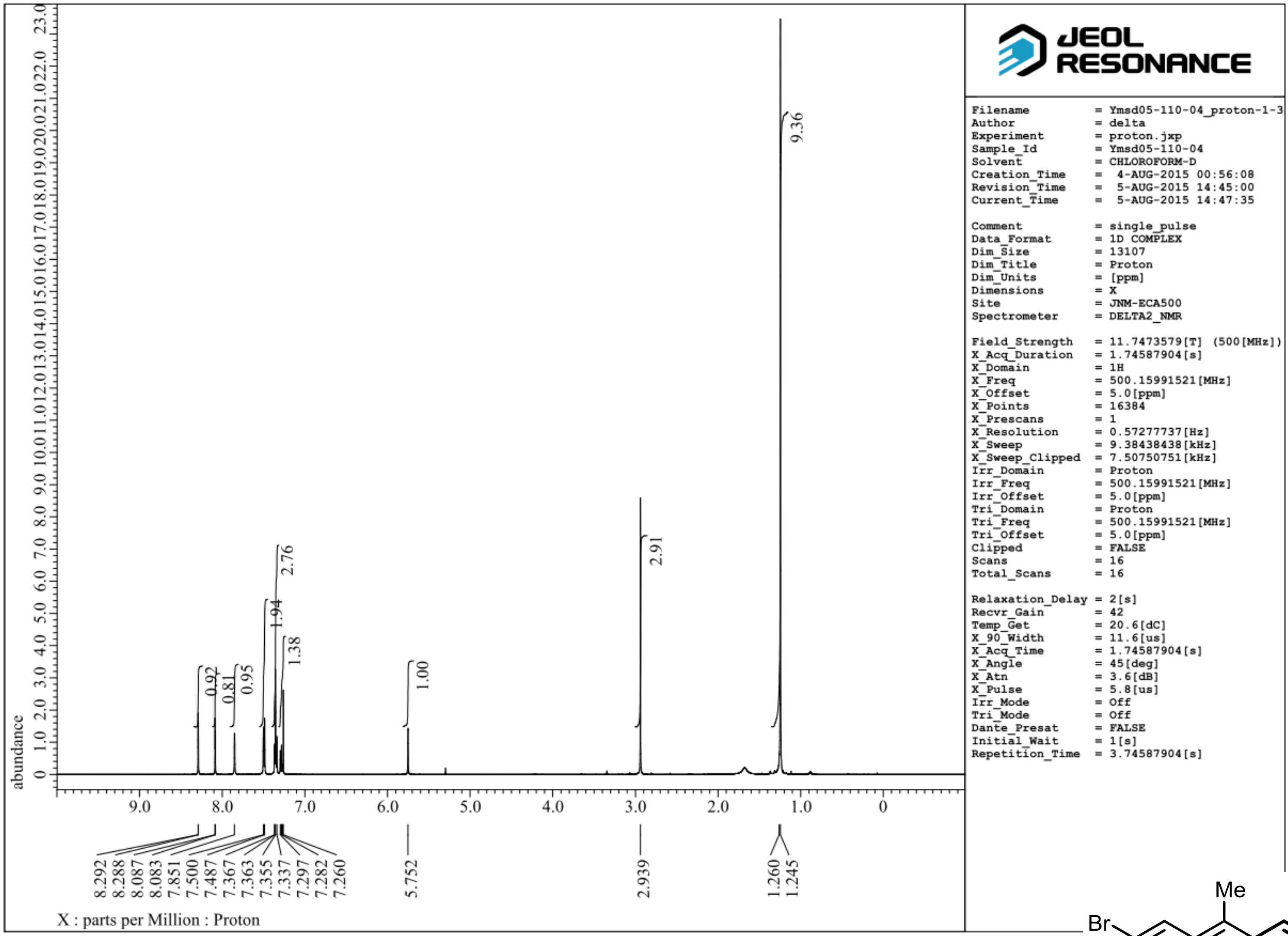

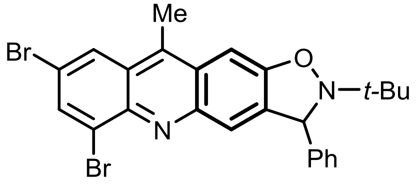

distal-**19b**

You created this PDF from an application that is not licensed to print to novaPDF printer (http://www.novapdf.com)

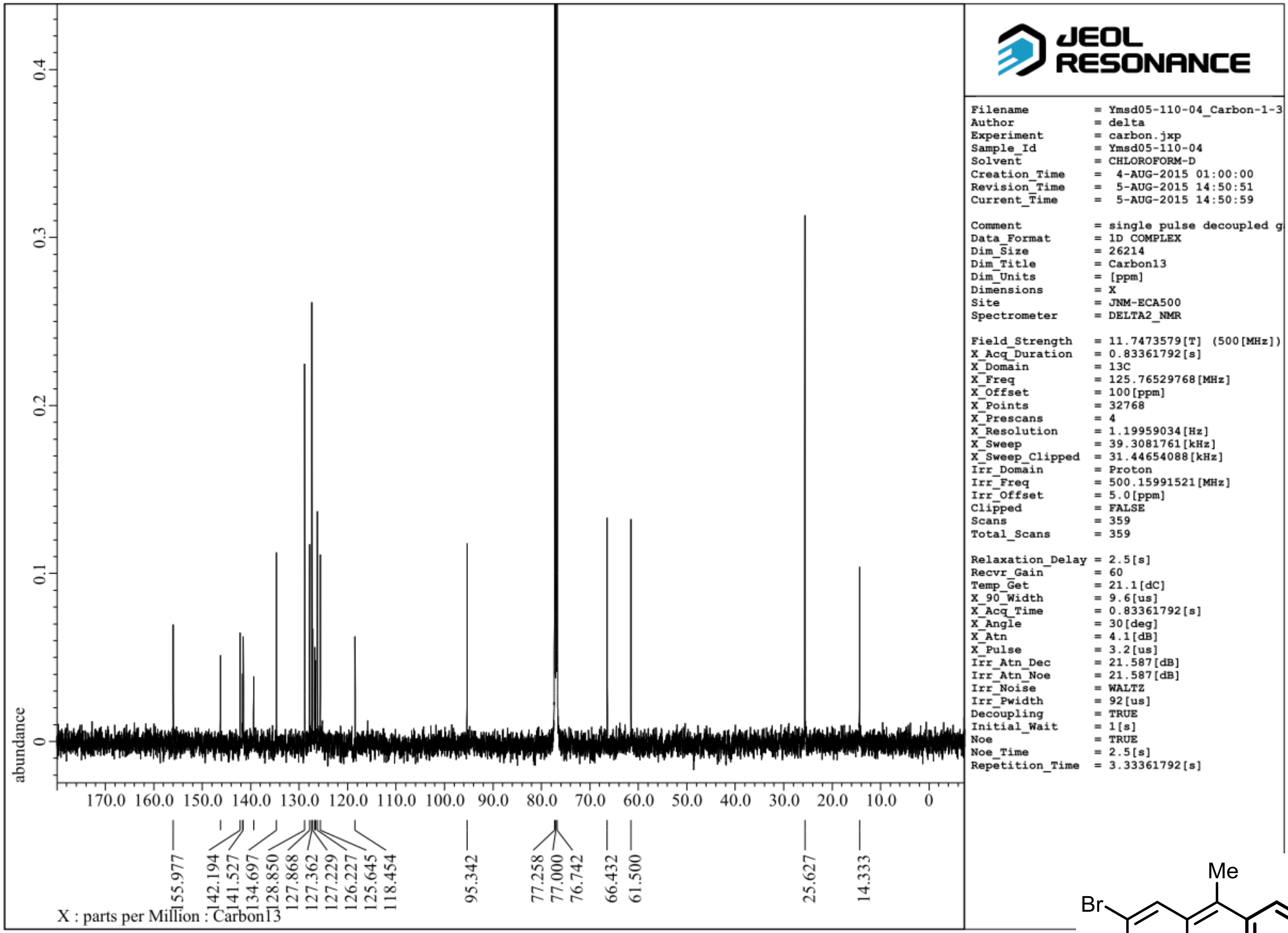

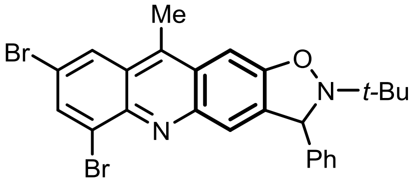

distal-**19b**

You created this PDF from an application that is not licensed to print to novaPDF printer (http://www.novapdf.com)

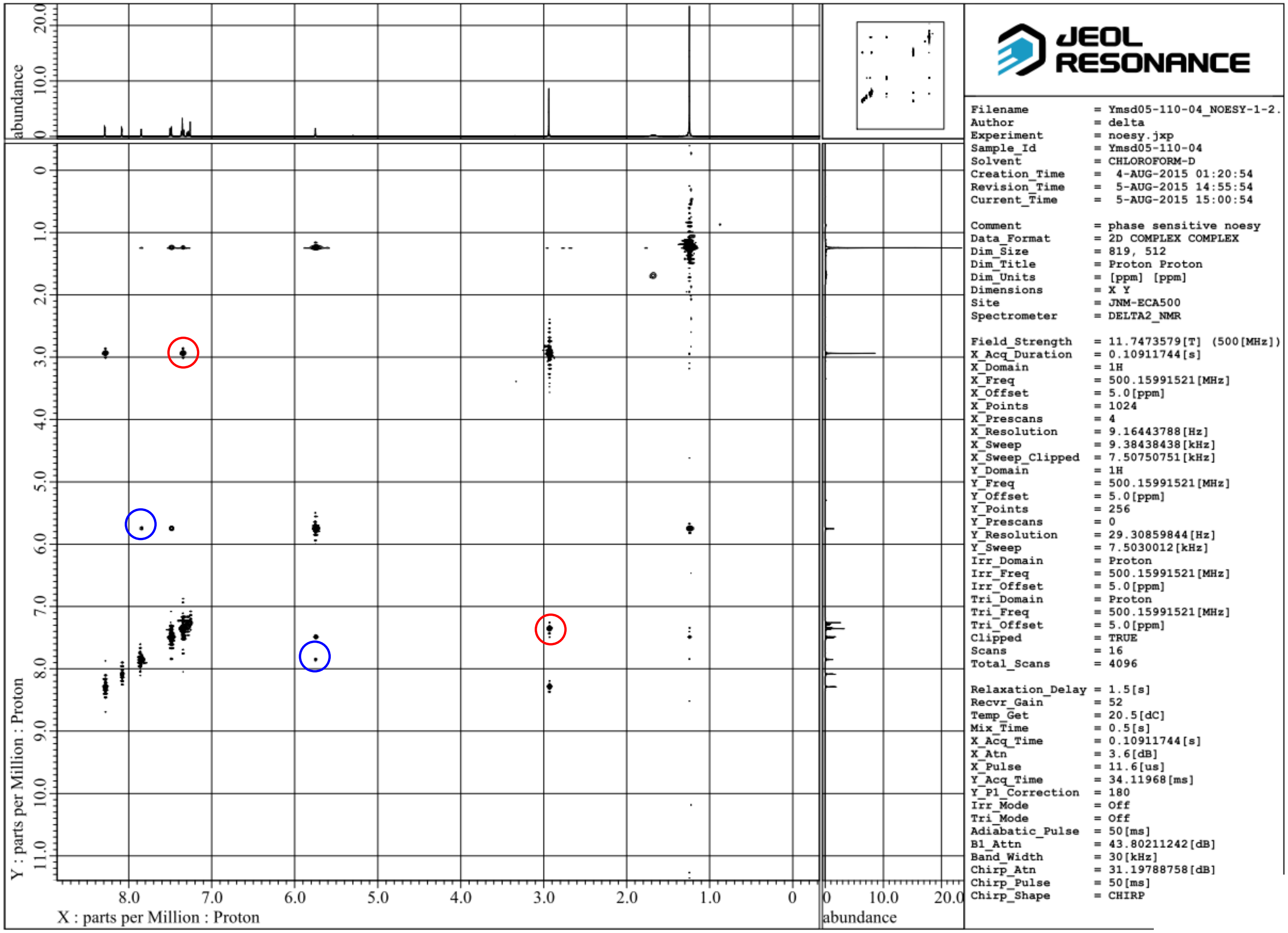

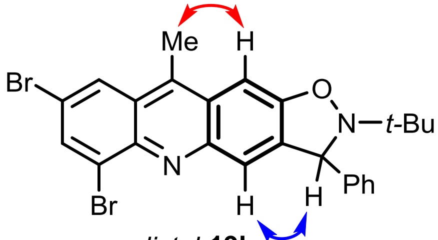

distal-**19b**

You created this PDF from an application that is not licensed to print to novaPDF printer (http://www.novapdf.com)

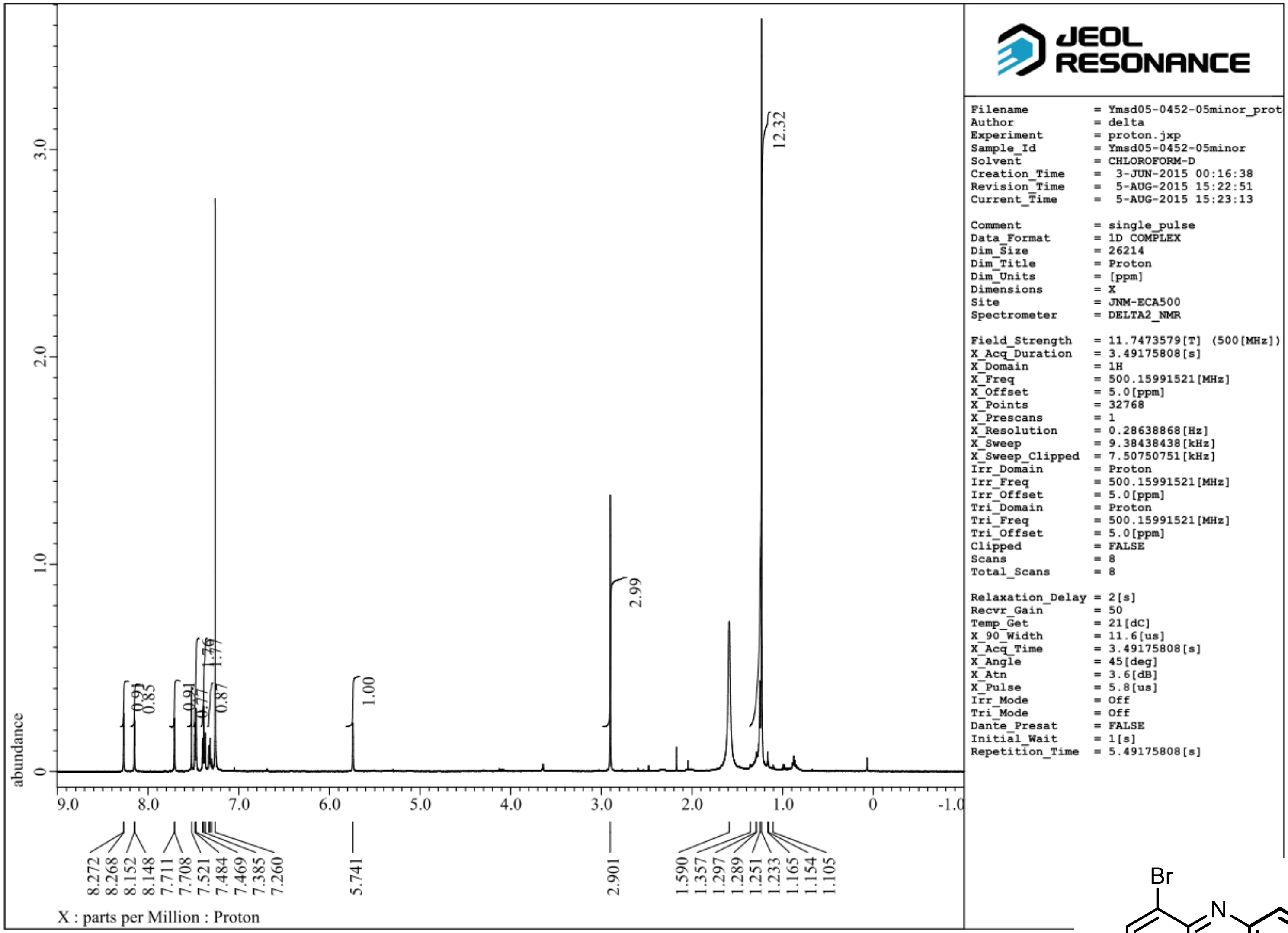

You created this PDF from an application that is not licensed to print to novaPDF printer (http://www.novapdf.com)

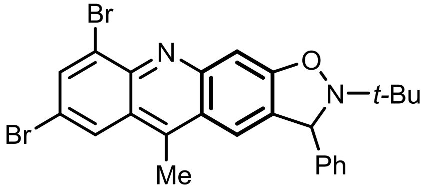

proximal-**19b**

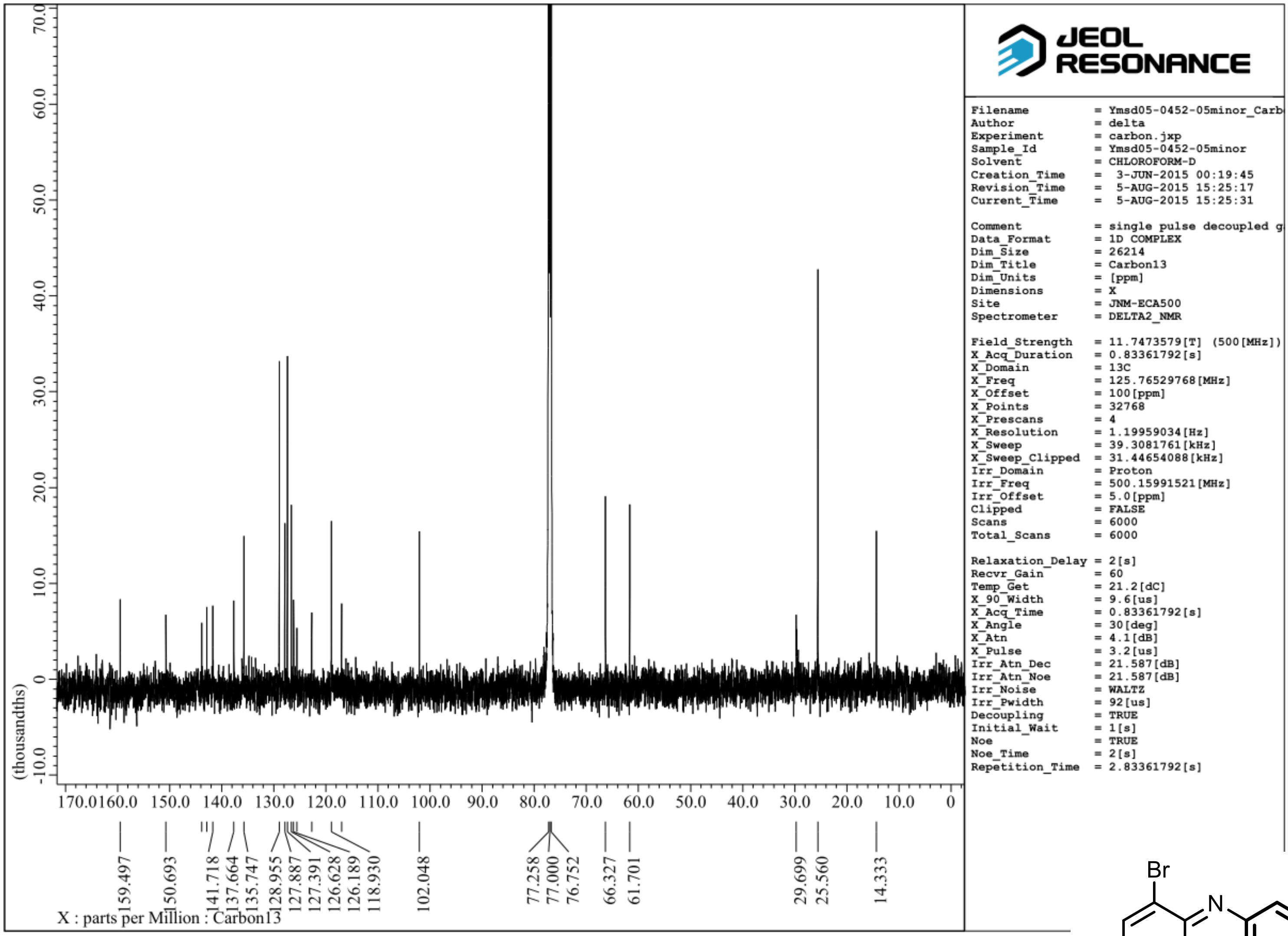

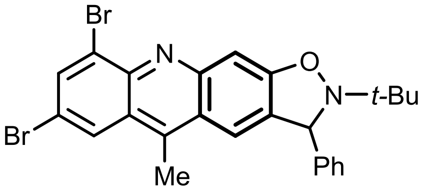

proximal-**19b**

You created this PDF from an application that is not licensed to print to novaPDF printer (http://www.novapdf.com)

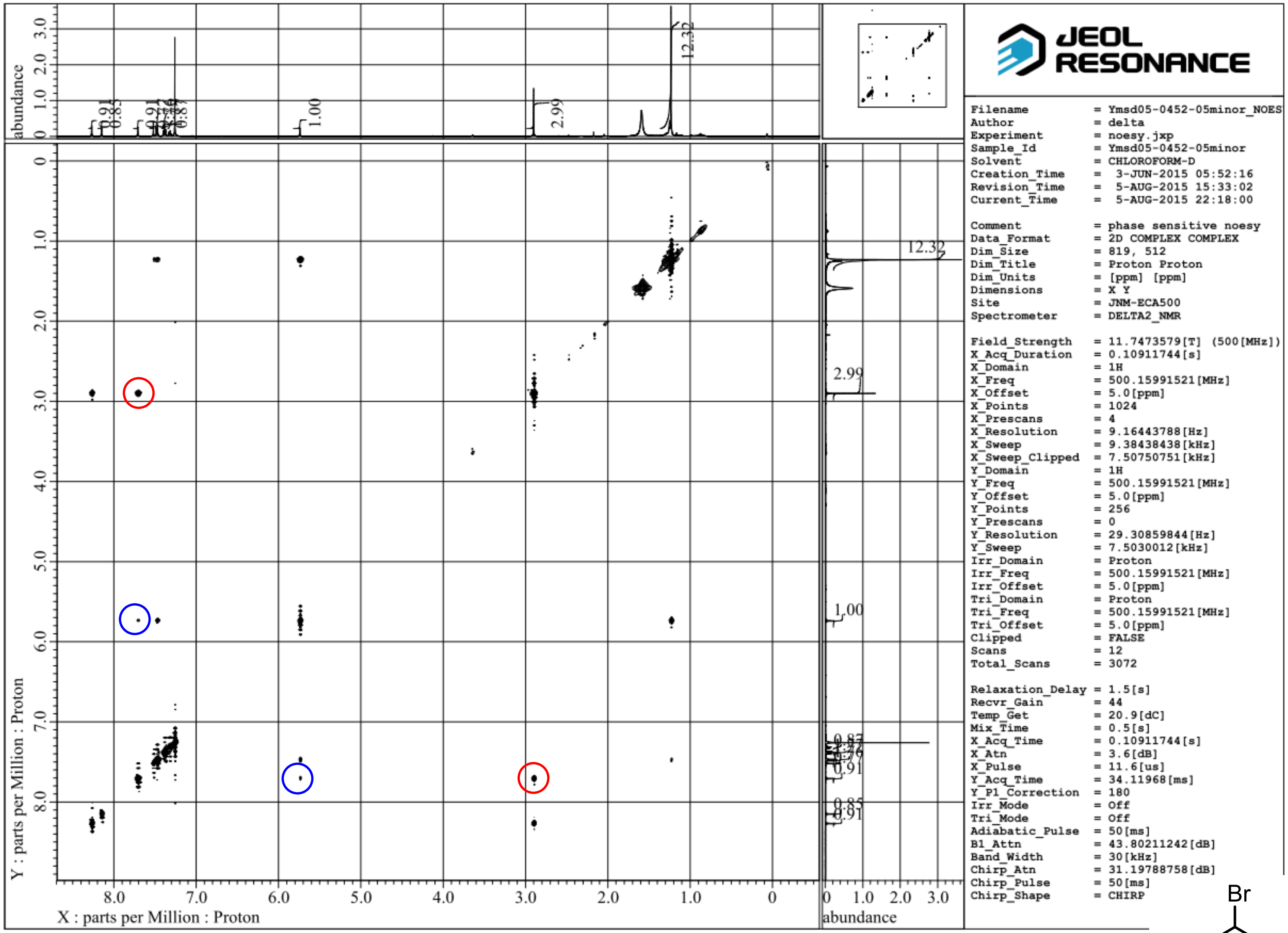

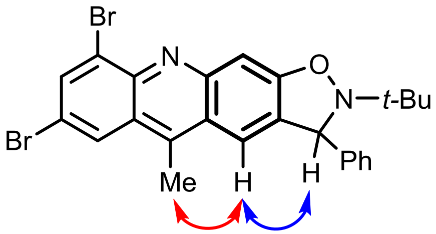

proximal-**19b**

You created this PDF from an application that is not licensed to print to novaPDF printer (http://www.novapdf.com)

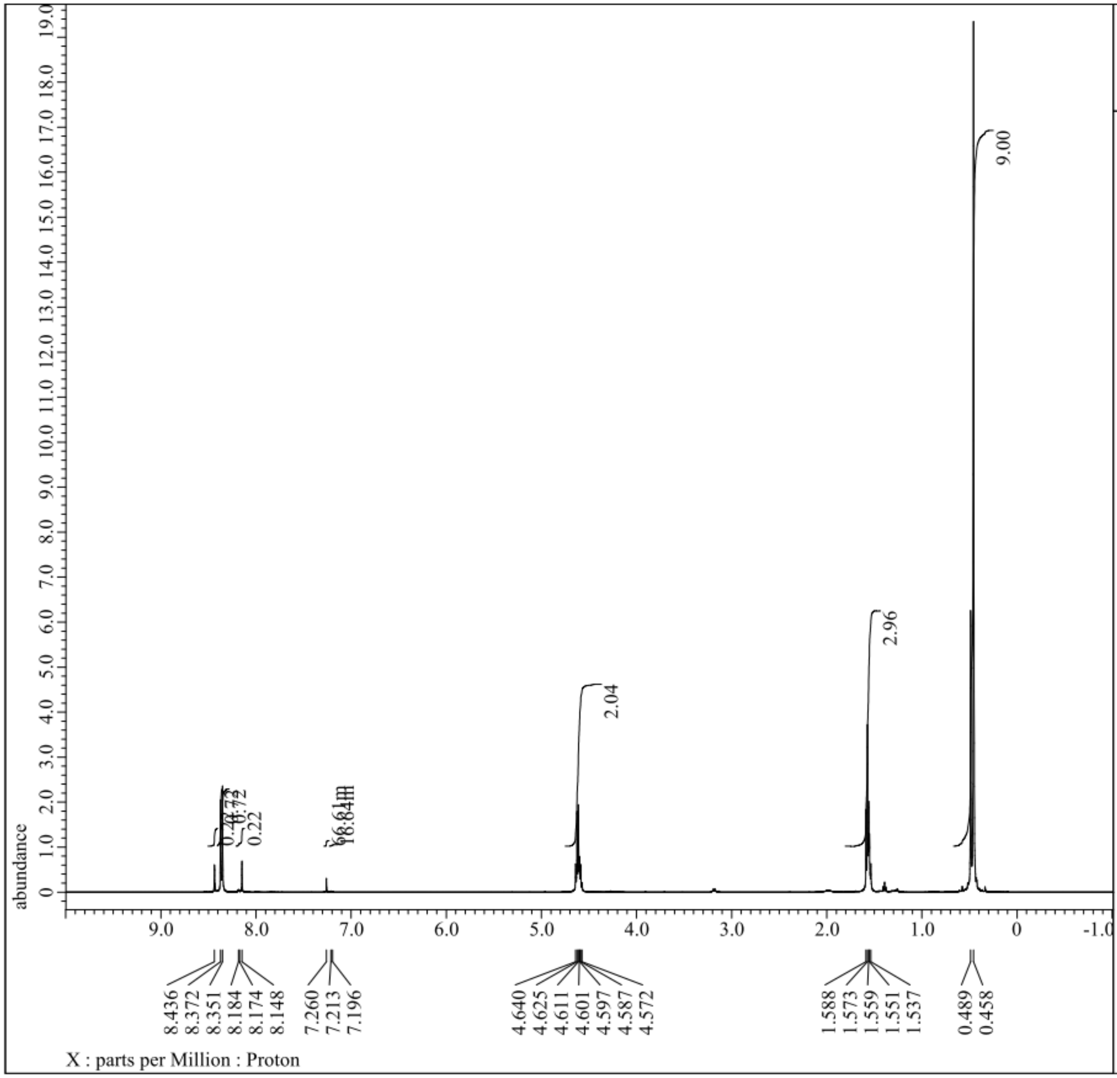


```
Filename           = Ymsd04-175-06_proton-1-4
Author             = delta
Experiment         = proton.jxp
Sample_Id          = Ymsd04-175-06
Solvent            = CHLOROFORM-D
Creation_Time      = 16-APR-2015 02:01:35
Revision_Time      = 29-MAY-2015 18:45:23
Current_Time       = 29-MAY-2015 18:47:37

Comment            = single_pulse
Data_Format        = 1D COMPLEX
Dim_Size           = 13107
Dim_Title          = Proton
Dim_Units          = [ppm]
Dimensions         = X
Site               = JNM-ECA500
Spectrometer       = DELTA2_NMR

Field_Strength     = 11.7473579[T] (500[MHz])
X_Acq_Duration     = 1.74587904[s]
X_Domain           = 1H
X_Freq             = 500.15991521[MHz]
X_Offset           = 5.0[ppm]
X_Points           = 16384
X_Prescans         = 1
X_Resolution       = 0.57277737[Hz]
X_Sweep            = 9.38438438[kHz]
X_Sweep_Clipped    = 7.50750751[kHz]
Irr_Domain         = Proton
Irr_Freq           = 500.15991521[MHz]
Irr_Offset         = 5.0[ppm]
Tri_Domain         = Proton
Tri_Freq           = 500.15991521[MHz]
Tri_Offset         = 5.0[ppm]
Clipped            = FALSE
Scans              = 8
Total_Scans        = 8

Relaxation_Delay   = 2[s]
Recvr_Gain         = 30
Temp_Get           = 17.7[dC]
X_90_Width         = 11.6[us]
X_Acq_Time         = 1.74587904[s]
X_Angle            = 45[deg]
X_Atn              = 3.6[dB]
X_Pulse            = 5.8[us]
Irr_Mode           = Off
Tri_Mode           = Off
Dante_Presat       = FALSE
Initial_Wait       = 1[s]
Repetition_Time    = 3.74587904[s]
```

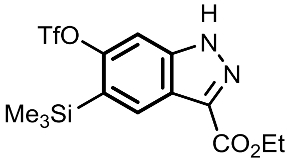

proximal-**17c**

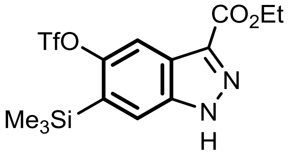

distal-**17c**

You created this PDF from an application that is not licensed to print to novaPDF printer (http://www.novapdf.com)

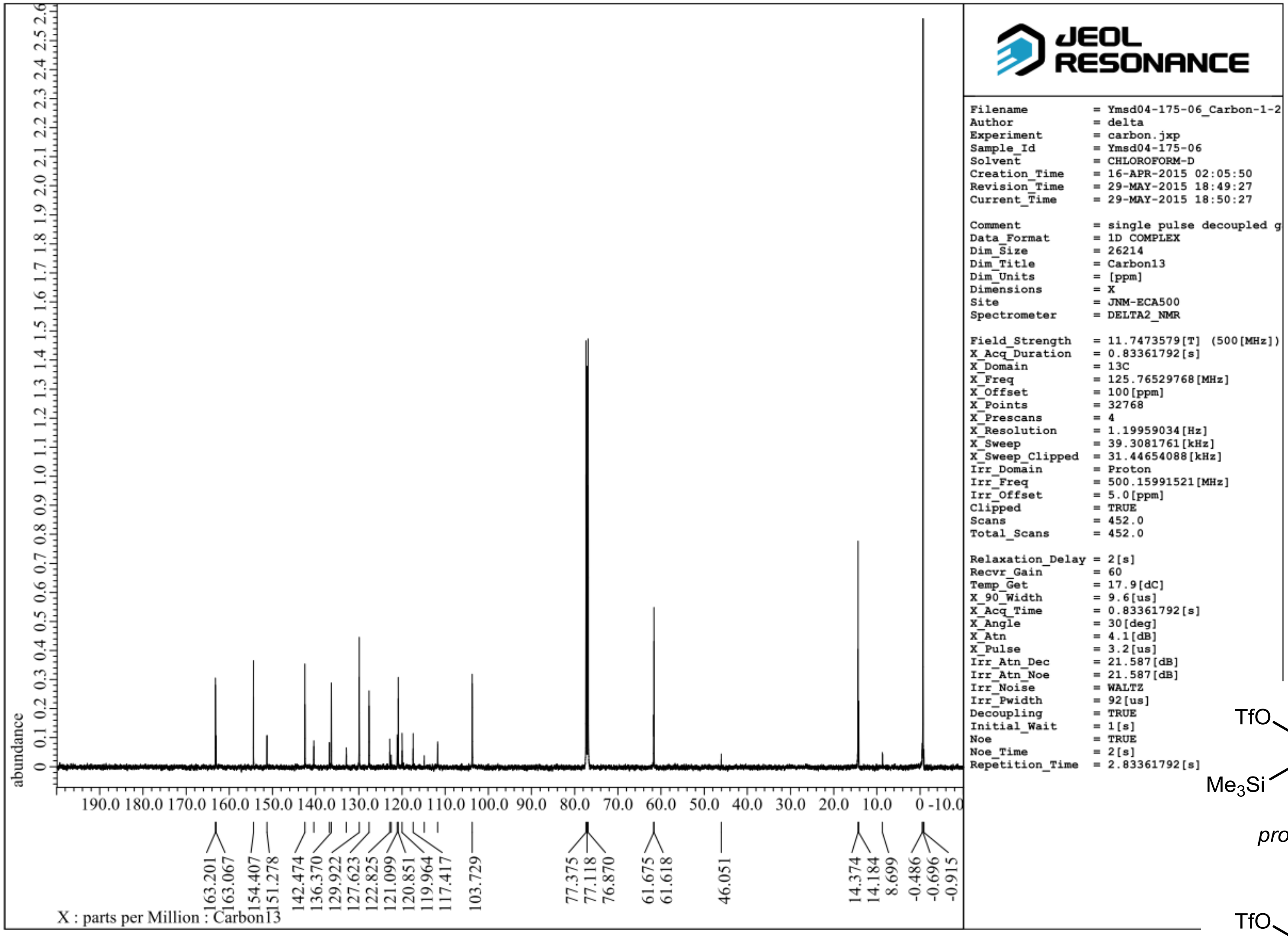

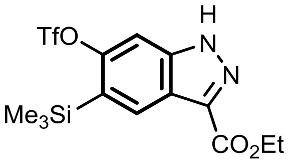

proximal-**17c**

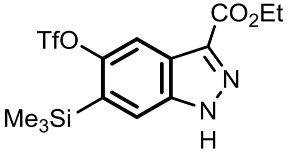

distal-**17c**

You created this PDF from an application that is not licensed to print to novaPDF printer (http://www.novapdf.com)

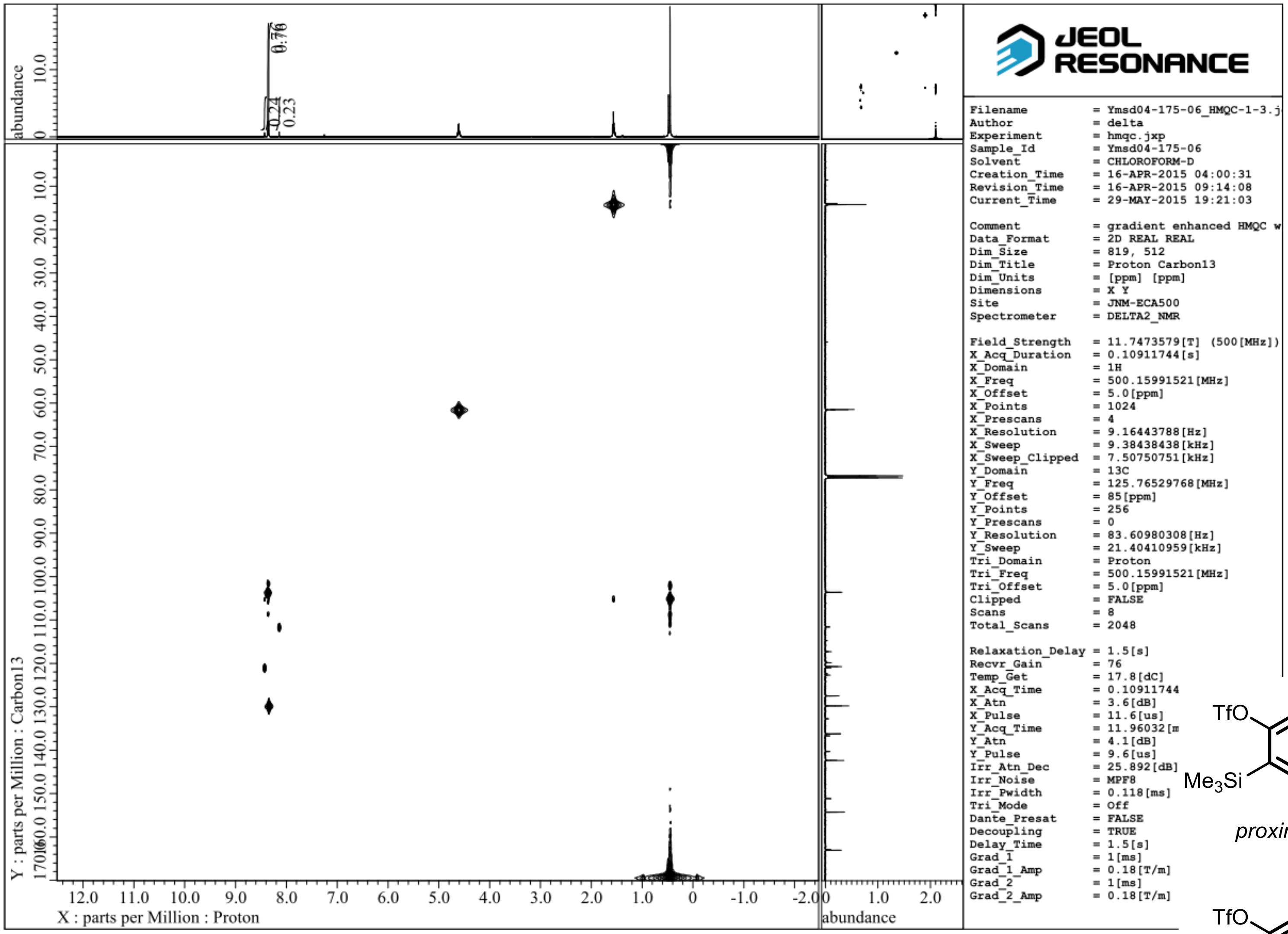

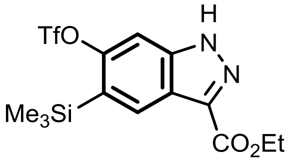

proximal-**17c**

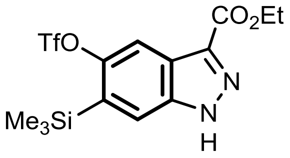

distal-**17c**

You created this PDF from an application that is not licensed to print to novaPDF printer (http://www.novapdf.com)

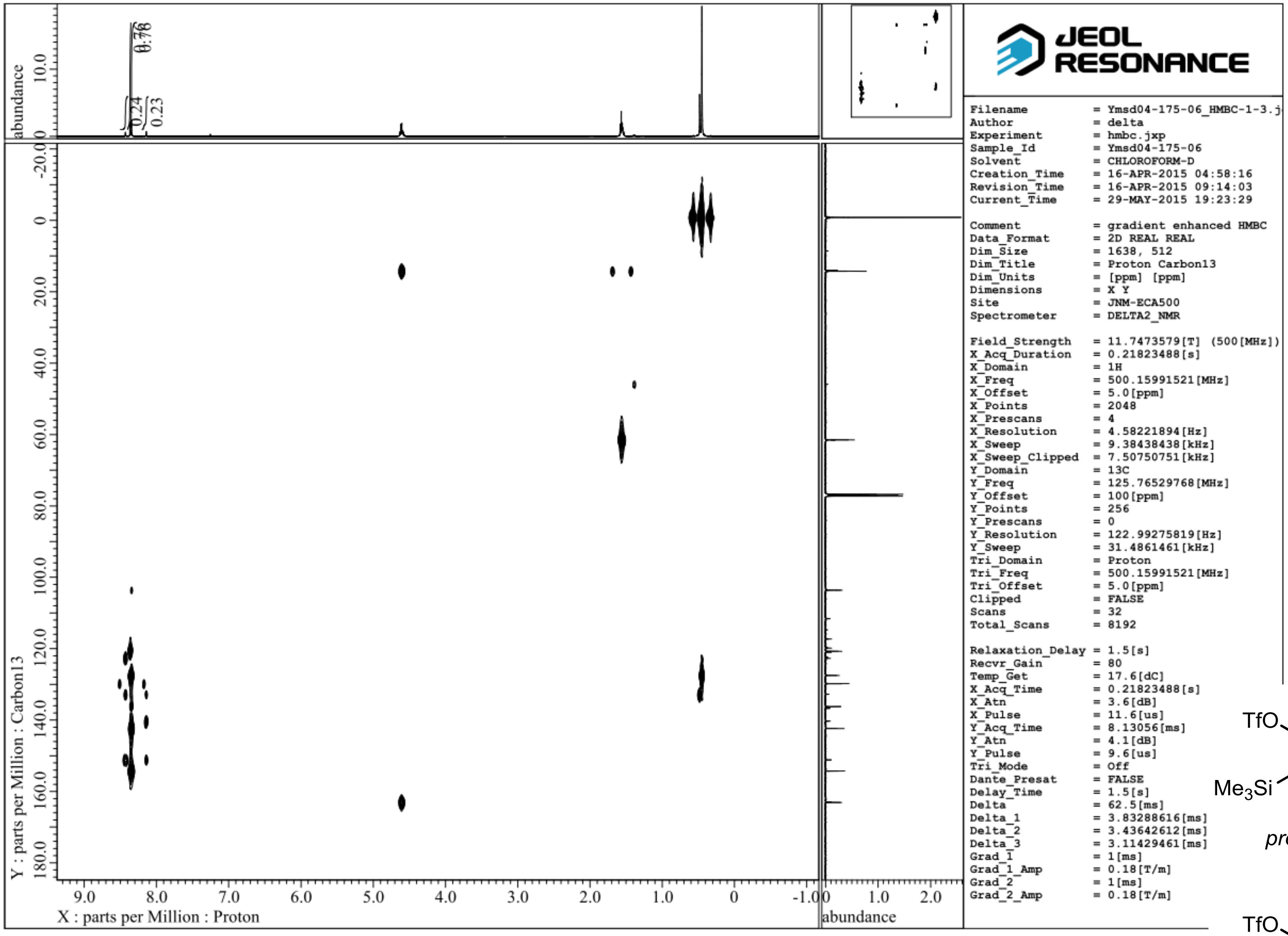

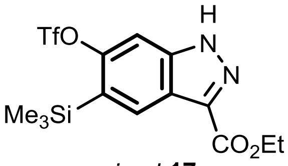

proximal-**17c**

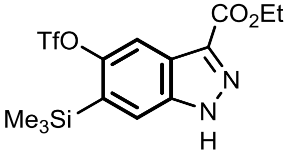

distal-**17c**

You created this PDF from an application that is not licensed to print to novaPDF printer (http://www.novapdf.com)

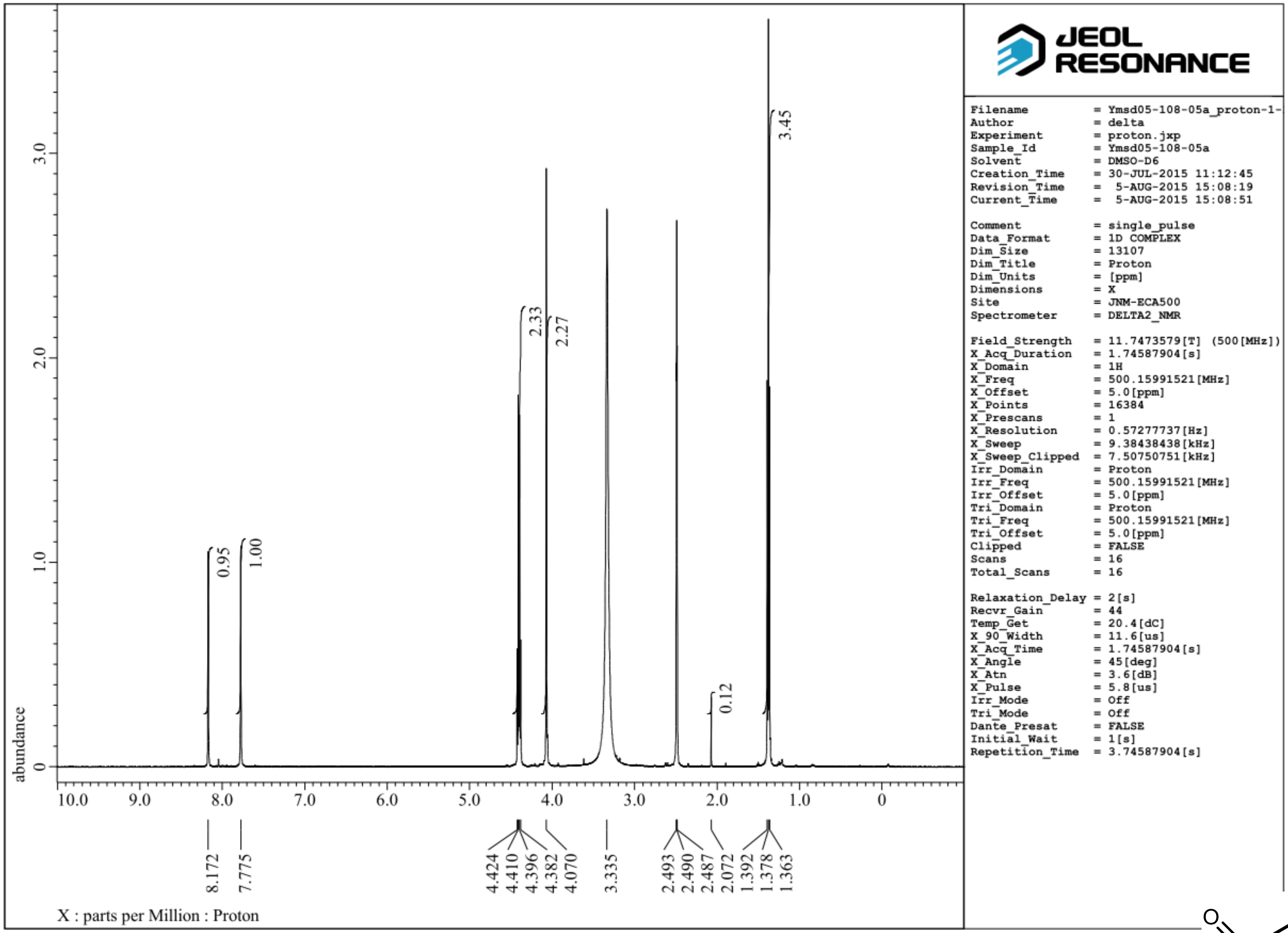

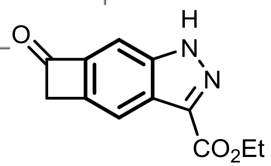

distal-**19c'**

You created this PDF from an application that is not licensed to print to novaPDF printer (http://www.novapdf.com)

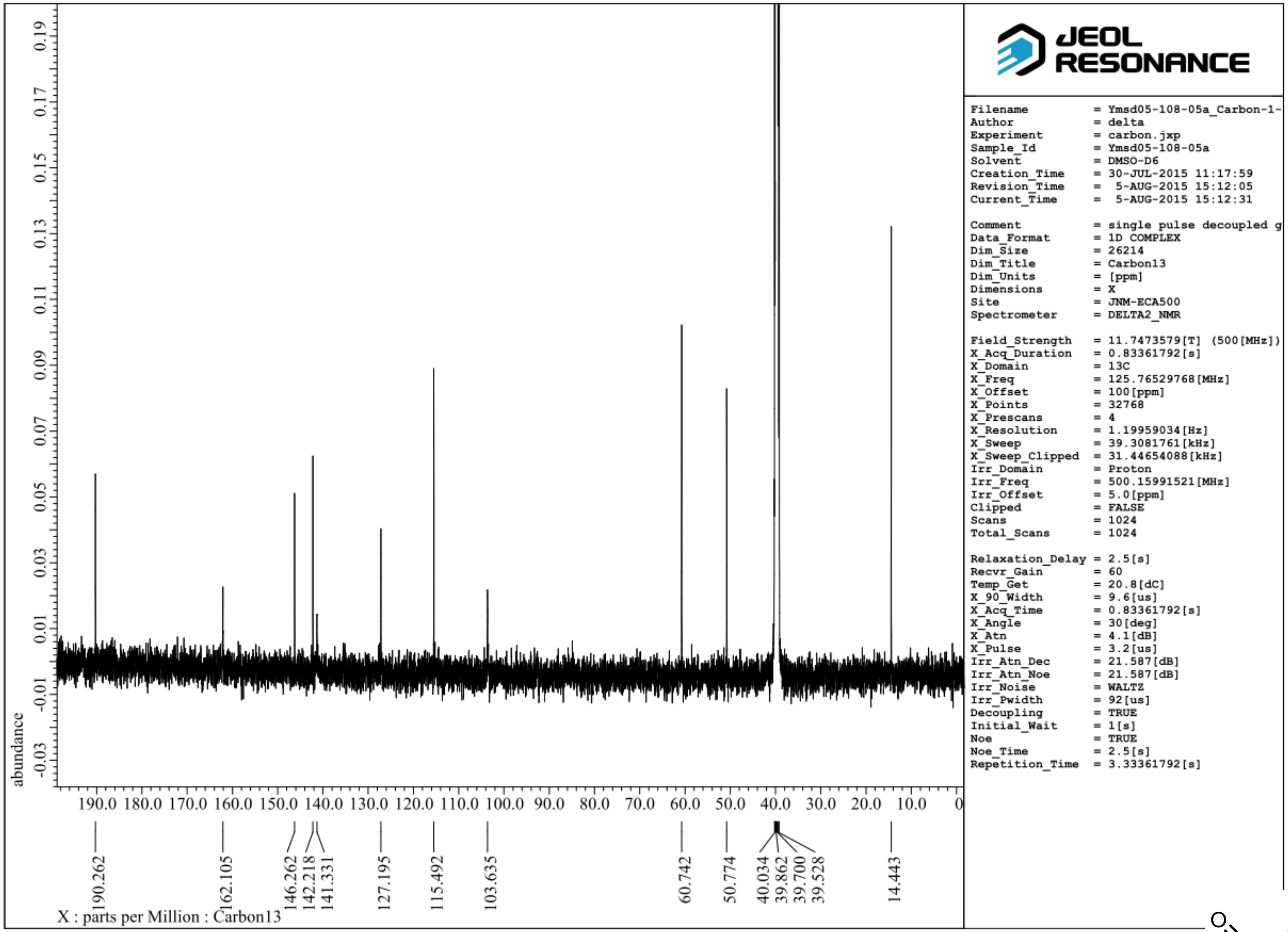

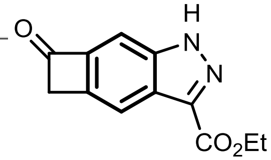

distal-**19c'**

You created this PDF from an application that is not licensed to print to novaPDF printer (http://www.novapdf.com)

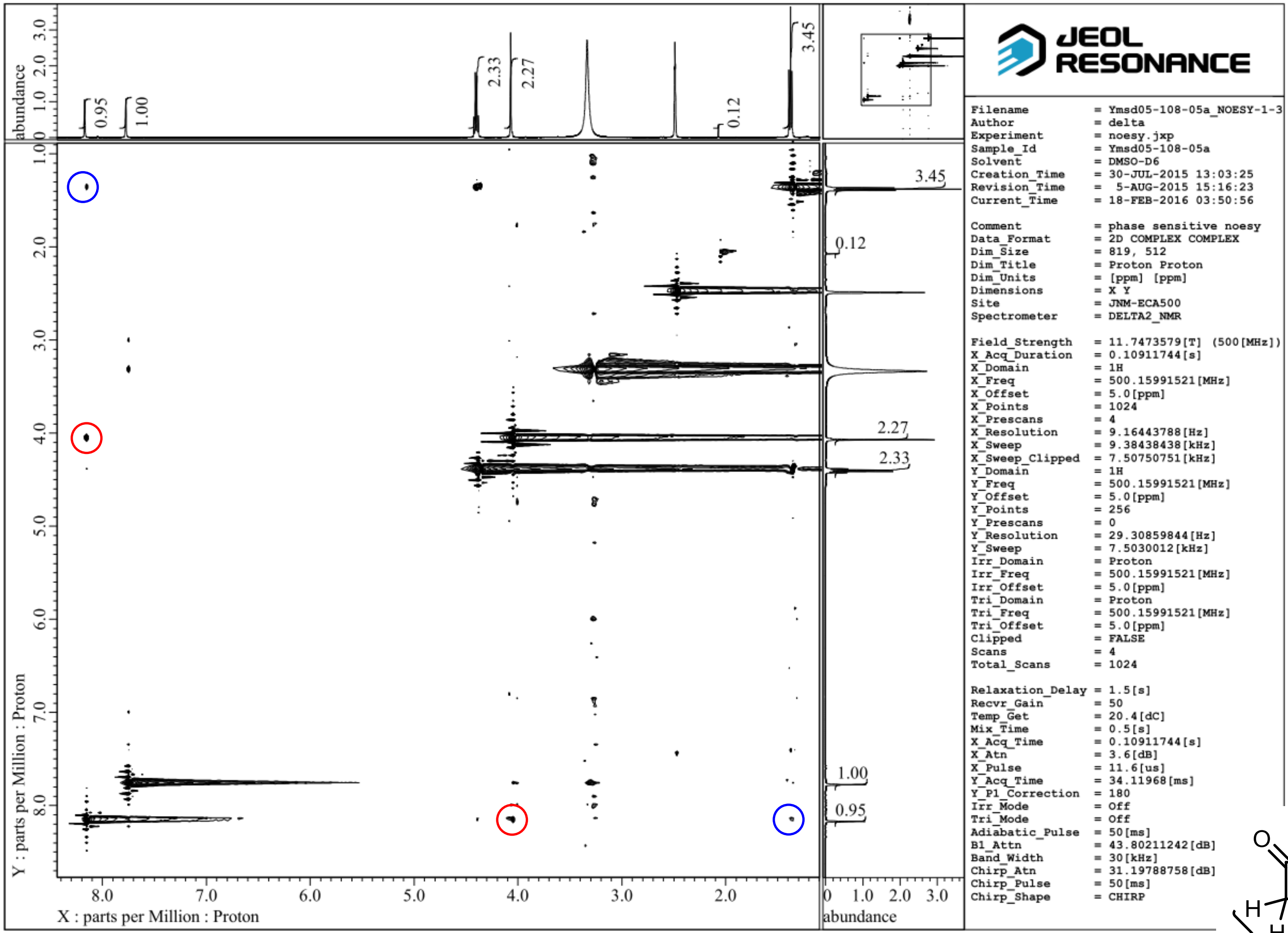
JEOL RESONANCE
Filename = Ymsd05-108-05a_NOESY-1-3
Author = delta
Experiment = noesy.jxp
Sample_Id = Ymsd05-108-05a
Solvent = DMSO-D6
Creation_Time = 30-JUL-2015 13:03:25
Revision_Time = 5-AUG-2015 15:16:23
Current_Time = 18-FEB-2016 03:50:56
Comment = phase sensitive noesy
Data_Format = 2D COMPLEX COMPLEX
Dim_Size = 819, 512
Dim_Title = Proton Proton
Dim_Units = [ppm] [ppm]
Dimensions = X Y
Site = JNM-ECA500
Spectrometer = DELTA2_NMR
Field_Strength = 11.7473579[T] (500[MHz])
X_Acq_Duration = 0.10911744[s]
X_Domain = 1H
X_Freq = 500.15991521[MHz]
X_Offset = 5.0[ppm]
X_Points = 1024
X_Prescans = 4
X_Resolution = 9.16443788[Hz]
X_Sweep = 9.38438438[kHz]
X_Sweep_Clipped = 7.50750751[kHz]
Y_Domain = 1H
Y_Freq = 500.15991521[MHz]
Y_Offset = 5.0[ppm]
Y_Points = 256
Y_Prescans = 0
Y_Resolution = 29.30859844[Hz]
Y_Sweep = 7.5030012[kHz]
Irr_Domain = Proton
Irr_Freq = 500.15991521[MHz]
Irr_Offset = 5.0[ppm]
Tri_Domain = Proton
Tri_Freq = 500.15991521[MHz]
Tri_Offset = 5.0[ppm]
Clipped = FALSE
Scans = 4
Total_Scans = 1024
Relaxation_Delay = 1.5[s]
Recvr_Gain = 50
Temp_Get = 20.4[dC]
Mix_Time = 0.5[s]
X_Acq_Time = 0.10911744[s]
X_Atn = 3.6[dB]
X_Pulse = 11.6[us]
Y_Acq_Time = 34.11968[ms]
Y_P1_Correction = 180
Irr_Mode = Off
Tri_Mode = Off
Adiabatic_Pulse = 50[ms]
B1_Attn = 43.80211242[dB]
Band_Width = 30[kHz]
Chirp_Atn = 31.19788758[dB]
Chirp_Pulse = 50[ms]
Chirp_Shape = CHIRP
abundance
X : parts per Million : Proton
Y : parts per Million : Proton
0.95
1.00
2.33
2.27
0.12
3.45

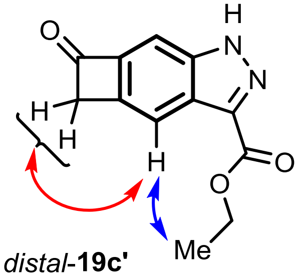
O
H
N
N
H
H
H
O
O
Me

distal-**19c'**

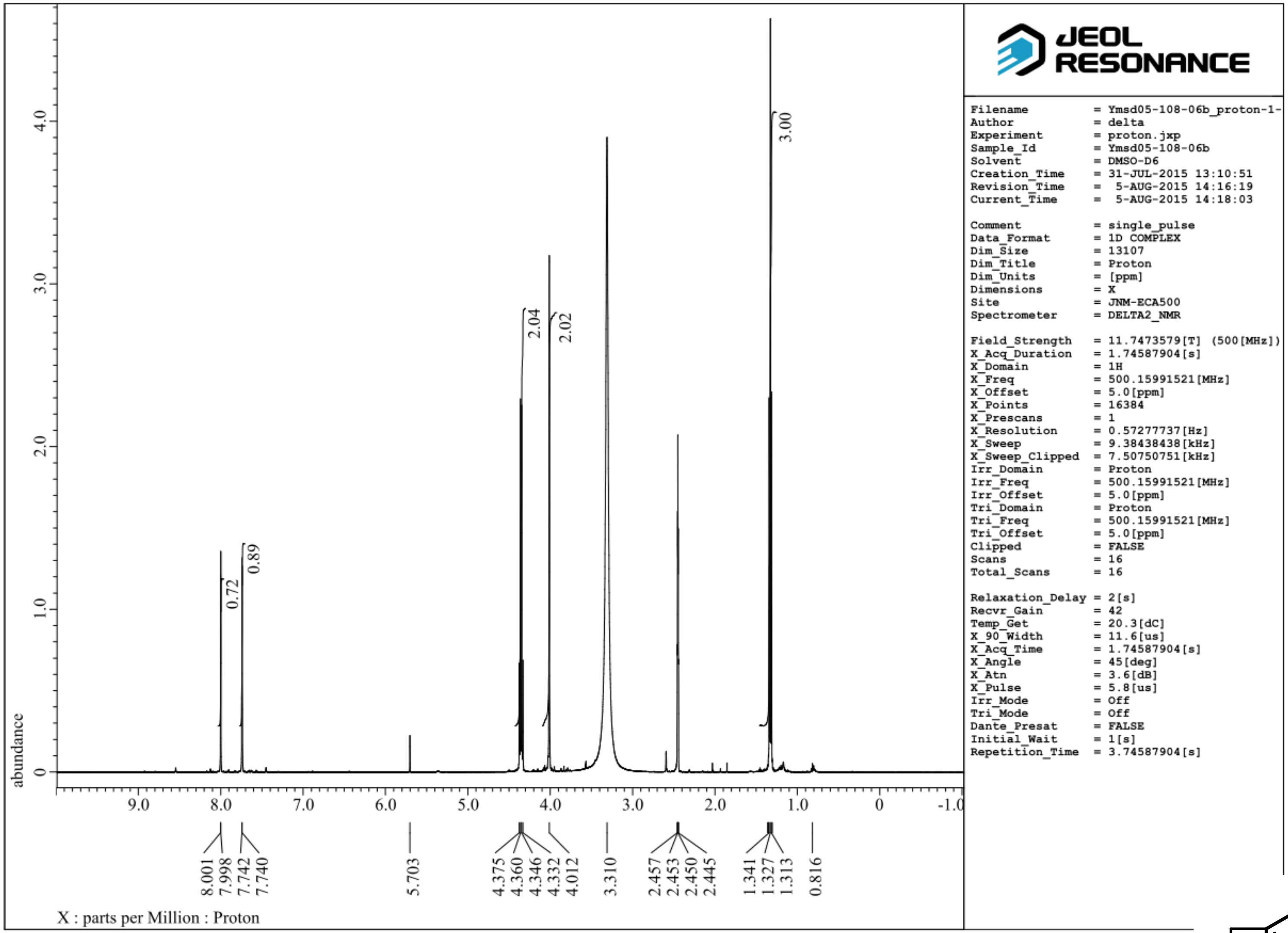

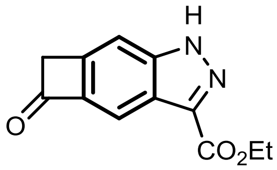

proximal-**19c'**

You created this PDF from an application that is not licensed to print to novaPDF printer (http://www.novapdf.com)

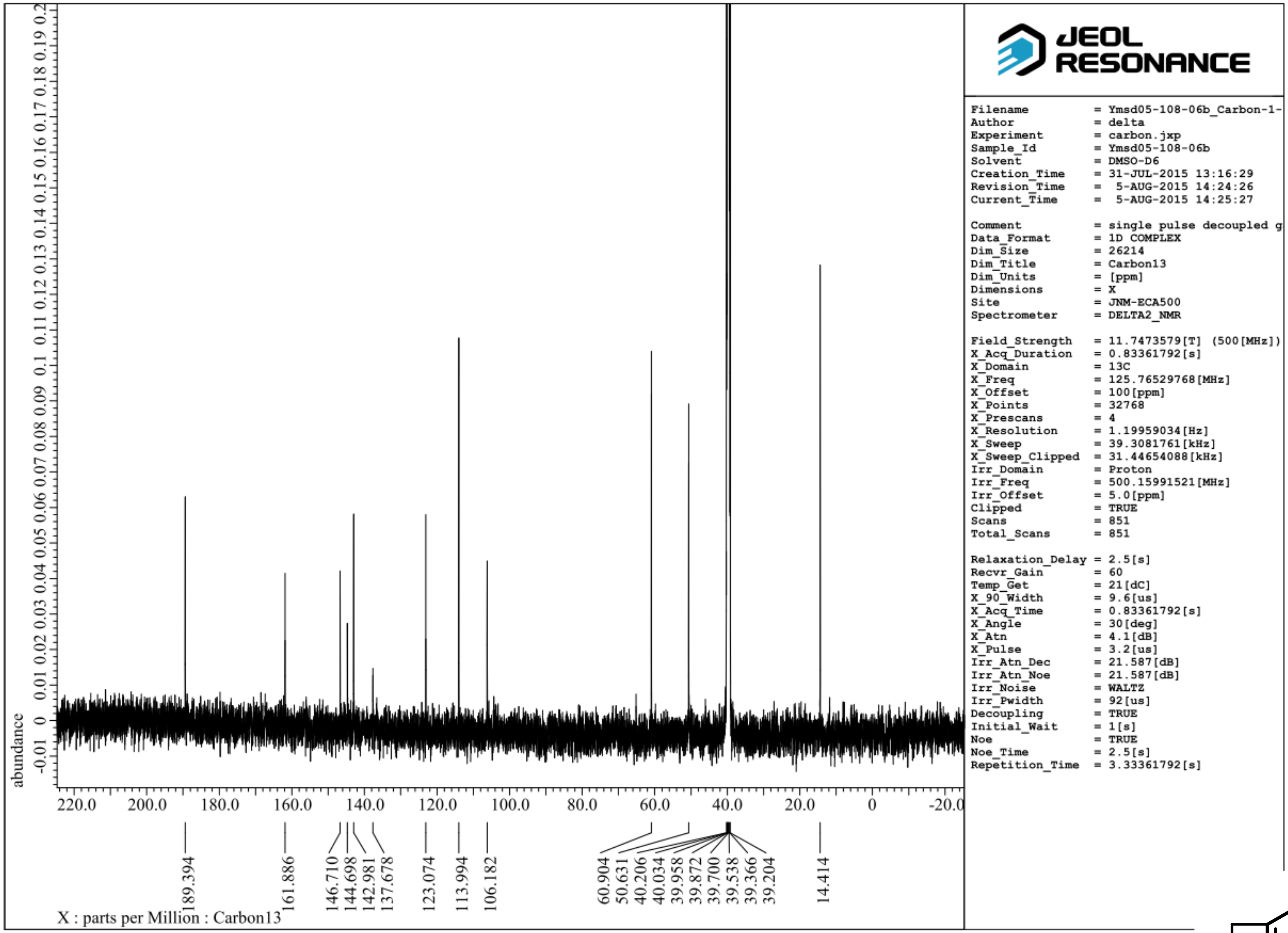

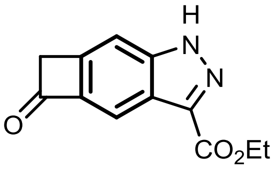

proximal-**19c'**

You created this PDF from an application that is not licensed to print to novaPDF printer (http://www.novapdf.com)

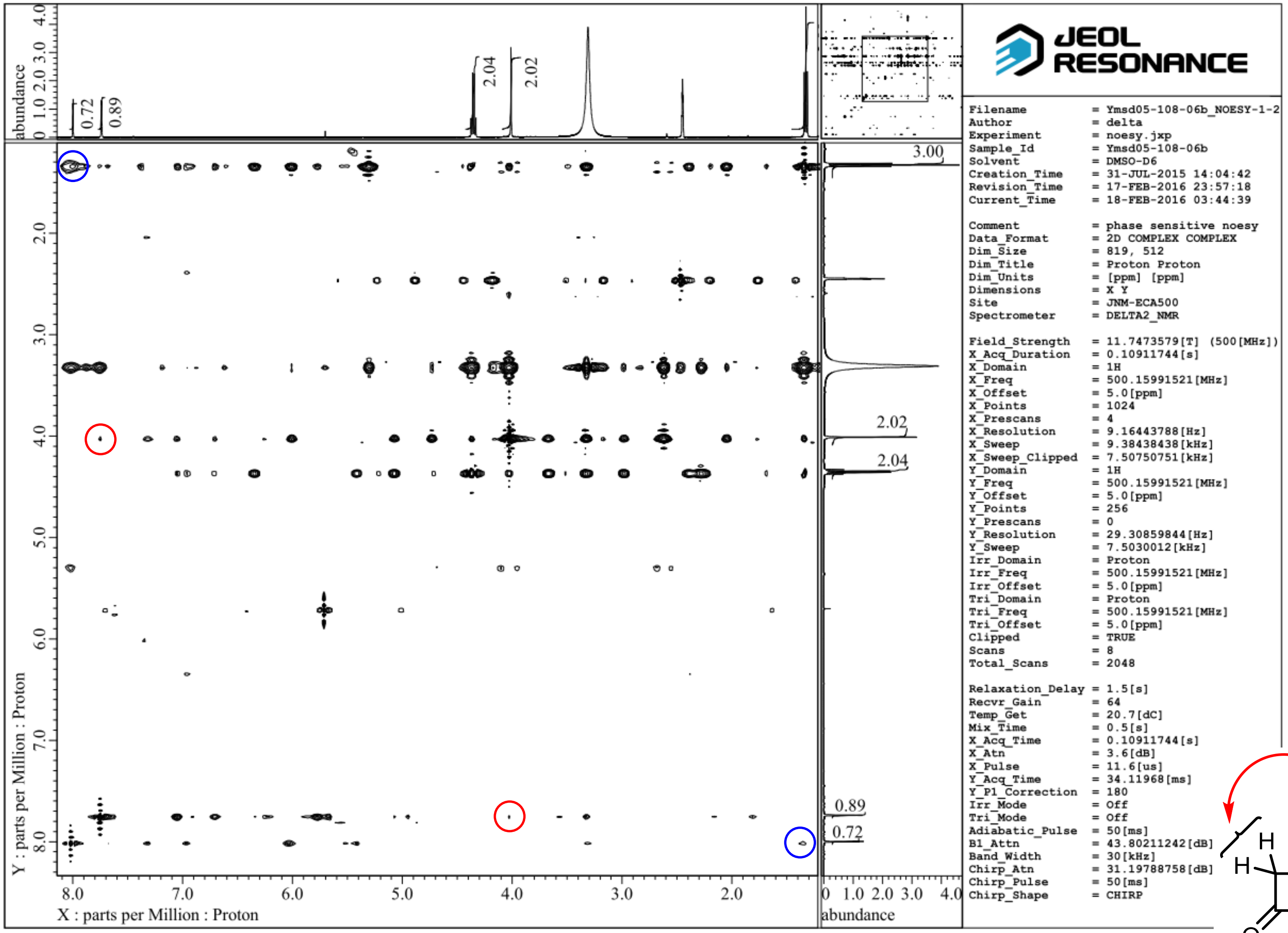

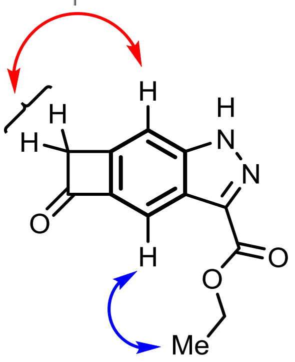

proximal-**19c'**

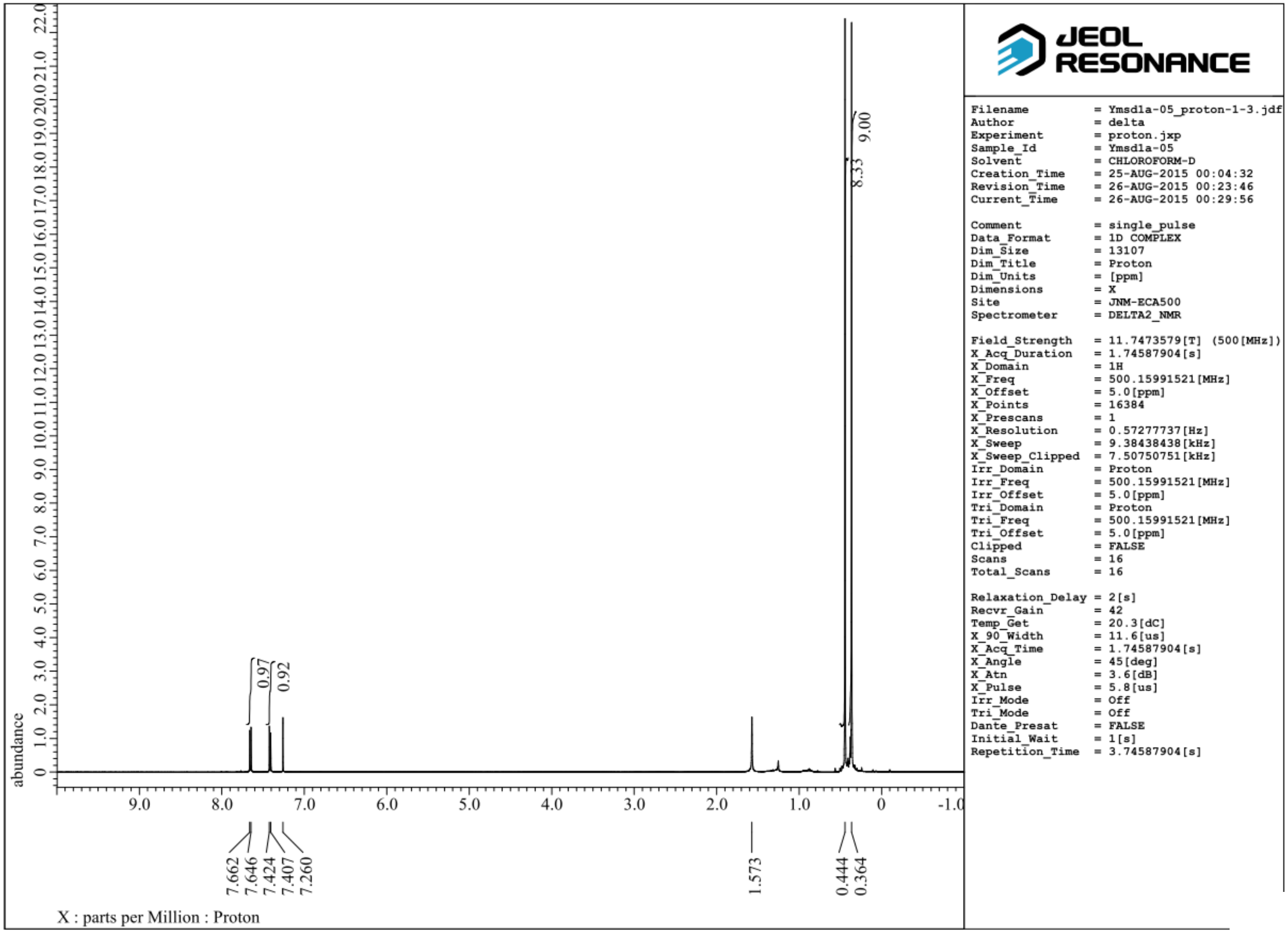

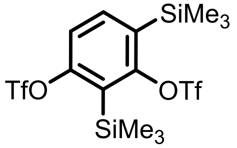

1a

You created this PDF from an application that is not licensed to print to novaPDF printer (http://www.novapdf.com)

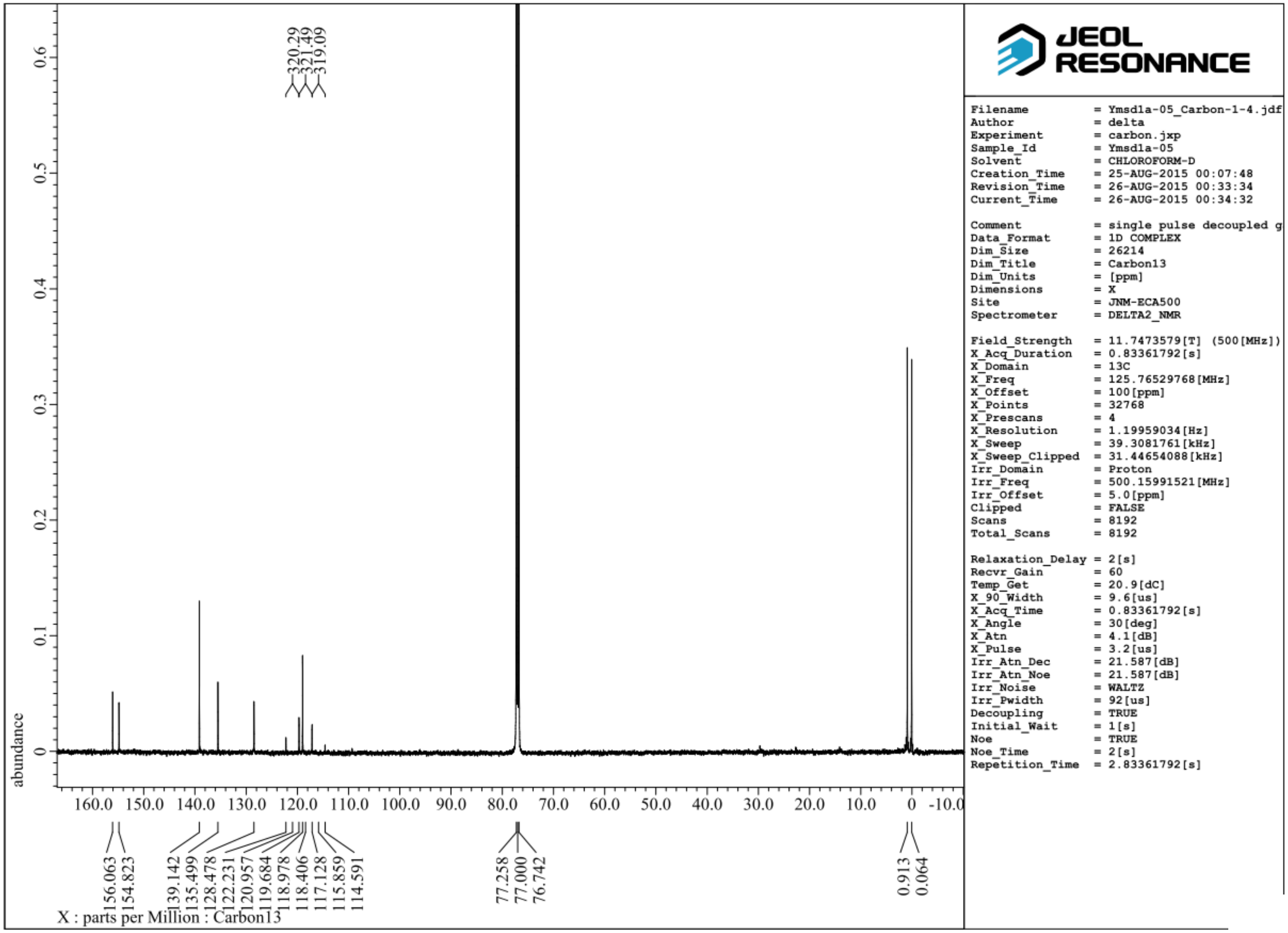

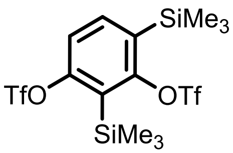

1a

You created this PDF from an application that is not licensed to print to novaPDF printer (http://www.novapdf.com)

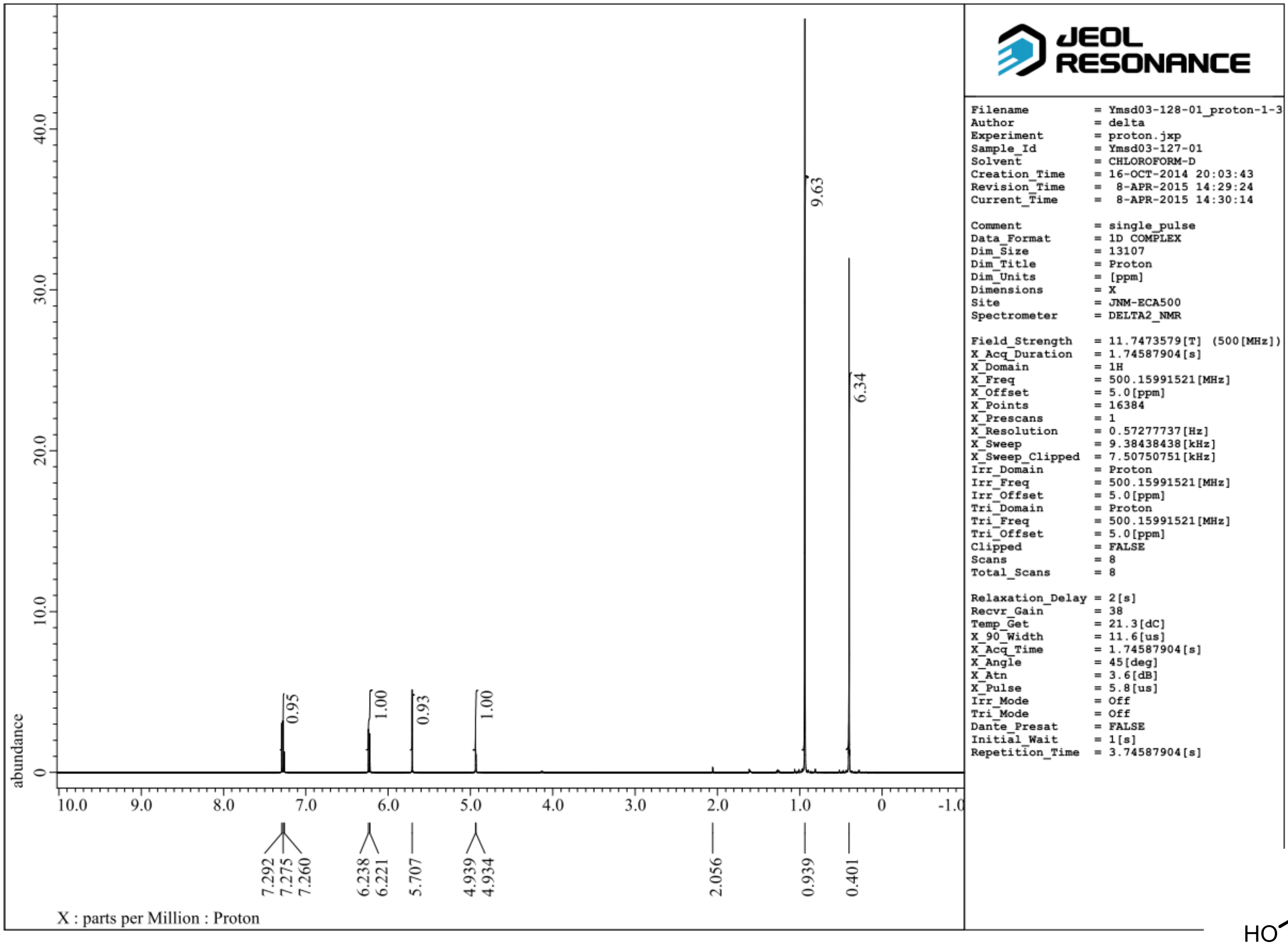

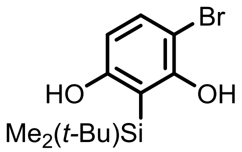

S7

You created this PDF from an application that is not licensed to print to novaPDF printer (http://www.novapdf.com)

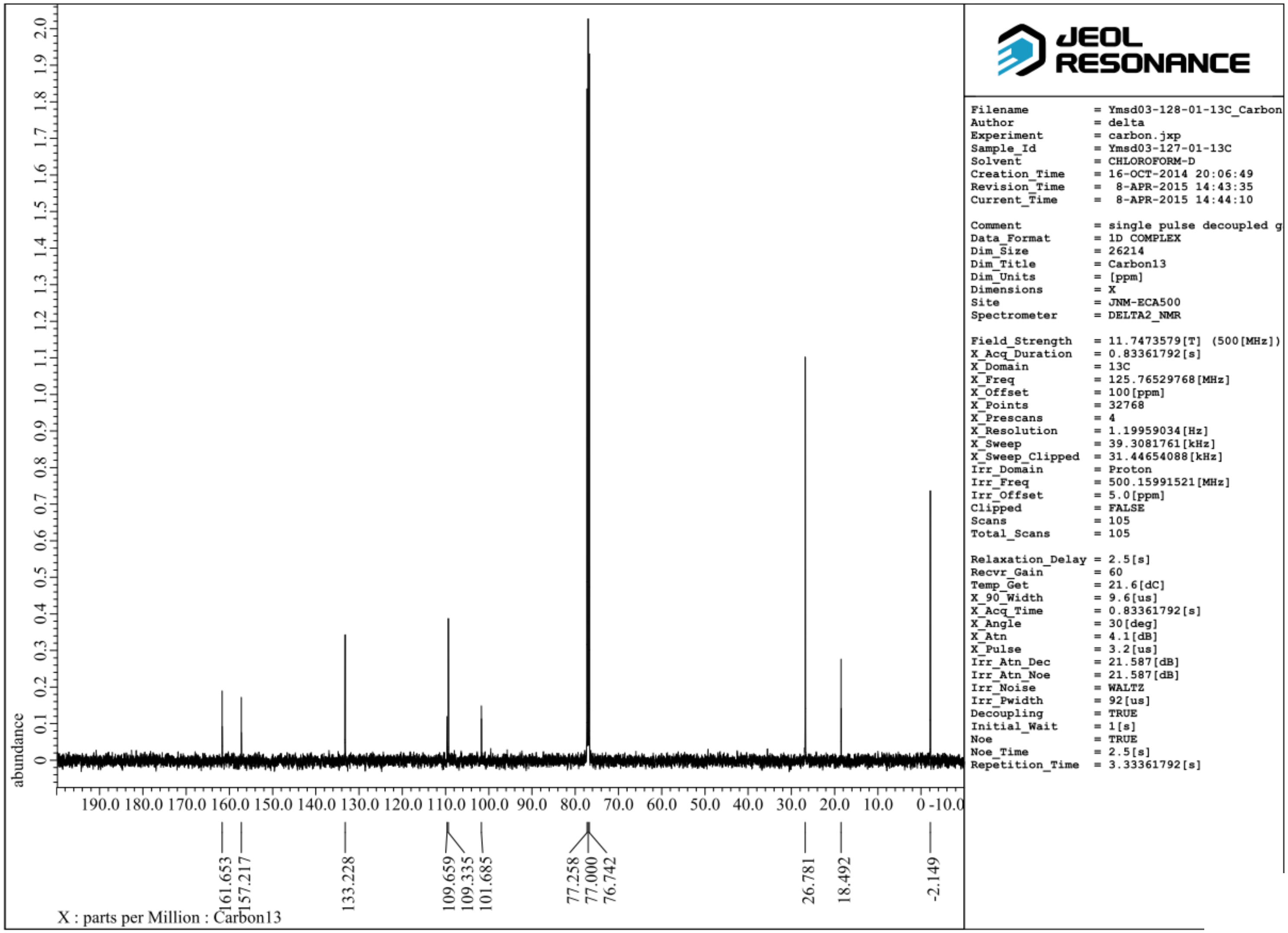

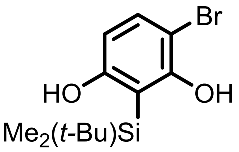

S7

You created this PDF from an application that is not licensed to print to novaPDF printer (http://www.novapdf.com)

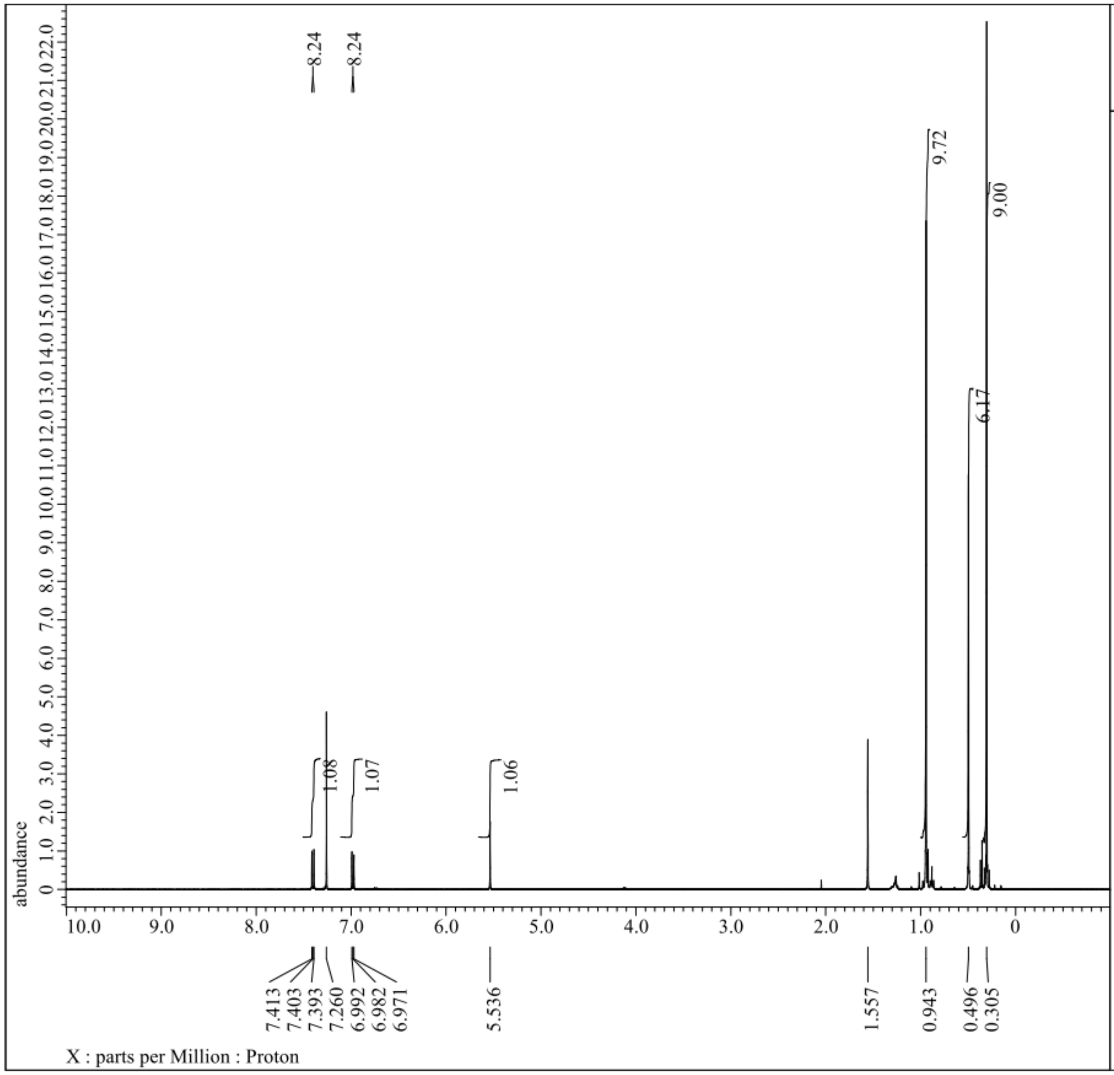

JEOL RESONANCE

```
Filename          = s32-2.jdf
Author            = delta
Experiment        = proton.jxp
Sample_Id         = Ymsd05-007-02
Solvent           = CHLOROFORM-D
Creation_Time     = 28-APR-2015 21:13:44
Revision_Time     = 27-AUG-2015 18:24:07
Current_Time      = 27-AUG-2015 18:24:35

Comment           = single_pulse
Data_Format       = 1D COMPLEX
Dim_Size          = 13107
Dim_Title         = Proton
Dim_Units         = [ppm]
Dimensions        = X
Site              = ECS 400
Spectrometer      = DELTA2_NMR

Field_Strength    = 9.389766[T] (400[MHz])
X_Acq_Duration    = 2.18365952[s]
X_Domain          = 1H
X_Freq            = 399.78219838[MHz]
X_Offset          = 5[ppm]
X_Points          = 16384
X_Prescans        = 1
X_Resolution      = 0.45794685[Hz]
X_Sweep           = 7.5030012[kHz]
X_Sweep_Clipped   = 6.00240096[kHz]
Irr_Domain        = Proton
Irr_Freq          = 399.78219838[MHz]
Irr_Offset        = 5[ppm]
Tri_Domain        = Proton
Tri_Freq          = 399.78219838[MHz]
Tri_Offset        = 5[ppm]
Clipped           = FALSE
Scans             = 8
Total_Scans       = 8

Relaxation_Delay  = 2[s]
Recvr_Gain        = 46
Temp_Get          = 19.4[dC]
X_90_Width        = 11.27[us]
X_Acq_Time        = 2.18365952[s]
X_Angle           = 45[deg]
X_Atn             = 2.4[dB]
X_Pulse           = 5.635[us]
Irr_Mode          = Off
Tri_Mode          = Off
Dante_Presat      = FALSE
Initial_Wait      = 1[s]
Repetition_Time   = 4.18365952[s]
```

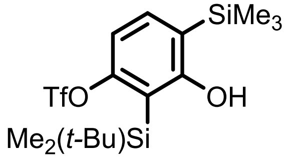

S8

You created this PDF from an application that is not licensed to print to novaPDF printer (http://www.novapdf.com)

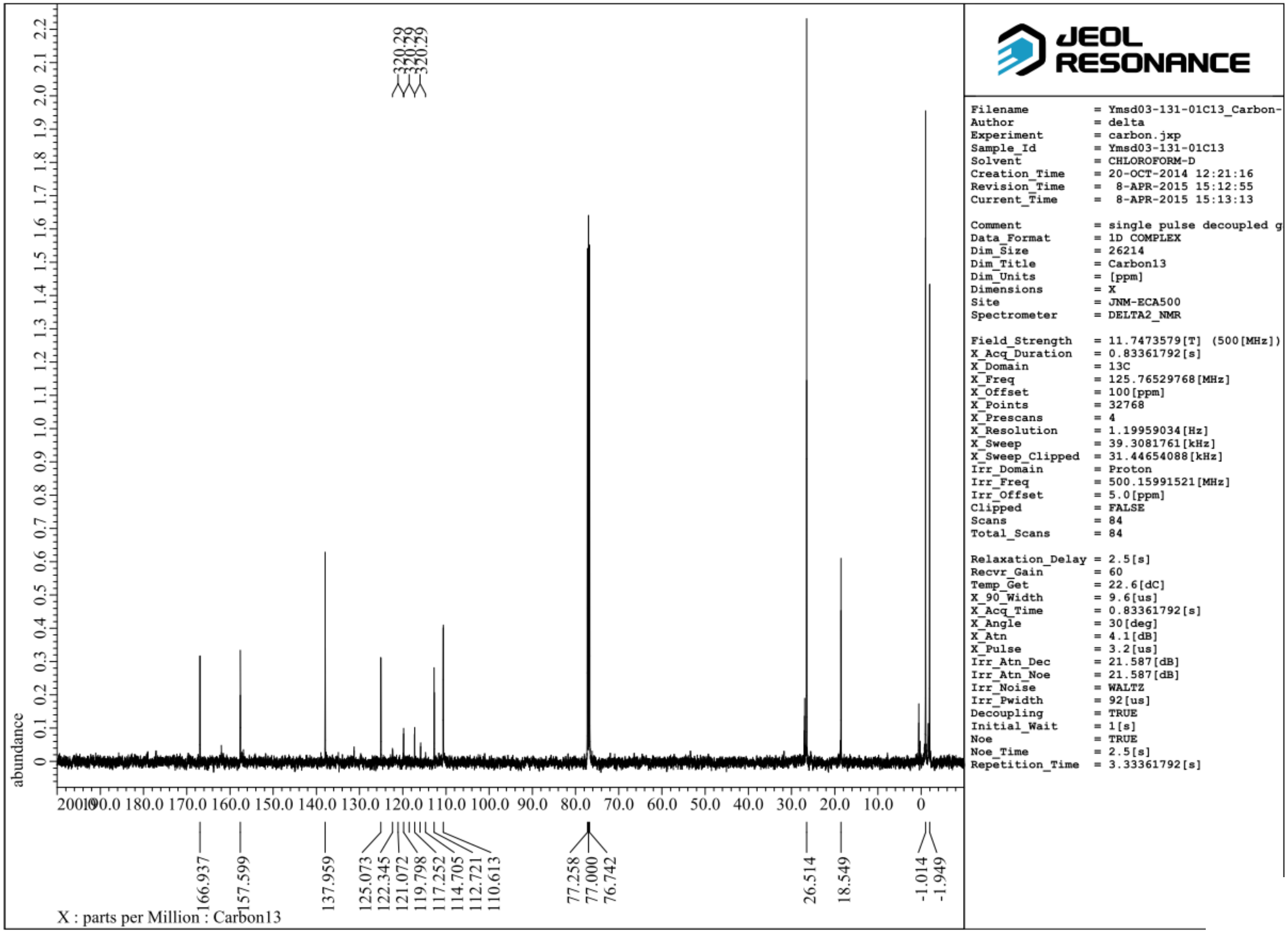

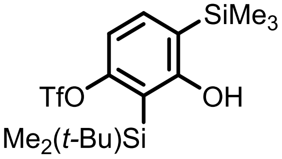

S8

You created this PDF from an application that is not licensed to print to novaPDF printer (http://www.novapdf.com)

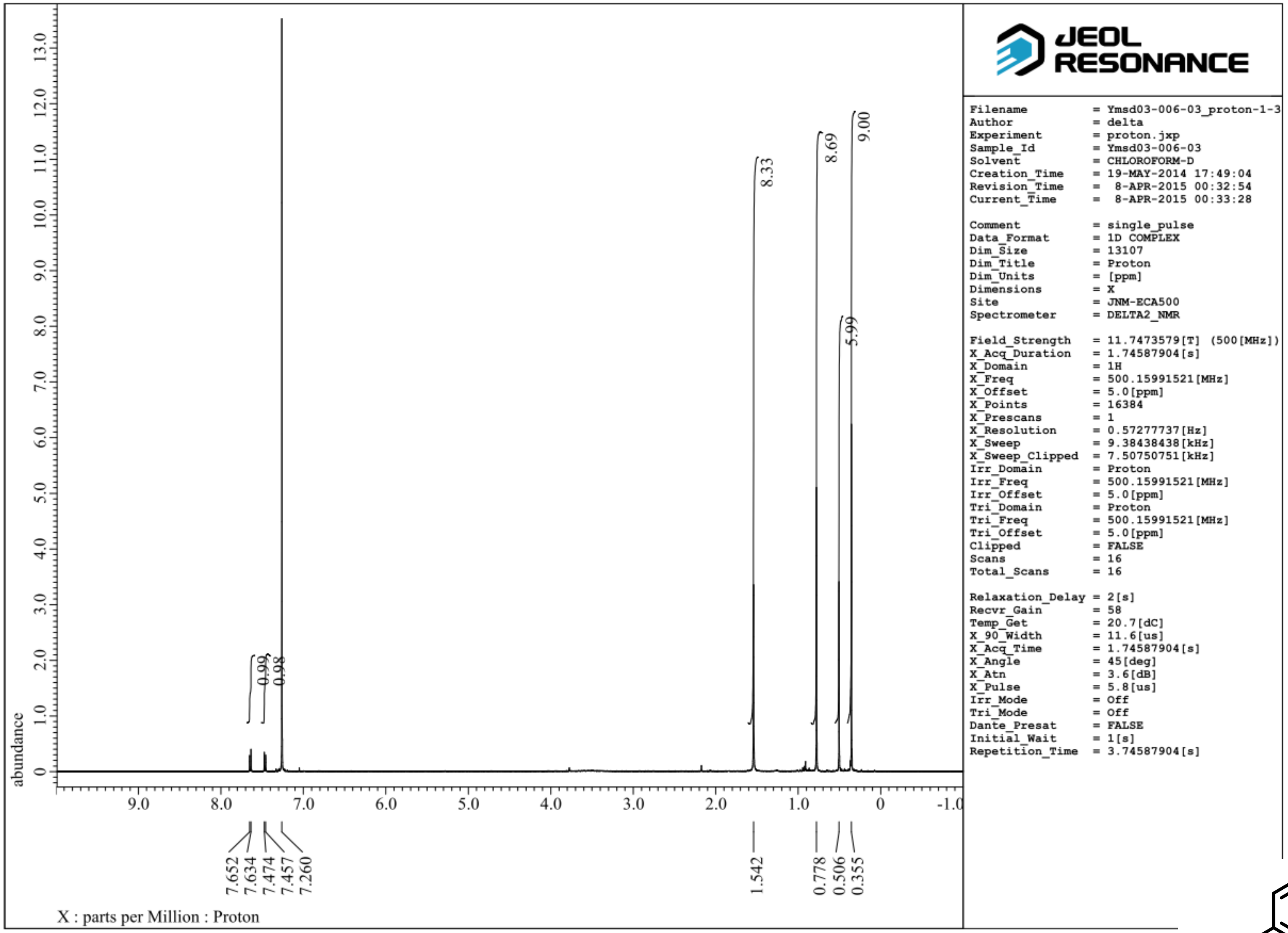

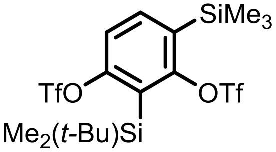

1b

You created this PDF from an application that is not licensed to print to novaPDF printer (http://www.novapdf.com)

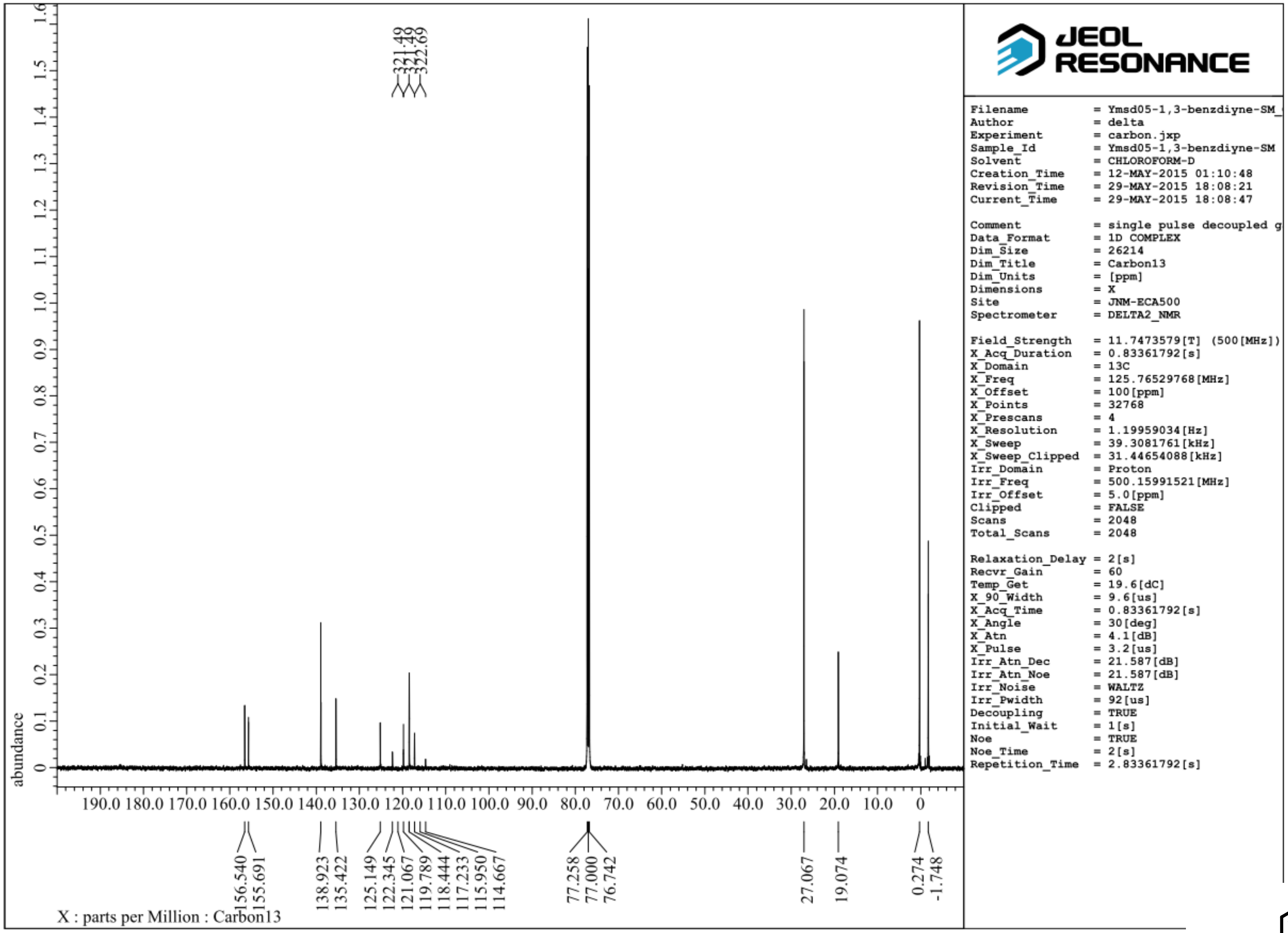

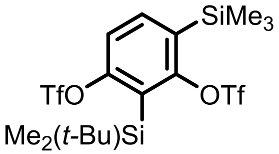

1b

You created this PDF from an application that is not licensed to print to novaPDF printer (http://www.novapdf.com)

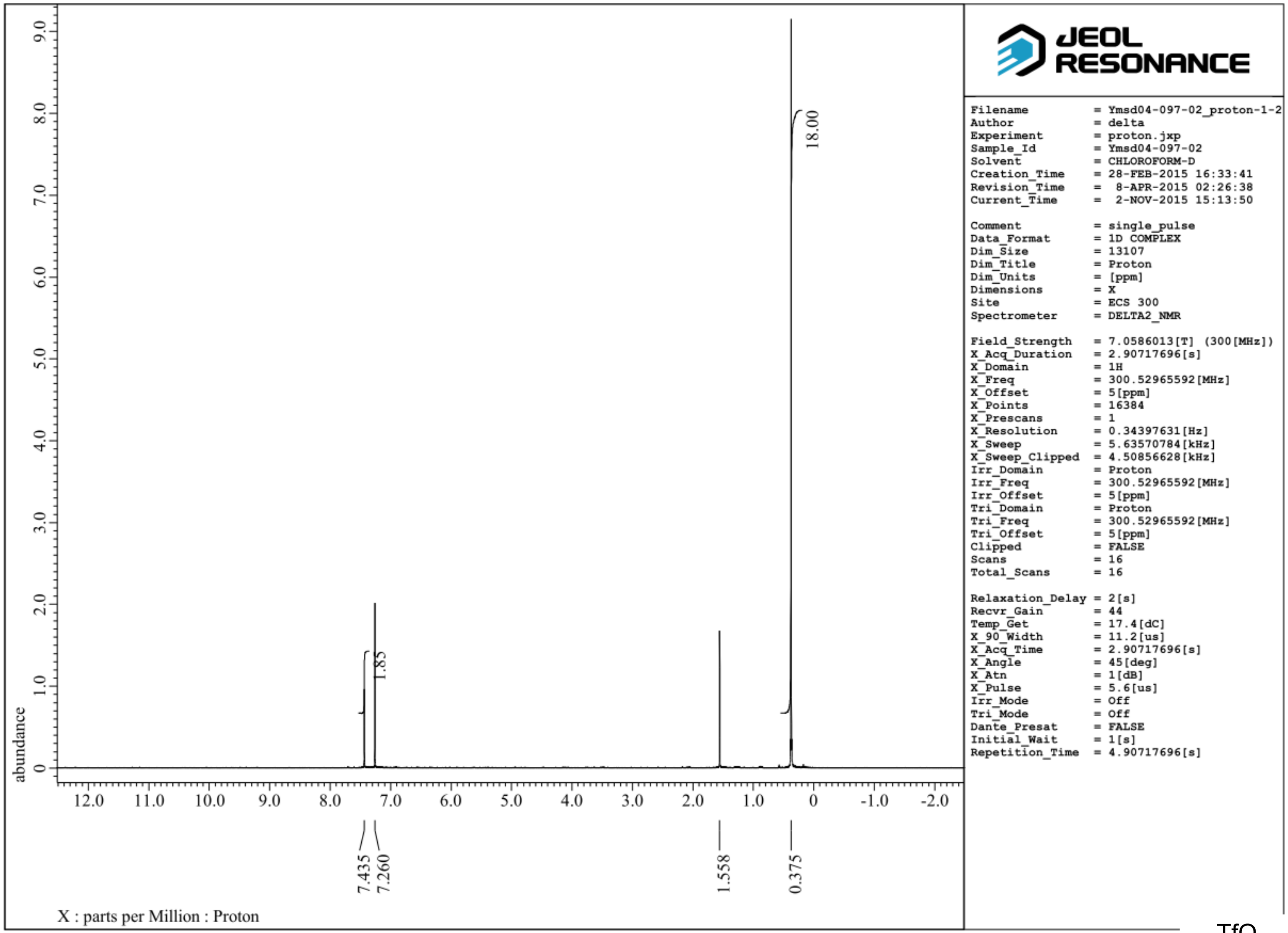

You created this PDF from an application that is not licensed to print to novaPDF printer (http://www.novapdf.com)

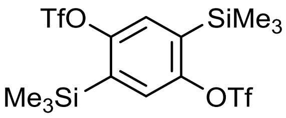

15

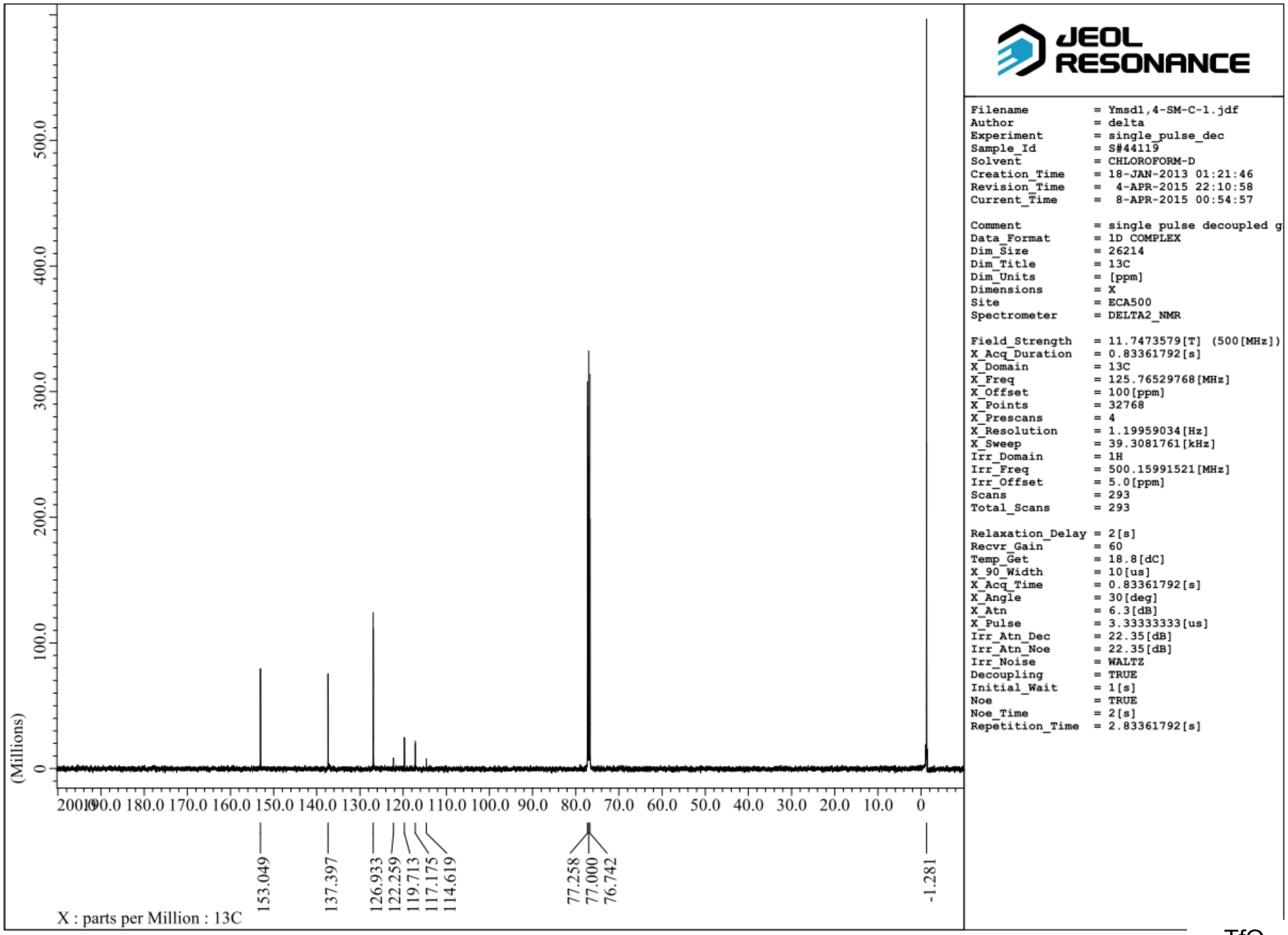

You created this PDF from an application that is not licensed to print to novaPDF printer (http://www.novapdf.com)

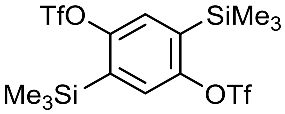

15
